# Targeted *O*‐glycoproteomics explored increased sialylation and identified MUC16 as a poor prognosis biomarker in advanced‐stage bladder tumours

**DOI:** 10.1002/1878-0261.12035

**Published:** 2017-03-02

**Authors:** Sofia Cotton, Rita Azevedo, Cristiana Gaiteiro, Dylan Ferreira, Luís Lima, Andreia Peixoto, Elisabete Fernandes, Manuel Neves, Diogo Neves, Teresina Amaro, Ricardo Cruz, Ana Tavares, Maria Rangel, André M. N. Silva, Lúcio Lara Santos, José Alexandre Ferreira

**Affiliations:** ^1^ Experimental Pathology and Therapeutics Group Portuguese Institute of Oncology Porto Portugal; ^2^ Institute of Biomedical Sciences Abel Salazar University of Porto Portugal; ^3^ Instituto de Investigação e Inovação em Saúde Universidade do Porto Portugal; ^4^ Department of Pathology Hospital Pedro Hispano Matosinhos Portugal; ^5^ Department of Urology Portuguese Institute of Oncology of Porto Portugal; ^6^ Department of Pathology Portuguese Institute of Oncology of Porto Portugal; ^7^ UCIBIO‐REQUIMTE Instituto de Ciências Biomédicas Abel Salazar University of Porto Portugal; ^8^ UCIBIO‐REQUIMTE/Department of Chemistry and Biochemistry Faculty of Sciences University of Porto Portugal; ^9^ Health School of University Fernando Pessoa Porto Portugal; ^10^ Department of Surgical Oncology Portuguese Institute of Oncology Porto Portugal; ^11^ Porto Comprehensive Cancer Center (P.ccc) Portugal

**Keywords:** bladder cancer, glycoproteomics, glycosylation, MUC16, precision medicine, sialic acids

## Abstract

Bladder carcinogenesis and tumour progression is accompanied by profound alterations in protein glycosylation on the cell surface, which may be explored for improving disease management. In a search for prognosis biomarkers and novel therapeutic targets we have screened, using immunohistochemistry, a series of bladder tumours with differing clinicopathology for short‐chain *O*‐glycans commonly found in glycoproteins of human solid tumours. These included the Tn and T antigens and their sialylated counterparts sialyl‐Tn(STn) and sialyl‐T(ST), which are generally associated with poor prognosis. We have also explored the nature of T antigen sialylation, namely the sialyl‐3‐T(S3T) and sialyl‐6‐T(S6T) sialoforms, based on combinations of enzymatic treatments. We observed a predominance of sialoglycans over neutral glycoforms (Tn and T antigens) in bladder tumours. In particular, the STn antigen was associated with high‐grade disease and muscle invasion, in accordance with our previous observations. The S3T and S6T antigens were detected for the first time in bladder tumours, but not in healthy urothelia, highlighting their cancer‐specific nature. These glycans were also overexpressed in advanced lesions, especially in cases showing muscle invasion. Glycoproteomic analyses of advanced bladder tumours based on enzymatic treatments, *Vicia villosa* lectin‐affinity chromatography enrichment and nanoLC‐ESI‐MS/MS analysis resulted in the identification of several key cancer‐associated glycoproteins (MUC16, CD44, integrins) carrying altered glycosylation. Of particular interest were MUC16 STn^+^‐glycoforms, characteristic of ovarian cancers, which were found in a subset of advanced‐stage bladder tumours facing the worst prognosis. In summary, significant alterations in the *O*‐glycome and *O*‐glycoproteome of bladder tumours hold promise for the development of novel noninvasive diagnostic tools and targeted therapeutics. Furthermore, abnormal MUC16 glycoforms hold potential as surrogate biomarkers of poor prognosis and unique molecular signatures for designing highly specific targeted therapeutics.

**Abbreviations**
CD44: cluster of differentiation 44
CEA: carcinoembryonic antigen
CID: collision‐induced dissociation
CSS: cancer‐specific survival
DAGR: Database of Anti‐Glycan Reagents
dST: disialylated sialyl‐T
DTT: 1,4‐dithiothreitol
ESI: electrospray ionization
FFPE: formalin‐fixed, paraffin‐embedded tissue section
HRP: horseradish peroxidase
ITGB1: integrin beta 1
LTQ‐Orbitrap XL: hybrid linear ion trap‐Orbitrap mass spectrometer
MIBC: muscle‐invasive bladder cancer
MS/MS: tandem mass spectrometry
MS: mass spectrometry
MUC16: mucin‐16
nanoLC: nanoliquid chromatography
NMIBC: non‐muscle‐invasive bladder cancer
*O*‐GalNAc: *O*‐N‐acetylgalactosamine
PLA: *in situ* proximity ligation assay
S3T: sialyl‐3‐T
S6T: sialyl‐6‐T
STn: sialyl‐tn
ST: sialyl‐T
TUR: transurethral resection
WHO: World Health Organization

## 1 Introduction

Bladder cancer is the fifth most common cancer in Western society and a growing concern in developing countries, as a result of demographic expansion, increased life expectancy and, in some areas, *Schistosoma haematobium* infection (Antoni *et al*., 2016; Burger *et al*., 2013; Ploeg *et al*., 2009). At the time of clinical diagnosis, most cases are non‐muscle‐invasive bladder cancers (NMIBC), conservatively treated by complete transurethral resection (TUR) (Babjuk *et al*., 2016; Bryan, 2011). In turn, high‐grade tumours are generally characterized by high recurrence rates and elevated risk of progression to muscle invasion (Babjuk *et al*., 2016; Bryan, 2011). Muscle‐invasive bladder cancer (MIBC) is amongst the most common and deadliest genitourinary cancer (Witjes *et al*., 2013). The mainstay treatment includes cisplatin‐based regimens (Witjes *et al*., 2013), which fail to avoid tumour relapse and disease dissemination (Chen *et al*., 2015; Weight *et al*., 2009), urging the introduction of predictive biomarkers and novel therapeutics (Azevedo *et al*., 2015; Ecke, 2015).

Glycosylation is the most common post‐translational modification of proteins, and more than 50% of human proteins are thought to be glycosylated (Ferreira *et al*., 2016a; Spiro, 2002). The patterns of protein glycosylation are cell and tissue specific, closely reflecting the physiological status of cells (Moremen *et al*., 2012; Pinho and Reis, 2015; Spiro, 2002). Therefore, glycosylation changes have been described for several pathological conditions, including cancer (Abou‐Abbass *et al*., 2016; Maverakis *et al*., 2015; Nardy *et al*., 2016). Taking advantage of their cell surface nature, many cancer‐associated glycobiomarkers (CA72‐4; CA19‐9; CA125 which detects MUC16, CEA) have been exploited for noninvasive cancer detection, follow‐up and therapy development (Bottoni and Scatena, 2015; Santos *et al*., 2014; Silva, 2015). Moreover, alterations in glycosylation often render protein glycoforms holding tremendous potential for targeted therapy (Azevedo *et al*., 2015; Fernandes *et al*., 2015; Ferreira *et al*., 2016b). In this context, it has also been long demonstrated that advanced‐stage tumours present significant deregulations in glycosylation pathways, translated by the loss of ABO blood group determinants (Sheinfeld *et al*., 1992). Nevertheless, there are little insights on bladder cancer glycome remodelling accompanying malignant transformation, disease progression and dissemination. Still, few reports have suggested that bladder cancer cells mimic other advanced‐stage solid tumours by promoting a premature stop in protein *O*‐glycosylation (Ferreira *et al*., 2013; Langkilde, 1995; Yamada *et al*., 1988) (biosynthesis pathway depicted in detail in Fig. S1). This causes the accumulation of short‐chain *O*‐glycans as a consequence of (a) altered glycosyltransferase expressions (Vazquez‐Martin *et al*., 2004); (b) mutations in key enzymes involved in *O*‐glycans biosynthesis (Guda *et al*., 2009); (c) mislocalization of glycosyltransferases in secretory organelles (Rivinoja *et al*., 2009); (d) metabolic deregulations (Pinho and Reis, 2015), amongst other factors. The accumulation of short‐chain *O*‐N‐acetylgalactosamine (*O*‐GalNAc) glycans at the cell surface of tumour cells affects their adhesive properties while promoting their invasive, metastatic and angiogenic potential, as well as immune scape (Bapu *et al*., 2016; Carrascal *et al*., 2014; Ferreira *et al*., 2013). Moreover, it may modulate intracellular signalling and activate key oncogenic pathways (Bapu *et al*., 2016). Reinforcing these notions, we have previously demonstrated that 70% of advanced‐stage bladder tumours express the cancer‐associated carbohydrate antigen sialyl‐Tn (Costa *et al*., 2015; Ferreira *et al*., 2013); conversely, the healthy urothelium and most superficial tumours do not (Ferreira *et al*., 2013). STn expression favours cell invasion, motility (Ferreira *et al*., 2013; Peixoto *et al*., 2016) and immune tolerance (Carrascal *et al*., 2014) and has been associated with poor overall survival (Costa *et al*., 2015). In addition, solid tumours often accumulate the more complex T antigen and its sialylated form ST, whose overexpression has also been associated with poor prognosis (Dow *et al*., 1989; Videira *et al*., 2009). However, the ST antigen comprises a heterogeneous group of mono‐ (sialyl‐6‐T: S6T; sialyl‐3‐T: S3T) and disialylated glycoforms that remain to be individually evaluated in cancer. Building on these insights, we aimed to screen bladder tumours and corresponding urine samples for the above‐mentioned cancer‐associated short‐chain *O*‐glycoproteins, envisaging a molecular rationale for the development of novel noninvasive diagnostic tools and highly specific targeted therapeutics towards precision medicine.

## 2 Materials and methods

### 2.1 Patient and sampling

The screening of cancer‐associated short‐chain *O*‐glycans (Tn and STn; T and ST, S6T and S3T) was performed on 47 formalin‐fixed, paraffin‐embedded tissue sections prospectively collected from 37 male and 10 female patients, mean age of 70 years (ranging 45–89 years old), who underwent bladder surgery in the Portuguese Institute for Oncology of Porto (IPO‐Porto, Portugal), between July 2011 and May 2012. Based on urothelial carcinoma grading and staging criteria of the World Health Organization (Eble *et al*., 2004), three different groups were considered: low‐grade (LG; *n* = 17), high‐grade (HG; *n* = 12) non‐muscle‐invasive papillary bladder cancers (NMIBC) and muscle‐invasive (*n* = 18) bladder cancers (MIBC). For molecular target validation, a larger subset of samples was used, composed by a retrospective series of 176 bladder cancer cases (74 NMIBC and 102 MIBC). In NMIBC, the male/female gender ratio was of 61 : 13 and the median age was 64 years. The male/female gender ratio in MIBC was of 9 : 1 and the median age was 71 years. Forty cases were considered stage Ta, 34 stage T1, 25 stage T2, 48 stage T3 and 29 stage T4 (for further analysis, T1‐ to T4‐staged tumours were compared against Ta‐staged tumours). All MIBC patients were treated with cystectomy, 27 of which were also treated with adjuvant chemotherapy (cisplatin+gemcitabine). All tumour samples were revised by a pathologist (TA) according to 2004 WHO grading criteria. As such, 38 cases were considered low‐grade and 138 high‐grade tumours. All procedures were performed under the approval of Institutional Ethics Committee of IPO‐Porto after obtaining informed patient's consent. All clinicopathological information was obtained from patient's clinical records.

### 2.2 Immunohistochemistry

FFPE urothelium sections were screened for the glycans of interest by immunohistochemistry using the avidin/streptavidin peroxidase method, as described by Ferreira *et al*. (2013). The expression of the Tn, sialyl‐Tn and T antigens was directly evaluated using in‐house mouse monoclonal antibodies 1E3, TKH2 and 3C9, respectively (Clausen *et al*., 1988; Kjeldsen *et al*., 1988; Marcos *et al*., 2004). All available information on these antibodies including immunogens, specificity and associated bibliography is deposited in the Database of Anti‐Glycan Reagents (https://ccr2.cancer.gov/resources/Cbl/Tools/Antibody/About.aspx) (Sterner *et al*., 2016). The expression of sialylated T antigens (mono‐ and disialylated glycoforms) was determined by comparing histological sections probed for the T antigen before and after digestion with an α‐neuraminidase from *Clostridium perfringens* (Sigma Aldrich, St. Louis, MO, USA). The S3T antigen expression was determined by comparing histological sections probed for the T antigen before and after digestion with an α‐(2,3)‐neuraminidase from *Streptococcus pneumonia* (Sigma Aldrich) according to Fig. S1A. The S6T antigen expression was accessed by comparing histological sections probed for STn before and after digestion with a recombinant β‐(1,3)‐galactosidase from *Xanthomonas campestris* (R&D systems, Minneapolis, MN, USA) according to Fig. S1B. The chromogen 3,3‐diaminobenzidine tetrahydrochloride (ImmPACT DAB; Vector Laboratories, Burlingame, CA, USA) was used to visualize antibody‐binding sites, and sections were counterstained with Harris's haematoxylin. Negative controls were performed by replacing the primary antibody with 5% bovine serum albumin (BSA). Positive controls were known positive tissues for the antigens under study. Bladder tumours and metastasis were also screened for MUC16 using rabbit anti‐human CA‐125 monoclonal antibody EPR1020 (1 : 200 in PBS; Abcam, Cambridge, UK) at room temperature for 1 h. Furthermore, tumour tissues were screened for CD44 using anti‐CD44 (1 : 150 in PBS; EPR1013Y; Abcam) and anti‐ITGB1 (1 : 100 in PBS, A‐4 clone; Santa Cruz Biotechnology, Dallas, TX, USA). In addition, prior to glycoproteomics studies, FFPE tissues were screened for blood group A determinants using mouse monoclonal anti‐human blood group A antibody HE‐195 (1 : 100 in PBS; Thermo Fisher Scientific, Waltham, MA, USA) after 1‐h incubation at 37 °C. This approach aimed to elect negative cases for downstream glycoproteomics studies. The immunoreactive tissue sections were assessed double‐blindly through light microscopy by two independent observers (LL and DF) and validated by an experienced pathologist (TA). Although the interobserver agreement was high (*k* = 0.961, *P* < 0.001), discordant readings were re‐analysed using a double‐headed microscope (Olympus BX46; Olympus Corporation, Tokyo, Japan), and consensus was reached. A semiquantitative approach was established to score the immunohistochemical labelling based on the percentage of positively stained cells. For the evaluation of glycans, the tissues were categorized as follows: negative (−), when no staining was observed; positive (+), 1–19% of positive cells; positive (++), 20–49% of positive cells; positive (+++), 50–79% of positive cells; positive (++++), 80–100% of positive cells. Regarding MUC16 evaluation, samples were classified as positive whenever the antigen was present or negative in the absence of the antigen.

### 2.3 Glycoprotein extraction and enrichment

Proteins were extracted from FFPE STn‐positive bladder tumours of male MIBC patients (*n* = 5) using Qproteome FFPE tissue kit (Qiagen, Hilden, Germany) according to the supplier's instructions. To avoid false positives in downstream glycoprotein enrichment steps based on GalNAc affinity chromatography, only Tn and blood group A antigen‐negative tumours were included in this study. Five 10‐μm‐thick tumour sections from each patient were used for this propose. The amount of protein in each extract was estimated with RC DC protein assay kit (Bio‐Rad, Hercules, CA, USA). The extracted glycoproteins were then blotted for STn as previously described (Peixoto *et al*., 2016) to confirm the presence of glycoproteins yielding the STn antigen. For proteomics analysis, 20 μg of the protein pool was separated by 4–16% gradient SDS/PAGE under reducing conditions; the bands were excised from the gels; and proteins were reduced with 5 mm 1,4‐dithiothreitol (Sigma Aldrich) for 40 min at 60 °C, alkylated with 10 mm iodoacetamide (Sigma Aldrich) for 45 min in the dark and digested with trypsin (Promega, Madison, WI‎, USA) *in situ* for MS analysis (Ferreira *et al*., 2011) (according to Fig. S3A). For glycoproteomics analysis, approximately 1 mg of total protein was precipitated by the addition of four volumes of −20 °C acetone to a sample extract and dried under vacuum on a speedvac. The extract was resuspended in 0.05% RapiGest (Waters, Milford, MA, USA), digested with PNGase F (10 U PNGase F from *Elizabethkingia meningoseptica*; Sigma Aldrich) to remove *N*‐glycans, facilitating downstream trypsin digestion and peptide identification. Subsequently, the extract was subjected to neuraminidase treatment [10 U *C. perfringens* neuraminidase type VI (Sigma Aldrich)] to remove neuraminic acids from STn, thereby exposing the GalNAc residue (Tn antigen). The sample was then loaded on 300 μL of agarose‐bound *Vicia villosa* agglutinin (VVA; Vector Laboratories) column to enrich the extract in Tn‐expressing glycoproteins. The column was then washed with 10 column volumes of 0.4 m glucose in LAC A buffer (20 mm Tris/HCl pH 7.4, 150 mm NaCl, 1 m urea, 1 mm CaCl_2_, MgCl_2_, MnCl_2_ and ZnCl_2_) followed by 1 mL 50 mm NH_4_HCO_3_ (all reagents were purchased from Sigma Aldrich). The glycoproteins were then eluted by 4 × 500 μL 0.05% RapiGest (Waters) with heating to 90 °C for 10 min. The glycoprotein fraction was then directly reduced, alkylated and digested with trypsin as previously described (Ferreira *et al*., 2011) (according to Fig. S3B).

### 2.4 NanoLC‐ESI‐LTQ‐Orbitrap‐CID‐MS/MS

A nanoLC system (3000 Ultimate nano‐LC; Dionex, Sunnyvale, CA, USA) was coupled online to a LTQ‐Orbitrap XL mass spectrometer (Thermo Scientific, Waltham, MA, USA) equipped with a nano‐electrospray ion source (EASY‐Spray source; Thermo Scientific). Eluent A was aqueous formic acid (0.2%), and eluent B was formic acid (0.2%) in acetonitrile. Samples (20 μL) were injected directly into a trapping column (C18 PepMap 100, 5 μm particle size) and washed over with an isocratic flux of 95% eluent A and 5% eluent B at a flow rate of 30 μL·min^−1^. After 3 min, the flux was redirected to the analytical column (EASY‐Spray C18 PepMap, 100 Å, 150 mm × 75 μm ID and 3 μm particle size) at a flow rate of 0.3 μL·min^−1^. Column temperature was set at 35 °C. Peptide separation occurred using a linear gradient of 5–40% eluent B over 117 min, 50–90% eluent B over 5 and 5 min with 90% eluent B. In order to favour the separation and identification of peptides presenting high hydrophobicity, samples were also analysed with a two‐step gradient protocol: 5–35% eluent B over 37 min, 35–65% eluent B over 80 min, followed by 65–90% eluent B over 5 min and 5 min with 90% buffer B. The mass spectrometer was operated in the positive ion mode, with a spray voltage of 1.9 kV and a transfer capillary temperature of 250 °C. Tube lens voltage was set to 120 V. MS survey scans were acquired at an Orbitrap resolution of 60 000 for an *m*/*z* range from 300 to 2000. Tandem MS (MS/MS) data were acquired in the linear ion trap using a data‐dependent method with dynamic exclusion: the top six most intense ions were selected for collision‐induced dissociation (CID). CID settings were 35% normalized collision energy, 2‐Da isolation window, 30‐ms activation time, and an activation Q of 0.250. A window of 90 s was used for dynamic exclusion. Automatic gain control was enabled and target values were 1.00e+6 for the Orbitrap and 1.00e+4 for LTQ MSn analysis. Data were recorded with xcalibur software version 2.1 (Thermo Fisher Scientific).

### 2.5 MS/MS data curation

Data were analysed automatically using the SequestHT search engine with the Percolator algorithm for validation of protein identifications (Proteome Discoverer 1.4; Thermo Scientific). Data were searched against the human proteome obtained from the SwissProt database on 22/11/2015, selecting trypsin as the enzyme and allowing for up to two missed cleavage sites, a precursor ion mass tolerance of 10 p.p.m. and 0.6 Da for product ions. Carbamidomethylcysteine was selected as a fixed modification, while oxidation of methionine (+15.994u), modification of serine and threonine with HexNac (+203.08u), and/or HexNacNeuNac (STn) (+494.17u), considering the possibility of partially inefficient α‐neuraminidase treatment, and/or T (+365.13u) were defined as variable modifications. For whole tumour proteome analysis, only high confidence peptides were considered. In glycoproteomics studies, due to the high lability of the sugar moieties under CID conditions, and the consequent difficulty in identifying modified peptides, Sequest results of low confidence peptides were also considered. Protein grouping filters were thus set to consider glycosylations with low confidence and ΔCn better than 0.05. The strict maximum parsimony principle was applied. A protein filter counting peptides only on top‐scored proteins was also set. Peptides were filtered for Xcorr ≥ 1.0 and ΔCn ≤ 0.05. Cytoplasm membrane proteins with at least one annotated glycosylation site were selected and the modifications were validated manually. Membrane proteins were sorted using NetOGlyc version 4.0 (http://www.cbs.dtu.dk/services/NetOGlyc/) (Steentoft *et al*., 2013) to generate the final protein list. Protein molecular and biological functions were interpreted using Panther (Mi *et al*., 2016).

### 2.6 *In situ* proximity ligation assays on tissue sections

The simultaneous detection of mucin‐16 (MUC16), ITGB1 and CD44 STn^+^‐glycoforms was made by *in situ* proximity ligation assays (PLA) using the Duolink *in situ* detection reagents Brightfield and Red, respectively (Olink Bioscience, Uppsala, Sweden) according to the manufacturer's instructions and based on previous reports (Campos *et al*., 2015; Ricardo *et al*., 2015). Briefly, FFPE tissues were deparaffinized, rehydrated and subjected to acid‐ and heat‐induced antigen retrieval, followed by incubation with 3% hydrogen peroxide and blocking solution in a humidity chamber, as previously described (Ferreira *et al*., 2013). MUC16 was detected by direct PLA using monoclonal antibody CA125 (clone M11; DAKO, Santa Clara, CA, USA) conjugated with PLA probe PLUS (concentration of 0.005 mg·mL^−1^) and B72.3 monoclonal antibody against STn, which showed similar recognition but lower background when compared with TKH2 monoclonal antibody used for immunohistochemistry, with PLA probe MINUS (concentration of 5 ng·mL^−1^). Antibodies were conjugated according to the instructions of Duolink *in situ* Probemaker and were hybridized for 1 h at 37 °C. Next, ligation was performed for 30 min at 37 °C and amplification was carried out for 120 min at 37 °C to produce rolling circle products, followed by incubation with horseradish peroxidase (HRP)‐labelled probes and addition of the chromogen. Finally, sections were counterstained with haematoxylin, dehydrated, cleared and mounted for optical microscope analysis. Regarding the indirect PLA for ITGB1 and CD44, FFPE tissues were incubated with anti‐CD44 (EPR1013Y; Abcam) and anti‐ITGB1 (A‐4 clone; Santa Cruz Biotechnology) overnight at 4 °C in a humidity chamber. Then, the PLA probes anti‐rabbit MINUS and anti‐mouse PLUS were both added and sections were incubated at 37 °C for 1 h. The following steps of ligation and amplification were performed in the same conditions of the direct PLA. Sections were incubated with 4′,6‐diamidino‐2‐phenylindole for 10 min at room temperature and mounted for fluorescence microscopy. PLA results were evaluated by two observers and validated by an experienced pathologist, who independently registered cytolocalization of staining. PLA validation was conducted using MUC16‐expressing cell lines OVCAR3 wild‐type which do not express STn (Kui *et al*., 2003) and sequential ovarian cancer tissue sections showing MUC16 and STn colocalization by immunohistochemistry (Ricardo *et al*., 2015).

### 2.7 *MUC16* transcription in bladder tumours

RNA was isolated from FFPE tissue samples using the Absolutely RNA FFPE Kit (Stratagene, San Diego, CA, USA), as previously described (Lima *et al*., 2014). Up to 2 mg of total RNA was reverse‐transcribed with random primers, using the ‘High Capacity cDNA Reverse Transcription Kit’ (Applied Biosystems, Foster City, CA, USA). Real‐time PCR amplification of cDNA samples was performed in a StepOne Real‐Time PCR System (Applied Biosystems) using TaqMan Gene Expression Master Mix, primers and probes provided by Applied Biosystems. *MUC16* expression was measured with TaqMan expression assay (ID: Hs01065189_m1) from Applied Biosystems. The raw −Δ*C*_t_ was used to analyse *MUC16* expression and therefore used as an estimate of the mRNA relative levels. Δ*C*_t_ stands for the difference between the cycle threshold (*C*_t_) of the amplification curve of the target gene and that of the *GAPDH* (ID: Hs03929097_g1). The efficiency of the amplification reaction for each primer/probe is more than 95%, as determined by the manufacturer.

### 2.8 Immunoprecipitation for CD44 and ITGB1

CD44 and ITGB1 were immunoprecipitated from total protein extracts (IP) with anti‐CD44 (EPR1013Y; Abcam) and anti‐ITGB1 (A‐4 clone; Santa Cruz Biotechnology) monoclonal antibodies using Pierce Direct IP Kit (Thermo Scientific) according to the supplier's instructions. Protein samples were separated in reducing SDS/PAGE gels, transferred to 0.45‐mm nitrocellulose membrane (GE Healthcare Life Sciences, Uppsala, Sweden) and blotted for the CD44 and ITGB1, respectively, as well as for STn with TKH2 monoclonal antibody. Protein extracts treated with α‐neuraminidase (Sigma Aldrich) were used as controls.

### 2.9 Statistical methods

Statistical data analysis was performed with IBM Statistical Package for Social Sciences – spss for Windows (version 20.0; IBM, New York, NY, USA). Chi‐square analysis was used to compare categorical variables. Kaplan–Meier survival curves were used to evaluate correlation between MUC16‐positive tumours and cancer‐specific survival (CSS) and were compared using log‐rank statistical test. CSS was defined as the period between the tumour removal surgery and patient death from cancer and the last follow‐up information.

## 3 Results and discussion

### 3.1 Expression of short‐chain *O*‐glycans in bladder cancer

Despite the biological and clinical relevance of altered *O*‐glycosylation in cancer, few studies have comprehensively addressed this matter in the context of bladder malignancies. Herein, 47 bladder cancer sections were screened by immunohistochemistry for short‐chain *O*‐glycans, using specific monoclonal antibodies. These included the Tn, STn and T antigens, as well as sialylated T glycoforms (mono‐ and disialylated forms) exposed after digestion of the histological sections with a neuraminidase. Particular emphasis was given to the expression of T antigen monosialylated forms S3T and also S6T, which is regarded as rare *O*‐glycan, until now mostly observed *in vitro* (Pinho *et al*., 2007) and more recently in superficial bladder tumours (Lima *et al*., 2013). Table 1 summarizes the expression of these glycans in the studied samples according to their disease subtype.

**Table 1:** Expression of short‐chain *O*‐GalNAC glycans in bladder tumours of different clinicopathological natures determined by immunohistochemistry

		Tn	STn	T	ST	S6T	S3T
Non‐muscle invasive bladder cancer (NMIBC) (%)							
Low Grade	17						
−		15 (88)	13 (76)	13 (76)	0 (0)	2 (12)	10 (59)
+		2 (12)	4 (24)	4 (24)	5 (29)	11 (65)	6 (35)
++					10 (59)	4 (24)	1 (6)
+++					2 (12)		
++++							
Positive cases, *n* (%)		2 (12)	4 (24)	4 (24)	17 (100)	15 (89)	7 (41)
High Grade	12						
−		5 (42)	3 (25)	9 (75)	0 (0)	0 (0)	3 (29)
+		7 (58)	7 (58)	3 (25)	3 (25)	5 (42)	7 (57)
++			2 (17)		2 (17)	7 (58)	2 (14)
+++					7 (58)		
++++							
Positive cases, *n* (%)		7 (58)	9 (75)	3 (25)	12 (100)	12 (100)	9 (71)
Muscle invasive bladder cancer (MIBC)							
	18						
−		16 (89)	2 (11)	0 (0)	0 (0)	5 (28)	7 (39)
+		2 (11)	16 (89)	4 (22)	3 (17)	8 (44)	7 (39)
++				6 (33)	5 (28)	6 (33)	4 (22)
+++				5 (28)	6 (33)		
++++				3 (16)	4 (22)		
Positive cases, *n* (%)		2 (11)	16 (89)	18 (100)	18 (100)	14 (78)	11 (61)
Total positive cases	47	11 (23)	13 (62)	25 (53)	47 (100)	38 (81)	27 (57)

Scoring: −, negative; +: > 0–19%; ++: 20–49%; +++: 50–79%; ++++: ≥ 80%.

#### 3.1.1 Expression of nonsialylated short‐chain *O*‐glycans (Tn and T antigens)

Table 1 highlights that Tn and T antigens are poorly expressed in bladder tumours (20–50% of total cases) in comparison with their sialylated counterparts (62% and 100%, respectively). More importantly, these antigens are mostly found in high‐grade tumours, irrespective of the degree of invasion. Nevertheless, the number of T antigen‐positive cases largely exceeds the Tn‐positive cases (53% *vs* 23%), which was particularly notorious in advanced tumours when compared to low‐grade superficial lesions. These observations suggest a possible overexpression of C1GalT1 (core 1 synthase, T‐synthase) or downregulation of other glycosyltransferases involved in *O*‐glycan extension in bladder tumours, which warrants careful evaluation in future studies. Possible modulation by secreted galactosidases, sialidases are also a possibility that should be investigated. Noteworthily, we have previously observed that bladder cancer cells exposed to hypoxia, a common microenvironmental feature in advanced tumours, promoted a striking downregulation in *C2GnT* accompanied by an increase in *C1GalT1* (Peixoto *et al*., 2016). It is possible that similar events may account for T antigen accumulation in bladder tumours. More importantly, neither Tn nor T antigens were found in the six studied healthy urothelia cases, demonstrating the malignant nature of these molecular alterations. Finally, our observations reinforce early studies in bladder cancer glycosylation describing an association between T antigen expression and tumour invasion (Langkilde *et al*., 1992). Focus should now be set on understanding the biological and clinical implications of this profound alteration in *O*‐glycosylation.

#### 3.1.2 Expression of sialylated short‐chain *O*‐glycans in bladder (STn and mono‐ plus disialyl‐T)

**Figure 1:**
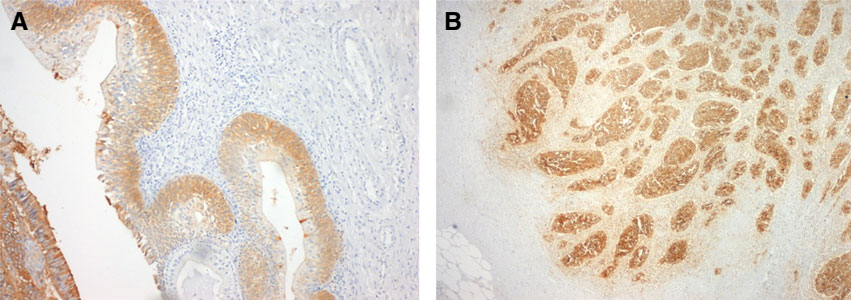
Immunohistochemistry for sialyl‐Tn (STn) antigen evidencing (A) expression in cells longing and invading the basal layer in high‐grade NMIBC and (B) extensive staining including in cells invading the muscle layer in MIBC.

**Figure 2:**
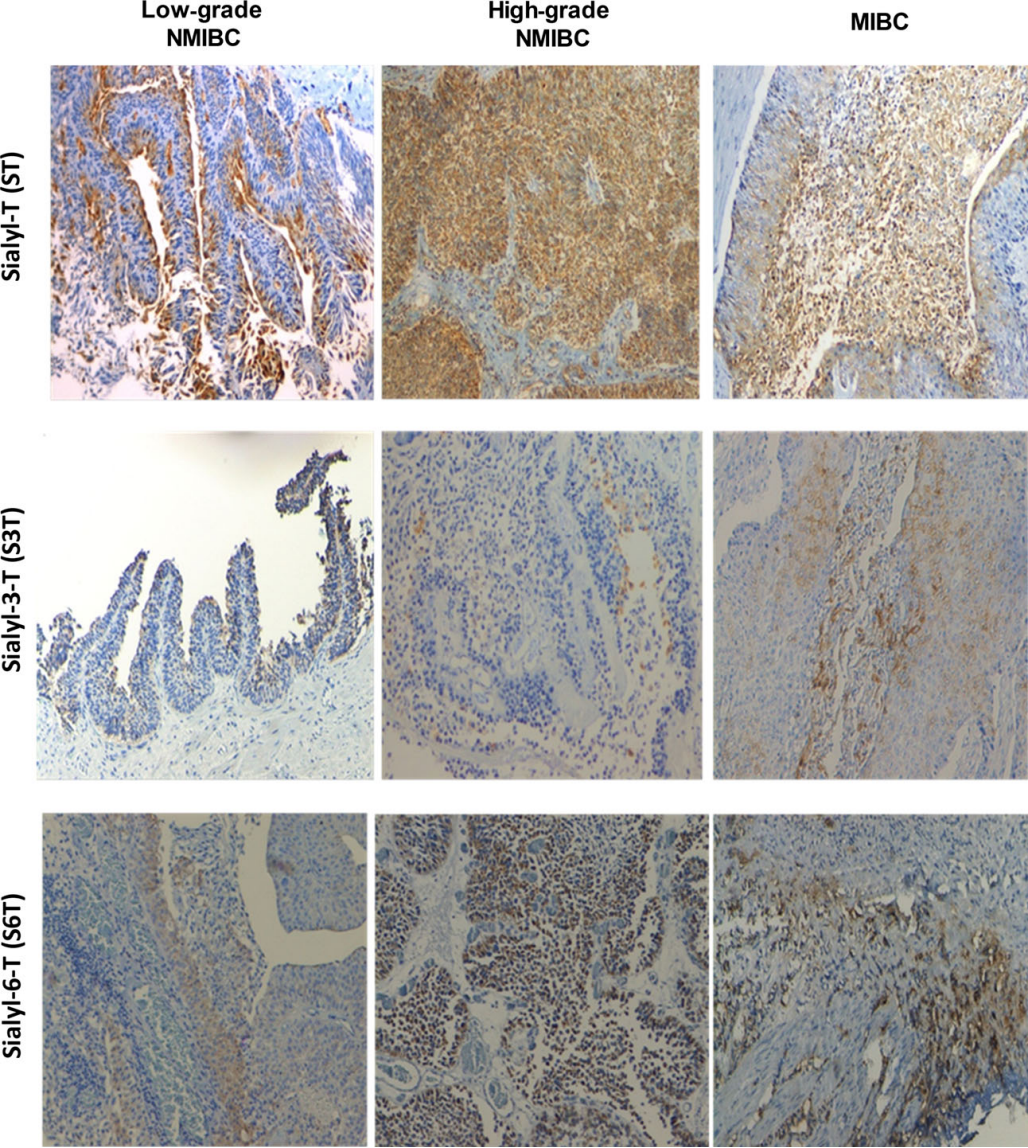
Immunohistochemistry for sialylated T antigens (ST: corresponding to mono‐ and disialylated T glycoforms; S3T and S6T) for low‐ and high‐grade superficial papillary muscle‐invasive bladder tumours. The figure highlights the increase in T sialylation with the severity of the lesions. As the S6T antigen was determined based on comparisons with STn expression after β‐(1,3)‐galactosidase digestion, only STn‐negative tumour lesions are being presented in this figure. Moreover, because the S3T antigen expression was determined based on comparisons with T antigen expression after α‐(2,3)‐neuraminidase treatment, only T‐negative tissues are being presented.

Contrasting with neutral short‐chain *O*‐glycans, sialylated Tn and T antigens, including mono‐ and/or disialyl‐T, are widely detected in bladder tumours irrespective of their grade and degree of invasion (62–100%; Table 1). In agreement with previous studies (Carrascal *et al*., 2014; Costa *et al*., 2015), the STn antigen was found in high‐grade and invasive tumours (75 and 89%, respectively), whereas only 24% of low‐grade cases were positive. The majority of the positive cases presented a low extension of expression (< 20%), of focal and polydisperse nature, throughout the tumour. STn was mostly found in cells of the basal layer (Fig. 1A); yet in tumour areas presenting extensive staining (> 50%) (Fig. 1B), it could also be detected in papillary urothelium and invasive fronts (Fig. 1B). Moreover, whenever present in the tumour, STn was also detected in the adjacent but not in the distal mucosa, also in agreement with previous reports (Carrascal *et al*., 2014; Costa *et al*., 2015). Hence, cells neighbouring the tumour are thought to carry significant alterations that result in the expression of this antigen. We also note that increase in STn is generally accompanied by a loss of Tn, reinforcing the association between increase in sialylation of *O*‐glycan precursors and the severity of the lesions (Table 1). On the other hand, the sialylated forms of the T antigen, including mono‐ and/or disialylated glycans, are diffusely expressed by all studied bladder tumours (Table 1 and Fig. 2). However, a significant increase in the extension of sialylated T antigen could be observed in more advanced cases, suggesting an overexpression and/or increased activity of sialyltransferases (Fig. 2). In agreement with these observations, it has been demonstrated that advanced‐stage bladder tumours overexpress ST3Gal‐I (Videira *et al*., 2009), the glycosyltransferase responsible for T antigen sialylation. In summary, while superficial tumours mostly present sialylated T antigens, more advanced‐stage tumours also co‐express more immature *O*‐glycans, including the STn antigen (Table 1) that has been frequently associated with more malignant phenotypes and poor outcome (Bernardo *et al*., 2014; Cabral *et al*., 2010; Costa *et al*., 2015; Ferreira *et al*., 2013).

#### 3.1.3 Exploring the nature of T antigen sialylation

Despite the widespread nature of sialylated T antigens in healthy and, particularly, malignant tissues, few studies have focused on disclosing the nature of T antigen sialylation, most likely due to the lack of specific monoclonal antibodies and limitations in glycomics approaches. Facing these problems, we digested bladder tumour sections with a β‐(1‐3)‐galactosidase prior to incubation with the anti‐STn monoclonal antibody, to address the possibility of *O*‐6 GalNAc sialylation (S6T). This procedure was responsible for the removal of *Ο*‐3‐linked Gal residues from S6T antigens exposing STn antigens for recognition (Fig. S2A). Accordingly, we observed positive staining after enzymatic treatment in STn‐negative tumours (Fig. S2A), as well as an increased STn expression in several cases (Table 1), suggesting the presence of the S6T antigen. This glycan was found in approximately 80% of the studied tumours, with similar percentage of positive cases between NMIBC and MIBC. However, increased extension of expression could be observed in advanced tumour (Table 1 and Fig. 2). The S6T was further evaluated in FFPE healthy urothelium from six necropsied male individuals, which confirmed its cancer‐associated nature. Recently, we have described that the presence of S6T and STn in bladder tumours was associated with a better response to BCG immunotherapy for more aggressive NMIBC, suggesting that *O*‐6 sialylation plays a key role in *bacillus* binding to the epithelium (Lima *et al*., 2013). Such observations reinforce the importance of including alterations in glycosylation in panomics predictive molecular models. Moreover, we have described an overexpression of ST6GalNAc‐I, a key glycosyltransferase involved in *O*‐6 sialylation of Tn antigens (Sewell *et al*., 2006) in advanced‐stage bladder tumours (Ferreira *et al*., 2013). Future studies should be conducted to disclose the transcription of ST6GalNAc‐I/II and possibly ST6GalNAc‐IV, known to be involved in the *O*‐6 sialylation of Tn antigens (Spiro, 2002), gaining more insights on the biological mechanisms underlying these molecular alterations and its clinical relevance.

On the other hand, incubation with a α‐neuraminidase specific for cleaving *O*‐3‐linked sialic acids allowed T antigen detection in some negative tissues (Fig. S2B) and increased the extension and intensity of expression in T antigen‐positive cases (Table 1), strongly suggesting the presence of the S3T antigen. Contrasting with the ubiquitous nature of S6T, the S3T antigen was mostly found in high‐grade NMIBC (41% low‐grade NMIBC; 71% high‐grade NMIBC; 61% MIBC). Nevertheless, we should note that many high‐grade tumours co‐express both T sialylated forms. These observations support previous associations between the overexpression of both sialyl‐T and ST3Gal‐I, the sialyltransferase responsible for T antigen *O*‐3 sialylation, in high‐grade tumours (Videira *et al*., 2009). Moreover, similar to S6T, the S3T antigen was also not detected in the healthy urothelium, reinforcing the cancer‐associated nature of these antigens.

In summary, we have demonstrated that there are minor subsets of advanced‐stage tumours that co‐overexpress nonsialylated short‐chain *O*‐glycans (Tn and T antigens) in association with their sialylated glycoforms. Moreover, we have highlighted the structural diversity of T antigen sialylation in bladder tumours, its cancer‐associated nature and the prevalence of up until now neglected *O*‐6 sialoforms. Interestingly, this mimics the sialylation of the Tn antigen, whose biological and clinical significance has been extensively studied by our group. Furthermore, we have again reinforced the association between STn antigen expression and aggressive disease, raising to over 300 the number of evaluated tumour sections of different clinicopathological classifications and aetiologies (Bernardo *et al*., 2014; Cabral *et al*., 2010; Costa *et al*., 2015; Ferreira *et al*., 2013; Lima *et al*., 2013; Peixoto *et al*., 2016; Santos *et al*., 2014). Significant efforts should be put on providing accurate quantification of these antigens using high‐throughput glycomics approaches and on developing highly specific ligands. This would set the necessary means for large‐scale clinical studies and targeted therapeutics. Moreover, it will be crucial for understanding the molecular mechanisms underlying glycomic alterations, including (a) to determine the events modulating the expression and activity of glycosyltransferases and glycosidases in bladder tumours; (b) to access the distribution of glycosyltransferases throughout the secretory organelles and pathways; (c) to explore mutations in key enzymes involved in *O*‐glycans biosynthesis and its functional impact. Such information will be crucial for accessing the biological and clinical significance of altered *O*‐glycosylation in bladder cancer, providing relevant insights for glycoproteomics studies and ultimately the design of novel and more effective therapeutics (Ferreira *et al*., 2016a).

### 3.2 Bladder cancer‐targeted glycoproteomics

**Figure 3:**
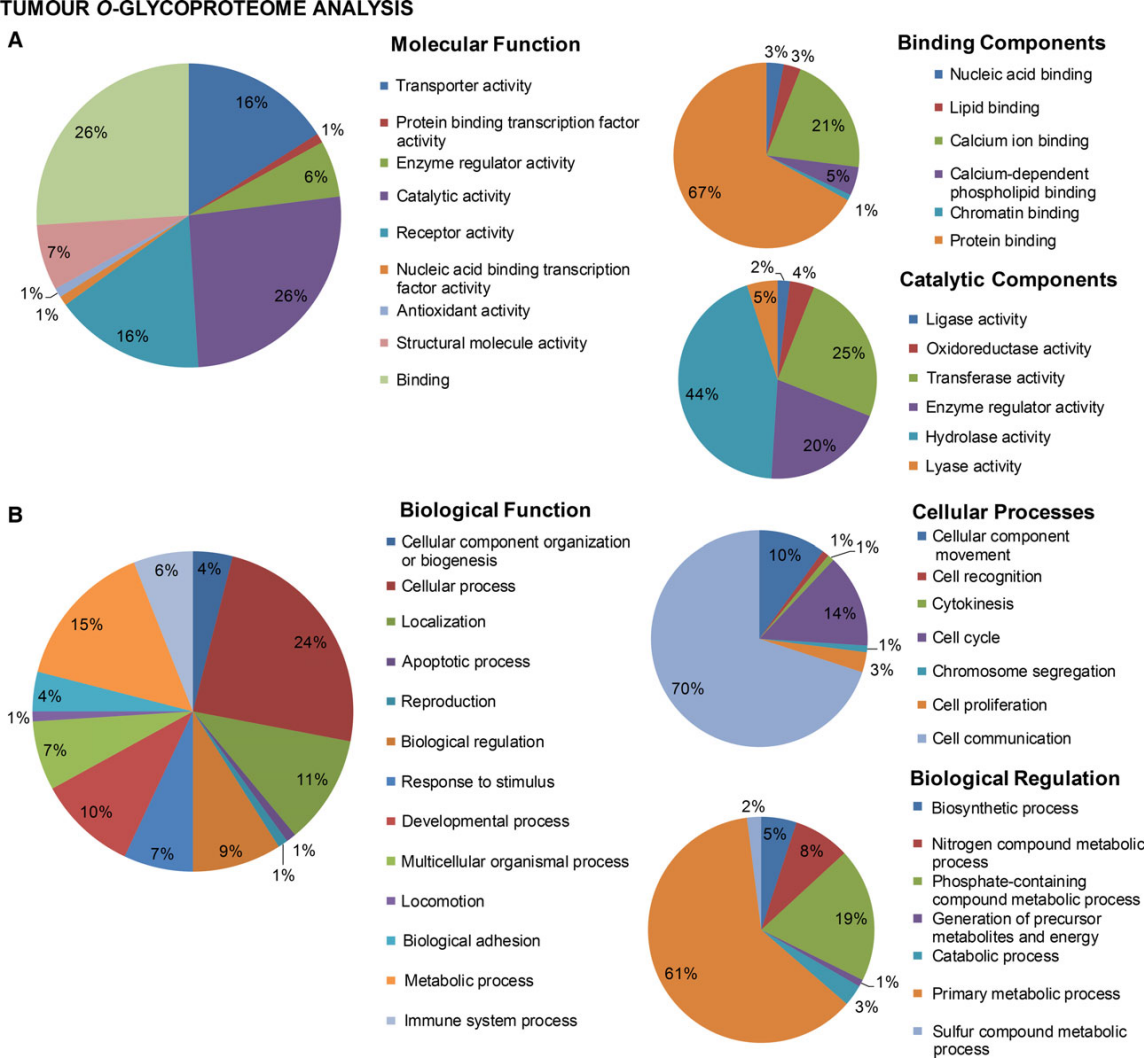
Distribution of candidate STn‐expressing glycoproteins in muscle‐invasive bladder tumours (detailed in Table S2) comprehensively integrated according to cellular localization (A), molecular (B) and cell functions (C) based on gene ontology analysis by Panther bioinformatics tool. STn‐expressing proteins were found to be associated with a wide array of molecular and biological functions as depicted in detail in the figure. Accordingly, the identified glycoproteins were involved in nine main classes of molecular functions, with an overrepresentation of catalytic activities (hydrolase, lyase and transferase activities) and protein binding mediation. Moreover, 13 main biological functions were highlighted, being the most representative cellular processes such as cell communication and, to some extent, cell cycle control. These observations suggest that altered glycosylation may influence a wide range of key cell events, which warrants evaluation in future studies.

**Figure 4:**
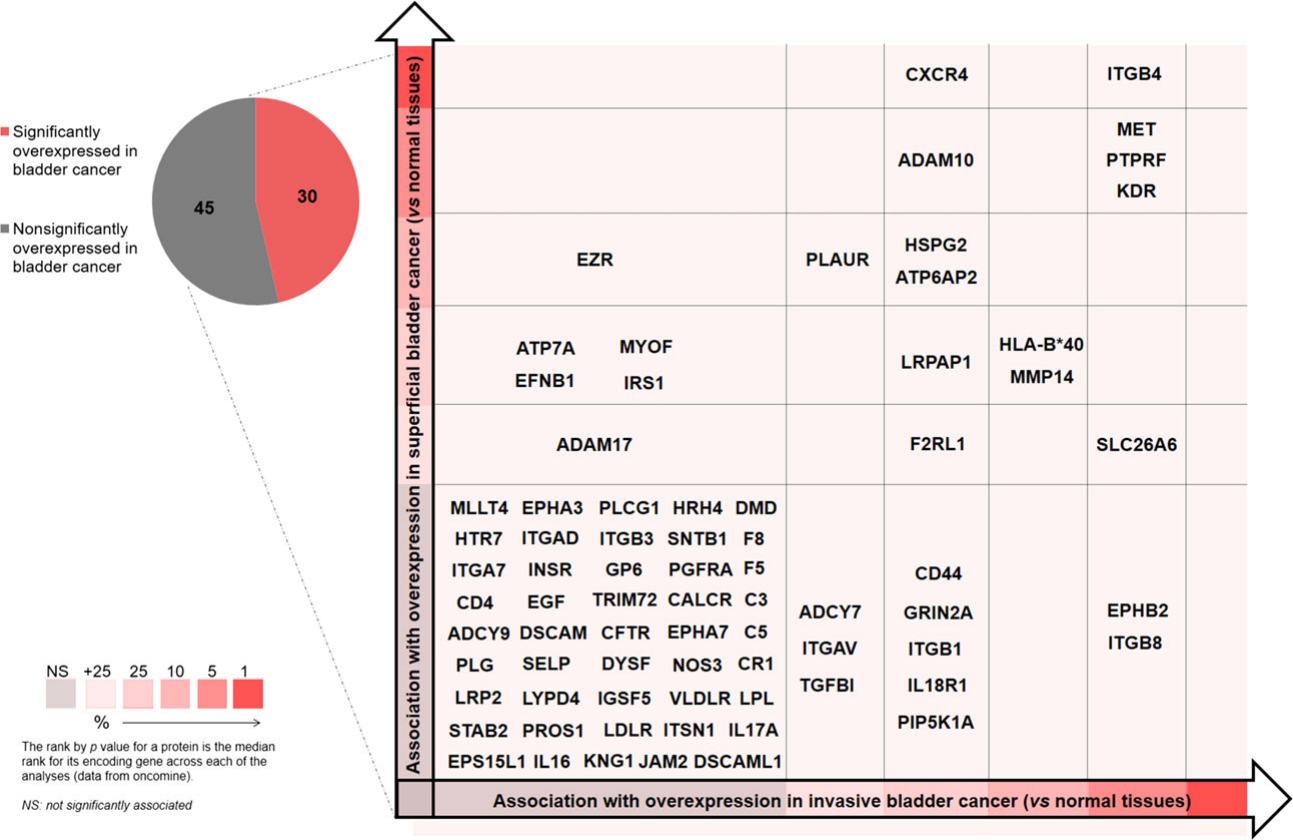
Candidate STn‐expressing glycoproteins in muscle‐invasive bladder tumours comprehensively distributed according to its association with the severity of the lesions. Briefly, the identified glycoproteins were distributed according to associations with the type of lesion based on an *in silico* analysis with Oncomine. Proteins identified for the first time in bladder tumours have not been included in the graph due to the lack of associations with the type of disease.

**Figure 5:**
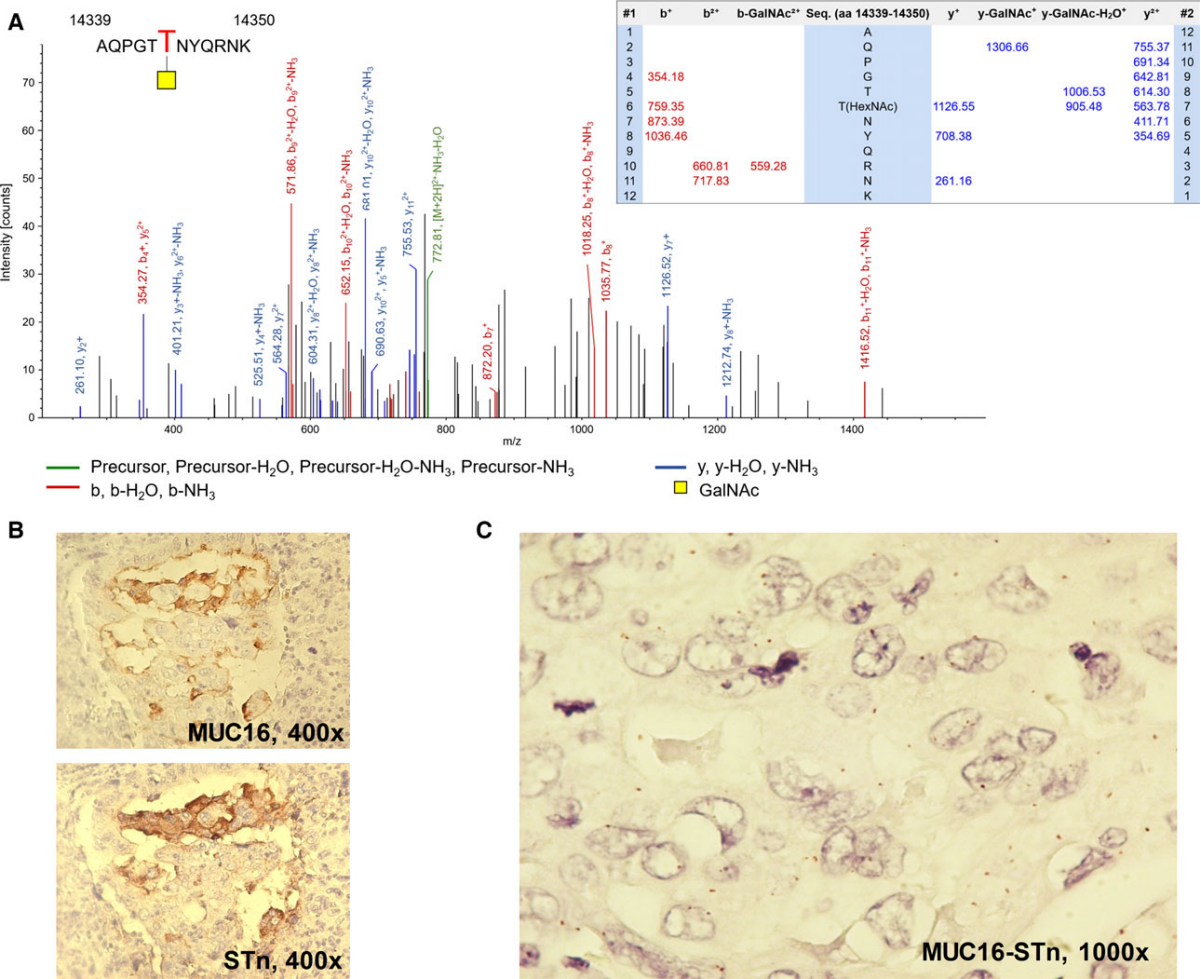
(A) Exemplificative annotated nanoLC‐ESI‐LTQ‐Orbitrap‐CID‐MS/MS spectra for a MUC16 glycopeptide substituted with a HexNAc residue evidencing the specific glycosite; (B) colocalization of MUC16 and STn in bladder tumours by immunohistochemistry; (C) expression of MUC16 STn glycoforms in bladder tumours based on PLA analysis. This work identified for the first time MUC16 in bladder tumours and its association with abnormal glycoforms such as the STn antigen. The mass spectrum shows a MUC16 glycopeptide substituted with a HexNAc residue, strongly suggesting the presence of STn. The colocalization of MUC16 and STn (B) in bladder tumours also reinforces this hypothesis. Finally, the red dots on the PLA image (C) in areas of colocalization result from the simultaneous detection of both antigens, reinforcing this evidence.

Based on our previous and current observations, the STn constitutes a key cancer‐associated antigen highly associated with advanced disease and poor prognosis (Bernardo *et al*., 2014; Costa *et al*., 2015; Ferreira *et al*., 2013; Lima *et al*., 2013; Peixoto *et al*., 2016). Moreover, we have observed that STn expression significantly favours cell motility and capacity to invade (Ferreira *et al*., 2013; Peixoto *et al*., 2016) as well as immune escape (Carrascal *et al*., 2014). Therefore, mapping the STn‐glycoproteome is crucial for developing highly specific targeted therapeutics against advanced‐stage bladder tumours. However, while the majority of glycoproteomics studies presented so far have focused mostly on body fluids and, to lower extent, human tissues, none has attempted to address protein glycosylation in FFPE tissues. Herein, we extracted proteins from five MIBC tumours and screened the samples for STn expression by western blot, which retrieved similar expressions patterns (Fig. S5A). These samples were then pooled and analysed by a conventional gel‐based and nanoLC‐MS/MS proteomics approach (Fig. S3A), which allowed the identification of 2578 peptides, corresponding to 294 proteins (Table S1). This illustrated that the feasibility of using FFPE has starting material for retrospective proteomic studies on clinical samples, despite the significant modifications and degradation induced by paraffin embedding. Gene ontology interpretation of the results using Panther highlighted the presence of proteins from all cell compartments, including plasma membrane proteins known to yield the STn antigen (4%; Fig. S4A); nevertheless, an overrepresentation of cytoplasmic and cytoskeleton proteins could be observed (Fig. S4A), in accordance with its higher abundance in the cellular milieu. The main represented molecular functions included binding, structural and catalytic activities, whereas the main biological functions were set on metabolic and cellular processes (Fig. S4B,C), in accordance with the wide range of identified proteins. Nevertheless, due to the low abundance of STn‐expressing membrane glycoproteins potentially yielding the STn antigen, an enrichment step was introduced based on affinity to VVA lectin that selectively binds terminal GalNAc residues. To render the glycoproteins with affinity for the chosen lectin, the extracts were digested with a α‐neuraminidase prior to the enrichment step, which removed sialic acids from STn exposing the GalNAc residue (Tn antigen). The absence of Tn and blood group A determinants in the chosen cases ensured the specificity of the enrichment for STn‐expressing proteins (Fig. S3B). Subsequent nanoLC‐MS/MS analysis led to the identification of over 400 *O*‐glycosites and 143 membrane glycoproteins putatively expressing the STn antigen (Table S2), which may be potential targets for targeted therapies. These glycoproteins were found associated with a wide array of molecular and biological functions, as depicted in detail in Fig. 3. In particular, STn‐expressing proteins mostly mediate binding to other proteins and have hydrolase catalytic activities. They also mediate cell–cell communication and signalling and regulate primary metabolic processes. These observations strongly suggest that altered glycosylation may influence a wide array of cell functions, thereby providing key preliminary insights to understand the role of STn expression in bladder cancer. Approximately half of the identified glycoproteins had been previously studied in the context of bladder cancer and could be comprehensively distributed according to its association with disease on an analysis *in silico* with Oncomine (Rhodes *et al*., 2007) (Fig. 4). This list included CD44, a typical bladder cancer stem‐cell associated glycoprotein also associated with drug‐resistant phenotypes and poor prognosis (Kobayashi *et al*., 2016), and several integrins, in accordance with previous observations (Peixoto *et al*., 2016). For validation purposes, we have immunoprecipitated CD44 and ITGB1 in these samples and confirmed the expression of STn by western blot (Fig. S5B). Furthermore, immunohistochemistry showed the co‐expression of these antigens in the same tumour area, which was confirmed by PL (Fig. S5C), which allows the simultaneous detection of the protein and the glycan whenever there is close proximity. In addition to these glycoproteins, we have also identified, for the first time, MUC16 and abnormal MUC16 glycoforms in bladder tumours (Table S1). Interestingly, these high molecular weight glycoproteins are generally found in ovarian tumours facing poor prognosis, being frequently used for serological monitoring and as diagnostic marker of ovarian cancer (CA125 test) (Duffy *et al*., 2005; Felder *et al*., 2014; Vasudev *et al*., 2011). Again, we have confirmed the presence of STn in MUC16‐derived glycopeptides based on characteristics of CID‐MS/MS fragmentation spectra (Fig. 5A). Moreover, we found glycopeptides carrying both GalNAc and Gal‐GalNAc substituents, highlighting the complex antigenic glycoarray presented by bladder cancer‐associated glycoproteins (Fig. S6). In addition, the analysis of consecutive bladder tumour sections revealed that MUC16 expression is associated and colocalized with STn expression in 95% of the cases, irrespective of their histological classification (Fig. 5B). Moreover, PLA confirmed the presence of MUC16 STn^+^ glycoforms in clinical samples (Fig. 5C). Despite unequivocal data supporting the existence of MUC16 STn‐ glycoforms, we have further attempted to IP and blot this glycoprotein as it has been done for CD44 and ITGB1; however, its high molecular weight (Mw < 2000 kDa) and the lack of suitable antibodies for this procedure have posed as a significant analytical limitation that will be addressed in future studies. Even though this is the first report regarding MUC16 expression in bladder cancer, CA125 elevation has been previously observed in the serum of patients with advanced pathological stage in comparison with lower‐stage disease, suggesting that this antigen may predict advanced bladder cancer (Margel *et al*., 2007; Vasudev *et al*., 2011). Furthermore, abnormal CA125 levels have been associated with unresectable tumours, again reinforcing its association with worse prognosis (Vasudev *et al*., 2011).

### 3.3 Clinical significance of MUC16 expression in bladder cancer

**Figure 6:**
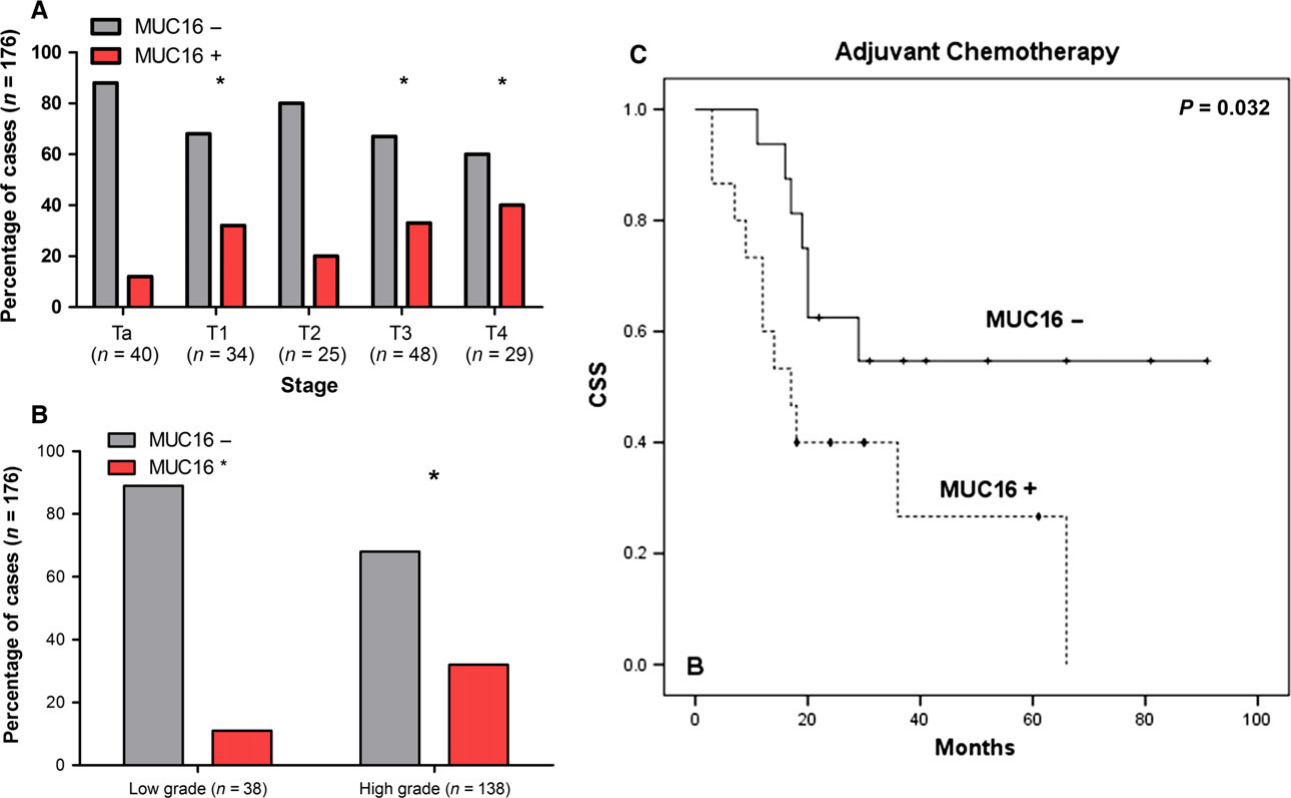
(A) Associations of MUC16 with the stage; (B) grade of the disease; and (C) decreased overall survival in patients with MIBC subjected to cisplatin‐based chemotherapy. Accordingly, MUC16 was associated with more aggressive bladder tumours, namely advanced stages and grade of the disease. Moreover, its presence in MIBC associates with decreased survival in MIBC subjected to chemotherapy. For (A) and (B), comparisons between data sets were made by chi‐square test (*P < 0.05); in (C), a log‐rank test was performed, *P* = 0.032. + censored MUC16‐negative tumours; ♦ censored MUC16‐positive tumours.

Given the key role of MUC16 in ovarian cancer (Felder *et al*., 2014; Ricardo *et al*., 2015), and building on the lack of clinical data for bladder cancer, we have screened a retrospective series of 176 tumours spanning different classifications (74 NMIBC and 102 MIBC). MUC16 was mainly expressed in the cell membrane and cytoplasm, with moderated and focal expression that did not exceed 20% of tumour cells for the majority of the positive cases (Fig. 5B), irrespective of their histological/TNM classification. The MUC16 antigen was observed in approximately 27% of cases (48 of 176), mainly in tumours showing *lamina propria* (T1; 30%) and *muscularis propria* (≥ T2; 20–40%) invasion; conversely, the number of MUC16‐positive Ta tumours was lower than 15% (Fig. 6A; *P* < 0.005). Concerning WHO criteria, MUC16‐positive cells were mostly observed in the high‐grade cases (*P* = 0.008; Fig. 6B), reinforcing the association between MUC16 expression and poor prognosis. In agreement with these observations, we have also observed an increased transcription of *MUC16* gene in MUC16‐positive tumours in comparison with MUC16‐negative tumours (Fig. S7, *P* = 0.005). Moreover, we found that MUC16 expression associates with lower CSS in MIBC patients treated with cisplatin and gemcitabine, suggesting a possible role in drug resistance that is being currently evaluated. These observations are in agreement with the findings from serological CA125 evaluation (Felder *et al*., 2014; Rao *et al*., 2015) and strongly support the need for a deeper investigation on the biological and clinical significance of MUC16 in bladder cancer.

## 4 Conclusions

It has been long known that advanced bladder tumours present significant alterations in glycosylation that relate to worst prognosis; however, there is a lack of information on the structural nature of cancer‐specific glycans. This work highlights that advanced bladder tumours overexpress and frequently co‐express an array of short‐chain *O*‐glycans resulting from a premature stop in the glycosylation of membrane and secreted proteins. Moreover, it clearly demonstrates a predominance of sialylated over neutral glycoforms, with emphasis on sialylated Tn and T antigens. In addition, for the first time, we provide key insights on the nature of the T antigen sialylation, which will be crucial for guiding future glycomics and glycoproteomics studies and for designing specific ligands against bladder cancer cells. Moreover, we have highlighted a significant increase in *O*‐6 sialylation in bladder tumours, particularly the STn antigen. Finally, we have mined the glycoproteome of advanced bladder tumours for STn‐expressing glycoproteins. This resulted in the identification of MUC16 as a novel biomarker for a subset of bladder tumours presenting poor prognosis. It also highlighted a molecular link between bladder and ovarian cancer, where abnormally glycosylated MUC16 plays a key role in disease progression and dissemination. Future studies should now be focusing on the biological role of this glycoprotein in bladder cancer. Our findings also reinforce the need to comprehensively address the CA125 antigen in the sera and, possibly, also urine of patients with bladder cancer. Furthermore, we augment that a careful mapping of MUC16 and other cancer‐associated glycoproteins may provide the necessary structural information for highly specific biomarkers and targeted therapeutics.

## Author contributions

LLS and JAF conceived and designed the project; RC and LLS provided the samples; SC, RA, CG, DF, AP, EF, MN, DN, AT, MR and AMNS acquired the data; SC, RA, DF, TA, LL, RC, AMNS, LLS and JAF analysed and interpreted the data; RA and JAF wrote the manuscript.

## Supporting information


**Fig. S1.** Schematic representation protein *O*‐GalNAc glycosylation biosynthesis evidencing the cancer‐associated short‐chain glycans explored in this study.
**Fig. S2.** Schematic representation of the analytical strategy for S6T and S3T evaluation by immunohistochemistry.
**Fig. S3.** Analytical workflow for (A) whole proteome analysis starting from FFPE tissues and (B) identification of STn expressing glycoproteins in bladder tumours.
**Fig. S4.** Proteins isolated from FFPE muscle‐invasive bladder tumours distributed according to cellular localization (A), molecular (B) and cell functions (C) based on gene ontology analysis.
**Fig. S5.** (A) Western blot for glycoproteins expressing the STn antigen in advanced bladder tumours. (B) Identification of STn glycoforms in CD44 and ITGB1 glycoproteins isolated from advanced bladder tumours by immunoprecipitation. (C) Immunohistochemistry and PLA for CD44, ITGB1 and STn in bladder tumours.
**Fig. S6.** Annotated nanoLC‐ESI‐LTQ‐orbitrap‐CID‐MS/MS spectra for a MUC16 glycopeptide substituted with a HexNAc and HexNAc‐Hex residues evidencing the specific glycosites (highlighted in the assignment table below).
**Fig. S7.** Association between MUC16 classification by immunohistochemistry in FFPE cancer tissues (IHC; negative vs positive) and *MUC16* expression.
**Table S1.** Proteins identified with high confidence level in Tn‐negative, blood group A negative, STn‐positive tumour samples recovered from formalin‐fixed paraffin embedded tissues.
**Table S2.** Identified membrane glycoproteins from Tn‐negative, blood group A negative, STn‐positive MIBC, with O‐HexNAc as posttranslational modifications after neuraminiase treatment.
