# Supplementary material for: Targeted O‐glycoproteomics explored increased sialylation and identified MUC16 as a poor prognosis biomarker in advanced‐stage bladder tumours
Source: Mol Oncol. 2017 Mar 2;11(8):895–912. doi: 10.1002/1878-0261.12035 (PMC5537688; doi:10.1002/1878-0261.12035)
Supplement: Supplementary file 1 — Fig. S1. Schematic representation protein O‐GalNAc glycosylation biosynthesis evidencing the cancer‐associated short‐chain glycans explored in this study. Fig. S2. Schematic representation of the analytical strategy for S6T and S3T evaluation by immunohistochemistry. Fig. S3. Analytical workflow for (A) whole proteome analysis starting from FFPE tissues and (B) identification of STn expressing glycoproteins in bladder tumours. Fig. S4. Proteins isolated from FFPE muscle‐invasive bladder tumours distributed according to cellular localization (A), molecular (B) and cell functions (C) based on gene ontology analysis. Fig. S5. (A) Western blot for glycoproteins expressing the STn antigen in advanced bladder tumours. (B) Identification of STn glycoforms in CD44 and ITGB1 glycoproteins isolated from advanced bladder tumours by immunoprecipitation. (C) Immunohistochemistry and PLA for CD44, ITGB1 and STn in bladder tumours. Fig. S6. Annotated nanoLC‐ESI‐LTQ‐orbitrap‐CID‐MS/MS spectra for a MUC16 glycopeptide substituted with a HexNAc and HexNAc‐Hex residues evidencing the specific glycosites (highlighted in the assignment table below). Fig. S7. Association between MUC16 classification by immunohistochemistry in FFPE cancer tissues (IHC; negative vs positive) and MUC16 expression. Table S1. Proteins identified with high confidence level in Tn‐negative, blood group A negative, STn‐positive tumour samples recovered from formalin‐fixed paraffin embedded tissues. Table S2. Identified membrane glycoproteins from Tn‐negative, blood group A negative, STn‐positive MIBC, with O‐HexNAc as posttranslational modifications after neuraminiase treatment. [file MOL2-11-895-s001.docx]

**Exploring increased sialylation in advanced stage bladder tumours by targeted O-glycoproteomics identifies MUC16 as a biomarker of poor prognosis**

Sofia Cotton^1*^, Rita Azevedo^1,2*^, Cristiana Gaiteiro^1^, Dylan, Ferreira^1^, Luís Lima^1,3^, Andreia Peixoto^1,2,3^, Elisabete Fernandes^1,2,3^, Manuel Neves^1^, Diogo Neves^1^, Teresina Amaro^4^, Ricardo Cruz^5^, Ana Tavares^1,6^, Maria Rangel M, André M. N. Silva^8^, Lúcio Lara Santos^1,9,10^ and José Alexandre Ferreira^1,2,3,11^

^1^Experimental Pathology and Therapeutics Group, Portuguese Institute of Oncology, Porto, Portugal; ^2^Institute of Biomedical Sciences Abel Salazar, University of Porto, Porto, Portugal; ^3^Instituto de Investigação e Inovação em Saúde, Universidade do Porto, Portugal; ^4^Department of Pathology, Hospital Pedro Hispano, Matosinhos, Portugal; ^5^Department of Urology, Portuguese Institute of Oncology of Porto, Portugal; ^6^Department of Pathology, Portuguese Institute of Oncology of Porto, Portugal; ^7^UCIBIO-REQUIMTE, Instituto de Ciências Biomédicas Abel Salazar, University of Porto, Porto, Portugal; ^8^UCIBIO-REQUIMTE/Department of Chemistry and Biochemistry, Faculty of Sciences, University of Porto, Porto; ^9^Health School of University Fernando Pessoa, Porto, Portugal; ^10^Department of Surgical Oncology, Portuguese Institute of Oncology, Porto, Portugal; ^11^Porto Comprehensive Cancer Center (P.ccc), Porto, Portugal.

**Corresponding authors:** José Alexandre Ferreira (josef@ipatimup.pt)

*****Equal contribution

**Keywords:** bladder cancer; glycosylation; sialic acids; precision medicine; glycoproteomics; MUC16

**Running title:** Targeted glycoproteomics in bladder cancer


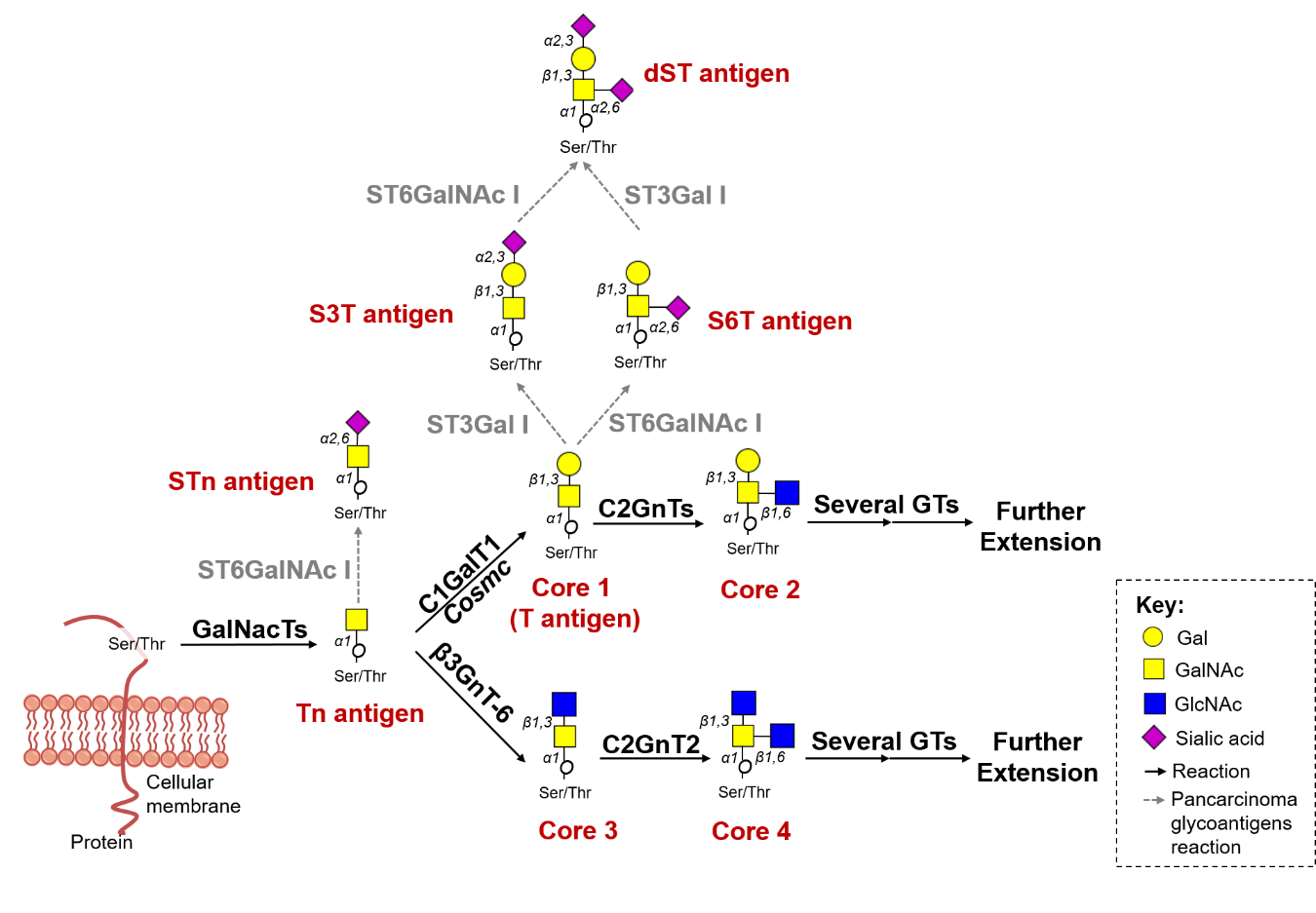


**Figure S1. Schematic representation protein *O*-GalNAc glycosylation biosynthesis evidencing the cancer-associated short-chain glycans explored in this study.** *O*-GalNAc glycosylation is a posttranslational modification commonly found in membrane glycoproteins extracellular domains and secreted glycoproteins. It can also be observed in secretory organelles inside the cell. This type of *O*-glycosylation plays a key role in the definition of protein conformation and key biological functions including: i) cell-cell and cell-extracellular matrix adhesion; ii) recognition by the immune system, pathogens and virus; iii) protection of glycoproteins against proteolysis; iv) regulation of key intracellular signalling pathways through the modulation of cell receptors activities, amongst other roles. Glycoprotein *O*-GalNac glycosylation initiates in the endoplasmatic reticulum and its further elongated into more structurally complex structures in the golgi. Briefly, *O*-glycosylation begins with the addition of GalNAc to a serine or threonine residue of a given protein backbone originating the simplest form of *O*-glycosylation, the Tn antigen. The reaction is catalyzed by polypeptide N-Acetylgalactosamine transferases (ppGalNAcTs), a superfamily of 20 enzymes with high substrate and tissue specificity, whose expression is regulated at cellular and tissue levels. The coordinated action of these glycosyltransferases determines the density and distribution of glycosylation sites on a given glycoprotein. Frequently, β-(1-3)-galactosyltransferase, C1Gal-T1 and its molecular chaperone COSMC, elongates the Tn antigen by adding a Gal residue to the *O*-3 GalNAc residue. This originates the core 1 structure, also designated T antigens, which functions as precursor of more elongated core structures by coordinated action of several glycosyltransferases, as depicted in more detail in this Figure. Of note, mature *O*-glycans may present ABO and Lewis blood group related antigens that decisively contribute to the definition of the antigenic profile of a given cell. However, in cancer cells, early sialylation of Tn and T antigens form the sialyl-Tn (STn), and sialyl-T (ST) antigens, whose biosynthesis is highlighted in detail in the Figure. Accordingly, ST6GalNAcs may promote the *O*-6 sialylation of the Tn antigen but also T, originating S6T. On the other hand, ST3Gal.I promote the *O*-3 sialylation of the T antigen originating S3T. The coordinated action of these enzymes give rise to the disialylated T glycoforms. These event stop further glycan elongation and may dramatically change the conformational and functional properties of a given glycoprotein, favouring cell migration, immune escape and activation of key oncogenic pathways.

**Figure S2. Schematic representation of the analytical strategy for S6T and S3T evaluation by immunohistochemistry.** A) The S6T antigen may be considered structurally related with STn, since it presents an *O*-6 sialylation of its GalNAc residue. As such we have used a β-(1,3)-galactosidase to remove the Gal residue exposing an STn antigen for recognition by TKH2 monoclonal antibody. This allowed the detection of STn in previously negative STn tumours sections, strongly suggesting the expression of S6T. In addition, there was a significant increase in staining in several STn positive tumours after enzymatic digestion (not shown). B) The S3T antigen presents an *O*-3 Gal sialylation and after α-(2,3)-neuraminidase digestion exposes the T antigen for recognition by the anti-T antigen monoclonal antibody 3C9. The presence of S3T was determined by comparing T antigen expression in tumours sections prior and after enzymatic digestion.


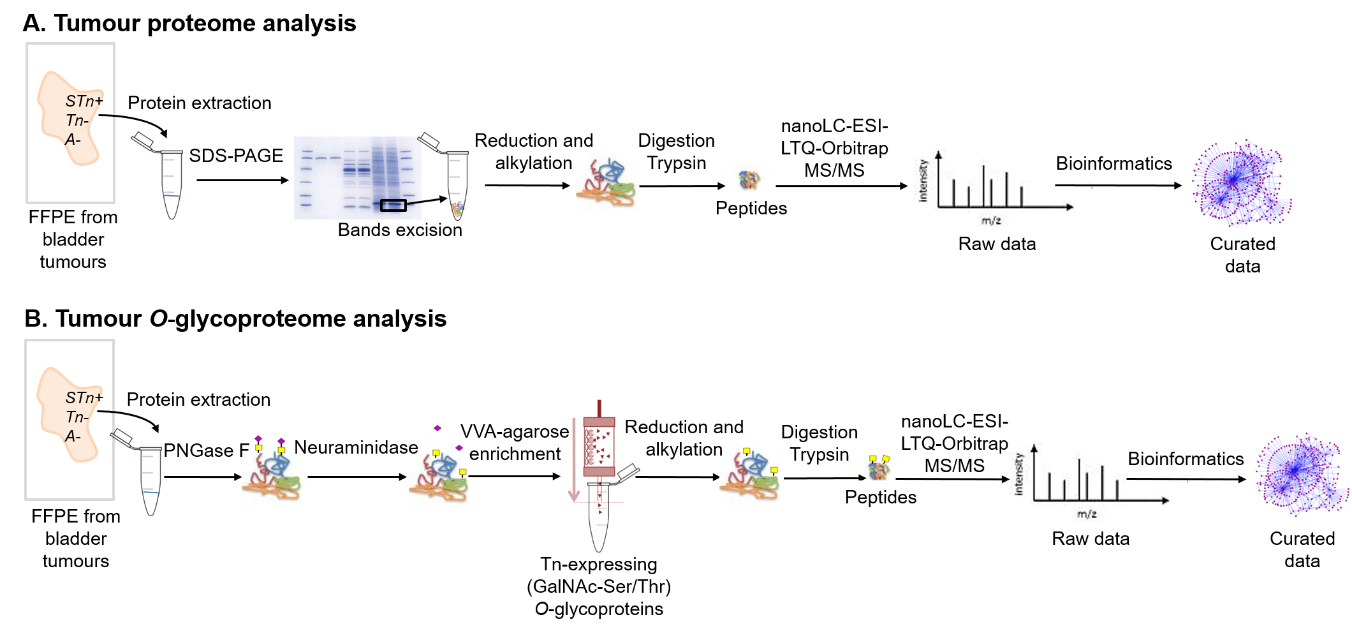


**Figure S3. Analytical workflow for A) whole proteome analysis starting from FFPE tissues and B) identification of STn expressing glycoproteins in bladder tumours.** A) **Analytical workflow to access the proteome.** Briefly, the proteins were extracted from FFPE muscle-invasive tumors isolated from male patients using the Qproteome FFPE tissue kit (Qiagen, Hilden, Germany) according to the vendor instructions. The proteins were pooled and then separated on 4-20% SDS-PAGE gels, excised, digested with trypsin and analyzed by nanoLC-ESI-LTQ-orbit-MS/MS. Data was comprehensively interpreted using Phanter. B) Analytical workflow used to access putative STn-expressing glycoproteins in a pool of proteins extracted from tumours of five male patients with muscle invasive bladder tumours. The glycoproteins were first N-deglycosylated with PnGAse F to facilitated downstream mass spectrometry analysis by improving proteolytic digestions and protein coverage. Glycoproteins modified with the STn antigen were then identified by nanoLC-ESI-LTQ-obritrap-CID-MS/MS after α-neuraminidase digestion followed by enrichment for GalNAc-expressing protein glycoforms. Enrichment was done based on affinity for the VVA lectin, which selectively bindings glycans with terminal GalNAc residues. To decrease the possibility of false positives only Tn and blood group A negative tumours the mentioned structural feature were excluded from analysis. After enrichment the glycoproteins were reduced, alkylated, digested trypsin to yield peptides for nanoLC-ESI-LTQ-orbitrap-CID-MS/MS (orbitrap MS) analysis. The final protein list included only membrane glycoproteins with putative *O*-glycosylation domains as determined by the NetOGlyc 4.0 software [42] and/or exhibiting a glycopeptide with at least one HexNAc substituent.

**Figure S4. Proteins isolated from FFPE muscle-invasive bladder tumours distributed according to cellular localization (A), molecular (B) and cell functions (C) based on gene ontology analysis.** Briefly, SDS-PAGE-nanoLC-ESI-MS/MS analysis of a pool of proteins isolated from FFPE muscle-invasive bladder tumour sections led to the identification of 294 proteins (detailed in Table S1). The integration of these results based on gene ontology analysis using Panther [43 highlighted the presence of proteins from all cell compartments, with an overrepresentation of cytoplasmatic and cytoskeleton proteins, in accordance with their higher concentration in the cellular millieu; nevertheless, low percentages of plasma membrane proteins (4%) and extracellular matrix (3%) that generally present *O*-GalNAc type glycosylation are also detected. The main represented molecular functions included binding, structural and catalytic activities, whereas main molecular functions were set on metabolic and cellular processes. In summary, a wide array of proteins from different cellular components, involved in different molecular and biological functions could be identified. Moreover, this demonstrates the possibility of performing proteomics-based analysis in proteins recovered from challenging matrixes such as FFPE tissues.


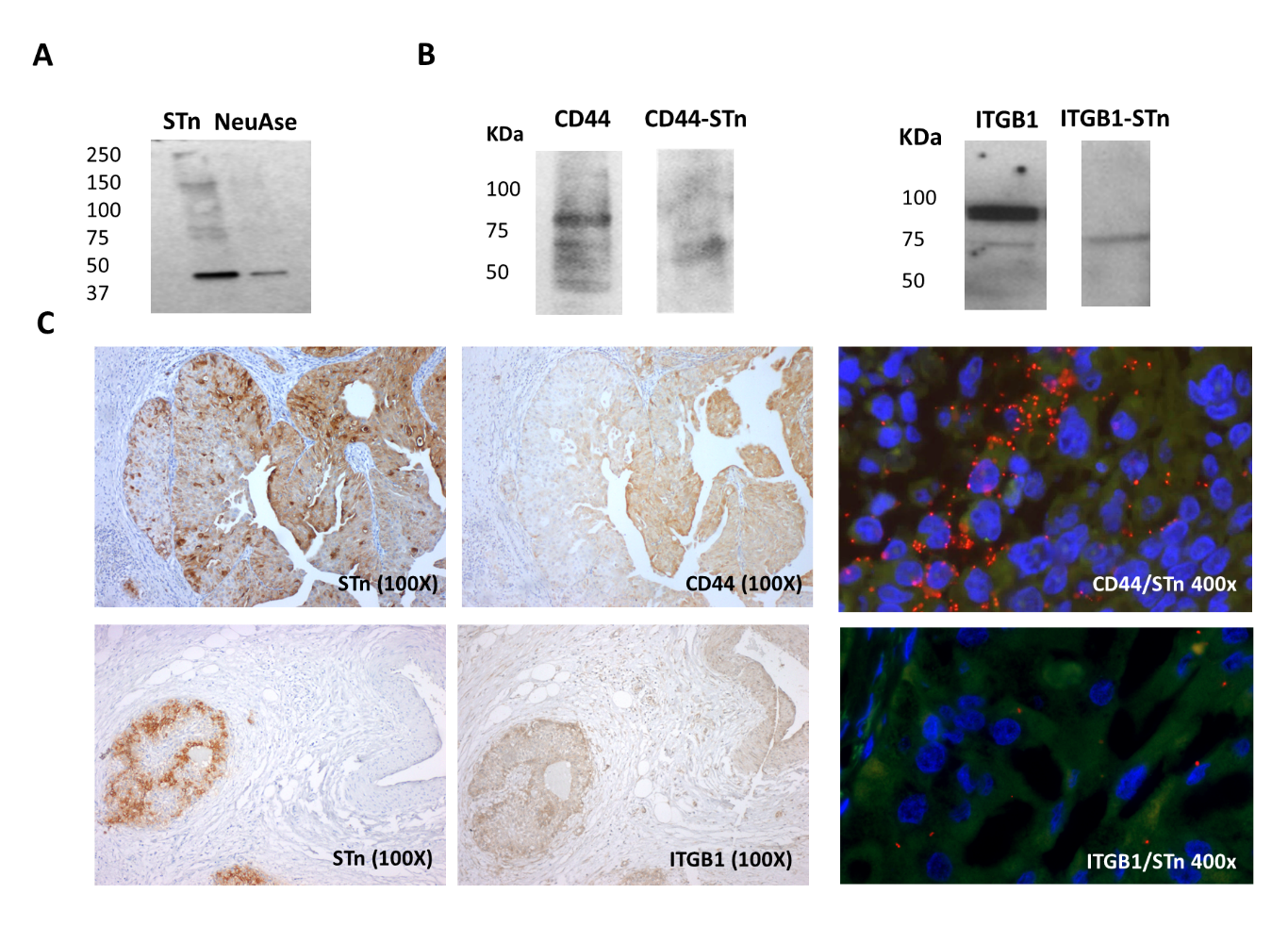
**Figure S5. A) Western blot for glycoproteins expressing the STn antigen in advanced bladder tumours.** All studied tumours (n=5) presented a western blot pattern similar to the one presented in the right lane. The signals were sensitive to neuraminidase treatment confirming the specificity of the signal (left lane). **B) Identification of STn glycoforms in CD44 and ITGB1 glycoproteins isolated from advanced bladder tumours by immunoprecipitation.** CD44 and ITGB1 were immunoprecipitated from protein extracts using antibody-immobilized agarose beads and blotted for CD44 and ITGB1 and STn thereafter. This was a neuramidase-sensitive signal (data not shown), confirming the presence of the antigen. CD44 presents a strong signal just bellow 100 kDa, corresponding to the most expressed splice variant, and lower intensity signals derived from different glycoforms and less expressed splice variants. The CD44 STn-glycoforms could be found between 50 and 75 kDa, in accordance with protein species presenting less extended glycans. ITGB1 presented an intense band at apprimately at 100 kDa, most likely derived from highly glycosylated forms of the protein, and low intensity bands approximately at 75 kDa, presumably originated by variants carrying low molecular weight glycans. Accordingly, the STn antigen was detected in the lower molecular weight protein forms. Alltogther these findings confirm that CD44 and ITGB1 are substituted with the STn antigen in advanced bladder tumours, in aggremment with glycoproteomics analysis. **C) Immunohistochemistry and PLA for CD44, ITGB1 and STn in bladder tumours.** Immunohistochemistry (left and midle panels) show a co-localization between CD44 and ITGB1 and STn expressions. The red dots in the right panel are generated from positive PLA for CD44-STn and ITGB1-STn, confirming these assignments.


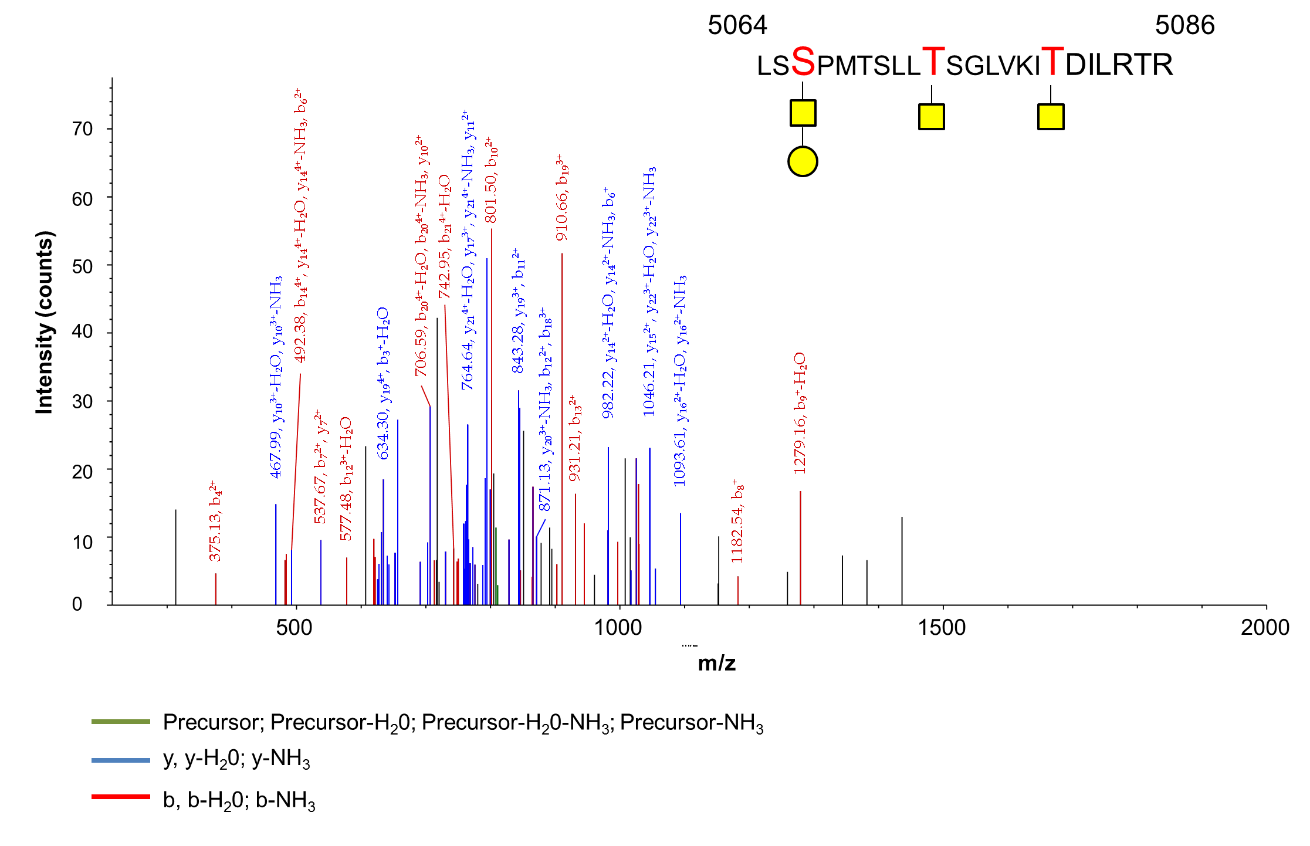

**Figure S6. Annotated nanoLC-ESI-LTQ-orbitrap-CID-MS/MS spectra for a MUC16 glycopeptide substituted with a HexNAc and HexNAc-Hex residues evidencing the specific glycosites (highlighted in the assignment table bellow).**

**Figure S7. Association between MUC16 classification by immunohistochemistry in FFPE cancer tissues (IHC; negative vs positive) and *MUC16* expression.** The graph clearly demonstrates an overexpression of *MUC16* in tumours considered positive for this glycoprotein.

**Table S1.** Proteins identified with high confidence level in Tn-negative, blood group A negative, STn-positive tumour samples recovered from formalin-fixed paraffin embedded tissues.

| Accession | Description |
| --- | --- |
| O14874 | [3-methyl-2-oxobutanoate dehydrogenase [lipoamide]] kinase, mitochondrial OS=Homo sapiens GN=BCKDK PE=1 SV=2 - [BCKD_HUMAN] |
| Q15118 | [Pyruvate dehydrogenase (acetyl-transferring)] kinase isozyme 1, mitochondrial OS=Homo sapiens GN=PDK1 PE=1 SV=1 - [PDK1_HUMAN] |
| P31947 | 14-3-3 protein sigma OS=Homo sapiens GN=SFN PE=1 SV=1 - [1433S_HUMAN] |
| P63104 | 14-3-3 protein zeta/delta OS=Homo sapiens GN=YWHAZ PE=1 SV=1 - [1433Z_HUMAN] |
| Q9NRZ7 | 1-acyl-sn-glycerol-3-phosphate acyltransferase gamma OS=Homo sapiens GN=AGPAT3 PE=1 SV=1 - [PLCC_HUMAN] |
| Q9Y2I7 | 1-phosphatidylinositol 3-phosphate 5-kinase OS=Homo sapiens GN=PIKFYVE PE=1 SV=3 - [FYV1_HUMAN] |
| Q00722 | 1-phosphatidylinositol 4,5-bisphosphate phosphodiesterase beta-2 OS=Homo sapiens GN=PLCB2 PE=1 SV=2 - [PLCB2_HUMAN] |
| Q9BRC7 | 1-phosphatidylinositol 4,5-bisphosphate phosphodiesterase delta-4 OS=Homo sapiens GN=PLCD4 PE=2 SV=1 - [PLCD4_HUMAN] |
| P16885 | 1-phosphatidylinositol 4,5-bisphosphate phosphodiesterase gamma-2 OS=Homo sapiens GN=PLCG2 PE=1 SV=4 - [PLCG2_HUMAN] |
| Q6L8Q7 | 2',5'-phosphodiesterase 12 OS=Homo sapiens GN=PDE12 PE=1 SV=2 - [PDE12_HUMAN] |
| Q9NYL5 | 24-hydroxycholesterol 7-alpha-hydroxylase OS=Homo sapiens GN=CYP39A1 PE=2 SV=2 - [CP39A_HUMAN] |
| P29728 | 2'-5'-oligoadenylate synthase 2 OS=Homo sapiens GN=OAS2 PE=1 SV=3 - [OAS2_HUMAN] |
| P17980 | 26S protease regulatory subunit 6A OS=Homo sapiens GN=PSMC3 PE=1 SV=3 - [PRS6A_HUMAN] |
| Q99460 | 26S proteasome non-ATPase regulatory subunit 1 OS=Homo sapiens GN=PSMD1 PE=1 SV=2 - [PSMD1_HUMAN] |
| O00232 | 26S proteasome non-ATPase regulatory subunit 12 OS=Homo sapiens GN=PSMD12 PE=1 SV=3 - [PSD12_HUMAN] |
| Q16401 | 26S proteasome non-ATPase regulatory subunit 5 OS=Homo sapiens GN=PSMD5 PE=1 SV=3 - [PSMD5_HUMAN] |
| Q15008 | 26S proteasome non-ATPase regulatory subunit 6 OS=Homo sapiens GN=PSMD6 PE=1 SV=1 - [PSMD6_HUMAN] |
| P82664 | 28S ribosomal protein S10, mitochondrial OS=Homo sapiens GN=MRPS10 PE=1 SV=2 - [RT10_HUMAN] |
| P82912 | 28S ribosomal protein S11, mitochondrial OS=Homo sapiens GN=MRPS11 PE=1 SV=2 - [RT11_HUMAN] |
| P82650 | 28S ribosomal protein S22, mitochondrial OS=Homo sapiens GN=MRPS22 PE=1 SV=1 - [RT22_HUMAN] |
| Q92552 | 28S ribosomal protein S27, mitochondrial OS=Homo sapiens GN=MRPS27 PE=1 SV=3 - [RT27_HUMAN] |
| P12694 | 2-oxoisovalerate dehydrogenase subunit alpha, mitochondrial OS=Homo sapiens GN=BCKDHA PE=1 SV=2 - [ODBA_HUMAN] |
| Q9H0U6 | 39S ribosomal protein L18, mitochondrial OS=Homo sapiens GN=MRPL18 PE=1 SV=1 - [RM18_HUMAN] |
| Q9P0M9 | 39S ribosomal protein L27, mitochondrial OS=Homo sapiens GN=MRPL27 PE=1 SV=1 - [RM27_HUMAN] |
| Q9BYD2 | 39S ribosomal protein L9, mitochondrial OS=Homo sapiens GN=MRPL9 PE=1 SV=2 - [RM09_HUMAN] |
| P04035 | 3-hydroxy-3-methylglutaryl-coenzyme A reductase OS=Homo sapiens GN=HMGCR PE=1 SV=1 - [HMDH_HUMAN] |
| Q9BUT1 | 3-hydroxybutyrate dehydrogenase type 2 OS=Homo sapiens GN=BDH2 PE=1 SV=2 - [BDH2_HUMAN] |
| Q6NVY1 | 3-hydroxyisobutyryl-CoA hydrolase, mitochondrial OS=Homo sapiens GN=HIBCH PE=1 SV=2 - [HIBCH_HUMAN] |
| P62263 | 40S ribosomal protein S14 OS=Homo sapiens GN=RPS14 PE=1 SV=3 - [RS14_HUMAN] |
| P62249 | 40S ribosomal protein S16 OS=Homo sapiens GN=RPS16 PE=1 SV=2 - [RS16_HUMAN] |
| P62269 | 40S ribosomal protein S18 OS=Homo sapiens GN=RPS18 PE=1 SV=3 - [RS18_HUMAN] |
| P15880 | 40S ribosomal protein S2 OS=Homo sapiens GN=RPS2 PE=1 SV=2 - [RS2_HUMAN] |
| P60866 | 40S ribosomal protein S20 OS=Homo sapiens GN=RPS20 PE=1 SV=1 - [RS20_HUMAN] |
| P62857 | 40S ribosomal protein S28 OS=Homo sapiens GN=RPS28 PE=1 SV=1 - [RS28_HUMAN] |
| P23396 | 40S ribosomal protein S3 OS=Homo sapiens GN=RPS3 PE=1 SV=2 - [RS3_HUMAN] |
| Q8TD47 | 40S ribosomal protein S4, Y isoform 2 OS=Homo sapiens GN=RPS4Y2 PE=2 SV=3 - [RS4Y2_HUMAN] |
| P62081 | 40S ribosomal protein S7 OS=Homo sapiens GN=RPS7 PE=1 SV=1 - [RS7_HUMAN] |
| P46781 | 40S ribosomal protein S9 OS=Homo sapiens GN=RPS9 PE=1 SV=3 - [RS9_HUMAN] |
| P13196 | 5-aminolevulinate synthase, nonspecific, mitochondrial OS=Homo sapiens GN=ALAS1 PE=1 SV=2 - [HEM1_HUMAN] |
| P30939 | 5-hydroxytryptamine receptor 1F OS=Homo sapiens GN=HTR1F PE=2 SV=1 - [5HT1F_HUMAN] |
| P28223 | 5-hydroxytryptamine receptor 2A OS=Homo sapiens GN=HTR2A PE=1 SV=2 - [5HT2A_HUMAN] |
| Q86UY8 | 5'-nucleotidase domain-containing protein 3 OS=Homo sapiens GN=NT5DC3 PE=2 SV=1 - [NT5D3_HUMAN] |
| P10809 | 60 kDa heat shock protein, mitochondrial OS=Homo sapiens GN=HSPD1 PE=1 SV=2 - [CH60_HUMAN] |
| P30050 | 60S ribosomal protein L12 OS=Homo sapiens GN=RPL12 PE=1 SV=1 - [RL12_HUMAN] |
| P83731 | 60S ribosomal protein L24 OS=Homo sapiens GN=RPL24 PE=1 SV=1 - [RL24_HUMAN] |
| P62888 | 60S ribosomal protein L30 OS=Homo sapiens GN=RPL30 PE=1 SV=2 - [RL30_HUMAN] |
| P36578 | 60S ribosomal protein L4 OS=Homo sapiens GN=RPL4 PE=1 SV=5 - [RL4_HUMAN] |
| Q02878 | 60S ribosomal protein L6 OS=Homo sapiens GN=RPL6 PE=1 SV=3 - [RL6_HUMAN] |
| Q7L2J0 | 7SK snRNA methylphosphate capping enzyme OS=Homo sapiens GN=MEPCE PE=1 SV=1 - [MEPCE_HUMAN] |
| P58397 | A disintegrin and metalloproteinase with thrombospondin motifs 12 OS=Homo sapiens GN=ADAMTS12 PE=1 SV=2 - [ATS12_HUMAN] |
| Q8WXS8 | A disintegrin and metalloproteinase with thrombospondin motifs 14 OS=Homo sapiens GN=ADAMTS14 PE=2 SV=2 - [ATS14_HUMAN] |
| Q9UNA0 | A disintegrin and metalloproteinase with thrombospondin motifs 5 OS=Homo sapiens GN=ADAMTS5 PE=1 SV=2 - [ATS5_HUMAN] |
| Q9UKP5 | A disintegrin and metalloproteinase with thrombospondin motifs 6 OS=Homo sapiens GN=ADAMTS6 PE=2 SV=2 - [ATS6_HUMAN] |
| Q9P2N4 | A disintegrin and metalloproteinase with thrombospondin motifs 9 OS=Homo sapiens GN=ADAMTS9 PE=1 SV=4 - [ATS9_HUMAN] |
| Q7Z5M8 | Abhydrolase domain-containing protein 12B OS=Homo sapiens GN=ABHD12B PE=2 SV=1 - [AB12B_HUMAN] |
| Q8IZP0 | Abl interactor 1 OS=Homo sapiens GN=ABI1 PE=1 SV=4 - [ABI1_HUMAN] |
| Q8IZT6 | Abnormal spindle-like microcephaly-associated protein OS=Homo sapiens GN=ASPM PE=1 SV=2 - [ASPM_HUMAN] |
| Q04844 | Acetylcholine receptor subunit epsilon OS=Homo sapiens GN=CHRNE PE=1 SV=2 - [ACHE_HUMAN] |
| Q13085 | Acetyl-CoA carboxylase 1 OS=Homo sapiens GN=ACACA PE=1 SV=2 - [ACACA_HUMAN] |
| Q9NUB1 | Acetyl-coenzyme A synthetase 2-like, mitochondrial OS=Homo sapiens GN=ACSS1 PE=1 SV=2 - [ACS2L_HUMAN] |
| Q9NR19 | Acetyl-coenzyme A synthetase, cytoplasmic OS=Homo sapiens GN=ACSS2 PE=1 SV=1 - [ACSA_HUMAN] |
| P39687 | Acidic leucine-rich nuclear phosphoprotein 32 family member A OS=Homo sapiens GN=ANP32A PE=1 SV=1 - [AN32A_HUMAN] |
| Q9BTT0 | Acidic leucine-rich nuclear phosphoprotein 32 family member E OS=Homo sapiens GN=ANP32E PE=1 SV=1 - [AN32E_HUMAN] |
| P10323 | Acrosin OS=Homo sapiens GN=ACR PE=2 SV=4 - [ACRO_HUMAN] |
| P60709 | Actin, cytoplasmic 1 OS=Homo sapiens GN=ACTB PE=1 SV=1 - [ACTB_HUMAN] |
| P61160 | Actin-related protein 2 OS=Homo sapiens GN=ACTR2 PE=1 SV=1 - [ARP2_HUMAN] |
| O15144 | Actin-related protein 2/3 complex subunit 2 OS=Homo sapiens GN=ARPC2 PE=1 SV=1 - [ARPC2_HUMAN] |
| Q8N3C0 | Activating signal cointegrator 1 complex subunit 3 OS=Homo sapiens GN=ASCC3 PE=1 SV=3 - [ASCC3_HUMAN] |
| Q6VMQ6 | Activating transcription factor 7-interacting protein 1 OS=Homo sapiens GN=ATF7IP PE=1 SV=3 - [MCAF1_HUMAN] |
| Q9NUZ1 | Acyl-coenzyme A oxidase-like protein OS=Homo sapiens GN=ACOXL PE=2 SV=3 - [ACOXL_HUMAN] |
| P0C7M7 | Acyl-coenzyme A synthetase ACSM4, mitochondrial OS=Homo sapiens GN=ACSM4 PE=2 SV=1 - [ACSM4_HUMAN] |
| Q8WYK0 | Acyl-coenzyme A thioesterase 12 OS=Homo sapiens GN=ACOT12 PE=1 SV=1 - [ACO12_HUMAN] |
| P28039 | Acyloxyacyl hydrolase OS=Homo sapiens GN=AOAH PE=1 SV=1 - [AOAH_HUMAN] |
| Q8N6G6 | ADAMTS-like protein 1 OS=Homo sapiens GN=ADAMTSL1 PE=1 SV=4 - [ATL1_HUMAN] |
| O43734 | Adapter protein CIKS OS=Homo sapiens GN=TRAF3IP2 PE=1 SV=3 - [CIKS_HUMAN] |
| O95996 | Adenomatous polyposis coli protein 2 OS=Homo sapiens GN=APC2 PE=1 SV=1 - [APC2_HUMAN] |
| Q6DHV7 | Adenosine deaminase-like protein OS=Homo sapiens GN=ADAL PE=2 SV=2 - [ADAL_HUMAN] |
| Q96PN6 | Adenylate cyclase type 10 OS=Homo sapiens GN=ADCY10 PE=1 SV=3 - [ADCYA_HUMAN] |
| P30566 | Adenylosuccinate lyase OS=Homo sapiens GN=ADSL PE=1 SV=2 - [PUR8_HUMAN] |
| P30520 | Adenylosuccinate synthetase isozyme 2 OS=Homo sapiens GN=ADSS PE=1 SV=3 - [PURA2_HUMAN] |
| Q01518 | Adenylyl cyclase-associated protein 1 OS=Homo sapiens GN=CAP1 PE=1 SV=5 - [CAP1_HUMAN] |
| P40123 | Adenylyl cyclase-associated protein 2 OS=Homo sapiens GN=CAP2 PE=1 SV=1 - [CAP2_HUMAN] |
| Q8IWK6 | Adhesion G protein-coupled receptor A3 OS=Homo sapiens GN=ADGRA3 PE=1 SV=2 - [AGRA3_HUMAN] |
| Q8IZF6 | Adhesion G-protein coupled receptor G4 OS=Homo sapiens GN=ADGRG4 PE=2 SV=2 - [AGRG4_HUMAN] |
| P18085 | ADP-ribosylation factor 4 OS=Homo sapiens GN=ARF4 PE=1 SV=3 - [ARF4_HUMAN] |
| Q8N4G2 | ADP-ribosylation factor-like protein 14 OS=Homo sapiens GN=ARL14 PE=1 SV=2 - [ARL14_HUMAN] |
| P51825 | AF4/FMR2 family member 1 OS=Homo sapiens GN=AFF1 PE=1 SV=1 - [AFF1_HUMAN] |
| Q9UHB7 | AF4/FMR2 family member 4 OS=Homo sapiens GN=AFF4 PE=1 SV=1 - [AFF4_HUMAN] |
| P55196 | Afadin OS=Homo sapiens GN=MLLT4 PE=1 SV=3 - [AFAD_HUMAN] |
| Q9Y4W6 | AFG3-like protein 2 OS=Homo sapiens GN=AFG3L2 PE=1 SV=2 - [AFG32_HUMAN] |
| Q6ULP2 | Aftiphilin OS=Homo sapiens GN=AFTPH PE=1 SV=2 - [AFTIN_HUMAN] |
| O00468 | Agrin OS=Homo sapiens GN=AGRN PE=1 SV=5 - [AGRIN_HUMAN] |
| O43572 | A-kinase anchor protein 10, mitochondrial OS=Homo sapiens GN=AKAP10 PE=1 SV=2 - [AKA10_HUMAN] |
| Q9UKA4 | A-kinase anchor protein 11 OS=Homo sapiens GN=AKAP11 PE=1 SV=1 - [AKA11_HUMAN] |
| Q12802 | A-kinase anchor protein 13 OS=Homo sapiens GN=AKAP13 PE=1 SV=2 - [AKP13_HUMAN] |
| O75969 | A-kinase anchor protein 3 OS=Homo sapiens GN=AKAP3 PE=1 SV=2 - [AKAP3_HUMAN] |
| Q5JQC9 | A-kinase anchor protein 4 OS=Homo sapiens GN=AKAP4 PE=1 SV=1 - [AKAP4_HUMAN] |
| Q99996 | A-kinase anchor protein 9 OS=Homo sapiens GN=AKAP9 PE=1 SV=3 - [AKAP9_HUMAN] |
| Q9NQ31 | A-kinase-interacting protein 1 OS=Homo sapiens GN=AKIP1 PE=1 SV=2 - [AKIP1_HUMAN] |
| P43353 | Aldehyde dehydrogenase family 3 member B1 OS=Homo sapiens GN=ALDH3B1 PE=1 SV=1 - [AL3B1_HUMAN] |
| P30838 | Aldehyde dehydrogenase, dimeric NADP-preferring OS=Homo sapiens GN=ALDH3A1 PE=1 SV=3 - [AL3A1_HUMAN] |
| C9JRZ8 | Aldo-keto reductase family 1 member B15 OS=Homo sapiens GN=AKR1B15 PE=1 SV=2 - [AK1BF_HUMAN] |
| Q9UM73 | ALK tyrosine kinase receptor OS=Homo sapiens GN=ALK PE=1 SV=3 - [ALK_HUMAN] |
| P10696 | Alkaline phosphatase, placental-like OS=Homo sapiens GN=ALPPL2 PE=2 SV=4 - [PPBN_HUMAN] |
| P05186 | Alkaline phosphatase, tissue-nonspecific isozyme OS=Homo sapiens GN=ALPL PE=1 SV=4 - [PPBT_HUMAN] |
| Q13686 | Alkylated DNA repair protein alkB homolog 1 OS=Homo sapiens GN=ALKBH1 PE=1 SV=2 - [ALKB1_HUMAN] |
| Q7Z6M3 | Allergin-1 OS=Homo sapiens GN=MILR1 PE=1 SV=2 - [MILR1_HUMAN] |
| Q6P4F1 | Alpha-(1,3)-fucosyltransferase 10 OS=Homo sapiens GN=FUT10 PE=2 SV=2 - [FUT10_HUMAN] |
| Q96IU4 | Alpha/beta hydrolase domain-containing protein 14B OS=Homo sapiens GN=ABHD14B PE=1 SV=1 - [ABHEB_HUMAN] |
| Q9H553 | Alpha-1,3/1,6-mannosyltransferase ALG2 OS=Homo sapiens GN=ALG2 PE=1 SV=1 - [ALG2_HUMAN] |
| Q9UQ53 | Alpha-1,3-mannosyl-glycoprotein 4-beta-N-acetylglucosaminyltransferase B OS=Homo sapiens GN=MGAT4B PE=1 SV=1 - [MGT4B_HUMAN] |
| Q3V5L5 | Alpha-1,6-mannosylglycoprotein 6-beta-N-acetylglucosaminyltransferase B OS=Homo sapiens GN=MGAT5B PE=1 SV=2 - [MGT5B_HUMAN] |
| P06733 | Alpha-enolase OS=Homo sapiens GN=ENO1 PE=1 SV=2 - [ENOA_HUMAN] |
| Q9NSC7 | Alpha-N-acetylgalactosaminide alpha-2,6-sialyltransferase 1 OS=Homo sapiens GN=ST6GALNAC1 PE=2 SV=1 - [SIA7A_HUMAN] |
| P54802 | Alpha-N-acetylglucosaminidase OS=Homo sapiens GN=NAGLU PE=1 SV=2 - [ANAG_HUMAN] |
| Q96L96 | Alpha-protein kinase 3 OS=Homo sapiens GN=ALPK3 PE=2 SV=2 - [ALPK3_HUMAN] |
| O75443 | Alpha-tectorin OS=Homo sapiens GN=TECTA PE=1 SV=3 - [TECTA_HUMAN] |
| P51170 | Amiloride-sensitive sodium channel subunit gamma OS=Homo sapiens GN=SCNN1G PE=1 SV=4 - [SCNNG_HUMAN] |
| P21397 | Amine oxidase [flavin-containing] A OS=Homo sapiens GN=MAOA PE=1 SV=1 - [AOFA_HUMAN] |
| P23109 | AMP deaminase 1 OS=Homo sapiens GN=AMPD1 PE=1 SV=2 - [AMPD1_HUMAN] |
| Q01433 | AMP deaminase 2 OS=Homo sapiens GN=AMPD2 PE=1 SV=2 - [AMPD2_HUMAN] |
| P15514 | Amphiregulin OS=Homo sapiens GN=AREG PE=1 SV=2 - [AREG_HUMAN] |
| Q7Z5R6 | Amyloid beta A4 precursor protein-binding family B member 1-interacting protein OS=Homo sapiens GN=APBB1IP PE=1 SV=1 - [AB1IP_HUMAN] |
| Q92624 | Amyloid protein-binding protein 2 OS=Homo sapiens GN=APPBP2 PE=1 SV=2 - [APBP2_HUMAN] |
| Q8N6M9 | AN1-type zinc finger protein 2A OS=Homo sapiens GN=ZFAND2A PE=2 SV=2 - [ZFN2A_HUMAN] |
| Q8N302 | Angiogenic factor with G patch and FHA domains 1 OS=Homo sapiens GN=AGGF1 PE=1 SV=2 - [AGGF1_HUMAN] |
| Q02763 | Angiopoietin-1 receptor OS=Homo sapiens GN=TEK PE=1 SV=2 - [TIE2_HUMAN] |
| Q9Y264 | Angiopoietin-4 OS=Homo sapiens GN=ANGPT4 PE=1 SV=1 - [ANGP4_HUMAN] |
| P12821 | Angiotensin-converting enzyme OS=Homo sapiens GN=ACE PE=1 SV=1 - [ACE_HUMAN] |
| Q96Q91 | Anion exchange protein 4 OS=Homo sapiens GN=SLC4A9 PE=2 SV=2 - [B3A4_HUMAN] |
| Q9P0K7 | Ankycorbin OS=Homo sapiens GN=RAI14 PE=1 SV=2 - [RAI14_HUMAN] |
| Q8N961 | Ankyrin repeat and BTB/POZ domain-containing protein 2 OS=Homo sapiens GN=ABTB2 PE=2 SV=2 - [ABTB2_HUMAN] |
| A6NHY2 | Ankyrin repeat and death domain-containing protein 1B OS=Homo sapiens GN=ANKDD1B PE=3 SV=3 - [AKD1B_HUMAN] |
| Q8IWZ3 | Ankyrin repeat and KH domain-containing protein 1 OS=Homo sapiens GN=ANKHD1 PE=1 SV=1 - [ANKH1_HUMAN] |
| Q8NFD2 | Ankyrin repeat and protein kinase domain-containing protein 1 OS=Homo sapiens GN=ANKK1 PE=2 SV=1 - [ANKK1_HUMAN] |
| Q92625 | Ankyrin repeat and SAM domain-containing protein 1A OS=Homo sapiens GN=ANKS1A PE=1 SV=4 - [ANS1A_HUMAN] |
| Q96Q27 | Ankyrin repeat and SOCS box protein 2 OS=Homo sapiens GN=ASB2 PE=1 SV=1 - [ASB2_HUMAN] |
| Q6UB99 | Ankyrin repeat domain-containing protein 11 OS=Homo sapiens GN=ANKRD11 PE=1 SV=3 - [ANR11_HUMAN] |
| Q4UJ75 | Ankyrin repeat domain-containing protein 20A4 OS=Homo sapiens GN=ANKRD20A4 PE=3 SV=1 - [A20A4_HUMAN] |
| Q8N2N9 | Ankyrin repeat domain-containing protein 36B OS=Homo sapiens GN=ANKRD36B PE=1 SV=4 - [AN36B_HUMAN] |
| Q5TZF3 | Ankyrin repeat domain-containing protein 45 OS=Homo sapiens GN=ANKRD45 PE=1 SV=1 - [ANR45_HUMAN] |
| Q8WVL7 | Ankyrin repeat domain-containing protein 49 OS=Homo sapiens GN=ANKRD49 PE=1 SV=1 - [ANR49_HUMAN] |
| Q9Y2G4 | Ankyrin repeat domain-containing protein 6 OS=Homo sapiens GN=ANKRD6 PE=1 SV=3 - [ANKR6_HUMAN] |
| Q9BZ19 | Ankyrin repeat domain-containing protein 60 OS=Homo sapiens GN=ANKRD60 PE=3 SV=3 - [ANR60_HUMAN] |
| Q96BM1 | Ankyrin repeat domain-containing protein 9 OS=Homo sapiens GN=ANKRD9 PE=2 SV=1 - [ANKR9_HUMAN] |
| Q01484 | Ankyrin-2 OS=Homo sapiens GN=ANK2 PE=1 SV=4 - [ANK2_HUMAN] |
| Q12955 | Ankyrin-3 OS=Homo sapiens GN=ANK3 PE=1 SV=3 - [ANK3_HUMAN] |
| P08758 | Annexin A5 OS=Homo sapiens GN=ANXA5 PE=1 SV=2 - [ANXA5_HUMAN] |
| Q32M45 | Anoctamin-4 OS=Homo sapiens GN=ANO4 PE=2 SV=1 - [ANO4_HUMAN] |
| P23352 | Anosmin-1 OS=Homo sapiens GN=KAL1 PE=1 SV=3 - [KALM_HUMAN] |
| P46013 | Antigen KI-67 OS=Homo sapiens GN=MKI67 PE=1 SV=2 - [KI67_HUMAN] |
| O00203 | AP-3 complex subunit beta-1 OS=Homo sapiens GN=AP3B1 PE=1 SV=3 - [AP3B1_HUMAN] |
| Q96N21 | AP-4 complex accessory subunit tepsin OS=Homo sapiens GN=ENTHD2 PE=1 SV=1 - [AP4AT_HUMAN] |
| Q9UPM8 | AP-4 complex subunit epsilon-1 OS=Homo sapiens GN=AP4E1 PE=1 SV=2 - [AP4E1_HUMAN] |
| Q9H0R1 | AP-5 complex subunit mu-1 OS=Homo sapiens GN=AP5M1 PE=1 SV=2 - [AP5M1_HUMAN] |
| Q8N7J2 | APC membrane recruitment protein 2 OS=Homo sapiens GN=AMER2 PE=1 SV=3 - [AMER2_HUMAN] |
| Q0VD83 | Apolipoprotein B receptor OS=Homo sapiens GN=APOBR PE=1 SV=2 - [APOBR_HUMAN] |
| P04114 | Apolipoprotein B-100 OS=Homo sapiens GN=APOB PE=1 SV=2 - [APOB_HUMAN] |
| Q9BPW4 | Apolipoprotein L4 OS=Homo sapiens GN=APOL4 PE=2 SV=3 - [APOL4_HUMAN] |
| Q9BWW9 | Apolipoprotein L5 OS=Homo sapiens GN=APOL5 PE=2 SV=1 - [APOL5_HUMAN] |
| Q96NN9 | Apoptosis-inducing factor 3 OS=Homo sapiens GN=AIFM3 PE=1 SV=1 - [AIFM3_HUMAN] |
| Q9UKV3 | Apoptotic chromatin condensation inducer in the nucleus OS=Homo sapiens GN=ACIN1 PE=1 SV=2 - [ACINU_HUMAN] |
| Q9NPF8 | Arf-GAP with dual PH domain-containing protein 2 OS=Homo sapiens GN=ADAP2 PE=1 SV=1 - [ADAP2_HUMAN] |
| Q96P48 | Arf-GAP with Rho-GAP domain, ANK repeat and PH domain-containing protein 1 OS=Homo sapiens GN=ARAP1 PE=1 SV=3 - [ARAP1_HUMAN] |
| Q8WZ64 | Arf-GAP with Rho-GAP domain, ANK repeat and PH domain-containing protein 2 OS=Homo sapiens GN=ARAP2 PE=1 SV=3 - [ARAP2_HUMAN] |
| Q8WWN8 | Arf-GAP with Rho-GAP domain, ANK repeat and PH domain-containing protein 3 OS=Homo sapiens GN=ARAP3 PE=1 SV=1 - [ARAP3_HUMAN] |
| O00192 | Armadillo repeat protein deleted in velo-cardio-facial syndrome OS=Homo sapiens GN=ARVCF PE=1 SV=1 - [ARVC_HUMAN] |
| Q5W041 | Armadillo repeat-containing protein 3 OS=Homo sapiens GN=ARMC3 PE=2 SV=2 - [ARMC3_HUMAN] |
| Q8NCT1 | Arrestin domain-containing protein 4 OS=Homo sapiens GN=ARRDC4 PE=2 SV=3 - [ARRD4_HUMAN] |
| O00327 | Aryl hydrocarbon receptor nuclear translocator-like protein 1 OS=Homo sapiens GN=ARNTL PE=1 SV=2 - [BMAL1_HUMAN] |
| P15289 | Arylsulfatase A OS=Homo sapiens GN=ARSA PE=1 SV=3 - [ARSA_HUMAN] |
| P08243 | Asparagine synthetase [glutamine-hydrolyzing] OS=Homo sapiens GN=ASNS PE=1 SV=4 - [ASNS_HUMAN] |
| Q9NWL6 | Asparagine synthetase domain-containing protein 1 OS=Homo sapiens GN=ASNSD1 PE=2 SV=2 - [ASND1_HUMAN] |
| Q6PI48 | Aspartate--tRNA ligase, mitochondrial OS=Homo sapiens GN=DARS2 PE=1 SV=1 - [SYDM_HUMAN] |
| Q9BXN1 | Asporin OS=Homo sapiens GN=ASPN PE=1 SV=2 - [ASPN_HUMAN] |
| O75129 | Astrotactin-2 OS=Homo sapiens GN=ASTN2 PE=2 SV=2 - [ASTN2_HUMAN] |
| O15265 | Ataxin-7 OS=Homo sapiens GN=ATXN7 PE=1 SV=1 - [ATX7_HUMAN] |
| Q14CW9 | Ataxin-7-like protein 3 OS=Homo sapiens GN=ATXN7L3 PE=1 SV=1 - [AT7L3_HUMAN] |
| Q5TGY3 | AT-hook DNA-binding motif-containing protein 1 OS=Homo sapiens GN=AHDC1 PE=1 SV=1 - [AHDC1_HUMAN] |
| Q6DD88 | Atlastin-3 OS=Homo sapiens GN=ATL3 PE=1 SV=1 - [ATLA3_HUMAN] |
| O43313 | ATM interactor OS=Homo sapiens GN=ATMIN PE=1 SV=2 - [ATMIN_HUMAN] |
| P25705 | ATP synthase subunit alpha, mitochondrial OS=Homo sapiens GN=ATP5A1 PE=1 SV=1 - [ATPA_HUMAN] |
| P06576 | ATP synthase subunit beta, mitochondrial OS=Homo sapiens GN=ATP5B PE=1 SV=3 - [ATPB_HUMAN] |
| Q96QE3 | ATPase family AAA domain-containing protein 5 OS=Homo sapiens GN=ATAD5 PE=1 SV=4 - [ATAD5_HUMAN] |
| O95477 | ATP-binding cassette sub-family A member 1 OS=Homo sapiens GN=ABCA1 PE=1 SV=3 - [ABCA1_HUMAN] |
| Q8WWZ4 | ATP-binding cassette sub-family A member 10 OS=Homo sapiens GN=ABCA10 PE=2 SV=3 - [ABCAA_HUMAN] |
| Q86UK0 | ATP-binding cassette sub-family A member 12 OS=Homo sapiens GN=ABCA12 PE=1 SV=3 - [ABCAC_HUMAN] |
| O94911 | ATP-binding cassette sub-family A member 8 OS=Homo sapiens GN=ABCA8 PE=1 SV=3 - [ABCA8_HUMAN] |
| Q9NP58 | ATP-binding cassette sub-family B member 6, mitochondrial OS=Homo sapiens GN=ABCB6 PE=1 SV=1 - [ABCB6_HUMAN] |
| O75027 | ATP-binding cassette sub-family B member 7, mitochondrial OS=Homo sapiens GN=ABCB7 PE=1 SV=2 - [ABCB7_HUMAN] |
| Q09428 | ATP-binding cassette sub-family C member 8 OS=Homo sapiens GN=ABCC8 PE=1 SV=6 - [ABCC8_HUMAN] |
| O60706 | ATP-binding cassette sub-family C member 9 OS=Homo sapiens GN=ABCC9 PE=1 SV=2 - [ABCC9_HUMAN] |
| Q9H221 | ATP-binding cassette sub-family G member 8 OS=Homo sapiens GN=ABCG8 PE=1 SV=1 - [ABCG8_HUMAN] |
| P53396 | ATP-citrate synthase OS=Homo sapiens GN=ACLY PE=1 SV=3 - [ACLY_HUMAN] |
| P08237 | ATP-dependent 6-phosphofructokinase, muscle type OS=Homo sapiens GN=PFKM PE=1 SV=2 - [PFKAM_HUMAN] |
| O94761 | ATP-dependent DNA helicase Q4 OS=Homo sapiens GN=RECQL4 PE=1 SV=1 - [RECQ4_HUMAN] |
| Q08211 | ATP-dependent RNA helicase A OS=Homo sapiens GN=DHX9 PE=1 SV=4 - [DHX9_HUMAN] |
| Q9GZR7 | ATP-dependent RNA helicase DDX24 OS=Homo sapiens GN=DDX24 PE=1 SV=1 - [DDX24_HUMAN] |
| O15523 | ATP-dependent RNA helicase DDX3Y OS=Homo sapiens GN=DDX3Y PE=1 SV=2 - [DDX3Y_HUMAN] |
| Q9H2U1 | ATP-dependent RNA helicase DHX36 OS=Homo sapiens GN=DHX36 PE=1 SV=2 - [DHX36_HUMAN] |
| Q14562 | ATP-dependent RNA helicase DHX8 OS=Homo sapiens GN=DHX8 PE=1 SV=1 - [DHX8_HUMAN] |
| P16066 | Atrial natriuretic peptide receptor 1 OS=Homo sapiens GN=NPR1 PE=1 SV=1 - [ANPRA_HUMAN] |
| P20594 | Atrial natriuretic peptide receptor 2 OS=Homo sapiens GN=NPR2 PE=1 SV=1 - [ANPRB_HUMAN] |
| Q8NFD5 | AT-rich interactive domain-containing protein 1B OS=Homo sapiens GN=ARID1B PE=1 SV=2 - [ARI1B_HUMAN] |
| Q99856 | AT-rich interactive domain-containing protein 3A OS=Homo sapiens GN=ARID3A PE=1 SV=2 - [ARI3A_HUMAN] |
| Q4LE39 | AT-rich interactive domain-containing protein 4B OS=Homo sapiens GN=ARID4B PE=1 SV=2 - [ARI4B_HUMAN] |
| Q14865 | AT-rich interactive domain-containing protein 5B OS=Homo sapiens GN=ARID5B PE=1 SV=3 - [ARI5B_HUMAN] |
| Q5VV63 | Attractin-like protein 1 OS=Homo sapiens GN=ATRNL1 PE=2 SV=2 - [ATRN1_HUMAN] |
| Q9NWT8 | Aurora kinase A-interacting protein OS=Homo sapiens GN=AURKAIP1 PE=1 SV=1 - [AKIP_HUMAN] |
| Q8NAA4 | Autophagy-related protein 16-2 OS=Homo sapiens GN=ATG16L2 PE=1 SV=2 - [A16L2_HUMAN] |
| Q9Y2T1 | Axin-2 OS=Homo sapiens GN=AXIN2 PE=1 SV=1 - [AXIN2_HUMAN] |
| P20160 | Azurocidin OS=Homo sapiens GN=AZU1 PE=1 SV=3 - [CAP7_HUMAN] |
| P17213 | Bactericidal permeability-increasing protein OS=Homo sapiens GN=BPI PE=1 SV=4 - [BPI_HUMAN] |
| Q13489 | Baculoviral IAP repeat-containing protein 3 OS=Homo sapiens GN=BIRC3 PE=1 SV=2 - [BIRC3_HUMAN] |
| Q9NR09 | Baculoviral IAP repeat-containing protein 6 OS=Homo sapiens GN=BIRC6 PE=1 SV=2 - [BIRC6_HUMAN] |
| Q9P281 | BAH and coiled-coil domain-containing protein 1 OS=Homo sapiens GN=BAHCC1 PE=1 SV=3 - [BAHC1_HUMAN] |
| P02730 | Band 3 anion transport protein OS=Homo sapiens GN=SLC4A1 PE=1 SV=3 - [B3AT_HUMAN] |
| P50895 | Basal cell adhesion molecule OS=Homo sapiens GN=BCAM PE=1 SV=2 - [BCAM_HUMAN] |
| P98160 | Basement membrane-specific heparan sulfate proteoglycan core protein OS=Homo sapiens GN=HSPG2 PE=1 SV=4 - [PGBM_HUMAN] |
| Q68DE3 | Basic helix-loop-helix domain-containing protein KIAA2018 OS=Homo sapiens GN=KIAA2018 PE=1 SV=3 - [K2018_HUMAN] |
| Q9Y6E2 | Basic leucine zipper and W2 domain-containing protein 2 OS=Homo sapiens GN=BZW2 PE=1 SV=1 - [BZW2_HUMAN] |
| Q8N1L9 | Basic leucine zipper transcriptional factor ATF-like 2 OS=Homo sapiens GN=BATF2 PE=1 SV=1 - [BATF2_HUMAN] |
| Q8WV28 | B-cell linker protein OS=Homo sapiens GN=BLNK PE=1 SV=2 - [BLNK_HUMAN] |
| Q9NYF8 | Bcl-2-associated transcription factor 1 OS=Homo sapiens GN=BCLAF1 PE=1 SV=2 - [BCLF1_HUMAN] |
| Q9BXK5 | Bcl-2-like protein 13 OS=Homo sapiens GN=BCL2L13 PE=1 SV=1 - [B2L13_HUMAN] |
| O76090 | Bestrophin-1 OS=Homo sapiens GN=BEST1 PE=1 SV=1 - [BEST1_HUMAN] |
| Q8N1M1 | Bestrophin-3 OS=Homo sapiens GN=BEST3 PE=2 SV=1 - [BEST3_HUMAN] |
| O95395 | Beta-1,3-galactosyl-O-glycosyl-glycoprotein beta-1,6-N-acetylglucosaminyltransferase 3 OS=Homo sapiens GN=GCNT3 PE=2 SV=1 - [GCNT3_HUMAN] |
| Q9P109 | Beta-1,3-galactosyl-O-glycosyl-glycoprotein beta-1,6-N-acetylglucosaminyltransferase 4 OS=Homo sapiens GN=GCNT4 PE=2 SV=1 - [GCNT4_HUMAN] |
| Q00973 | Beta-1,4 N-acetylgalactosaminyltransferase 1 OS=Homo sapiens GN=B4GALNT1 PE=1 SV=2 - [B4GN1_HUMAN] |
| O60512 | Beta-1,4-galactosyltransferase 3 OS=Homo sapiens GN=B4GALT3 PE=1 SV=2 - [B4GT3_HUMAN] |
| Q9UBV7 | Beta-1,4-galactosyltransferase 7 OS=Homo sapiens GN=B4GALT7 PE=1 SV=1 - [B4GT7_HUMAN] |
| P32121 | Beta-arrestin-2 OS=Homo sapiens GN=ARRB2 PE=1 SV=2 - [ARRB2_HUMAN] |
| Q8N687 | Beta-defensin 125 OS=Homo sapiens GN=DEFB125 PE=2 SV=2 - [DB125_HUMAN] |
| P13929 | Beta-enolase OS=Homo sapiens GN=ENO3 PE=1 SV=5 - [ENOB_HUMAN] |
| Q9HBI1 | Beta-parvin OS=Homo sapiens GN=PARVB PE=1 SV=1 - [PARVB_HUMAN] |
| Q6NYC1 | Bifunctional arginine demethylase and lysyl-hydroxylase JMJD6 OS=Homo sapiens GN=JMJD6 PE=1 SV=1 - [JMJD6_HUMAN] |
| Q3LXA3 | Bifunctional ATP-dependent dihydroxyacetone kinase/FAD-AMP lyase (cyclizing) OS=Homo sapiens GN=DAK PE=1 SV=2 - [DHAK_HUMAN] |
| P31939 | Bifunctional purine biosynthesis protein PURH OS=Homo sapiens GN=ATIC PE=1 SV=3 - [PUR9_HUMAN] |
| Q14032 | Bile acid-CoA:amino acid N-acyltransferase OS=Homo sapiens GN=BAAT PE=1 SV=1 - [BAAT_HUMAN] |
| P50747 | Biotin--protein ligase OS=Homo sapiens GN=HLCS PE=1 SV=1 - [BPL1_HUMAN] |
| P18577 | Blood group Rh(CE) polypeptide OS=Homo sapiens GN=RHCE PE=1 SV=2 - [RHCE_HUMAN] |
| O60477 | BMP/retinoic acid-inducible neural-specific protein 1 OS=Homo sapiens GN=BRINP1 PE=1 SV=2 - [BRNP1_HUMAN] |
| Q76B58 | BMP/retinoic acid-inducible neural-specific protein 3 OS=Homo sapiens GN=BRINP3 PE=1 SV=1 - [BRNP3_HUMAN] |
| P13727 | Bone marrow proteoglycan OS=Homo sapiens GN=PRG2 PE=1 SV=2 - [PRG2_HUMAN] |
| P13497 | Bone morphogenetic protein 1 OS=Homo sapiens GN=BMP1 PE=1 SV=2 - [BMP1_HUMAN] |
| O95393 | Bone morphogenetic protein 10 OS=Homo sapiens GN=BMP10 PE=1 SV=1 - [BMP10_HUMAN] |
| O95972 | Bone morphogenetic protein 15 OS=Homo sapiens GN=BMP15 PE=1 SV=2 - [BMP15_HUMAN] |
| P12644 | Bone morphogenetic protein 4 OS=Homo sapiens GN=BMP4 PE=1 SV=1 - [BMP4_HUMAN] |
| P80723 | Brain acid soluble protein 1 OS=Homo sapiens GN=BASP1 PE=1 SV=2 - [BASP1_HUMAN] |
| Q8WXS3 | Brain and acute leukemia cytoplasmic protein OS=Homo sapiens GN=BAALC PE=2 SV=3 - [BAALC_HUMAN] |
| Q7Z569 | BRCA1-associated protein OS=Homo sapiens GN=BRAP PE=1 SV=2 - [BRAP_HUMAN] |
| P11274 | Breakpoint cluster region protein OS=Homo sapiens GN=BCR PE=1 SV=2 - [BCR_HUMAN] |
| Q9Y6D6 | Brefeldin A-inhibited guanine nucleotide-exchange protein 1 OS=Homo sapiens GN=ARFGEF1 PE=1 SV=2 - [BIG1_HUMAN] |
| Q5TH69 | Brefeldin A-inhibited guanine nucleotide-exchange protein 3 OS=Homo sapiens GN=ARFGEF3 PE=1 SV=3 - [BIG3_HUMAN] |
| Q9ULD4 | Bromodomain and PHD finger-containing protein 3 OS=Homo sapiens GN=BRPF3 PE=1 SV=2 - [BRPF3_HUMAN] |
| O95696 | Bromodomain-containing protein 1 OS=Homo sapiens GN=BRD1 PE=1 SV=1 - [BRD1_HUMAN] |
| Q9H0E9 | Bromodomain-containing protein 8 OS=Homo sapiens GN=BRD8 PE=1 SV=2 - [BRD8_HUMAN] |
| Q9NW68 | BSD domain-containing protein 1 OS=Homo sapiens GN=BSDC1 PE=1 SV=1 - [BSDC1_HUMAN] |
| Q9H0C5 | BTB/POZ domain-containing protein 1 OS=Homo sapiens GN=BTBD1 PE=1 SV=1 - [BTBD1_HUMAN] |
| Q96Q07 | BTB/POZ domain-containing protein 9 OS=Homo sapiens GN=BTBD9 PE=2 SV=2 - [BTBD9_HUMAN] |
| Q7Z5Y7 | BTB/POZ domain-containing protein KCTD20 OS=Homo sapiens GN=KCTD20 PE=1 SV=1 - [KCD20_HUMAN] |
| Q9Y597 | BTB/POZ domain-containing protein KCTD3 OS=Homo sapiens GN=KCTD3 PE=1 SV=2 - [KCTD3_HUMAN] |
| P11586 | C-1-tetrahydrofolate synthase, cytoplasmic OS=Homo sapiens GN=MTHFD1 PE=1 SV=3 - [C1TC_HUMAN] |
| O75844 | CAAX prenyl protease 1 homolog OS=Homo sapiens GN=ZMPSTE24 PE=1 SV=2 - [FACE1_HUMAN] |
| Q8WUQ7 | Cactin OS=Homo sapiens GN=CACTIN PE=1 SV=3 - [CATIN_HUMAN] |
| Q9NYQ6 | Cadherin EGF LAG seven-pass G-type receptor 1 OS=Homo sapiens GN=CELSR1 PE=1 SV=1 - [CELR1_HUMAN] |
| Q9HCU4 | Cadherin EGF LAG seven-pass G-type receptor 2 OS=Homo sapiens GN=CELSR2 PE=1 SV=1 - [CELR2_HUMAN] |
| Q9NYQ7 | Cadherin EGF LAG seven-pass G-type receptor 3 OS=Homo sapiens GN=CELSR3 PE=1 SV=2 - [CELR3_HUMAN] |
| P55290 | Cadherin-13 OS=Homo sapiens GN=CDH13 PE=1 SV=1 - [CAD13_HUMAN] |
| Q12864 | Cadherin-17 OS=Homo sapiens GN=CDH17 PE=2 SV=3 - [CAD17_HUMAN] |
| P22223 | Cadherin-3 OS=Homo sapiens GN=CDH3 PE=1 SV=2 - [CADH3_HUMAN] |
| P55283 | Cadherin-4 OS=Homo sapiens GN=CDH4 PE=2 SV=2 - [CADH4_HUMAN] |
| Q9ULB5 | Cadherin-7 OS=Homo sapiens GN=CDH7 PE=2 SV=2 - [CADH7_HUMAN] |
| Q6ZTQ4 | Cadherin-related family member 3 OS=Homo sapiens GN=CDHR3 PE=1 SV=1 - [CDHR3_HUMAN] |
| Q99653 | Calcineurin B homologous protein 1 OS=Homo sapiens GN=CHP1 PE=1 SV=3 - [CHP1_HUMAN] |
| P49069 | Calcium signal-modulating cyclophilin ligand OS=Homo sapiens GN=CAMLG PE=1 SV=1 - [CAMLG_HUMAN] |
| Q9BPX6 | Calcium uptake protein 1, mitochondrial OS=Homo sapiens GN=MICU1 PE=1 SV=1 - [MICU1_HUMAN] |
| Q8IU85 | Calcium/calmodulin-dependent protein kinase type 1D OS=Homo sapiens GN=CAMK1D PE=1 SV=1 - [KCC1D_HUMAN] |
| Q9H9S4 | Calcium-binding protein 39-like OS=Homo sapiens GN=CAB39L PE=1 SV=3 - [CB39L_HUMAN] |
| Q9NP86 | Calcium-binding protein 5 OS=Homo sapiens GN=CABP5 PE=1 SV=1 - [CABP5_HUMAN] |
| Q9Y6Y1 | Calmodulin-binding transcription activator 1 OS=Homo sapiens GN=CAMTA1 PE=1 SV=4 - [CMTA1_HUMAN] |
| Q5T5Y3 | Calmodulin-regulated spectrin-associated protein 1 OS=Homo sapiens GN=CAMSAP1 PE=1 SV=2 - [CAMP1_HUMAN] |
| O15484 | Calpain-5 OS=Homo sapiens GN=CAPN5 PE=1 SV=2 - [CAN5_HUMAN] |
| A6NHC0 | Calpain-8 OS=Homo sapiens GN=CAPN8 PE=2 SV=3 - [CAN8_HUMAN] |
| P22612 | cAMP-dependent protein kinase catalytic subunit gamma OS=Homo sapiens GN=PRKACG PE=1 SV=3 - [KAPCG_HUMAN] |
| P61925 | cAMP-dependent protein kinase inhibitor alpha OS=Homo sapiens GN=PKIA PE=1 SV=2 - [IPKA_HUMAN] |
| Q08493 | cAMP-specific 3',5'-cyclic phosphodiesterase 4C OS=Homo sapiens GN=PDE4C PE=1 SV=2 - [PDE4C_HUMAN] |
| Q08499 | cAMP-specific 3',5'-cyclic phosphodiesterase 4D OS=Homo sapiens GN=PDE4D PE=1 SV=2 - [PDE4D_HUMAN] |
| Q92887 | Canalicular multispecific organic anion transporter 1 OS=Homo sapiens GN=ABCC2 PE=1 SV=3 - [MRP2_HUMAN] |
| Q14444 | Caprin-1 OS=Homo sapiens GN=CAPRIN1 PE=1 SV=2 - [CAPR1_HUMAN] |
| Q6IMN6 | Caprin-2 OS=Homo sapiens GN=CAPRIN2 PE=1 SV=1 - [CAPR2_HUMAN] |
| Q8N1G2 | Cap-specific mRNA (nucleoside-2'-O-)-methyltransferase 1 OS=Homo sapiens GN=CMTR1 PE=1 SV=1 - [CMTR1_HUMAN] |
| P31327 | Carbamoyl-phosphate synthase [ammonia], mitochondrial OS=Homo sapiens GN=CPS1 PE=1 SV=2 - [CPSM_HUMAN] |
| Q9NPF2 | Carbohydrate sulfotransferase 11 OS=Homo sapiens GN=CHST11 PE=1 SV=1 - [CHSTB_HUMAN] |
| Q8NCH0 | Carbohydrate sulfotransferase 14 OS=Homo sapiens GN=CHST14 PE=1 SV=2 - [CHSTE_HUMAN] |
| Q7LGC8 | Carbohydrate sulfotransferase 3 OS=Homo sapiens GN=CHST3 PE=1 SV=3 - [CHST3_HUMAN] |
| Q8NCG5 | Carbohydrate sulfotransferase 4 OS=Homo sapiens GN=CHST4 PE=1 SV=2 - [CHST4_HUMAN] |
| Q7L1S5 | Carbohydrate sulfotransferase 9 OS=Homo sapiens GN=CHST9 PE=2 SV=2 - [CHST9_HUMAN] |
| P00915 | Carbonic anhydrase 1 OS=Homo sapiens GN=CA1 PE=1 SV=2 - [CAH1_HUMAN] |
| P22748 | Carbonic anhydrase 4 OS=Homo sapiens GN=CA4 PE=1 SV=2 - [CAH4_HUMAN] |
| O75052 | Carboxyl-terminal PDZ ligand of neuronal nitric oxide synthase protein OS=Homo sapiens GN=NOS1AP PE=1 SV=3 - [CAPON_HUMAN] |
| P15086 | Carboxypeptidase B OS=Homo sapiens GN=CPB1 PE=1 SV=4 - [CBPB1_HUMAN] |
| O75976 | Carboxypeptidase D OS=Homo sapiens GN=CPD PE=1 SV=2 - [CBPD_HUMAN] |
| Q9Y646 | Carboxypeptidase Q OS=Homo sapiens GN=CPQ PE=1 SV=1 - [CBPQ_HUMAN] |
| P13688 | Carcinoembryonic antigen-related cell adhesion molecule 1 OS=Homo sapiens GN=CEACAM1 PE=1 SV=2 - [CEAM1_HUMAN] |
| Q8N3K9 | Cardiomyopathy-associated protein 5 OS=Homo sapiens GN=CMYA5 PE=1 SV=3 - [CMYA5_HUMAN] |
| Q8N4J0 | Carnosine N-methyltransferase OS=Homo sapiens GN=C9orf41 PE=1 SV=1 - [CARME_HUMAN] |
| A5YM72 | Carnosine synthase 1 OS=Homo sapiens GN=CARNS1 PE=1 SV=3 - [CRNS1_HUMAN] |
| P48730 | Casein kinase I isoform delta OS=Homo sapiens GN=CSNK1D PE=1 SV=2 - [KC1D_HUMAN] |
| Q5EG05 | Caspase recruitment domain-containing protein 16 OS=Homo sapiens GN=CARD16 PE=1 SV=1 - [CAR16_HUMAN] |
| Q9Y2G2 | Caspase recruitment domain-containing protein 8 OS=Homo sapiens GN=CARD8 PE=1 SV=1 - [CARD8_HUMAN] |
| P29466 | Caspase-1 OS=Homo sapiens GN=CASP1 PE=1 SV=1 - [CASP1_HUMAN] |
| Q14790 | Caspase-8 OS=Homo sapiens GN=CASP8 PE=1 SV=1 - [CASP8_HUMAN] |
| O60716 | Catenin delta-1 OS=Homo sapiens GN=CTNND1 PE=1 SV=1 - [CTND1_HUMAN] |
| Q9UQB3 | Catenin delta-2 OS=Homo sapiens GN=CTNND2 PE=1 SV=3 - [CTND2_HUMAN] |
| P07858 | Cathepsin B OS=Homo sapiens GN=CTSB PE=1 SV=3 - [CATB_HUMAN] |
| P08311 | Cathepsin G OS=Homo sapiens GN=CTSG PE=1 SV=2 - [CATG_HUMAN] |
| P07711 | Cathepsin L1 OS=Homo sapiens GN=CTSL PE=1 SV=2 - [CATL1_HUMAN] |
| Q9UBR2 | Cathepsin Z OS=Homo sapiens GN=CTSZ PE=1 SV=1 - [CATZ_HUMAN] |
| P20645 | Cation-dependent mannose-6-phosphate receptor OS=Homo sapiens GN=M6PR PE=1 SV=1 - [MPRD_HUMAN] |
| Q86WG3 | Caytaxin OS=Homo sapiens GN=ATCAY PE=1 SV=2 - [ATCAY_HUMAN] |
| P51684 | C-C chemokine receptor type 6 OS=Homo sapiens GN=CCR6 PE=2 SV=2 - [CCR6_HUMAN] |
| O15444 | C-C motif chemokine 25 OS=Homo sapiens GN=CCL25 PE=1 SV=2 - [CCL25_HUMAN] |
| P49716 | CCAAT/enhancer-binding protein delta OS=Homo sapiens GN=CEBPD PE=1 SV=2 - [CEBPD_HUMAN] |
| Q9H9A5 | CCR4-NOT transcription complex subunit 10 OS=Homo sapiens GN=CNOT10 PE=1 SV=1 - [CNO10_HUMAN] |
| Q01151 | CD83 antigen OS=Homo sapiens GN=CD83 PE=1 SV=1 - [CD83_HUMAN] |
| Q96SN8 | CDK5 regulatory subunit-associated protein 2 OS=Homo sapiens GN=CDK5RAP2 PE=1 SV=5 - [CK5P2_HUMAN] |
| Q8N126 | Cell adhesion molecule 3 OS=Homo sapiens GN=CADM3 PE=1 SV=1 - [CADM3_HUMAN] |
| O75943 | Cell cycle checkpoint protein RAD17 OS=Homo sapiens GN=RAD17 PE=1 SV=2 - [RAD17_HUMAN] |
| P30260 | Cell division cycle protein 27 homolog OS=Homo sapiens GN=CDC27 PE=1 SV=2 - [CDC27_HUMAN] |
| P29373 | Cellular retinoic acid-binding protein 2 OS=Homo sapiens GN=CRABP2 PE=1 SV=2 - [RABP2_HUMAN] |
| Q12798 | Centrin-1 OS=Homo sapiens GN=CETN1 PE=1 SV=1 - [CETN1_HUMAN] |
| Q7Z7A1 | Centriolin OS=Homo sapiens GN=CNTRL PE=1 SV=2 - [CNTRL_HUMAN] |
| P49454 | Centromere protein F OS=Homo sapiens GN=CENPF PE=1 SV=2 - [CENPF_HUMAN] |
| Q9H3R5 | Centromere protein H OS=Homo sapiens GN=CENPH PE=1 SV=1 - [CENPH_HUMAN] |
| Q96BT3 | Centromere protein T OS=Homo sapiens GN=CENPT PE=1 SV=2 - [CENPT_HUMAN] |
| Q71F23 | Centromere protein U OS=Homo sapiens GN=CENPU PE=1 SV=1 - [CENPU_HUMAN] |
| Q8N960 | Centrosomal protein of 120 kDa OS=Homo sapiens GN=CEP120 PE=1 SV=2 - [CE120_HUMAN] |
| Q66GS9 | Centrosomal protein of 135 kDa OS=Homo sapiens GN=CEP135 PE=1 SV=2 - [CP135_HUMAN] |
| O94986 | Centrosomal protein of 152 kDa OS=Homo sapiens GN=CEP152 PE=1 SV=4 - [CE152_HUMAN] |
| Q5TB80 | Centrosomal protein of 162 kDa OS=Homo sapiens GN=CEP162 PE=1 SV=2 - [CE162_HUMAN] |
| Q8TEP8 | Centrosomal protein of 192 kDa OS=Homo sapiens GN=CEP192 PE=1 SV=2 - [CE192_HUMAN] |
| Q9C0F1 | Centrosomal protein of 44 kDa OS=Homo sapiens GN=CEP44 PE=1 SV=2 - [CEP44_HUMAN] |
| Q86XR8 | Centrosomal protein of 57 kDa OS=Homo sapiens GN=CEP57 PE=1 SV=2 - [CEP57_HUMAN] |
| Q6P2H3 | Centrosomal protein of 85 kDa OS=Homo sapiens GN=CEP85 PE=1 SV=1 - [CEP85_HUMAN] |
| Q5VT06 | Centrosome-associated protein 350 OS=Homo sapiens GN=CEP350 PE=1 SV=1 - [CE350_HUMAN] |
| Q9BV73 | Centrosome-associated protein CEP250 OS=Homo sapiens GN=CEP250 PE=1 SV=2 - [CP250_HUMAN] |
| Q96MC4 | CEP295 N-terminal-like protein OS=Homo sapiens GN=CEP295NL PE=2 SV=1 - [C295L_HUMAN] |
| O95813 | Cerberus OS=Homo sapiens GN=CER1 PE=1 SV=1 - [CER1_HUMAN] |
| Q9NUG4 | Cerebral cavernous malformations 2 protein-like OS=Homo sapiens GN=CCM2L PE=2 SV=3 - [CCM2L_HUMAN] |
| Q9HD42 | Charged multivesicular body protein 1a OS=Homo sapiens GN=CHMP1A PE=1 SV=1 - [CHM1A_HUMAN] |
| Q8NHY5 | Checkpoint protein HUS1B OS=Homo sapiens GN=HUS1B PE=1 SV=2 - [HUS1B_HUMAN] |
| P51788 | Chloride channel protein 2 OS=Homo sapiens GN=CLCN2 PE=1 SV=2 - [CLCN2_HUMAN] |
| Q9Y6A2 | Cholesterol 24-hydroxylase OS=Homo sapiens GN=CYP46A1 PE=1 SV=1 - [CP46A_HUMAN] |
| Q8N4M1 | Choline transporter-like protein 3 OS=Homo sapiens GN=SLC44A3 PE=1 SV=4 - [CTL3_HUMAN] |
| Q8N6G5 | Chondroitin sulfate N-acetylgalactosaminyltransferase 2 OS=Homo sapiens GN=CSGALNACT2 PE=1 SV=1 - [CGAT2_HUMAN] |
| Q70JA7 | Chondroitin sulfate synthase 3 OS=Homo sapiens GN=CHSY3 PE=2 SV=3 - [CHSS3_HUMAN] |
| P01233 | Choriogonadotropin subunit beta OS=Homo sapiens GN=CGB PE=1 SV=1 - [CGHB_HUMAN] |
| P0DML2 | Chorionic somatomammotropin hormone 1 OS=Homo sapiens GN=CSH1 PE=1 SV=1 - [CSH1_HUMAN] |
| Q13111 | Chromatin assembly factor 1 subunit A OS=Homo sapiens GN=CHAF1A PE=1 SV=2 - [CAF1A_HUMAN] |
| O14646 | Chromodomain-helicase-DNA-binding protein 1 OS=Homo sapiens GN=CHD1 PE=1 SV=2 - [CHD1_HUMAN] |
| Q12873 | Chromodomain-helicase-DNA-binding protein 3 OS=Homo sapiens GN=CHD3 PE=1 SV=3 - [CHD3_HUMAN] |
| Q14839 | Chromodomain-helicase-DNA-binding protein 4 OS=Homo sapiens GN=CHD4 PE=1 SV=2 - [CHD4_HUMAN] |
| Q9P2D1 | Chromodomain-helicase-DNA-binding protein 7 OS=Homo sapiens GN=CHD7 PE=1 SV=3 - [CHD7_HUMAN] |
| Q3L8U1 | Chromodomain-helicase-DNA-binding protein 9 OS=Homo sapiens GN=CHD9 PE=1 SV=2 - [CHD9_HUMAN] |
| Q96JM3 | Chromosome alignment-maintaining phosphoprotein 1 OS=Homo sapiens GN=CHAMP1 PE=1 SV=2 - [CHAP1_HUMAN] |
| Q9P2M7 | Cingulin OS=Homo sapiens GN=CGN PE=1 SV=2 - [CING_HUMAN] |
| Q0VF96 | Cingulin-like protein 1 OS=Homo sapiens GN=CGNL1 PE=1 SV=2 - [CGNL1_HUMAN] |
| Q9ULV3 | Cip1-interacting zinc finger protein OS=Homo sapiens GN=CIZ1 PE=1 SV=2 - [CIZ1_HUMAN] |
| Q00610 | Clathrin heavy chain 1 OS=Homo sapiens GN=CLTC PE=1 SV=5 - [CLH1_HUMAN] |
| Q8NHS4 | Clathrin heavy chain linker domain-containing protein 1 OS=Homo sapiens GN=CLHC1 PE=1 SV=3 - [CLHC1_HUMAN] |
| Q8N7P3 | Claudin-22 OS=Homo sapiens GN=CLDN22 PE=2 SV=3 - [CLD22_HUMAN] |
| P56747 | Claudin-6 OS=Homo sapiens GN=CLDN6 PE=1 SV=2 - [CLD6_HUMAN] |
| Q10570 | Cleavage and polyadenylation specificity factor subunit 1 OS=Homo sapiens GN=CPSF1 PE=1 SV=2 - [CPSF1_HUMAN] |
| Q9UKF6 | Cleavage and polyadenylation specificity factor subunit 3 OS=Homo sapiens GN=CPSF3 PE=1 SV=1 - [CPSF3_HUMAN] |
| P33240 | Cleavage stimulation factor subunit 2 OS=Homo sapiens GN=CSTF2 PE=1 SV=1 - [CSTF2_HUMAN] |
| Q16842 | CMP-N-acetylneuraminate-beta-galactosamide-alpha-2,3-sialyltransferase 2 OS=Homo sapiens GN=ST3GAL2 PE=2 SV=1 - [SIA4B_HUMAN] |
| Q11206 | CMP-N-acetylneuraminate-beta-galactosamide-alpha-2,3-sialyltransferase 4 OS=Homo sapiens GN=ST3GAL4 PE=2 SV=1 - [SIA4C_HUMAN] |
| Q8TDQ1 | CMRF35-like molecule 1 OS=Homo sapiens GN=CD300LF PE=1 SV=3 - [CLM1_HUMAN] |
| Q496F6 | CMRF35-like molecule 2 OS=Homo sapiens GN=CD300E PE=1 SV=2 - [CLM2_HUMAN] |
| Q6UXZ3 | CMRF35-like molecule 4 OS=Homo sapiens GN=CD300LD PE=1 SV=1 - [CLM4_HUMAN] |
| Q6UXG3 | CMRF35-like molecule 9 OS=Homo sapiens GN=CD300LG PE=1 SV=2 - [CLM9_HUMAN] |
| Q8N9R6 | CMT1A duplicated region transcript 4 protein OS=Homo sapiens GN=CDRT4 PE=2 SV=2 - [CDRT4_HUMAN] |
| P12259 | Coagulation factor V OS=Homo sapiens GN=F5 PE=1 SV=4 - [FA5_HUMAN] |
| P00451 | Coagulation factor VIII OS=Homo sapiens GN=F8 PE=1 SV=1 - [FA8_HUMAN] |
| Q9Y678 | Coatomer subunit gamma-1 OS=Homo sapiens GN=COPG1 PE=1 SV=1 - [COPG1_HUMAN] |
| Q9UBF2 | Coatomer subunit gamma-2 OS=Homo sapiens GN=COPG2 PE=1 SV=1 - [COPG2_HUMAN] |
| O43405 | Cochlin OS=Homo sapiens GN=COCH PE=1 SV=1 - [COCH_HUMAN] |
| P23528 | Cofilin-1 OS=Homo sapiens GN=CFL1 PE=1 SV=3 - [COF1_HUMAN] |
| Q8N3U4 | Cohesin subunit SA-2 OS=Homo sapiens GN=STAG2 PE=1 SV=3 - [STAG2_HUMAN] |
| Q6P1N0 | Coiled-coil and C2 domain-containing protein 1A OS=Homo sapiens GN=CC2D1A PE=1 SV=1 - [C2D1A_HUMAN] |
| Q8IW40 | Coiled-coil domain-containing protein 103 OS=Homo sapiens GN=CCDC103 PE=1 SV=1 - [CC103_HUMAN] |
| Q6ZU64 | Coiled-coil domain-containing protein 108 OS=Homo sapiens GN=CCDC108 PE=1 SV=2 - [CC108_HUMAN] |
| Q8NEF3 | Coiled-coil domain-containing protein 112 OS=Homo sapiens GN=CCDC112 PE=1 SV=2 - [CC112_HUMAN] |
| Q8WUD4 | Coiled-coil domain-containing protein 12 OS=Homo sapiens GN=CCDC12 PE=1 SV=1 - [CCD12_HUMAN] |
| Q96HB5 | Coiled-coil domain-containing protein 120 OS=Homo sapiens GN=CCDC120 PE=1 SV=1 - [CC120_HUMAN] |
| Q96CT7 | Coiled-coil domain-containing protein 124 OS=Homo sapiens GN=CCDC124 PE=1 SV=1 - [CC124_HUMAN] |
| Q6PK04 | Coiled-coil domain-containing protein 137 OS=Homo sapiens GN=CCDC137 PE=1 SV=1 - [CC137_HUMAN] |
| Q49A88 | Coiled-coil domain-containing protein 14 OS=Homo sapiens GN=CCDC14 PE=1 SV=3 - [CCD14_HUMAN] |
| Q6ZP82 | Coiled-coil domain-containing protein 141 OS=Homo sapiens GN=CCDC141 PE=1 SV=2 - [CC141_HUMAN] |
| Q8NCX0 | Coiled-coil domain-containing protein 150 OS=Homo sapiens GN=CCDC150 PE=1 SV=2 - [CC150_HUMAN] |
| A6NI56 | Coiled-coil domain-containing protein 154 OS=Homo sapiens GN=CCDC154 PE=2 SV=4 - [CC154_HUMAN] |
| Q9P0B6 | Coiled-coil domain-containing protein 167 OS=Homo sapiens GN=CCDC167 PE=1 SV=2 - [CC167_HUMAN] |
| Q8NDH2 | Coiled-coil domain-containing protein 168 OS=Homo sapiens GN=CCDC168 PE=2 SV=2 - [CC168_HUMAN] |
| Q9P1Z9 | Coiled-coil domain-containing protein 180 OS=Homo sapiens GN=CCDC180 PE=2 SV=2 - [CC180_HUMAN] |
| Q5VVM6 | Coiled-coil domain-containing protein 30 OS=Homo sapiens GN=CCDC30 PE=2 SV=1 - [CCD30_HUMAN] |
| Q8IWA6 | Coiled-coil domain-containing protein 60 OS=Homo sapiens GN=CCDC60 PE=1 SV=2 - [CCD60_HUMAN] |
| Q8NA47 | Coiled-coil domain-containing protein 63 OS=Homo sapiens GN=CCDC63 PE=2 SV=1 - [CCD63_HUMAN] |
| Q8IXS2 | Coiled-coil domain-containing protein 65 OS=Homo sapiens GN=CCDC65 PE=1 SV=2 - [CCD65_HUMAN] |
| Q8IV32 | Coiled-coil domain-containing protein 71 OS=Homo sapiens GN=CCDC71 PE=2 SV=3 - [CCD71_HUMAN] |
| Q6ZRK6 | Coiled-coil domain-containing protein 73 OS=Homo sapiens GN=CCDC73 PE=2 SV=2 - [CCD73_HUMAN] |
| Q96AQ1 | Coiled-coil domain-containing protein 74A OS=Homo sapiens GN=CCDC74A PE=2 SV=1 - [CC74A_HUMAN] |
| Q76M96 | Coiled-coil domain-containing protein 80 OS=Homo sapiens GN=CCDC80 PE=1 SV=1 - [CCD80_HUMAN] |
| Q8N4S0 | Coiled-coil domain-containing protein 82 OS=Homo sapiens GN=CCDC82 PE=1 SV=2 - [CCD82_HUMAN] |
| A6NC98 | Coiled-coil domain-containing protein 88B OS=Homo sapiens GN=CCDC88B PE=1 SV=1 - [CC88B_HUMAN] |
| Q567U6 | Coiled-coil domain-containing protein 93 OS=Homo sapiens GN=CCDC93 PE=1 SV=2 - [CCD93_HUMAN] |
| A1A4V9 | Coiled-coil domain-containing protein C16orf93 OS=Homo sapiens GN=C16orf93 PE=2 SV=1 - [CP093_HUMAN] |
| Q8NCU4 | Coiled-coil domain-containing protein KIAA1407 OS=Homo sapiens GN=KIAA1407 PE=2 SV=1 - [K1407_HUMAN] |
| Q9Y534 | Cold shock domain-containing protein C2 OS=Homo sapiens GN=CSDC2 PE=1 SV=1 - [CSDC2_HUMAN] |
| P02452 | Collagen alpha-1(I) chain OS=Homo sapiens GN=COL1A1 PE=1 SV=5 - [CO1A1_HUMAN] |
| P20908 | Collagen alpha-1(V) chain OS=Homo sapiens GN=COL5A1 PE=1 SV=3 - [CO5A1_HUMAN] |
| Q02388 | Collagen alpha-1(VII) chain OS=Homo sapiens GN=COL7A1 PE=1 SV=2 - [CO7A1_HUMAN] |
| Q99715 | Collagen alpha-1(XII) chain OS=Homo sapiens GN=COL12A1 PE=1 SV=2 - [COCA1_HUMAN] |
| Q5TAT6 | Collagen alpha-1(XIII) chain OS=Homo sapiens GN=COL13A1 PE=1 SV=1 - [CODA1_HUMAN] |
| Q17RW2 | Collagen alpha-1(XXIV) chain OS=Homo sapiens GN=COL24A1 PE=1 SV=2 - [COOA1_HUMAN] |
| Q9BXS0 | Collagen alpha-1(XXV) chain OS=Homo sapiens GN=COL25A1 PE=1 SV=2 - [COPA1_HUMAN] |
| P12110 | Collagen alpha-2(VI) chain OS=Homo sapiens GN=COL6A2 PE=1 SV=4 - [CO6A2_HUMAN] |
| P25940 | Collagen alpha-3(V) chain OS=Homo sapiens GN=COL5A3 PE=1 SV=3 - [CO5A3_HUMAN] |
| P12111 | Collagen alpha-3(VI) chain OS=Homo sapiens GN=COL6A3 PE=1 SV=5 - [CO6A3_HUMAN] |
| P53420 | Collagen alpha-4(IV) chain OS=Homo sapiens GN=COL4A4 PE=1 SV=3 - [CO4A4_HUMAN] |
| A8TX70 | Collagen alpha-5(VI) chain OS=Homo sapiens GN=COL6A5 PE=1 SV=1 - [CO6A5_HUMAN] |
| Q9H0A8 | COMM domain-containing protein 4 OS=Homo sapiens GN=COMMD4 PE=1 SV=1 - [COMD4_HUMAN] |
| Q9BXJ2 | Complement C1q tumor necrosis factor-related protein 7 OS=Homo sapiens GN=C1QTNF7 PE=2 SV=1 - [C1QT7_HUMAN] |
| P60827 | Complement C1q tumor necrosis factor-related protein 8 OS=Homo sapiens GN=C1QTNF8 PE=1 SV=2 - [C1QT8_HUMAN] |
| P06681 | Complement C2 OS=Homo sapiens GN=C2 PE=1 SV=2 - [CO2_HUMAN] |
| P07357 | Complement component C8 alpha chain OS=Homo sapiens GN=C8A PE=1 SV=2 - [CO8A_HUMAN] |
| P08174 | Complement decay-accelerating factor OS=Homo sapiens GN=CD55 PE=1 SV=4 - [DAF_HUMAN] |
| P20023 | Complement receptor type 2 OS=Homo sapiens GN=CR2 PE=1 SV=2 - [CR2_HUMAN] |
| Q9BPX3 | Condensin complex subunit 3 OS=Homo sapiens GN=NCAPG PE=1 SV=1 - [CND3_HUMAN] |
| P83436 | Conserved oligomeric Golgi complex subunit 7 OS=Homo sapiens GN=COG7 PE=1 SV=1 - [COG7_HUMAN] |
| Q96MW5 | Conserved oligomeric Golgi complex subunit 8 OS=Homo sapiens GN=COG8 PE=1 SV=2 - [COG8_HUMAN] |
| O94779 | Contactin-5 OS=Homo sapiens GN=CNTN5 PE=1 SV=2 - [CNTN5_HUMAN] |
| Q9C0A0 | Contactin-associated protein-like 4 OS=Homo sapiens GN=CNTNAP4 PE=1 SV=3 - [CNTP4_HUMAN] |
| O75131 | Copine-3 OS=Homo sapiens GN=CPNE3 PE=1 SV=1 - [CPNE3_HUMAN] |
| Q96A23 | Copine-4 OS=Homo sapiens GN=CPNE4 PE=2 SV=1 - [CPNE4_HUMAN] |
| Q9UBL6 | Copine-7 OS=Homo sapiens GN=CPNE7 PE=1 SV=1 - [CPNE7_HUMAN] |
| Q53SF7 | Cordon-bleu protein-like 1 OS=Homo sapiens GN=COBLL1 PE=1 SV=2 - [COBL1_HUMAN] |
| P57737 | Coronin-7 OS=Homo sapiens GN=CORO7 PE=1 SV=2 - [CORO7_HUMAN] |
| Q7Z7K0 | COX assembly mitochondrial protein homolog OS=Homo sapiens GN=CMC1 PE=1 SV=1 - [COXM1_HUMAN] |
| Q8N123 | CPX chromosomal region candidate gene 1 protein OS=Homo sapiens GN=CPXCR1 PE=2 SV=2 - [CPXCR_HUMAN] |
| P12277 | Creatine kinase B-type OS=Homo sapiens GN=CKB PE=1 SV=1 - [KCRB_HUMAN] |
| Q8IUR6 | CREB3 regulatory factor OS=Homo sapiens GN=CREBRF PE=1 SV=2 - [CRERF_HUMAN] |
| Q5T3F8 | CSC1-like protein 2 OS=Homo sapiens GN=TMEM63B PE=1 SV=1 - [CSCL2_HUMAN] |
| P56545 | C-terminal-binding protein 2 OS=Homo sapiens GN=CTBP2 PE=1 SV=1 - [CTBP2_HUMAN] |
| Q9P126 | C-type lectin domain family 1 member B OS=Homo sapiens GN=CLEC1B PE=1 SV=2 - [CLC1B_HUMAN] |
| Q8N1N0 | C-type lectin domain family 4 member F OS=Homo sapiens GN=CLEC4F PE=2 SV=2 - [CLC4F_HUMAN] |
| Q9NY25 | C-type lectin domain family 5 member A OS=Homo sapiens GN=CLEC5A PE=1 SV=1 - [CLC5A_HUMAN] |
| Q6EIG7 | C-type lectin domain family 6 member A OS=Homo sapiens GN=CLEC6A PE=2 SV=1 - [CLC6A_HUMAN] |
| Q7Z408 | CUB and sushi domain-containing protein 2 OS=Homo sapiens GN=CSMD2 PE=1 SV=2 - [CSMD2_HUMAN] |
| Q13618 | Cullin-3 OS=Homo sapiens GN=CUL3 PE=1 SV=2 - [CUL3_HUMAN] |
| Q13619 | Cullin-4A OS=Homo sapiens GN=CUL4A PE=1 SV=3 - [CUL4A_HUMAN] |
| Q2TBE0 | CWF19-like protein 2 OS=Homo sapiens GN=CWF19L2 PE=1 SV=4 - [C19L2_HUMAN] |
| Q9P0U4 | CXXC-type zinc finger protein 1 OS=Homo sapiens GN=CXXC1 PE=1 SV=2 - [CXXC1_HUMAN] |
| Q8N884 | Cyclic GMP-AMP synthase OS=Homo sapiens GN=MB21D1 PE=1 SV=2 - [CGAS_HUMAN] |
| P24863 | Cyclin-C OS=Homo sapiens GN=CCNC PE=1 SV=2 - [CCNC_HUMAN] |
| Q14004 | Cyclin-dependent kinase 13 OS=Homo sapiens GN=CDK13 PE=1 SV=2 - [CDK13_HUMAN] |
| Q07002 | Cyclin-dependent kinase 18 OS=Homo sapiens GN=CDK18 PE=1 SV=3 - [CDK18_HUMAN] |
| P38936 | Cyclin-dependent kinase inhibitor 1 OS=Homo sapiens GN=CDKN1A PE=1 SV=3 - [CDN1A_HUMAN] |
| O76039 | Cyclin-dependent kinase-like 5 OS=Homo sapiens GN=CDKL5 PE=1 SV=1 - [CDKL5_HUMAN] |
| O14976 | Cyclin-G-associated kinase OS=Homo sapiens GN=GAK PE=1 SV=2 - [GAK_HUMAN] |
| Q8ND76 | Cyclin-Y OS=Homo sapiens GN=CCNY PE=1 SV=2 - [CCNY_HUMAN] |
| Q14093 | Cylicin-2 OS=Homo sapiens GN=CYLC2 PE=2 SV=1 - [CYLC2_HUMAN] |
| Q9NZV1 | Cysteine-rich motor neuron 1 protein OS=Homo sapiens GN=CRIM1 PE=1 SV=1 - [CRIM1_HUMAN] |
| P54108 | Cysteine-rich secretory protein 3 OS=Homo sapiens GN=CRISP3 PE=1 SV=1 - [CRIS3_HUMAN] |
| P13073 | Cytochrome c oxidase subunit 4 isoform 1, mitochondrial OS=Homo sapiens GN=COX4I1 PE=1 SV=1 - [COX41_HUMAN] |
| P24310 | Cytochrome c oxidase subunit 7A1, mitochondrial OS=Homo sapiens GN=COX7A1 PE=1 SV=2 - [CX7A1_HUMAN] |
| P53701 | Cytochrome c-type heme lyase OS=Homo sapiens GN=HCCS PE=1 SV=1 - [CCHL_HUMAN] |
| O43174 | Cytochrome P450 26A1 OS=Homo sapiens GN=CYP26A1 PE=2 SV=2 - [CP26A_HUMAN] |
| P10632 | Cytochrome P450 2C8 OS=Homo sapiens GN=CYP2C8 PE=1 SV=2 - [CP2C8_HUMAN] |
| P98187 | Cytochrome P450 4F8 OS=Homo sapiens GN=CYP4F8 PE=1 SV=1 - [CP4F8_HUMAN] |
| Q86W10 | Cytochrome P450 4Z1 OS=Homo sapiens GN=CYP4Z1 PE=2 SV=1 - [CP4Z1_HUMAN] |
| Q8WWM9 | Cytoglobin OS=Homo sapiens GN=CYGB PE=1 SV=1 - [CYGB_HUMAN] |
| Q15438 | Cytohesin-1 OS=Homo sapiens GN=CYTH1 PE=1 SV=1 - [CYH1_HUMAN] |
| Q14204 | Cytoplasmic dynein 1 heavy chain 1 OS=Homo sapiens GN=DYNC1H1 PE=1 SV=5 - [DYHC1_HUMAN] |
| Q8NCM8 | Cytoplasmic dynein 2 heavy chain 1 OS=Homo sapiens GN=DYNC2H1 PE=1 SV=4 - [DYHC2_HUMAN] |
| Q14008 | Cytoskeleton-associated protein 5 OS=Homo sapiens GN=CKAP5 PE=1 SV=3 - [CKAP5_HUMAN] |
| P28838 | Cytosol aminopeptidase OS=Homo sapiens GN=LAP3 PE=1 SV=3 - [AMPL_HUMAN] |
| O75891 | Cytosolic 10-formyltetrahydrofolate dehydrogenase OS=Homo sapiens GN=ALDH1L1 PE=1 SV=2 - [AL1L1_HUMAN] |
| Q96P26 | Cytosolic 5'-nucleotidase 1B OS=Homo sapiens GN=NT5C1B PE=2 SV=2 - [5NT1B_HUMAN] |
| Q9UPW5 | Cytosolic carboxypeptidase 1 OS=Homo sapiens GN=AGTPBP1 PE=1 SV=3 - [CBPC1_HUMAN] |
| P0C869 | Cytosolic phospholipase A2 beta OS=Homo sapiens GN=PLA2G4B PE=1 SV=2 - [PA24B_HUMAN] |
| Q68DD2 | Cytosolic phospholipase A2 zeta OS=Homo sapiens GN=PLA2G4F PE=2 SV=3 - [PA24F_HUMAN] |
| Q5M775 | Cytospin-B OS=Homo sapiens GN=SPECC1 PE=1 SV=1 - [CYTSB_HUMAN] |
| P21918 | D(1B) dopamine receptor OS=Homo sapiens GN=DRD5 PE=1 SV=2 - [DRD5_HUMAN] |
| Q8N465 | D-2-hydroxyglutarate dehydrogenase, mitochondrial OS=Homo sapiens GN=D2HGDH PE=1 SV=3 - [D2HDH_HUMAN] |
| Q9HCK1 | DBF4-type zinc finger-containing protein 2 OS=Homo sapiens GN=ZDBF2 PE=1 SV=3 - [ZDBF2_HUMAN] |
| Q8WV16 | DDB1- and CUL4-associated factor 4 OS=Homo sapiens GN=DCAF4 PE=1 SV=3 - [DCAF4_HUMAN] |
| Q9UER7 | Death domain-associated protein 6 OS=Homo sapiens GN=DAXX PE=1 SV=2 - [DAXX_HUMAN] |
| Q6ZMT9 | Death domain-containing protein 1 OS=Homo sapiens GN=DTHD1 PE=2 SV=3 - [DTHD1_HUMAN] |
| P53355 | Death-associated protein kinase 1 OS=Homo sapiens GN=DAPK1 PE=1 SV=6 - [DAPK1_HUMAN] |
| O43293 | Death-associated protein kinase 3 OS=Homo sapiens GN=DAPK3 PE=1 SV=1 - [DAPK3_HUMAN] |
| Q96BY6 | Dedicator of cytokinesis protein 10 OS=Homo sapiens GN=DOCK10 PE=1 SV=3 - [DOC10_HUMAN] |
| Q5JSL3 | Dedicator of cytokinesis protein 11 OS=Homo sapiens GN=DOCK11 PE=1 SV=2 - [DOC11_HUMAN] |
| Q8IZD9 | Dedicator of cytokinesis protein 3 OS=Homo sapiens GN=DOCK3 PE=1 SV=1 - [DOCK3_HUMAN] |
| Q96HP0 | Dedicator of cytokinesis protein 6 OS=Homo sapiens GN=DOCK6 PE=1 SV=3 - [DOCK6_HUMAN] |
| Q8NF50 | Dedicator of cytokinesis protein 8 OS=Homo sapiens GN=DOCK8 PE=1 SV=3 - [DOCK8_HUMAN] |
| Q9BZ29 | Dedicator of cytokinesis protein 9 OS=Homo sapiens GN=DOCK9 PE=1 SV=2 - [DOCK9_HUMAN] |
| Q9Y238 | Deleted in lung and esophageal cancer protein 1 OS=Homo sapiens GN=DLEC1 PE=2 SV=2 - [DLEC1_HUMAN] |
| Q9NR61 | Delta-like protein 4 OS=Homo sapiens GN=DLL4 PE=1 SV=1 - [DLL4_HUMAN] |
| Q08495 | Dematin OS=Homo sapiens GN=DMTN PE=1 SV=3 - [DEMA_HUMAN] |
| Q8TEH3 | DENN domain-containing protein 1A OS=Homo sapiens GN=DENND1A PE=1 SV=2 - [DEN1A_HUMAN] |
| Q6IQ26 | DENN domain-containing protein 5A OS=Homo sapiens GN=DENND5A PE=1 SV=2 - [DEN5A_HUMAN] |
| Q16854 | Deoxyguanosine kinase, mitochondrial OS=Homo sapiens GN=DGUOK PE=1 SV=2 - [DGUOK_HUMAN] |
| Q9H147 | Deoxynucleotidyltransferase terminal-interacting protein 1 OS=Homo sapiens GN=DNTTIP1 PE=1 SV=2 - [TDIF1_HUMAN] |
| P81605 | Dermcidin OS=Homo sapiens GN=DCD PE=1 SV=2 - [DCD_HUMAN] |
| P17661 | Desmin OS=Homo sapiens GN=DES PE=1 SV=3 - [DESM_HUMAN] |
| Q02487 | Desmocollin-2 OS=Homo sapiens GN=DSC2 PE=1 SV=1 - [DSC2_HUMAN] |
| Q14574 | Desmocollin-3 OS=Homo sapiens GN=DSC3 PE=1 SV=3 - [DSC3_HUMAN] |
| Q02413 | Desmoglein-1 OS=Homo sapiens GN=DSG1 PE=1 SV=2 - [DSG1_HUMAN] |
| Q16760 | Diacylglycerol kinase delta OS=Homo sapiens GN=DGKD PE=1 SV=4 - [DGKD_HUMAN] |
| P49619 | Diacylglycerol kinase gamma OS=Homo sapiens GN=DGKG PE=2 SV=3 - [DGKG_HUMAN] |
| P52824 | Diacylglycerol kinase theta OS=Homo sapiens GN=DGKQ PE=1 SV=2 - [DGKQ_HUMAN] |
| Q9UBU2 | Dickkopf-related protein 2 OS=Homo sapiens GN=DKK2 PE=2 SV=1 - [DKK2_HUMAN] |
| Q3MIW9 | Diffuse panbronchiolitis critical region protein 1 OS=Homo sapiens GN=DPCR1 PE=2 SV=2 - [DPCR1_HUMAN] |
| Q9BQC3 | Diphthamide biosynthesis protein 2 OS=Homo sapiens GN=DPH2 PE=1 SV=1 - [DPH2_HUMAN] |
| Q8TF46 | DIS3-like exonuclease 1 OS=Homo sapiens GN=DIS3L PE=1 SV=2 - [DI3L1_HUMAN] |
| Q16832 | Discoidin domain-containing receptor 2 OS=Homo sapiens GN=DDR2 PE=1 SV=2 - [DDR2_HUMAN] |
| Q14689 | Disco-interacting protein 2 homolog A OS=Homo sapiens GN=DIP2A PE=1 SV=2 - [DIP2A_HUMAN] |
| Q86T65 | Disheveled-associated activator of morphogenesis 2 OS=Homo sapiens GN=DAAM2 PE=1 SV=3 - [DAAM2_HUMAN] |
| P78536 | Disintegrin and metalloproteinase domain-containing protein 17 OS=Homo sapiens GN=ADAM17 PE=1 SV=1 - [ADA17_HUMAN] |
| Q9Y3Q7 | Disintegrin and metalloproteinase domain-containing protein 18 OS=Homo sapiens GN=ADAM18 PE=2 SV=1 - [ADA18_HUMAN] |
| Q9UKQ2 | Disintegrin and metalloproteinase domain-containing protein 28 OS=Homo sapiens GN=ADAM28 PE=2 SV=3 - [ADA28_HUMAN] |
| Q8TDM6 | Disks large homolog 5 OS=Homo sapiens GN=DLG5 PE=1 SV=4 - [DLG5_HUMAN] |
| O95886 | Disks large-associated protein 3 OS=Homo sapiens GN=DLGAP3 PE=1 SV=3 - [DLGP3_HUMAN] |
| Q9Y485 | DmX-like protein 1 OS=Homo sapiens GN=DMXL1 PE=1 SV=3 - [DMXL1_HUMAN] |
| Q8TDJ6 | DmX-like protein 2 OS=Homo sapiens GN=DMXL2 PE=1 SV=2 - [DMXL2_HUMAN] |
| Q9Y6K1 | DNA (cytosine-5)-methyltransferase 3A OS=Homo sapiens GN=DNMT3A PE=1 SV=4 - [DNM3A_HUMAN] |
| Q9UBC3 | DNA (cytosine-5)-methyltransferase 3B OS=Homo sapiens GN=DNMT3B PE=1 SV=1 - [DNM3B_HUMAN] |
| Q6PJP8 | DNA cross-link repair 1A protein OS=Homo sapiens GN=DCLRE1A PE=1 SV=3 - [DCR1A_HUMAN] |
| Q96D03 | DNA damage-inducible transcript 4-like protein OS=Homo sapiens GN=DDIT4L PE=1 SV=1 - [DDT4L_HUMAN] |
| Q9ULG1 | DNA helicase INO80 OS=Homo sapiens GN=INO80 PE=1 SV=2 - [INO80_HUMAN] |
| Q9NPF5 | DNA methyltransferase 1-associated protein 1 OS=Homo sapiens GN=DMAP1 PE=1 SV=1 - [DMAP1_HUMAN] |
| P40692 | DNA mismatch repair protein Mlh1 OS=Homo sapiens GN=MLH1 PE=1 SV=1 - [MLH1_HUMAN] |
| P43246 | DNA mismatch repair protein Msh2 OS=Homo sapiens GN=MSH2 PE=1 SV=1 - [MSH2_HUMAN] |
| Q15054 | DNA polymerase delta subunit 3 OS=Homo sapiens GN=POLD3 PE=1 SV=2 - [DPOD3_HUMAN] |
| Q07864 | DNA polymerase epsilon catalytic subunit A OS=Homo sapiens GN=POLE PE=1 SV=5 - [DPOE1_HUMAN] |
| Q9Y253 | DNA polymerase eta OS=Homo sapiens GN=POLH PE=1 SV=1 - [POLH_HUMAN] |
| Q9UBT6 | DNA polymerase kappa OS=Homo sapiens GN=POLK PE=1 SV=1 - [POLK_HUMAN] |
| P54098 | DNA polymerase subunit gamma-1 OS=Homo sapiens GN=POLG PE=1 SV=1 - [DPOG1_HUMAN] |
| O75417 | DNA polymerase theta OS=Homo sapiens GN=POLQ PE=1 SV=2 - [DPOLQ_HUMAN] |
| O60673 | DNA polymerase zeta catalytic subunit OS=Homo sapiens GN=REV3L PE=1 SV=2 - [DPOLZ_HUMAN] |
| Q92698 | DNA repair and recombination protein RAD54-like OS=Homo sapiens GN=RAD54L PE=1 SV=2 - [RAD54_HUMAN] |
| P23025 | DNA repair protein complementing XP-A cells OS=Homo sapiens GN=XPA PE=1 SV=1 - [XPA_HUMAN] |
| Q01831 | DNA repair protein complementing XP-C cells OS=Homo sapiens GN=XPC PE=1 SV=4 - [XPC_HUMAN] |
| Q92878 | DNA repair protein RAD50 OS=Homo sapiens GN=RAD50 PE=1 SV=1 - [RAD50_HUMAN] |
| Q9UBZ9 | DNA repair protein REV1 OS=Homo sapiens GN=REV1 PE=1 SV=1 - [REV1_HUMAN] |
| P51530 | DNA replication ATP-dependent helicase/nuclease DNA2 OS=Homo sapiens GN=DNA2 PE=1 SV=3 - [DNA2_HUMAN] |
| P33991 | DNA replication licensing factor MCM4 OS=Homo sapiens GN=MCM4 PE=1 SV=5 - [MCM4_HUMAN] |
| P33992 | DNA replication licensing factor MCM5 OS=Homo sapiens GN=MCM5 PE=1 SV=5 - [MCM5_HUMAN] |
| Q14566 | DNA replication licensing factor MCM6 OS=Homo sapiens GN=MCM6 PE=1 SV=1 - [MCM6_HUMAN] |
| P33993 | DNA replication licensing factor MCM7 OS=Homo sapiens GN=MCM7 PE=1 SV=4 - [MCM7_HUMAN] |
| Q02880 | DNA topoisomerase 2-beta OS=Homo sapiens GN=TOP2B PE=1 SV=3 - [TOP2B_HUMAN] |
| Q9H5L6 | DNA transposase THAP9 OS=Homo sapiens GN=THAP9 PE=1 SV=2 - [THAP9_HUMAN] |
| Q2KHR2 | DNA-binding protein RFX7 OS=Homo sapiens GN=RFX7 PE=1 SV=1 - [RFX7_HUMAN] |
| Q01826 | DNA-binding protein SATB1 OS=Homo sapiens GN=SATB1 PE=1 SV=1 - [SATB1_HUMAN] |
| P78527 | DNA-dependent protein kinase catalytic subunit OS=Homo sapiens GN=PRKDC PE=1 SV=3 - [PRKDC_HUMAN] |
| Q9NP87 | DNA-directed DNA/RNA polymerase mu OS=Homo sapiens GN=POLM PE=1 SV=1 - [DPOLM_HUMAN] |
| O95602 | DNA-directed RNA polymerase I subunit RPA1 OS=Homo sapiens GN=POLR1A PE=1 SV=2 - [RPA1_HUMAN] |
| O14802 | DNA-directed RNA polymerase III subunit RPC1 OS=Homo sapiens GN=POLR3A PE=1 SV=2 - [RPC1_HUMAN] |
| O00411 | DNA-directed RNA polymerase, mitochondrial OS=Homo sapiens GN=POLRMT PE=1 SV=2 - [RPOM_HUMAN] |
| P19388 | DNA-directed RNA polymerases I, II, and III subunit RPABC1 OS=Homo sapiens GN=POLR2E PE=1 SV=4 - [RPAB1_HUMAN] |
| P59910 | DnaJ homolog subfamily B member 13 OS=Homo sapiens GN=DNAJB13 PE=2 SV=1 - [DJB13_HUMAN] |
| Q9NVH1 | DnaJ homolog subfamily C member 11 OS=Homo sapiens GN=DNAJC11 PE=1 SV=2 - [DJC11_HUMAN] |
| Q9NNZ3 | DnaJ homolog subfamily C member 4 OS=Homo sapiens GN=DNAJC4 PE=1 SV=1 - [DNJC4_HUMAN] |
| O60496 | Docking protein 2 OS=Homo sapiens GN=DOK2 PE=1 SV=2 - [DOK2_HUMAN] |
| Q6PKX4 | Docking protein 6 OS=Homo sapiens GN=DOK6 PE=1 SV=1 - [DOK6_HUMAN] |
| O60762 | Dolichol-phosphate mannosyltransferase subunit 1 OS=Homo sapiens GN=DPM1 PE=1 SV=1 - [DPM1_HUMAN] |
| Q9Y672 | Dolichyl pyrophosphate Man9GlcNAc2 alpha-1,3-glucosyltransferase OS=Homo sapiens GN=ALG6 PE=1 SV=1 - [ALG6_HUMAN] |
| P46977 | Dolichyl-diphosphooligosaccharide--protein glycosyltransferase subunit STT3A OS=Homo sapiens GN=STT3A PE=1 SV=2 - [STT3A_HUMAN] |
| Q92685 | Dol-P-Man:Man(5)GlcNAc(2)-PP-Dol alpha-1,3-mannosyltransferase OS=Homo sapiens GN=ALG3 PE=1 SV=1 - [ALG3_HUMAN] |
| Q6RFH8 | Double homeobox protein 4C OS=Homo sapiens GN=DUX4L9 PE=1 SV=1 - [DUX4C_HUMAN] |
| P49959 | Double-strand break repair protein MRE11A OS=Homo sapiens GN=MRE11A PE=1 SV=3 - [MRE11_HUMAN] |
| Q9NUL3 | Double-stranded RNA-binding protein Staufen homolog 2 OS=Homo sapiens GN=STAU2 PE=1 SV=1 - [STAU2_HUMAN] |
| O60469 | Down syndrome cell adhesion molecule OS=Homo sapiens GN=DSCAM PE=1 SV=2 - [DSCAM_HUMAN] |
| Q9UJU6 | Drebrin-like protein OS=Homo sapiens GN=DBNL PE=1 SV=1 - [DBNL_HUMAN] |
| P45985 | Dual specificity mitogen-activated protein kinase kinase 4 OS=Homo sapiens GN=MAP2K4 PE=1 SV=1 - [MP2K4_HUMAN] |
| Q13163 | Dual specificity mitogen-activated protein kinase kinase 5 OS=Homo sapiens GN=MAP2K5 PE=1 SV=2 - [MP2K5_HUMAN] |
| O14733 | Dual specificity mitogen-activated protein kinase kinase 7 OS=Homo sapiens GN=MAP2K7 PE=1 SV=2 - [MP2K7_HUMAN] |
| Q9Y6W6 | Dual specificity protein phosphatase 10 OS=Homo sapiens GN=DUSP10 PE=1 SV=1 - [DUS10_HUMAN] |
| Q6B8I1 | Dual specificity protein phosphatase 13 isoform A OS=Homo sapiens GN=DUSP13 PE=1 SV=1 - [DS13A_HUMAN] |
| Q9UII6 | Dual specificity protein phosphatase 13 isoform B OS=Homo sapiens GN=DUSP13 PE=1 SV=3 - [DS13B_HUMAN] |
| Q9H596 | Dual specificity protein phosphatase 21 OS=Homo sapiens GN=DUSP21 PE=1 SV=1 - [DUS21_HUMAN] |
| Q9UNH5 | Dual specificity protein phosphatase CDC14A OS=Homo sapiens GN=CDC14A PE=1 SV=1 - [CC14A_HUMAN] |
| Q96S53 | Dual specificity testis-specific protein kinase 2 OS=Homo sapiens GN=TESK2 PE=1 SV=1 - [TESK2_HUMAN] |
| Q6XZF7 | Dynamin-binding protein OS=Homo sapiens GN=DNMBP PE=1 SV=1 - [DNMBP_HUMAN] |
| O60313 | Dynamin-like 120 kDa protein, mitochondrial OS=Homo sapiens GN=OPA1 PE=1 SV=3 - [OPA1_HUMAN] |
| Q8IVF4 | Dynein heavy chain 10, axonemal OS=Homo sapiens GN=DNAH10 PE=1 SV=4 - [DYH10_HUMAN] |
| Q96DT5 | Dynein heavy chain 11, axonemal OS=Homo sapiens GN=DNAH11 PE=1 SV=4 - [DYH11_HUMAN] |
| Q9UFH2 | Dynein heavy chain 17, axonemal OS=Homo sapiens GN=DNAH17 PE=1 SV=2 - [DYH17_HUMAN] |
| Q8TD57 | Dynein heavy chain 3, axonemal OS=Homo sapiens GN=DNAH3 PE=2 SV=1 - [DYH3_HUMAN] |
| Q8TE73 | Dynein heavy chain 5, axonemal OS=Homo sapiens GN=DNAH5 PE=1 SV=3 - [DYH5_HUMAN] |
| Q8WXX0 | Dynein heavy chain 7, axonemal OS=Homo sapiens GN=DNAH7 PE=1 SV=2 - [DYH7_HUMAN] |
| Q9NYC9 | Dynein heavy chain 9, axonemal OS=Homo sapiens GN=DNAH9 PE=1 SV=3 - [DYH9_HUMAN] |
| Q96M86 | Dynein heavy chain domain-containing protein 1 OS=Homo sapiens GN=DNHD1 PE=2 SV=2 - [DNHD1_HUMAN] |
| Q5VV43 | Dyslexia-associated protein KIAA0319 OS=Homo sapiens GN=KIAA0319 PE=1 SV=1 - [K0319_HUMAN] |
| Q03001 | Dystonin OS=Homo sapiens GN=DST PE=1 SV=4 - [DYST_HUMAN] |
| P11532 | Dystrophin OS=Homo sapiens GN=DMD PE=1 SV=3 - [DMD_HUMAN] |
| O00257 | E3 SUMO-protein ligase CBX4 OS=Homo sapiens GN=CBX4 PE=1 SV=3 - [CBX4_HUMAN] |
| O75150 | E3 ubiquitin-protein ligase BRE1B OS=Homo sapiens GN=RNF40 PE=1 SV=4 - [BRE1B_HUMAN] |
| Q13191 | E3 ubiquitin-protein ligase CBL-B OS=Homo sapiens GN=CBLB PE=1 SV=2 - [CBLB_HUMAN] |
| Q9UNE7 | E3 ubiquitin-protein ligase CHIP OS=Homo sapiens GN=STUB1 PE=1 SV=2 - [CHIP_HUMAN] |
| Q9Y2E6 | E3 ubiquitin-protein ligase DTX4 OS=Homo sapiens GN=DTX4 PE=1 SV=2 - [DTX4_HUMAN] |
| Q9ULT8 | E3 ubiquitin-protein ligase HECTD1 OS=Homo sapiens GN=HECTD1 PE=1 SV=3 - [HECD1_HUMAN] |
| O95714 | E3 ubiquitin-protein ligase HERC2 OS=Homo sapiens GN=HERC2 PE=1 SV=2 - [HERC2_HUMAN] |
| Q7Z6Z7 | E3 ubiquitin-protein ligase HUWE1 OS=Homo sapiens GN=HUWE1 PE=1 SV=3 - [HUWE1_HUMAN] |
| O94822 | E3 ubiquitin-protein ligase listerin OS=Homo sapiens GN=LTN1 PE=1 SV=6 - [LTN1_HUMAN] |
| Q8TBB1 | E3 ubiquitin-protein ligase LNX OS=Homo sapiens GN=LNX1 PE=1 SV=1 - [LNX1_HUMAN] |
| Q6UWE0 | E3 ubiquitin-protein ligase LRSAM1 OS=Homo sapiens GN=LRSAM1 PE=1 SV=1 - [LRSM1_HUMAN] |
| Q9UHC7 | E3 ubiquitin-protein ligase makorin-1 OS=Homo sapiens GN=MKRN1 PE=1 SV=3 - [MKRN1_HUMAN] |
| O75592 | E3 ubiquitin-protein ligase MYCBP2 OS=Homo sapiens GN=MYCBP2 PE=1 SV=3 - [MYCB2_HUMAN] |
| P46934 | E3 ubiquitin-protein ligase NEDD4 OS=Homo sapiens GN=NEDD4 PE=1 SV=4 - [NEDD4_HUMAN] |
| Q8NG27 | E3 ubiquitin-protein ligase Praja-1 OS=Homo sapiens GN=PJA1 PE=1 SV=2 - [PJA1_HUMAN] |
| Q7Z6E9 | E3 ubiquitin-protein ligase RBBP6 OS=Homo sapiens GN=RBBP6 PE=1 SV=1 - [RBBP6_HUMAN] |
| Q86XS8 | E3 ubiquitin-protein ligase RNF130 OS=Homo sapiens GN=RNF130 PE=1 SV=1 - [GOLI_HUMAN] |
| Q8NCN4 | E3 ubiquitin-protein ligase RNF169 OS=Homo sapiens GN=RNF169 PE=1 SV=2 - [RN169_HUMAN] |
| Q63HN8 | E3 ubiquitin-protein ligase RNF213 OS=Homo sapiens GN=RNF213 PE=1 SV=3 - [RN213_HUMAN] |
| Q96EP0 | E3 ubiquitin-protein ligase RNF31 OS=Homo sapiens GN=RNF31 PE=1 SV=1 - [RNF31_HUMAN] |
| Q9Y252 | E3 ubiquitin-protein ligase RNF6 OS=Homo sapiens GN=RNF6 PE=1 SV=1 - [RNF6_HUMAN] |
| Q9NS56 | E3 ubiquitin-protein ligase Topors OS=Homo sapiens GN=TOPORS PE=1 SV=1 - [TOPRS_HUMAN] |
| Q9BZY9 | E3 ubiquitin-protein ligase TRIM31 OS=Homo sapiens GN=TRIM31 PE=1 SV=2 - [TRI31_HUMAN] |
| Q13049 | E3 ubiquitin-protein ligase TRIM32 OS=Homo sapiens GN=TRIM32 PE=1 SV=2 - [TRI32_HUMAN] |
| Q8WV44 | E3 ubiquitin-protein ligase TRIM41 OS=Homo sapiens GN=TRIM41 PE=1 SV=3 - [TRI41_HUMAN] |
| Q9BRZ2 | E3 ubiquitin-protein ligase TRIM56 OS=Homo sapiens GN=TRIM56 PE=1 SV=3 - [TRI56_HUMAN] |
| Q6AZZ1 | E3 ubiquitin-protein ligase TRIM68 OS=Homo sapiens GN=TRIM68 PE=1 SV=1 - [TRI68_HUMAN] |
| Q14669 | E3 ubiquitin-protein ligase TRIP12 OS=Homo sapiens GN=TRIP12 PE=1 SV=1 - [TRIPC_HUMAN] |
| Q8IWV7 | E3 ubiquitin-protein ligase UBR1 OS=Homo sapiens GN=UBR1 PE=1 SV=1 - [UBR1_HUMAN] |
| Q6ZT12 | E3 ubiquitin-protein ligase UBR3 OS=Homo sapiens GN=UBR3 PE=2 SV=2 - [UBR3_HUMAN] |
| Q9HC35 | Echinoderm microtubule-associated protein-like 4 OS=Homo sapiens GN=EML4 PE=1 SV=3 - [EMAL4_HUMAN] |
| Q9Y5L3 | Ectonucleoside triphosphate diphosphohydrolase 2 OS=Homo sapiens GN=ENTPD2 PE=1 SV=1 - [ENTP2_HUMAN] |
| Q9Y227 | Ectonucleoside triphosphate diphosphohydrolase 4 OS=Homo sapiens GN=ENTPD4 PE=1 SV=1 - [ENTP4_HUMAN] |
| O14638 | Ectonucleotide pyrophosphatase/phosphodiesterase family member 3 OS=Homo sapiens GN=ENPP3 PE=1 SV=2 - [ENPP3_HUMAN] |
| Q9HCE0 | Ectopic P granules protein 5 homolog OS=Homo sapiens GN=EPG5 PE=2 SV=2 - [EPG5_HUMAN] |
| Q9HA90 | EF-hand and coiled-coil domain-containing protein 1 OS=Homo sapiens GN=EFCC1 PE=2 SV=2 - [EFCC1_HUMAN] |
| Q8IY85 | EF-hand calcium-binding domain-containing protein 13 OS=Homo sapiens GN=EFCAB13 PE=2 SV=2 - [EFC13_HUMAN] |
| A4FU69 | EF-hand calcium-binding domain-containing protein 5 OS=Homo sapiens GN=EFCAB5 PE=1 SV=3 - [EFCB5_HUMAN] |
| Q5JVL4 | EF-hand domain-containing protein 1 OS=Homo sapiens GN=EFHC1 PE=1 SV=1 - [EFHC1_HUMAN] |
| Q8N3D4 | EH domain-binding protein 1-like protein 1 OS=Homo sapiens GN=EHBP1L1 PE=1 SV=2 - [EH1L1_HUMAN] |
| Q9BY07 | Electrogenic sodium bicarbonate cotransporter 4 OS=Homo sapiens GN=SLC4A5 PE=2 SV=2 - [S4A5_HUMAN] |
| P57679 | Ellis-van Creveld syndrome protein OS=Homo sapiens GN=EVC PE=1 SV=1 - [EVC_HUMAN] |
| Q6PJG2 | ELM2 and SANT domain-containing protein 1 OS=Homo sapiens GN=ELMSAN1 PE=1 SV=2 - [EMSA1_HUMAN] |
| Q96FG2 | ELMO domain-containing protein 3 OS=Homo sapiens GN=ELMOD3 PE=1 SV=2 - [ELMD3_HUMAN] |
| P13639 | Elongation factor 2 OS=Homo sapiens GN=EEF2 PE=1 SV=4 - [EF2_HUMAN] |
| Q9Y6C2 | EMILIN-1 OS=Homo sapiens GN=EMILIN1 PE=1 SV=2 - [EMIL1_HUMAN] |
| Q9YNA8 | Endogenous retrovirus group K member 19 Gag polyprotein OS=Homo sapiens GN=ERVK-19 PE=1 SV=3 - [GAK19_HUMAN] |
| P61566 | Endogenous retrovirus group K member 24 Env polyprotein OS=Homo sapiens GN=ERVK-24 PE=2 SV=1 - [ENK24_HUMAN] |
| Q7LDI9 | Endogenous retrovirus group K member 6 Gag polyprotein OS=Homo sapiens GN=ERVK-6 PE=1 SV=3 - [GAK6_HUMAN] |
| P63135 | Endogenous retrovirus group K member 7 Pol protein OS=Homo sapiens GN=ERVK-7 PE=3 SV=1 - [POK7_HUMAN] |
| Q9UKH3 | Endogenous retrovirus group K member 9 Env polyprotein OS=Homo sapiens GN=ERVK-9 PE=1 SV=1 - [ENK9_HUMAN] |
| P61550 | Endogenous retrovirus group S71 member 1 Env polyprotein OS=Homo sapiens GN=ERVS71-1 PE=2 SV=1 - [ENVT1_HUMAN] |
| Q96FI4 | Endonuclease 8-like 1 OS=Homo sapiens GN=NEIL1 PE=1 SV=3 - [NEIL1_HUMAN] |
| Q14249 | Endonuclease G, mitochondrial OS=Homo sapiens GN=ENDOG PE=1 SV=4 - [NUCG_HUMAN] |
| Q99961 | Endophilin-A2 OS=Homo sapiens GN=SH3GL1 PE=1 SV=1 - [SH3G1_HUMAN] |
| Q6P179 | Endoplasmic reticulum aminopeptidase 2 OS=Homo sapiens GN=ERAP2 PE=1 SV=2 - [ERAP2_HUMAN] |
| Q9UPY3 | Endoribonuclease Dicer OS=Homo sapiens GN=DICER1 PE=1 SV=3 - [DICER_HUMAN] |
| P24530 | Endothelin B receptor OS=Homo sapiens GN=EDNRB PE=1 SV=1 - [EDNRB_HUMAN] |
| P42892 | Endothelin-converting enzyme 1 OS=Homo sapiens GN=ECE1 PE=1 SV=2 - [ECE1_HUMAN] |
| Q96JJ3 | Engulfment and cell motility protein 2 OS=Homo sapiens GN=ELMO2 PE=1 SV=2 - [ELMO2_HUMAN] |
| Q96BJ8 | Engulfment and cell motility protein 3 OS=Homo sapiens GN=ELMO3 PE=2 SV=3 - [ELMO3_HUMAN] |
| Q6P2E9 | Enhancer of mRNA-decapping protein 4 OS=Homo sapiens GN=EDC4 PE=1 SV=1 - [EDC4_HUMAN] |
| P42126 | Enoyl-CoA delta isomerase 1, mitochondrial OS=Homo sapiens GN=ECI1 PE=1 SV=1 - [ECI1_HUMAN] |
| Q96DC8 | Enoyl-CoA hydratase domain-containing protein 3, mitochondrial OS=Homo sapiens GN=ECHDC3 PE=1 SV=2 - [ECHD3_HUMAN] |
| Q92817 | Envoplakin OS=Homo sapiens GN=EVPL PE=1 SV=3 - [EVPL_HUMAN] |
| P12724 | Eosinophil cationic protein OS=Homo sapiens GN=RNASE3 PE=1 SV=2 - [ECP_HUMAN] |
| P11678 | Eosinophil peroxidase OS=Homo sapiens GN=EPX PE=1 SV=2 - [PERE_HUMAN] |
| P29323 | Ephrin type-B receptor 2 OS=Homo sapiens GN=EPHB2 PE=1 SV=5 - [EPHB2_HUMAN] |
| P20827 | Ephrin-A1 OS=Homo sapiens GN=EFNA1 PE=1 SV=2 - [EFNA1_HUMAN] |
| Q8TE67 | Epidermal growth factor receptor kinase substrate 8-like protein 3 OS=Homo sapiens GN=EPS8L3 PE=1 SV=2 - [ES8L3_HUMAN] |
| Q9UHF1 | Epidermal growth factor-like protein 7 OS=Homo sapiens GN=EGFL7 PE=1 SV=3 - [EGFL7_HUMAN] |
| Q99645 | Epiphycan OS=Homo sapiens GN=EPYC PE=2 SV=3 - [EPYC_HUMAN] |
| P58107 | Epiplakin OS=Homo sapiens GN=EPPK1 PE=1 SV=2 - [EPIPL_HUMAN] |
| Q6NXG1 | Epithelial splicing regulatory protein 1 OS=Homo sapiens GN=ESRP1 PE=1 SV=2 - [ESRP1_HUMAN] |
| O95925 | Eppin OS=Homo sapiens GN=EPPIN PE=1 SV=1 - [EPPI_HUMAN] |
| Q9BV94 | ER degradation-enhancing alpha-mannosidase-like protein 2 OS=Homo sapiens GN=EDEM2 PE=1 SV=2 - [EDEM2_HUMAN] |
| Q8N766 | ER membrane protein complex subunit 1 OS=Homo sapiens GN=EMC1 PE=1 SV=1 - [EMC1_HUMAN] |
| Q5UCC4 | ER membrane protein complex subunit 10 OS=Homo sapiens GN=EMC10 PE=1 SV=1 - [EMC10_HUMAN] |
| Q9H501 | ESF1 homolog OS=Homo sapiens GN=ESF1 PE=1 SV=1 - [ESF1_HUMAN] |
| Q6ZN32 | ETS translocation variant 3-like protein OS=Homo sapiens GN=ETV3L PE=2 SV=1 - [ETV3L_HUMAN] |
| O00418 | Eukaryotic elongation factor 2 kinase OS=Homo sapiens GN=EEF2K PE=1 SV=2 - [EF2K_HUMAN] |
| P15170 | Eukaryotic peptide chain release factor GTP-binding subunit ERF3A OS=Homo sapiens GN=GSPT1 PE=1 SV=1 - [ERF3A_HUMAN] |
| P62495 | Eukaryotic peptide chain release factor subunit 1 OS=Homo sapiens GN=ETF1 PE=1 SV=3 - [ERF1_HUMAN] |
| Q9NZJ5 | Eukaryotic translation initiation factor 2-alpha kinase 3 OS=Homo sapiens GN=EIF2AK3 PE=1 SV=3 - [E2AK3_HUMAN] |
| P41214 | Eukaryotic translation initiation factor 2D OS=Homo sapiens GN=EIF2D PE=1 SV=3 - [EIF2D_HUMAN] |
| O75822 | Eukaryotic translation initiation factor 3 subunit J OS=Homo sapiens GN=EIF3J PE=1 SV=2 - [EIF3J_HUMAN] |
| Q04637 | Eukaryotic translation initiation factor 4 gamma 1 OS=Homo sapiens GN=EIF4G1 PE=1 SV=4 - [IF4G1_HUMAN] |
| P78344 | Eukaryotic translation initiation factor 4 gamma 2 OS=Homo sapiens GN=EIF4G2 PE=1 SV=1 - [IF4G2_HUMAN] |
| Q9NRA8 | Eukaryotic translation initiation factor 4E transporter OS=Homo sapiens GN=EIF4ENIF1 PE=1 SV=2 - [4ET_HUMAN] |
| Q15056 | Eukaryotic translation initiation factor 4H OS=Homo sapiens GN=EIF4H PE=1 SV=5 - [IF4H_HUMAN] |
| Q9GZV4 | Eukaryotic translation initiation factor 5A-2 OS=Homo sapiens GN=EIF5A2 PE=1 SV=3 - [IF5A2_HUMAN] |
| P43004 | Excitatory amino acid transporter 2 OS=Homo sapiens GN=SLC1A2 PE=1 SV=2 - [EAA2_HUMAN] |
| O00471 | Exocyst complex component 5 OS=Homo sapiens GN=EXOC5 PE=1 SV=1 - [EXOC5_HUMAN] |
| Q9UQ84 | Exonuclease 1 OS=Homo sapiens GN=EXO1 PE=1 SV=2 - [EXO1_HUMAN] |
| Q9NVH0 | Exonuclease 3'-5' domain-containing protein 2 OS=Homo sapiens GN=EXD2 PE=1 SV=2 - [EXD2_HUMAN] |
| Q8NEV8 | Exophilin-5 OS=Homo sapiens GN=EXPH5 PE=1 SV=3 - [EXPH5_HUMAN] |
| Q9NQT4 | Exosome complex component RRP46 OS=Homo sapiens GN=EXOSC5 PE=1 SV=1 - [EXOS5_HUMAN] |
| Q01780 | Exosome component 10 OS=Homo sapiens GN=EXOSC10 PE=1 SV=2 - [EXOSX_HUMAN] |
| P55060 | Exportin-2 OS=Homo sapiens GN=CSE1L PE=1 SV=3 - [XPO2_HUMAN] |
| Q96QU8 | Exportin-6 OS=Homo sapiens GN=XPO6 PE=1 SV=1 - [XPO6_HUMAN] |
| A0FGR9 | Extended synaptotagmin-3 OS=Homo sapiens GN=ESYT3 PE=1 SV=1 - [ESYT3_HUMAN] |
| P41180 | Extracellular calcium-sensing receptor OS=Homo sapiens GN=CASR PE=1 SV=2 - [CASR_HUMAN] |
| Q86XX4 | Extracellular matrix protein FRAS1 OS=Homo sapiens GN=FRAS1 PE=1 SV=2 - [FRAS1_HUMAN] |
| Q8IWU6 | Extracellular sulfatase Sulf-1 OS=Homo sapiens GN=SULF1 PE=1 SV=1 - [SULF1_HUMAN] |
| Q6QHK4 | Factor in the germline alpha OS=Homo sapiens GN=FIGLA PE=1 SV=2 - [FIGLA_HUMAN] |
| Q96CU9 | FAD-dependent oxidoreductase domain-containing protein 1 OS=Homo sapiens GN=FOXRED1 PE=1 SV=2 - [FXRD1_HUMAN] |
| O15360 | Fanconi anemia group A protein OS=Homo sapiens GN=FANCA PE=1 SV=2 - [FANCA_HUMAN] |
| Q9BXW9 | Fanconi anemia group D2 protein OS=Homo sapiens GN=FANCD2 PE=1 SV=2 - [FACD2_HUMAN] |
| Q8IYD8 | Fanconi anemia group M protein OS=Homo sapiens GN=FANCM PE=1 SV=2 - [FANCM_HUMAN] |
| Q53R41 | FAST kinase domain-containing protein 1 OS=Homo sapiens GN=FASTKD1 PE=1 SV=1 - [FAKD1_HUMAN] |
| Q14CZ7 | FAST kinase domain-containing protein 3 OS=Homo sapiens GN=FASTKD3 PE=1 SV=2 - [FAKD3_HUMAN] |
| Q8NCQ5 | F-box only protein 15 OS=Homo sapiens GN=FBXO15 PE=2 SV=2 - [FBX15_HUMAN] |
| Q6PIJ6 | F-box only protein 38 OS=Homo sapiens GN=FBXO38 PE=1 SV=3 - [FBX38_HUMAN] |
| Q9NRD1 | F-box only protein 6 OS=Homo sapiens GN=FBXO6 PE=1 SV=1 - [FBX6_HUMAN] |
| Q8N1E6 | F-box/LRR-repeat protein 14 OS=Homo sapiens GN=FBXL14 PE=1 SV=1 - [FXL14_HUMAN] |
| Q9UJT9 | F-box/LRR-repeat protein 7 OS=Homo sapiens GN=FBXL7 PE=2 SV=1 - [FBXL7_HUMAN] |
| Q9Y297 | F-box/WD repeat-containing protein 1A OS=Homo sapiens GN=BTRC PE=1 SV=1 - [FBW1A_HUMAN] |
| Q96RD9 | Fc receptor-like protein 5 OS=Homo sapiens GN=FCRL5 PE=1 SV=3 - [FCRL5_HUMAN] |
| A0AVI2 | Fer-1-like protein 5 OS=Homo sapiens GN=FER1L5 PE=2 SV=2 - [FR1L5_HUMAN] |
| Q2WGJ9 | Fer-1-like protein 6 OS=Homo sapiens GN=FER1L6 PE=2 SV=2 - [FR1L6_HUMAN] |
| Q68DX3 | FERM and PDZ domain-containing protein 2 OS=Homo sapiens GN=FRMPD2 PE=1 SV=3 - [FRPD2_HUMAN] |
| Q5JV73 | FERM and PDZ domain-containing protein 3 OS=Homo sapiens GN=FRMPD3 PE=2 SV=2 - [FRPD3_HUMAN] |
| Q14CM0 | FERM and PDZ domain-containing protein 4 OS=Homo sapiens GN=FRMPD4 PE=1 SV=1 - [FRPD4_HUMAN] |
| Q8N878 | FERM domain-containing protein 1 OS=Homo sapiens GN=FRMD1 PE=2 SV=2 - [FRMD1_HUMAN] |
| Q9Y2L6 | FERM domain-containing protein 4B OS=Homo sapiens GN=FRMD4B PE=1 SV=4 - [FRM4B_HUMAN] |
| Q9Y4F1 | FERM, RhoGEF and pleckstrin domain-containing protein 1 OS=Homo sapiens GN=FARP1 PE=1 SV=1 - [FARP1_HUMAN] |
| P02792 | Ferritin light chain OS=Homo sapiens GN=FTL PE=1 SV=2 - [FRIL_HUMAN] |
| Q969F0 | Fetal and adult testis-expressed transcript protein OS=Homo sapiens GN=FATE1 PE=1 SV=1 - [FATE1_HUMAN] |
| A0PJY2 | Fez family zinc finger protein 1 OS=Homo sapiens GN=FEZF1 PE=1 SV=1 - [FEZF1_HUMAN] |
| P35555 | Fibrillin-1 OS=Homo sapiens GN=FBN1 PE=1 SV=3 - [FBN1_HUMAN] |
| P02675 | Fibrinogen beta chain OS=Homo sapiens GN=FGB PE=1 SV=2 - [FIBB_HUMAN] |
| P09038 | Fibroblast growth factor 2 OS=Homo sapiens GN=FGF2 PE=1 SV=3 - [FGF2_HUMAN] |
| P08620 | Fibroblast growth factor 4 OS=Homo sapiens GN=FGF4 PE=1 SV=1 - [FGF4_HUMAN] |
| Q86WI1 | Fibrocystin-L OS=Homo sapiens GN=PKHD1L1 PE=2 SV=2 - [PKHL1_HUMAN] |
| Q4ZHG4 | Fibronectin type III domain-containing protein 1 OS=Homo sapiens GN=FNDC1 PE=2 SV=4 - [FNDC1_HUMAN] |
| Q5CZC0 | Fibrous sheath-interacting protein 2 OS=Homo sapiens GN=FSIP2 PE=2 SV=4 - [FSIP2_HUMAN] |
| P20930 | Filaggrin OS=Homo sapiens GN=FLG PE=1 SV=3 - [FILA_HUMAN] |
| P21333 | Filamin-A OS=Homo sapiens GN=FLNA PE=1 SV=4 - [FLNA_HUMAN] |
| O75369 | Filamin-B OS=Homo sapiens GN=FLNB PE=1 SV=2 - [FLNB_HUMAN] |
| P39748 | Flap endonuclease 1 OS=Homo sapiens GN=FEN1 PE=1 SV=1 - [FEN1_HUMAN] |
| O75955 | Flotillin-1 OS=Homo sapiens GN=FLOT1 PE=1 SV=3 - [FLOT1_HUMAN] |
| Q4VC44 | FLYWCH-type zinc finger-containing protein 1 OS=Homo sapiens GN=FLYWCH1 PE=1 SV=2 - [FWCH1_HUMAN] |
| P41440 | Folate transporter 1 OS=Homo sapiens GN=SLC19A1 PE=1 SV=3 - [S19A1_HUMAN] |
| P23945 | Follicle-stimulating hormone receptor OS=Homo sapiens GN=FSHR PE=1 SV=3 - [FSHR_HUMAN] |
| Q9P278 | Folliculin-interacting protein 2 OS=Homo sapiens GN=FNIP2 PE=1 SV=2 - [FNIP2_HUMAN] |
| P98177 | Forkhead box protein O4 OS=Homo sapiens GN=FOXO4 PE=1 SV=5 - [FOXO4_HUMAN] |
| O43638 | Forkhead box protein S1 OS=Homo sapiens GN=FOXS1 PE=2 SV=2 - [FOXS1_HUMAN] |
| Q9NZ56 | Formin-2 OS=Homo sapiens GN=FMN2 PE=1 SV=4 - [FMN2_HUMAN] |
| Q8N3X1 | Formin-binding protein 4 OS=Homo sapiens GN=FNBP4 PE=1 SV=3 - [FNBP4_HUMAN] |
| P15408 | Fos-related antigen 2 OS=Homo sapiens GN=FOSL2 PE=1 SV=1 - [FOSL2_HUMAN] |
| P51114 | Fragile X mental retardation syndrome-related protein 1 OS=Homo sapiens GN=FXR1 PE=1 SV=3 - [FXR1_HUMAN] |
| Q9UP38 | Frizzled-1 OS=Homo sapiens GN=FZD1 PE=1 SV=2 - [FZD1_HUMAN] |
| Q9ULV1 | Frizzled-4 OS=Homo sapiens GN=FZD4 PE=1 SV=2 - [FZD4_HUMAN] |
| Q13467 | Frizzled-5 OS=Homo sapiens GN=FZD5 PE=1 SV=2 - [FZD5_HUMAN] |
| Q9H479 | Fructosamine-3-kinase OS=Homo sapiens GN=FN3K PE=1 SV=1 - [FN3K_HUMAN] |
| P09467 | Fructose-1,6-bisphosphatase 1 OS=Homo sapiens GN=FBP1 PE=1 SV=5 - [F16P1_HUMAN] |
| P04075 | Fructose-bisphosphate aldolase A OS=Homo sapiens GN=ALDOA PE=1 SV=2 - [ALDOA_HUMAN] |
| O75072 | Fukutin OS=Homo sapiens GN=FKTN PE=1 SV=2 - [FKTN_HUMAN] |
| Q6P2I3 | Fumarylacetoacetate hydrolase domain-containing protein 2B OS=Homo sapiens GN=FAHD2B PE=2 SV=1 - [FAH2B_HUMAN] |
| O15117 | FYN-binding protein OS=Homo sapiens GN=FYB PE=1 SV=2 - [FYB_HUMAN] |
| Q6ZV73 | FYVE, RhoGEF and PH domain-containing protein 6 OS=Homo sapiens GN=FGD6 PE=1 SV=2 - [FGD6_HUMAN] |
| Q9UKJ3 | G patch domain-containing protein 8 OS=Homo sapiens GN=GPATCH8 PE=1 SV=2 - [GPTC8_HUMAN] |
| P48549 | G protein-activated inward rectifier potassium channel 1 OS=Homo sapiens GN=KCNJ3 PE=1 SV=1 - [KCNJ3_HUMAN] |
| Q92806 | G protein-activated inward rectifier potassium channel 3 OS=Homo sapiens GN=KCNJ9 PE=2 SV=2 - [KCNJ9_HUMAN] |
| P27469 | G0/G1 switch protein 2 OS=Homo sapiens GN=G0S2 PE=1 SV=1 - [G0S2_HUMAN] |
| Q9NYZ3 | G2 and S phase-expressed protein 1 OS=Homo sapiens GN=GTSE1 PE=1 SV=3 - [GTSE1_HUMAN] |
| Q7L622 | G2/M phase-specific E3 ubiquitin-protein ligase OS=Homo sapiens GN=G2E3 PE=1 SV=1 - [G2E3_HUMAN] |
| Q8WWL7 | G2/mitotic-specific cyclin-B3 OS=Homo sapiens GN=CCNB3 PE=1 SV=2 - [CCNB3_HUMAN] |
| Q06546 | GA-binding protein alpha chain OS=Homo sapiens GN=GABPA PE=1 SV=1 - [GABPA_HUMAN] |
| P07902 | Galactose-1-phosphate uridylyltransferase OS=Homo sapiens GN=GALT PE=1 SV=3 - [GALT_HUMAN] |
| P09382 | Galectin-1 OS=Homo sapiens GN=LGALS1 PE=1 SV=2 - [LEG1_HUMAN] |
| Q86UU5 | Gametogenetin OS=Homo sapiens GN=GGN PE=1 SV=2 - [GGN_HUMAN] |
| P24046 | Gamma-aminobutyric acid receptor subunit rho-1 OS=Homo sapiens GN=GABRR1 PE=2 SV=2 - [GBRR1_HUMAN] |
| Q9UBS5 | Gamma-aminobutyric acid type B receptor subunit 1 OS=Homo sapiens GN=GABBR1 PE=1 SV=1 - [GABR1_HUMAN] |
| Q92820 | Gamma-glutamyl hydrolase OS=Homo sapiens GN=GGH PE=1 SV=2 - [GGH_HUMAN] |
| Q9UJ14 | Gamma-glutamyltransferase 7 OS=Homo sapiens GN=GGT7 PE=1 SV=2 - [GGT7_HUMAN] |
| Q9HBI0 | Gamma-parvin OS=Homo sapiens GN=PARVG PE=1 SV=1 - [PARVG_HUMAN] |
| Q9BSJ2 | Gamma-tubulin complex component 2 OS=Homo sapiens GN=TUBGCP2 PE=1 SV=2 - [GCP2_HUMAN] |
| P17302 | Gap junction alpha-1 protein OS=Homo sapiens GN=GJA1 PE=1 SV=2 - [CXA1_HUMAN] |
| Q9Y6H8 | Gap junction alpha-3 protein OS=Homo sapiens GN=GJA3 PE=1 SV=4 - [CXA3_HUMAN] |
| P57773 | Gap junction alpha-9 protein OS=Homo sapiens GN=GJA9 PE=2 SV=2 - [CXA9_HUMAN] |
| Q6PEY0 | Gap junction beta-7 protein OS=Homo sapiens GN=GJB7 PE=2 SV=1 - [CXB7_HUMAN] |
| Q86XJ1 | GAS2-like protein 3 OS=Homo sapiens GN=GAS2L3 PE=1 SV=1 - [GA2L3_HUMAN] |
| P32239 | Gastrin/cholecystokinin type B receptor OS=Homo sapiens GN=CCKBR PE=1 SV=1 - [GASR_HUMAN] |
| P0CG01 | Gastrokine-3 OS=Homo sapiens GN=GKN3P PE=3 SV=1 - [GKN3_HUMAN] |
| P57678 | Gem-associated protein 4 OS=Homo sapiens GN=GEMIN4 PE=1 SV=2 - [GEMI4_HUMAN] |
| Q12789 | General transcription factor 3C polypeptide 1 OS=Homo sapiens GN=GTF3C1 PE=1 SV=4 - [TF3C1_HUMAN] |
| P78347 | General transcription factor II-I OS=Homo sapiens GN=GTF2I PE=1 SV=2 - [GTF2I_HUMAN] |
| O60763 | General vesicular transport factor p115 OS=Homo sapiens GN=USO1 PE=1 SV=2 - [USO1_HUMAN] |
| Q14687 | Genetic suppressor element 1 OS=Homo sapiens GN=GSE1 PE=1 SV=3 - [GSE1_HUMAN] |
| O60318 | Germinal-center associated nuclear protein OS=Homo sapiens GN=MCM3AP PE=1 SV=2 - [GANP_HUMAN] |
| Q3V6T2 | Girdin OS=Homo sapiens GN=CCDC88A PE=1 SV=2 - [GRDN_HUMAN] |
| P07093 | Glia-derived nexin OS=Homo sapiens GN=SERPINE2 PE=1 SV=1 - [GDN_HUMAN] |
| Q6ZMI3 | Gliomedin OS=Homo sapiens GN=GLDN PE=2 SV=1 - [GLDN_HUMAN] |
| O95838 | Glucagon-like peptide 2 receptor OS=Homo sapiens GN=GLP2R PE=2 SV=1 - [GLP2R_HUMAN] |
| P04150 | Glucocorticoid receptor OS=Homo sapiens GN=NR3C1 PE=1 SV=1 - [GCR_HUMAN] |
| P06744 | Glucose-6-phosphate isomerase OS=Homo sapiens GN=GPI PE=1 SV=4 - [G6PI_HUMAN] |
| Q4G148 | Glucoside xylosyltransferase 1 OS=Homo sapiens GN=GXYLT1 PE=1 SV=2 - [GXLT1_HUMAN] |
| A0PJZ3 | Glucoside xylosyltransferase 2 OS=Homo sapiens GN=GXYLT2 PE=2 SV=2 - [GXLT2_HUMAN] |
| Q13002 | Glutamate receptor ionotropic, kainate 2 OS=Homo sapiens GN=GRIK2 PE=1 SV=1 - [GRIK2_HUMAN] |
| O15399 | Glutamate receptor ionotropic, NMDA 2D OS=Homo sapiens GN=GRIN2D PE=1 SV=2 - [NMDE4_HUMAN] |
| Q86X53 | Glutamate-rich protein 1 OS=Homo sapiens GN=ERICH1 PE=1 SV=1 - [ERIC1_HUMAN] |
| Q2KHR3 | Glutamine and serine-rich protein 1 OS=Homo sapiens GN=QSER1 PE=1 SV=3 - [QSER1_HUMAN] |
| Q06210 | Glutamine--fructose-6-phosphate aminotransferase [isomerizing] 1 OS=Homo sapiens GN=GFPT1 PE=1 SV=3 - [GFPT1_HUMAN] |
| Q9H0J4 | Glutamine-rich protein 2 OS=Homo sapiens GN=QRICH2 PE=1 SV=1 - [QRIC2_HUMAN] |
| P47897 | Glutamine--tRNA ligase OS=Homo sapiens GN=QARS PE=1 SV=1 - [SYQ_HUMAN] |
| Q07075 | Glutamyl aminopeptidase OS=Homo sapiens GN=ENPEP PE=1 SV=3 - [AMPE_HUMAN] |
| P00390 | Glutathione reductase, mitochondrial OS=Homo sapiens GN=GSR PE=1 SV=2 - [GSHR_HUMAN] |
| Q8NEC7 | Glutathione S-transferase C-terminal domain-containing protein OS=Homo sapiens GN=GSTCD PE=1 SV=2 - [GSTCD_HUMAN] |
| P09211 | Glutathione S-transferase P OS=Homo sapiens GN=GSTP1 PE=1 SV=2 - [GSTP1_HUMAN] |
| P04406 | Glyceraldehyde-3-phosphate dehydrogenase OS=Homo sapiens GN=GAPDH PE=1 SV=3 - [G3P_HUMAN] |
| Q8N335 | Glycerol-3-phosphate dehydrogenase 1-like protein OS=Homo sapiens GN=GPD1L PE=1 SV=1 - [GPD1L_HUMAN] |
| P41250 | Glycine--tRNA ligase OS=Homo sapiens GN=GARS PE=1 SV=3 - [SYG_HUMAN] |
| P13807 | Glycogen [starch] synthase, muscle OS=Homo sapiens GN=GYS1 PE=1 SV=2 - [GYS1_HUMAN] |
| P06737 | Glycogen phosphorylase, liver form OS=Homo sapiens GN=PYGL PE=1 SV=4 - [PYGL_HUMAN] |
| P11217 | Glycogen phosphorylase, muscle form OS=Homo sapiens GN=PYGM PE=1 SV=6 - [PYGM_HUMAN] |
| Q9NU53 | Glycoprotein integral membrane protein 1 OS=Homo sapiens GN=GINM1 PE=2 SV=1 - [GINM1_HUMAN] |
| Q9H1C3 | Glycosyltransferase 8 domain-containing protein 2 OS=Homo sapiens GN=GLT8D2 PE=2 SV=1 - [GL8D2_HUMAN] |
| Q13439 | Golgin subfamily A member 4 OS=Homo sapiens GN=GOLGA4 PE=1 SV=1 - [GOGA4_HUMAN] |
| Q14789 | Golgin subfamily B member 1 OS=Homo sapiens GN=GOLGB1 PE=1 SV=2 - [GOGB1_HUMAN] |
| Q92538 | Golgi-specific brefeldin A-resistance guanine nucleotide exchange factor 1 OS=Homo sapiens GN=GBF1 PE=1 SV=2 - [GBF1_HUMAN] |
| Q75T13 | GPI inositol-deacylase OS=Homo sapiens GN=PGAP1 PE=1 SV=1 - [PGAP1_HUMAN] |
| Q9NUD9 | GPI mannosyltransferase 2 OS=Homo sapiens GN=PIGV PE=1 SV=1 - [PIGV_HUMAN] |
| Q969N2 | GPI transamidase component PIG-T OS=Homo sapiens GN=PIGT PE=1 SV=1 - [PIGT_HUMAN] |
| Q96PE1 | G-protein coupled receptor 124 OS=Homo sapiens GN=GPR124 PE=1 SV=2 - [GP124_HUMAN] |
| P51810 | G-protein coupled receptor 143 OS=Homo sapiens GN=GPR143 PE=1 SV=2 - [GP143_HUMAN] |
| P32249 | G-protein coupled receptor 183 OS=Homo sapiens GN=GPR183 PE=1 SV=3 - [GP183_HUMAN] |
| Q8WXG9 | G-protein coupled receptor 98 OS=Homo sapiens GN=GPR98 PE=1 SV=2 - [GPR98_HUMAN] |
| Q96D09 | G-protein coupled receptor-associated sorting protein 2 OS=Homo sapiens GN=GPRASP2 PE=1 SV=1 - [GASP2_HUMAN] |
| Q96CP6 | GRAM domain-containing protein 1A OS=Homo sapiens GN=GRAMD1A PE=1 SV=2 - [GRM1A_HUMAN] |
| Q96HH9 | GRAM domain-containing protein 3 OS=Homo sapiens GN=GRAMD3 PE=1 SV=1 - [GRAM3_HUMAN] |
| Q8IWJ2 | GRIP and coiled-coil domain-containing protein 2 OS=Homo sapiens GN=GCC2 PE=1 SV=4 - [GCC2_HUMAN] |
| Q92847 | Growth hormone secretagogue receptor type 1 OS=Homo sapiens GN=GHSR PE=1 SV=1 - [GHSR_HUMAN] |
| P43026 | Growth/differentiation factor 5 OS=Homo sapiens GN=GDF5 PE=1 SV=3 - [GDF5_HUMAN] |
| Q7Z4P5 | Growth/differentiation factor 7 OS=Homo sapiens GN=GDF7 PE=2 SV=2 - [GDF7_HUMAN] |
| O14793 | Growth/differentiation factor 8 OS=Homo sapiens GN=MSTN PE=1 SV=1 - [GDF8_HUMAN] |
| Q9HAV7 | GrpE protein homolog 1, mitochondrial OS=Homo sapiens GN=GRPEL1 PE=1 SV=2 - [GRPE1_HUMAN] |
| Q6P9H5 | GTPase IMAP family member 6 OS=Homo sapiens GN=GIMAP6 PE=2 SV=1 - [GIMA6_HUMAN] |
| Q8NHV1 | GTPase IMAP family member 7 OS=Homo sapiens GN=GIMAP7 PE=1 SV=1 - [GIMA7_HUMAN] |
| Q8ND71 | GTPase IMAP family member 8 OS=Homo sapiens GN=GIMAP8 PE=2 SV=2 - [GIMA8_HUMAN] |
| Q8IYK8 | GTP-binding protein REM 2 OS=Homo sapiens GN=REM2 PE=1 SV=2 - [REM2_HUMAN] |
| Q5JWF2 | Guanine nucleotide-binding protein G(s) subunit alpha isoforms XLas OS=Homo sapiens GN=GNAS PE=1 SV=2 - [GNAS1_HUMAN] |
| P11488 | Guanine nucleotide-binding protein G(t) subunit alpha-1 OS=Homo sapiens GN=GNAT1 PE=1 SV=5 - [GNAT1_HUMAN] |
| P30679 | Guanine nucleotide-binding protein subunit alpha-15 OS=Homo sapiens GN=GNA15 PE=1 SV=2 - [GNA15_HUMAN] |
| Q9HAV0 | Guanine nucleotide-binding protein subunit beta-4 OS=Homo sapiens GN=GNB4 PE=1 SV=3 - [GBB4_HUMAN] |
| P36915 | Guanine nucleotide-binding protein-like 1 OS=Homo sapiens GN=GNL1 PE=1 SV=2 - [GNL1_HUMAN] |
| P33402 | Guanylate cyclase soluble subunit alpha-2 OS=Homo sapiens GN=GUCY1A2 PE=2 SV=1 - [GCYA2_HUMAN] |
| Q16774 | Guanylate kinase OS=Homo sapiens GN=GUK1 PE=1 SV=2 - [KGUA_HUMAN] |
| P51795 | H(+)/Cl(-) exchange transporter 5 OS=Homo sapiens GN=CLCN5 PE=1 SV=1 - [CLCN5_HUMAN] |
| P00738 | Haptoglobin OS=Homo sapiens GN=HP PE=1 SV=1 - [HPT_HUMAN] |
| Q9H6D7 | HAUS augmin-like complex subunit 4 OS=Homo sapiens GN=HAUS4 PE=1 SV=1 - [HAUS4_HUMAN] |
| Q99871 | HAUS augmin-like complex subunit 7 OS=Homo sapiens GN=HAUS7 PE=1 SV=3 - [HAUS7_HUMAN] |
| Q9Y450 | HBS1-like protein OS=Homo sapiens GN=HBS1L PE=1 SV=1 - [HBS1L_HUMAN] |
| Q9UBI9 | Headcase protein homolog OS=Homo sapiens GN=HECA PE=1 SV=1 - [HDC_HUMAN] |
| O43301 | Heat shock 70 kDa protein 12A OS=Homo sapiens GN=HSPA12A PE=1 SV=2 - [HS12A_HUMAN] |
| O95757 | Heat shock 70 kDa protein 4L OS=Homo sapiens GN=HSPA4L PE=1 SV=3 - [HS74L_HUMAN] |
| O75031 | Heat shock factor 2-binding protein OS=Homo sapiens GN=HSF2BP PE=1 SV=1 - [HSF2B_HUMAN] |
| P04792 | Heat shock protein beta-1 OS=Homo sapiens GN=HSPB1 PE=1 SV=2 - [HSPB1_HUMAN] |
| P08238 | Heat shock protein HSP 90-beta OS=Homo sapiens GN=HSP90AB1 PE=1 SV=4 - [HS90B_HUMAN] |
| P54652 | Heat shock-related 70 kDa protein 2 OS=Homo sapiens GN=HSPA2 PE=1 SV=1 - [HSP72_HUMAN] |
| Q9Y4B4 | Helicase ARIP4 OS=Homo sapiens GN=RAD54L2 PE=1 SV=4 - [ARIP4_HUMAN] |
| Q15477 | Helicase SKI2W OS=Homo sapiens GN=SKIV2L PE=1 SV=3 - [SKIV2_HUMAN] |
| Q6ZRS2 | Helicase SRCAP OS=Homo sapiens GN=SRCAP PE=1 SV=3 - [SRCAP_HUMAN] |
| P14317 | Hematopoietic lineage cell-specific protein OS=Homo sapiens GN=HCLS1 PE=1 SV=3 - [HCLS1_HUMAN] |
| P09601 | Heme oxygenase 1 OS=Homo sapiens GN=HMOX1 PE=1 SV=1 - [HMOX1_HUMAN] |
| Q96RW7 | Hemicentin-1 OS=Homo sapiens GN=HMCN1 PE=1 SV=2 - [HMCN1_HUMAN] |
| Q8NDA2 | Hemicentin-2 OS=Homo sapiens GN=HMCN2 PE=2 SV=2 - [HMCN2_HUMAN] |
| Q9Y5R4 | HemK methyltransferase family member 1 OS=Homo sapiens GN=HEMK1 PE=1 SV=1 - [HEMK1_HUMAN] |
| P69905 | Hemoglobin subunit alpha OS=Homo sapiens GN=HBA1 PE=1 SV=2 - [HBA_HUMAN] |
| P68871 | Hemoglobin subunit beta OS=Homo sapiens GN=HBB PE=1 SV=2 - [HBB_HUMAN] |
| Q96MM7 | Heparan-sulfate 6-O-sulfotransferase 2 OS=Homo sapiens GN=HS6ST2 PE=2 SV=2 - [H6ST2_HUMAN] |
| Q14541 | Hepatocyte nuclear factor 4-gamma OS=Homo sapiens GN=HNF4G PE=1 SV=3 - [HNF4G_HUMAN] |
| Q5TGJ6 | Hepatoma-derived growth factor-like protein 1 OS=Homo sapiens GN=HDGFL1 PE=2 SV=1 - [HDGL1_HUMAN] |
| Q9UPZ3 | Hermansky-Pudlak syndrome 5 protein OS=Homo sapiens GN=HPS5 PE=1 SV=2 - [HPS5_HUMAN] |
| Q86YV9 | Hermansky-Pudlak syndrome 6 protein OS=Homo sapiens GN=HPS6 PE=1 SV=1 - [HPS6_HUMAN] |
| Q9N2J8 | HERV-H_2q24.1 provirus ancestral Env polyprotein OS=Homo sapiens PE=2 SV=1 - [ENH3_HUMAN] |
| Q14103 | Heterogeneous nuclear ribonucleoprotein D0 OS=Homo sapiens GN=HNRNPD PE=1 SV=1 - [HNRPD_HUMAN] |
| O14979 | Heterogeneous nuclear ribonucleoprotein D-like OS=Homo sapiens GN=HNRNPDL PE=1 SV=3 - [HNRDL_HUMAN] |
| P52597 | Heterogeneous nuclear ribonucleoprotein F OS=Homo sapiens GN=HNRNPF PE=1 SV=3 - [HNRPF_HUMAN] |
| P52272 | Heterogeneous nuclear ribonucleoprotein M OS=Homo sapiens GN=HNRNPM PE=1 SV=3 - [HNRPM_HUMAN] |
| P22626 | Heterogeneous nuclear ribonucleoproteins A2/B1 OS=Homo sapiens GN=HNRNPA2B1 PE=1 SV=2 - [ROA2_HUMAN] |
| P07910 | Heterogeneous nuclear ribonucleoproteins C1/C2 OS=Homo sapiens GN=HNRNPC PE=1 SV=4 - [HNRPC_HUMAN] |
| Q6UWX4 | HHIP-like protein 2 OS=Homo sapiens GN=HHIPL2 PE=1 SV=1 - [HIPL2_HUMAN] |
| Q01362 | High affinity immunoglobulin epsilon receptor subunit beta OS=Homo sapiens GN=MS4A2 PE=2 SV=1 - [FCERB_HUMAN] |
| P82970 | High mobility group nucleosome-binding domain-containing protein 5 OS=Homo sapiens GN=HMGN5 PE=1 SV=1 - [HMGN5_HUMAN] |
| P17096 | High mobility group protein HMG-I/HMG-Y OS=Homo sapiens GN=HMGA1 PE=1 SV=3 - [HMGA1_HUMAN] |
| Q9BW71 | HIRA-interacting protein 3 OS=Homo sapiens GN=HIRIP3 PE=1 SV=3 - [HIRP3_HUMAN] |
| Q9Y5N1 | Histamine H3 receptor OS=Homo sapiens GN=HRH3 PE=1 SV=2 - [HRH3_HUMAN] |
| P19113 | Histidine decarboxylase OS=Homo sapiens GN=HDC PE=1 SV=2 - [DCHS_HUMAN] |
| Q92830 | Histone acetyltransferase KAT2A OS=Homo sapiens GN=KAT2A PE=1 SV=3 - [KAT2A_HUMAN] |
| Q92831 | Histone acetyltransferase KAT2B OS=Homo sapiens GN=KAT2B PE=1 SV=3 - [KAT2B_HUMAN] |
| Q92993 | Histone acetyltransferase KAT5 OS=Homo sapiens GN=KAT5 PE=1 SV=2 - [KAT5_HUMAN] |
| Q8WYB5 | Histone acetyltransferase KAT6B OS=Homo sapiens GN=KAT6B PE=1 SV=3 - [KAT6B_HUMAN] |
| O95251 | Histone acetyltransferase KAT7 OS=Homo sapiens GN=KAT7 PE=1 SV=1 - [KAT7_HUMAN] |
| Q09472 | Histone acetyltransferase p300 OS=Homo sapiens GN=EP300 PE=1 SV=2 - [EP300_HUMAN] |
| Q92769 | Histone deacetylase 2 OS=Homo sapiens GN=HDAC2 PE=1 SV=2 - [HDAC2_HUMAN] |
| Q9UQL6 | Histone deacetylase 5 OS=Homo sapiens GN=HDAC5 PE=1 SV=2 - [HDAC5_HUMAN] |
| Q9UKV0 | Histone deacetylase 9 OS=Homo sapiens GN=HDAC9 PE=1 SV=2 - [HDAC9_HUMAN] |
| P16403 | Histone H1.2 OS=Homo sapiens GN=HIST1H1C PE=1 SV=2 - [H12_HUMAN] |
| P16401 | Histone H1.5 OS=Homo sapiens GN=HIST1H1B PE=1 SV=3 - [H15_HUMAN] |
| Q5VVJ2 | Histone H2A deubiquitinase MYSM1 OS=Homo sapiens GN=MYSM1 PE=1 SV=1 - [MYSM1_HUMAN] |
| Q96KK5 | Histone H2A type 1-H OS=Homo sapiens GN=HIST1H2AH PE=1 SV=3 - [H2A1H_HUMAN] |
| Q16777 | Histone H2A type 2-C OS=Homo sapiens GN=HIST2H2AC PE=1 SV=4 - [H2A2C_HUMAN] |
| P23527 | Histone H2B type 1-O OS=Homo sapiens GN=HIST1H2BO PE=1 SV=3 - [H2B1O_HUMAN] |
| Q6NXT2 | Histone H3.3C OS=Homo sapiens GN=H3F3C PE=1 SV=3 - [H3C_HUMAN] |
| P62805 | Histone H4 OS=Homo sapiens GN=HIST1H4A PE=1 SV=2 - [H4_HUMAN] |
| Q9UMN6 | Histone-lysine N-methyltransferase 2B OS=Homo sapiens GN=KMT2B PE=1 SV=1 - [KMT2B_HUMAN] |
| Q8NEZ4 | Histone-lysine N-methyltransferase 2C OS=Homo sapiens GN=KMT2C PE=1 SV=3 - [KMT2C_HUMAN] |
| O14686 | Histone-lysine N-methyltransferase 2D OS=Homo sapiens GN=KMT2D PE=1 SV=2 - [KMT2D_HUMAN] |
| Q8IZD2 | Histone-lysine N-methyltransferase 2E OS=Homo sapiens GN=KMT2E PE=1 SV=1 - [KMT2E_HUMAN] |
| Q92800 | Histone-lysine N-methyltransferase EZH1 OS=Homo sapiens GN=EZH1 PE=1 SV=2 - [EZH1_HUMAN] |
| O15047 | Histone-lysine N-methyltransferase SETD1A OS=Homo sapiens GN=SETD1A PE=1 SV=3 - [SET1A_HUMAN] |
| Q9BYW2 | Histone-lysine N-methyltransferase SETD2 OS=Homo sapiens GN=SETD2 PE=1 SV=3 - [SETD2_HUMAN] |
| Q15047 | Histone-lysine N-methyltransferase SETDB1 OS=Homo sapiens GN=SETDB1 PE=1 SV=1 - [SETB1_HUMAN] |
| Q96T68 | Histone-lysine N-methyltransferase SETDB2 OS=Homo sapiens GN=SETDB2 PE=1 SV=2 - [SETB2_HUMAN] |
| O43463 | Histone-lysine N-methyltransferase SUV39H1 OS=Homo sapiens GN=SUV39H1 PE=1 SV=1 - [SUV91_HUMAN] |
| Q8TEK3 | Histone-lysine N-methyltransferase, H3 lysine-79 specific OS=Homo sapiens GN=DOT1L PE=1 SV=2 - [DOT1L_HUMAN] |
| P30511 | HLA class I histocompatibility antigen, alpha chain F OS=Homo sapiens GN=HLA-F PE=2 SV=3 - [HLAF_HUMAN] |
| P01909 | HLA class II histocompatibility antigen, DQ alpha 1 chain OS=Homo sapiens GN=HLA-DQA1 PE=1 SV=1 - [DQA1_HUMAN] |
| P05538 | HLA class II histocompatibility antigen, DQ beta 2 chain OS=Homo sapiens GN=HLA-DQB2 PE=1 SV=2 - [DQB2_HUMAN] |
| Q12766 | HMG domain-containing protein 3 OS=Homo sapiens GN=HMGXB3 PE=2 SV=2 - [HMGX3_HUMAN] |
| P47902 | Homeobox protein CDX-1 OS=Homo sapiens GN=CDX1 PE=1 SV=2 - [CDX1_HUMAN] |
| P39880 | Homeobox protein cut-like 1 OS=Homo sapiens GN=CUX1 PE=1 SV=3 - [CUX1_HUMAN] |
| Q9NYD6 | Homeobox protein Hox-C10 OS=Homo sapiens GN=HOXC10 PE=1 SV=2 - [HXC10_HUMAN] |
| P52952 | Homeobox protein Nkx-2.5 OS=Homo sapiens GN=NKX2-5 PE=1 SV=1 - [NKX25_HUMAN] |
| Q99801 | Homeobox protein Nkx-3.1 OS=Homo sapiens GN=NKX3-1 PE=1 SV=2 - [NKX31_HUMAN] |
| Q8IUE1 | Homeobox protein TGIF2LX OS=Homo sapiens GN=TGIF2LX PE=1 SV=1 - [TF2LX_HUMAN] |
| Q05469 | Hormone-sensitive lipase OS=Homo sapiens GN=LIPE PE=1 SV=4 - [LIPS_HUMAN] |
| P51610 | Host cell factor 1 OS=Homo sapiens GN=HCFC1 PE=1 SV=2 - [HCFC1_HUMAN] |
| P53816 | HRAS-like suppressor 3 OS=Homo sapiens GN=PLA2G16 PE=1 SV=2 - [HRSL3_HUMAN] |
| P0CJ69 | Humanin-like 2 OS=Homo sapiens GN=MTRNR2L2 PE=2 SV=1 - [HMN2_HUMAN] |
| P42858 | Huntingtin OS=Homo sapiens GN=HTT PE=1 SV=2 - [HD_HUMAN] |
| O00219 | Hyaluronan synthase 3 OS=Homo sapiens GN=HAS3 PE=2 SV=3 - [HYAS3_HUMAN] |
| P35914 | Hydroxymethylglutaryl-CoA lyase, mitochondrial OS=Homo sapiens GN=HMGCL PE=1 SV=2 - [HMGCL_HUMAN] |
| P01857 | Ig gamma-1 chain C region OS=Homo sapiens GN=IGHG1 PE=1 SV=1 - [IGHG1_HUMAN] |
| P01814 | Ig heavy chain V-II region OU OS=Homo sapiens PE=1 SV=1 - [HV201_HUMAN] |
| P01834 | Ig kappa chain C region OS=Homo sapiens GN=IGKC PE=1 SV=1 - [IGKC_HUMAN] |
| P01620 | Ig kappa chain V-III region SIE OS=Homo sapiens PE=1 SV=1 - [KV302_HUMAN] |
| P01706 | Ig lambda chain V-II region BOH OS=Homo sapiens PE=1 SV=1 - [LV203_HUMAN] |
| P01708 | Ig lambda chain V-II region BUR OS=Homo sapiens PE=1 SV=1 - [LV205_HUMAN] |
| Q8WZA9 | Immunity-related GTPase family Q protein OS=Homo sapiens GN=IRGQ PE=1 SV=1 - [IRGQ_HUMAN] |
| P01591 | Immunoglobulin J chain OS=Homo sapiens GN=IGJ PE=1 SV=4 - [IGJ_HUMAN] |
| Q6WRI0 | Immunoglobulin superfamily member 10 OS=Homo sapiens GN=IGSF10 PE=2 SV=1 - [IGS10_HUMAN] |
| Q93033 | Immunoglobulin superfamily member 2 OS=Homo sapiens GN=CD101 PE=1 SV=2 - [IGSF2_HUMAN] |
| O75054 | Immunoglobulin superfamily member 3 OS=Homo sapiens GN=IGSF3 PE=2 SV=3 - [IGSF3_HUMAN] |
| Q969P0 | Immunoglobulin superfamily member 8 OS=Homo sapiens GN=IGSF8 PE=1 SV=1 - [IGSF8_HUMAN] |
| Q86VF2 | Immunoglobulin-like and fibronectin type III domain-containing protein 1 OS=Homo sapiens GN=IGFN1 PE=1 SV=2 - [IGFN1_HUMAN] |
| Q8TEX9 | Importin-4 OS=Homo sapiens GN=IPO4 PE=1 SV=2 - [IPO4_HUMAN] |
| O00410 | Importin-5 OS=Homo sapiens GN=IPO5 PE=1 SV=4 - [IPO5_HUMAN] |
| Q8N608 | Inactive dipeptidyl peptidase 10 OS=Homo sapiens GN=DPP10 PE=1 SV=2 - [DPP10_HUMAN] |
| Q7Z4T8 | Inactive polypeptide N-acetylgalactosaminyltransferase-like protein 5 OS=Homo sapiens GN=GALNTL5 PE=2 SV=3 - [GLTL5_HUMAN] |
| Q8NI35 | InaD-like protein OS=Homo sapiens GN=INADL PE=1 SV=3 - [INADL_HUMAN] |
| P20839 | Inosine-5'-monophosphate dehydrogenase 1 OS=Homo sapiens GN=IMPDH1 PE=1 SV=2 - [IMDH1_HUMAN] |
| Q6GPH6 | Inositol 1,4,5-trisphosphate receptor-interacting protein-like 1 OS=Homo sapiens GN=ITPRIPL1 PE=1 SV=1 - [IPIL1_HUMAN] |
| P23677 | Inositol-trisphosphate 3-kinase A OS=Homo sapiens GN=ITPKA PE=1 SV=1 - [IP3KA_HUMAN] |
| Q96DU7 | Inositol-trisphosphate 3-kinase C OS=Homo sapiens GN=ITPKC PE=1 SV=1 - [IP3KC_HUMAN] |
| Q9Y4H2 | Insulin receptor substrate 2 OS=Homo sapiens GN=IRS2 PE=1 SV=2 - [IRS2_HUMAN] |
| O14654 | Insulin receptor substrate 4 OS=Homo sapiens GN=IRS4 PE=1 SV=1 - [IRS4_HUMAN] |
| Q9NZI8 | Insulin-like growth factor 2 mRNA-binding protein 1 OS=Homo sapiens GN=IGF2BP1 PE=1 SV=2 - [IF2B1_HUMAN] |
| P08833 | Insulin-like growth factor-binding protein 1 OS=Homo sapiens GN=IGFBP1 PE=1 SV=1 - [IBP1_HUMAN] |
| Q86V85 | Integral membrane protein GPR180 OS=Homo sapiens GN=GPR180 PE=2 SV=1 - [GP180_HUMAN] |
| Q8N201 | Integrator complex subunit 1 OS=Homo sapiens GN=INTS1 PE=1 SV=2 - [INT1_HUMAN] |
| Q9NVH2 | Integrator complex subunit 7 OS=Homo sapiens GN=INTS7 PE=1 SV=1 - [INT7_HUMAN] |
| O75578 | Integrin alpha-10 OS=Homo sapiens GN=ITGA10 PE=2 SV=2 - [ITA10_HUMAN] |
| P38570 | Integrin alpha-E OS=Homo sapiens GN=ITGAE PE=1 SV=3 - [ITAE_HUMAN] |
| P19827 | Inter-alpha-trypsin inhibitor heavy chain H1 OS=Homo sapiens GN=ITIH1 PE=1 SV=3 - [ITIH1_HUMAN] |
| P19823 | Inter-alpha-trypsin inhibitor heavy chain H2 OS=Homo sapiens GN=ITIH2 PE=1 SV=2 - [ITIH2_HUMAN] |
| Q9UMF0 | Intercellular adhesion molecule 5 OS=Homo sapiens GN=ICAM5 PE=1 SV=3 - [ICAM5_HUMAN] |
| P17181 | Interferon alpha/beta receptor 1 OS=Homo sapiens GN=IFNAR1 PE=1 SV=3 - [INAR1_HUMAN] |
| P01562 | Interferon alpha-1/13 OS=Homo sapiens GN=IFNA1 PE=1 SV=1 - [IFNA1_HUMAN] |
| Q8IU57 | Interferon lambda receptor 1 OS=Homo sapiens GN=IFNLR1 PE=1 SV=1 - [INLR1_HUMAN] |
| Q8IU81 | Interferon regulatory factor 2-binding protein 1 OS=Homo sapiens GN=IRF2BP1 PE=1 SV=1 - [I2BP1_HUMAN] |
| P20591 | Interferon-induced GTP-binding protein Mx1 OS=Homo sapiens GN=MX1 PE=1 SV=4 - [MX1_HUMAN] |
| Q9BYX4 | Interferon-induced helicase C domain-containing protein 1 OS=Homo sapiens GN=IFIH1 PE=1 SV=3 - [IFIH1_HUMAN] |
| Q5T764 | Interferon-induced protein with tetratricopeptide repeats 1B OS=Homo sapiens GN=IFIT1B PE=2 SV=1 - [IFT1B_HUMAN] |
| Q7Z2Y8 | Interferon-induced very large GTPase 1 OS=Homo sapiens GN=GVINP1 PE=2 SV=2 - [GVIN1_HUMAN] |
| O00458 | Interferon-related developmental regulator 1 OS=Homo sapiens GN=IFRD1 PE=1 SV=4 - [IFRD1_HUMAN] |
| Q9NPH3 | Interleukin-1 receptor accessory protein OS=Homo sapiens GN=IL1RAP PE=1 SV=2 - [IL1AP_HUMAN] |
| Q9Y616 | Interleukin-1 receptor-associated kinase 3 OS=Homo sapiens GN=IRAK3 PE=1 SV=2 - [IRAK3_HUMAN] |
| Q16552 | Interleukin-17A OS=Homo sapiens GN=IL17A PE=1 SV=1 - [IL17_HUMAN] |
| P14784 | Interleukin-2 receptor subunit beta OS=Homo sapiens GN=IL2RB PE=1 SV=1 - [IL2RB_HUMAN] |
| Q5VWK5 | Interleukin-23 receptor OS=Homo sapiens GN=IL23R PE=1 SV=3 - [IL23R_HUMAN] |
| Q6UWB1 | Interleukin-27 receptor subunit alpha OS=Homo sapiens GN=IL27RA PE=2 SV=2 - [I27RA_HUMAN] |
| P05112 | Interleukin-4 OS=Homo sapiens GN=IL4 PE=1 SV=1 - [IL4_HUMAN] |
| P16871 | Interleukin-7 receptor subunit alpha OS=Homo sapiens GN=IL7R PE=1 SV=2 - [IL7RA_HUMAN] |
| Q9P2H3 | Intraflagellar transport protein 80 homolog OS=Homo sapiens GN=IFT80 PE=1 SV=3 - [IFT80_HUMAN] |
| Q27J81 | Inverted formin-2 OS=Homo sapiens GN=INF2 PE=1 SV=2 - [INF2_HUMAN] |
| Q8N2Y8 | Iporin OS=Homo sapiens GN=RUSC2 PE=1 SV=3 - [RUSC2_HUMAN] |
| Q6IPM2 | IQ domain-containing protein E OS=Homo sapiens GN=IQCE PE=1 SV=2 - [IQCE_HUMAN] |
| P0C7M6 | IQ domain-containing protein F3 OS=Homo sapiens GN=IQCF3 PE=2 SV=1 - [IQCF3_HUMAN] |
| Q5JU85 | IQ motif and SEC7 domain-containing protein 2 OS=Homo sapiens GN=IQSEC2 PE=1 SV=1 - [IQEC2_HUMAN] |
| P78414 | Iroquois-class homeodomain protein IRX-1 OS=Homo sapiens GN=IRX1 PE=2 SV=3 - [IRX1_HUMAN] |
| P50213 | Isocitrate dehydrogenase [NAD] subunit alpha, mitochondrial OS=Homo sapiens GN=IDH3A PE=1 SV=1 - [IDH3A_HUMAN] |
| Q8N9B5 | Junction-mediating and -regulatory protein OS=Homo sapiens GN=JMY PE=1 SV=2 - [JMY_HUMAN] |
| Q9HDC5 | Junctophilin-1 OS=Homo sapiens GN=JPH1 PE=1 SV=2 - [JPH1_HUMAN] |
| O60229 | Kalirin OS=Homo sapiens GN=KALRN PE=1 SV=2 - [KALRN_HUMAN] |
| O75449 | Katanin p60 ATPase-containing subunit A1 OS=Homo sapiens GN=KATNA1 PE=1 SV=1 - [KTNA1_HUMAN] |
| Q6PID8 | Kelch domain-containing protein 10 OS=Homo sapiens GN=KLHDC10 PE=1 SV=1 - [KLD10_HUMAN] |
| Q9BQ90 | Kelch domain-containing protein 3 OS=Homo sapiens GN=KLHDC3 PE=2 SV=1 - [KLDC3_HUMAN] |
| Q5VTJ3 | Kelch domain-containing protein 7A OS=Homo sapiens GN=KLHDC7A PE=1 SV=5 - [KLD7A_HUMAN] |
| Q3ZCT8 | Kelch repeat and BTB domain-containing protein 12 OS=Homo sapiens GN=KBTBD12 PE=2 SV=2 - [KBTBC_HUMAN] |
| Q8IY47 | Kelch repeat and BTB domain-containing protein 2 OS=Homo sapiens GN=KBTBD2 PE=1 SV=2 - [KBTB2_HUMAN] |
| Q96M94 | Kelch-like protein 15 OS=Homo sapiens GN=KLHL15 PE=1 SV=2 - [KLH15_HUMAN] |
| Q9UJP4 | Kelch-like protein 21 OS=Homo sapiens GN=KLHL21 PE=1 SV=4 - [KLH21_HUMAN] |
| Q9H0H3 | Kelch-like protein 25 OS=Homo sapiens GN=KLHL25 PE=1 SV=1 - [KLH25_HUMAN] |
| Q96CT2 | Kelch-like protein 29 OS=Homo sapiens GN=KLHL29 PE=2 SV=3 - [KLH29_HUMAN] |
| Q8N4N3 | Kelch-like protein 36 OS=Homo sapiens GN=KLHL36 PE=1 SV=1 - [KLH36_HUMAN] |
| Q2WGJ6 | Kelch-like protein 38 OS=Homo sapiens GN=KLHL38 PE=1 SV=3 - [KLH38_HUMAN] |
| Q92764 | Keratin, type I cuticular Ha5 OS=Homo sapiens GN=KRT35 PE=2 SV=5 - [KRT35_HUMAN] |
| P19012 | Keratin, type I cytoskeletal 15 OS=Homo sapiens GN=KRT15 PE=1 SV=3 - [K1C15_HUMAN] |
| P35900 | Keratin, type I cytoskeletal 20 OS=Homo sapiens GN=KRT20 PE=1 SV=1 - [K1C20_HUMAN] |
| Q9C075 | Keratin, type I cytoskeletal 23 OS=Homo sapiens GN=KRT23 PE=1 SV=2 - [K1C23_HUMAN] |
| Q7Z3Z0 | Keratin, type I cytoskeletal 25 OS=Homo sapiens GN=KRT25 PE=1 SV=1 - [K1C25_HUMAN] |
| P35527 | Keratin, type I cytoskeletal 9 OS=Homo sapiens GN=KRT9 PE=1 SV=3 - [K1C9_HUMAN] |
| P04264 | Keratin, type II cytoskeletal 1 OS=Homo sapiens GN=KRT1 PE=1 SV=6 - [K2C1_HUMAN] |
| Q7Z794 | Keratin, type II cytoskeletal 1b OS=Homo sapiens GN=KRT77 PE=2 SV=3 - [K2C1B_HUMAN] |
| P35908 | Keratin, type II cytoskeletal 2 epidermal OS=Homo sapiens GN=KRT2 PE=1 SV=2 - [K22E_HUMAN] |
| P13647 | Keratin, type II cytoskeletal 5 OS=Homo sapiens GN=KRT5 PE=1 SV=3 - [K2C5_HUMAN] |
| P43628 | Killer cell immunoglobulin-like receptor 2DL3 OS=Homo sapiens GN=KIR2DL3 PE=1 SV=1 - [KI2L3_HUMAN] |
| Q14943 | Killer cell immunoglobulin-like receptor 3DS1 OS=Homo sapiens GN=KIR3DS1 PE=2 SV=1 - [KI3S1_HUMAN] |
| Q9ULH0 | Kinase D-interacting substrate of 220 kDa OS=Homo sapiens GN=KIDINS220 PE=1 SV=3 - [KDIS_HUMAN] |
| Q8IVT5 | Kinase suppressor of Ras 1 OS=Homo sapiens GN=KSR1 PE=1 SV=3 - [KSR1_HUMAN] |
| Q6VAB6 | Kinase suppressor of Ras 2 OS=Homo sapiens GN=KSR2 PE=1 SV=2 - [KSR2_HUMAN] |
| Q12840 | Kinesin heavy chain isoform 5A OS=Homo sapiens GN=KIF5A PE=1 SV=2 - [KIF5A_HUMAN] |
| O60282 | Kinesin heavy chain isoform 5C OS=Homo sapiens GN=KIF5C PE=1 SV=1 - [KIF5C_HUMAN] |
| P33176 | Kinesin-1 heavy chain OS=Homo sapiens GN=KIF5B PE=1 SV=1 - [KINH_HUMAN] |
| P52732 | Kinesin-like protein KIF11 OS=Homo sapiens GN=KIF11 PE=1 SV=2 - [KIF11_HUMAN] |
| Q96FN5 | Kinesin-like protein KIF12 OS=Homo sapiens GN=KIF12 PE=1 SV=3 - [KIF12_HUMAN] |
| Q9NQT8 | Kinesin-like protein KIF13B OS=Homo sapiens GN=KIF13B PE=1 SV=2 - [KI13B_HUMAN] |
| Q15058 | Kinesin-like protein KIF14 OS=Homo sapiens GN=KIF14 PE=1 SV=1 - [KIF14_HUMAN] |
| Q9NS87 | Kinesin-like protein KIF15 OS=Homo sapiens GN=KIF15 PE=1 SV=1 - [KIF15_HUMAN] |
| Q8NI77 | Kinesin-like protein KIF18A OS=Homo sapiens GN=KIF18A PE=1 SV=2 - [KI18A_HUMAN] |
| Q86Y91 | Kinesin-like protein KIF18B OS=Homo sapiens GN=KIF18B PE=1 SV=3 - [KI18B_HUMAN] |
| Q12756 | Kinesin-like protein KIF1A OS=Homo sapiens GN=KIF1A PE=1 SV=2 - [KIF1A_HUMAN] |
| O60333 | Kinesin-like protein KIF1B OS=Homo sapiens GN=KIF1B PE=1 SV=5 - [KIF1B_HUMAN] |
| O43896 | Kinesin-like protein KIF1C OS=Homo sapiens GN=KIF1C PE=1 SV=3 - [KIF1C_HUMAN] |
| Q96Q89 | Kinesin-like protein KIF20B OS=Homo sapiens GN=KIF20B PE=1 SV=3 - [KI20B_HUMAN] |
| Q7Z4S6 | Kinesin-like protein KIF21A OS=Homo sapiens GN=KIF21A PE=1 SV=2 - [KI21A_HUMAN] |
| Q5T7B8 | Kinesin-like protein KIF24 OS=Homo sapiens GN=KIF24 PE=1 SV=2 - [KIF24_HUMAN] |
| Q9ULI4 | Kinesin-like protein KIF26A OS=Homo sapiens GN=KIF26A PE=2 SV=3 - [KI26A_HUMAN] |
| Q2KJY2 | Kinesin-like protein KIF26B OS=Homo sapiens GN=KIF26B PE=2 SV=1 - [KI26B_HUMAN] |
| Q86VH2 | Kinesin-like protein KIF27 OS=Homo sapiens GN=KIF27 PE=2 SV=1 - [KIF27_HUMAN] |
| Q6ZMV9 | Kinesin-like protein KIF6 OS=Homo sapiens GN=KIF6 PE=1 SV=3 - [KIF6_HUMAN] |
| Q9H410 | Kinetochore-associated protein DSN1 homolog OS=Homo sapiens GN=DSN1 PE=1 SV=2 - [DSN1_HUMAN] |
| P01042 | Kininogen-1 OS=Homo sapiens GN=KNG1 PE=1 SV=2 - [KNG1_HUMAN] |
| Q14678 | KN motif and ankyrin repeat domain-containing protein 1 OS=Homo sapiens GN=KANK1 PE=1 SV=3 - [KANK1_HUMAN] |
| Q63ZY3 | KN motif and ankyrin repeat domain-containing protein 2 OS=Homo sapiens GN=KANK2 PE=1 SV=1 - [KANK2_HUMAN] |
| Q8NCW0 | Kremen protein 2 OS=Homo sapiens GN=KREMEN2 PE=2 SV=1 - [KREM2_HUMAN] |
| Q13601 | KRR1 small subunit processome component homolog OS=Homo sapiens GN=KRR1 PE=1 SV=4 - [KRR1_HUMAN] |
| Q9Y4X4 | Krueppel-like factor 12 OS=Homo sapiens GN=KLF12 PE=1 SV=2 - [KLF12_HUMAN] |
| Q6PIL6 | Kv channel-interacting protein 4 OS=Homo sapiens GN=KCNIP4 PE=1 SV=1 - [KCIP4_HUMAN] |
| Q9BYG0 | Lactosylceramide 1,3-N-acetyl-beta-D-glucosaminyltransferase OS=Homo sapiens GN=B3GNT5 PE=1 SV=1 - [B3GN5_HUMAN] |
| P02788 | Lactotransferrin OS=Homo sapiens GN=LTF PE=1 SV=6 - [TRFL_HUMAN] |
| Q16363 | Laminin subunit alpha-4 OS=Homo sapiens GN=LAMA4 PE=1 SV=4 - [LAMA4_HUMAN] |
| O15230 | Laminin subunit alpha-5 OS=Homo sapiens GN=LAMA5 PE=1 SV=8 - [LAMA5_HUMAN] |
| P55268 | Laminin subunit beta-2 OS=Homo sapiens GN=LAMB2 PE=1 SV=2 - [LAMB2_HUMAN] |
| A4D0S4 | Laminin subunit beta-4 OS=Homo sapiens GN=LAMB4 PE=2 SV=1 - [LAMB4_HUMAN] |
| Q6PKG0 | La-related protein 1 OS=Homo sapiens GN=LARP1 PE=1 SV=2 - [LARP1_HUMAN] |
| Q71RC2 | La-related protein 4 OS=Homo sapiens GN=LARP4 PE=1 SV=3 - [LARP4_HUMAN] |
| Q9BRS8 | La-related protein 6 OS=Homo sapiens GN=LARP6 PE=1 SV=1 - [LARP6_HUMAN] |
| Q14696 | LDLR chaperone MESD OS=Homo sapiens GN=MESDC2 PE=1 SV=2 - [MESD_HUMAN] |
| O75610 | Left-right determination factor 1 OS=Homo sapiens GN=LEFTY1 PE=2 SV=1 - [LFTY1_HUMAN] |
| Q68G75 | LEM domain-containing protein 1 OS=Homo sapiens GN=LEMD1 PE=2 SV=2 - [LEMD1_HUMAN] |
| Q8NC56 | LEM domain-containing protein 2 OS=Homo sapiens GN=LEMD2 PE=1 SV=1 - [LEMD2_HUMAN] |
| Q15334 | Lethal(2) giant larvae protein homolog 1 OS=Homo sapiens GN=LLGL1 PE=1 SV=3 - [L2GL1_HUMAN] |
| Q2VYF4 | LETM1 domain-containing protein LETM2, mitochondrial OS=Homo sapiens GN=LETM2 PE=2 SV=2 - [LETM2_HUMAN] |
| Q86V48 | Leucine zipper protein 1 OS=Homo sapiens GN=LUZP1 PE=1 SV=2 - [LUZP1_HUMAN] |
| Q9P127 | Leucine zipper protein 4 OS=Homo sapiens GN=LUZP4 PE=1 SV=1 - [LUZP4_HUMAN] |
| Q9Y250 | Leucine zipper putative tumor suppressor 1 OS=Homo sapiens GN=LZTS1 PE=1 SV=3 - [LZTS1_HUMAN] |
| Q6ZMV7 | Leucine-, glutamate- and lysine-rich protein 1 OS=Homo sapiens GN=LEKR1 PE=2 SV=2 - [LEKR1_HUMAN] |
| P42704 | Leucine-rich PPR motif-containing protein, mitochondrial OS=Homo sapiens GN=LRPPRC PE=1 SV=3 - [LPPRC_HUMAN] |
| Q9C099 | Leucine-rich repeat and coiled-coil domain-containing protein 1 OS=Homo sapiens GN=LRRCC1 PE=1 SV=2 - [LRCC1_HUMAN] |
| Q9BTN0 | Leucine-rich repeat and fibronectin type-III domain-containing protein 3 OS=Homo sapiens GN=LRFN3 PE=2 SV=1 - [LRFN3_HUMAN] |
| Q9Y608 | Leucine-rich repeat flightless-interacting protein 2 OS=Homo sapiens GN=LRRFIP2 PE=1 SV=1 - [LRRF2_HUMAN] |
| O75325 | Leucine-rich repeat neuronal protein 2 OS=Homo sapiens GN=LRRN2 PE=2 SV=2 - [LRRN2_HUMAN] |
| Q38SD2 | Leucine-rich repeat serine/threonine-protein kinase 1 OS=Homo sapiens GN=LRRK1 PE=1 SV=3 - [LRRK1_HUMAN] |
| Q5S007 | Leucine-rich repeat serine/threonine-protein kinase 2 OS=Homo sapiens GN=LRRK2 PE=1 SV=2 - [LRRK2_HUMAN] |
| O43300 | Leucine-rich repeat transmembrane neuronal protein 2 OS=Homo sapiens GN=LRRTM2 PE=2 SV=3 - [LRRT2_HUMAN] |
| Q86VH4 | Leucine-rich repeat transmembrane neuronal protein 4 OS=Homo sapiens GN=LRRTM4 PE=2 SV=2 - [LRRT4_HUMAN] |
| Q9P2V4 | Leucine-rich repeat, immunoglobulin-like domain and transmembrane domain-containing protein 1 OS=Homo sapiens GN=LRIT1 PE=2 SV=1 - [LRIT1_HUMAN] |
| A6NDA9 | Leucine-rich repeat, immunoglobulin-like domain and transmembrane domain-containing protein 2 OS=Homo sapiens GN=LRIT2 PE=2 SV=1 - [LRIT2_HUMAN] |
| O75473 | Leucine-rich repeat-containing G-protein coupled receptor 5 OS=Homo sapiens GN=LGR5 PE=1 SV=1 - [LGR5_HUMAN] |
| A6NIK2 | Leucine-rich repeat-containing protein 10B OS=Homo sapiens GN=LRRC10B PE=4 SV=2 - [LR10B_HUMAN] |
| Q6F5E8 | Leucine-rich repeat-containing protein 16C OS=Homo sapiens GN=RLTPR PE=1 SV=2 - [LR16C_HUMAN] |
| O60309 | Leucine-rich repeat-containing protein 37A3 OS=Homo sapiens GN=LRRC37A3 PE=2 SV=2 - [L37A3_HUMAN] |
| Q9NT99 | Leucine-rich repeat-containing protein 4B OS=Homo sapiens GN=LRRC4B PE=2 SV=3 - [LRC4B_HUMAN] |
| Q96CX6 | Leucine-rich repeat-containing protein 58 OS=Homo sapiens GN=LRRC58 PE=1 SV=2 - [LRC58_HUMAN] |
| Q05C16 | Leucine-rich repeat-containing protein 63 OS=Homo sapiens GN=LRRC63 PE=2 SV=2 - [LRC63_HUMAN] |
| Q5JTD7 | Leucine-rich repeat-containing protein 73 OS=Homo sapiens GN=LRRC73 PE=2 SV=1 - [LRC73_HUMAN] |
| Q0VAA2 | Leucine-rich repeat-containing protein 74A OS=Homo sapiens GN=LRRC74A PE=2 SV=2 - [LR74A_HUMAN] |
| Q96JA1 | Leucine-rich repeats and immunoglobulin-like domains protein 1 OS=Homo sapiens GN=LRIG1 PE=1 SV=2 - [LRIG1_HUMAN] |
| O75023 | Leukocyte immunoglobulin-like receptor subfamily B member 5 OS=Homo sapiens GN=LILRB5 PE=1 SV=1 - [LIRB5_HUMAN] |
| Q96BZ8 | Leukocyte receptor cluster member 1 OS=Homo sapiens GN=LENG1 PE=1 SV=1 - [LENG1_HUMAN] |
| Q96PV6 | Leukocyte receptor cluster member 8 OS=Homo sapiens GN=LENG8 PE=1 SV=2 - [LENG8_HUMAN] |
| Q08722 | Leukocyte surface antigen CD47 OS=Homo sapiens GN=CD47 PE=1 SV=1 - [CD47_HUMAN] |
| P29376 | Leukocyte tyrosine kinase receptor OS=Homo sapiens GN=LTK PE=1 SV=3 - [LTK_HUMAN] |
| Q8N3X6 | Ligand-dependent nuclear receptor corepressor-like protein OS=Homo sapiens GN=LCORL PE=1 SV=4 - [LCORL_HUMAN] |
| Q9NZU5 | LIM and cysteine-rich domains protein 1 OS=Homo sapiens GN=LMCD1 PE=1 SV=1 - [LMCD1_HUMAN] |
| Q8WWI1 | LIM domain only protein 7 OS=Homo sapiens GN=LMO7 PE=1 SV=3 - [LMO7_HUMAN] |
| Q8WVP7 | Limb region 1 protein homolog OS=Homo sapiens GN=LMBR1 PE=1 SV=1 - [LMBR1_HUMAN] |
| O00370 | LINE-1 retrotransposable element ORF2 protein OS=Homo sapiens PE=1 SV=1 - [LORF2_HUMAN] |
| Q96GM1 | Lipid phosphate phosphatase-related protein type 2 OS=Homo sapiens GN=LPPR2 PE=2 SV=1 - [LPPR2_HUMAN] |
| Q7Z2D5 | Lipid phosphate phosphatase-related protein type 4 OS=Homo sapiens GN=LPPR4 PE=1 SV=1 - [LPPR4_HUMAN] |
| O43688 | Lipid phosphate phosphohydrolase 2 OS=Homo sapiens GN=PPAP2C PE=1 SV=1 - [LPP2_HUMAN] |
| Q86UP9 | Lipoma HMGIC fusion partner-like 3 protein OS=Homo sapiens GN=LHFPL3 PE=2 SV=3 - [LHPL3_HUMAN] |
| P18428 | Lipopolysaccharide-binding protein OS=Homo sapiens GN=LBP PE=1 SV=3 - [LBP_HUMAN] |
| O75334 | Liprin-alpha-2 OS=Homo sapiens GN=PPFIA2 PE=1 SV=2 - [LIPA2_HUMAN] |
| O75145 | Liprin-alpha-3 OS=Homo sapiens GN=PPFIA3 PE=1 SV=3 - [LIPA3_HUMAN] |
| Q9Y2F5 | Little elongation complex subunit 1 OS=Homo sapiens GN=ICE1 PE=1 SV=5 - [ICE1_HUMAN] |
| Q8IVB5 | LIX1-like protein OS=Homo sapiens GN=LIX1L PE=2 SV=1 - [LIX1L_HUMAN] |
| P00338 | L-lactate dehydrogenase A chain OS=Homo sapiens GN=LDHA PE=1 SV=2 - [LDHA_HUMAN] |
| Q1L5Z9 | LON peptidase N-terminal domain and RING finger protein 2 OS=Homo sapiens GN=LONRF2 PE=2 SV=3 - [LONF2_HUMAN] |
| Q86WA8 | Lon protease homolog 2, peroxisomal OS=Homo sapiens GN=LONP2 PE=1 SV=1 - [LONP2_HUMAN] |
| P36776 | Lon protease homolog, mitochondrial OS=Homo sapiens GN=LONP1 PE=1 SV=2 - [LONM_HUMAN] |
| Q6PCB7 | Long-chain fatty acid transport protein 1 OS=Homo sapiens GN=SLC27A1 PE=2 SV=1 - [S27A1_HUMAN] |
| Q9Y2P4 | Long-chain fatty acid transport protein 6 OS=Homo sapiens GN=SLC27A6 PE=2 SV=1 - [S27A6_HUMAN] |
| Q96GR2 | Long-chain-fatty-acid--CoA ligase ACSBG1 OS=Homo sapiens GN=ACSBG1 PE=2 SV=2 - [ACBG1_HUMAN] |
| P12318 | Low affinity immunoglobulin gamma Fc region receptor II-a OS=Homo sapiens GN=FCGR2A PE=1 SV=4 - [FCG2A_HUMAN] |
| Q7Z4F1 | Low-density lipoprotein receptor-related protein 10 OS=Homo sapiens GN=LRP10 PE=1 SV=2 - [LRP10_HUMAN] |
| Q9NZR2 | Low-density lipoprotein receptor-related protein 1B OS=Homo sapiens GN=LRP1B PE=1 SV=2 - [LRP1B_HUMAN] |
| P98164 | Low-density lipoprotein receptor-related protein 2 OS=Homo sapiens GN=LRP2 PE=1 SV=3 - [LRP2_HUMAN] |
| O95232 | Luc7-like protein 3 OS=Homo sapiens GN=LUC7L3 PE=1 SV=2 - [LC7L3_HUMAN] |
| P05455 | Lupus La protein OS=Homo sapiens GN=SSB PE=1 SV=2 - [LA_HUMAN] |
| Q6UWN5 | Ly6/PLAUR domain-containing protein 5 OS=Homo sapiens GN=LYPD5 PE=1 SV=2 - [LYPD5_HUMAN] |
| Q8NI32 | Ly6/PLAUR domain-containing protein 6B OS=Homo sapiens GN=LYPD6B PE=2 SV=1 - [LPD6B_HUMAN] |
| O94772 | Lymphocyte antigen 6H OS=Homo sapiens GN=LY6H PE=1 SV=1 - [LY6H_HUMAN] |
| O60449 | Lymphocyte antigen 75 OS=Homo sapiens GN=LY75 PE=1 SV=3 - [LY75_HUMAN] |
| P47992 | Lymphotactin OS=Homo sapiens GN=XCL1 PE=1 SV=1 - [XCL1_HUMAN] |
| P46736 | Lys-63-specific deubiquitinase BRCC36 OS=Homo sapiens GN=BRCC3 PE=1 SV=2 - [BRCC3_HUMAN] |
| Q7LBC6 | Lysine-specific demethylase 3B OS=Homo sapiens GN=KDM3B PE=1 SV=2 - [KDM3B_HUMAN] |
| O94953 | Lysine-specific demethylase 4B OS=Homo sapiens GN=KDM4B PE=1 SV=4 - [KDM4B_HUMAN] |
| Q8N371 | Lysine-specific demethylase 8 OS=Homo sapiens GN=KDM8 PE=1 SV=1 - [KDM8_HUMAN] |
| Q99677 | Lysophosphatidic acid receptor 4 OS=Homo sapiens GN=LPAR4 PE=1 SV=1 - [LPAR4_HUMAN] |
| Q9H1C0 | Lysophosphatidic acid receptor 5 OS=Homo sapiens GN=LPAR5 PE=2 SV=1 - [LPAR5_HUMAN] |
| Q6ZNC8 | Lysophospholipid acyltransferase 1 OS=Homo sapiens GN=MBOAT1 PE=1 SV=1 - [MBOA1_HUMAN] |
| Q6ZP29 | Lysosomal amino acid transporter 1 homolog OS=Homo sapiens GN=PQLC2 PE=1 SV=1 - [LAAT1_HUMAN] |
| P42785 | Lysosomal Pro-X carboxypeptidase OS=Homo sapiens GN=PRCP PE=1 SV=1 - [PCP_HUMAN] |
| Q15012 | Lysosomal-associated transmembrane protein 4A OS=Homo sapiens GN=LAPTM4A PE=1 SV=1 - [LAP4A_HUMAN] |
| Q99698 | Lysosomal-trafficking regulator OS=Homo sapiens GN=LYST PE=1 SV=3 - [LYST_HUMAN] |
| P39900 | Macrophage metalloelastase OS=Homo sapiens GN=MMP12 PE=1 SV=1 - [MMP12_HUMAN] |
| Q9UEW3 | Macrophage receptor MARCO OS=Homo sapiens GN=MARCO PE=1 SV=1 - [MARCO_HUMAN] |
| P40121 | Macrophage-capping protein OS=Homo sapiens GN=CAPG PE=1 SV=2 - [CAPG_HUMAN] |
| Q8NDA8 | Maestro heat-like repeat-containing protein family member 1 OS=Homo sapiens GN=MROH1 PE=2 SV=3 - [MROH1_HUMAN] |
| Q7Z745 | Maestro heat-like repeat-containing protein family member 2B OS=Homo sapiens GN=MROH2B PE=2 SV=3 - [MRO2B_HUMAN] |
| Q9HD23 | Magnesium transporter MRS2 homolog, mitochondrial OS=Homo sapiens GN=MRS2 PE=1 SV=1 - [MRS2_HUMAN] |
| P40926 | Malate dehydrogenase, mitochondrial OS=Homo sapiens GN=MDH2 PE=1 SV=3 - [MDHM_HUMAN] |
| Q68DK7 | Male-specific lethal 1 homolog OS=Homo sapiens GN=MSL1 PE=1 SV=3 - [MSL1_HUMAN] |
| A6NHS7 | MANSC domain-containing protein 4 OS=Homo sapiens GN=MANSC4 PE=3 SV=3 - [MANS4_HUMAN] |
| Q8WXG6 | MAP kinase-activating death domain protein OS=Homo sapiens GN=MADD PE=1 SV=2 - [MADD_HUMAN] |
| P27448 | MAP/microtubule affinity-regulating kinase 3 OS=Homo sapiens GN=MARK3 PE=1 SV=4 - [MARK3_HUMAN] |
| Q3KQU3 | MAP7 domain-containing protein 1 OS=Homo sapiens GN=MAP7D1 PE=1 SV=1 - [MA7D1_HUMAN] |
| Q8IWC1 | MAP7 domain-containing protein 3 OS=Homo sapiens GN=MAP7D3 PE=1 SV=2 - [MA7D3_HUMAN] |
| Q96JK9 | Mastermind-like protein 3 OS=Homo sapiens GN=MAML3 PE=1 SV=4 - [MAML3_HUMAN] |
| O15232 | Matrilin-3 OS=Homo sapiens GN=MATN3 PE=1 SV=2 - [MATN3_HUMAN] |
| P51511 | Matrix metalloproteinase-15 OS=Homo sapiens GN=MMP15 PE=1 SV=1 - [MMP15_HUMAN] |
| O75900 | Matrix metalloproteinase-23 OS=Homo sapiens GN=MMP23A PE=1 SV=2 - [MMP23_HUMAN] |
| P14780 | Matrix metalloproteinase-9 OS=Homo sapiens GN=MMP9 PE=1 SV=3 - [MMP9_HUMAN] |
| Q8IWI9 | MAX gene-associated protein OS=Homo sapiens GN=MGA PE=1 SV=3 - [MGAP_HUMAN] |
| Q4G0Z9 | MCM domain-containing protein 2 OS=Homo sapiens GN=MCMDC2 PE=1 SV=3 - [MCMD2_HUMAN] |
| Q5HYA8 | Meckelin OS=Homo sapiens GN=TMEM67 PE=1 SV=2 - [MKS3_HUMAN] |
| Q15648 | Mediator of RNA polymerase II transcription subunit 1 OS=Homo sapiens GN=MED1 PE=1 SV=4 - [MED1_HUMAN] |
| Q86YW9 | Mediator of RNA polymerase II transcription subunit 12-like protein OS=Homo sapiens GN=MED12L PE=1 SV=2 - [MD12L_HUMAN] |
| O75448 | Mediator of RNA polymerase II transcription subunit 24 OS=Homo sapiens GN=MED24 PE=1 SV=1 - [MED24_HUMAN] |
| P42679 | Megakaryocyte-associated tyrosine-protein kinase OS=Homo sapiens GN=MATK PE=1 SV=1 - [MATK_HUMAN] |
| Q9Y4F3 | Meiosis arrest female protein 1 OS=Homo sapiens GN=KIAA0430 PE=1 SV=6 - [MARF1_HUMAN] |
| A0A087WXM9 | Meiosis-specific kinetochore protein OS=Homo sapiens GN=MEIKIN PE=2 SV=2 - [MEIKN_HUMAN] |
| Q5JRA6 | Melanoma inhibitory activity protein 3 OS=Homo sapiens GN=MIA3 PE=1 SV=1 - [MIA3_HUMAN] |
| Q9UNF1 | Melanoma-associated antigen D2 OS=Homo sapiens GN=MAGED2 PE=1 SV=2 - [MAGD2_HUMAN] |
| Q96QZ7 | Membrane-associated guanylate kinase, WW and PDZ domain-containing protein 1 OS=Homo sapiens GN=MAGI1 PE=1 SV=3 - [MAGI1_HUMAN] |
| Q86UL8 | Membrane-associated guanylate kinase, WW and PDZ domain-containing protein 2 OS=Homo sapiens GN=MAGI2 PE=1 SV=3 - [MAGI2_HUMAN] |
| Q14703 | Membrane-bound transcription factor site-1 protease OS=Homo sapiens GN=MBTPS1 PE=1 SV=1 - [MBTP1_HUMAN] |
| Q13421 | Mesothelin OS=Homo sapiens GN=MSLN PE=1 SV=2 - [MSLN_HUMAN] |
| Q13255 | Metabotropic glutamate receptor 1 OS=Homo sapiens GN=GRM1 PE=1 SV=3 - [GRM1_HUMAN] |
| Q14416 | Metabotropic glutamate receptor 2 OS=Homo sapiens GN=GRM2 PE=1 SV=2 - [GRM2_HUMAN] |
| O15303 | Metabotropic glutamate receptor 6 OS=Homo sapiens GN=GRM6 PE=1 SV=2 - [GRM6_HUMAN] |
| Q9UHE8 | Metalloreductase STEAP1 OS=Homo sapiens GN=STEAP1 PE=2 SV=1 - [STEA1_HUMAN] |
| Q6ZN28 | Metastasis-associated in colon cancer protein 1 OS=Homo sapiens GN=MACC1 PE=1 SV=2 - [MACC1_HUMAN] |
| O94776 | Metastasis-associated protein MTA2 OS=Homo sapiens GN=MTA2 PE=1 SV=1 - [MTA2_HUMAN] |
| P53582 | Methionine aminopeptidase 1 OS=Homo sapiens GN=METAP1 PE=1 SV=2 - [MAP11_HUMAN] |
| P50579 | Methionine aminopeptidase 2 OS=Homo sapiens GN=METAP2 PE=1 SV=1 - [MAP2_HUMAN] |
| Q9UBK8 | Methionine synthase reductase OS=Homo sapiens GN=MTRR PE=1 SV=3 - [MTRR_HUMAN] |
| Q8NHZ7 | Methyl-CpG-binding domain protein 3-like 2 OS=Homo sapiens GN=MBD3L2 PE=2 SV=3 - [MB3L2_HUMAN] |
| Q6N021 | Methylcytosine dioxygenase TET2 OS=Homo sapiens GN=TET2 PE=1 SV=3 - [TET2_HUMAN] |
| O43151 | Methylcytosine dioxygenase TET3 OS=Homo sapiens GN=TET3 PE=1 SV=3 - [TET3_HUMAN] |
| Q13825 | Methylglutaconyl-CoA hydratase, mitochondrial OS=Homo sapiens GN=AUH PE=1 SV=1 - [AUHM_HUMAN] |
| Q8IVH4 | Methylmalonic aciduria type A protein, mitochondrial OS=Homo sapiens GN=MMAA PE=1 SV=1 - [MMAA_HUMAN] |
| Q9BQA1 | Methylosome protein 50 OS=Homo sapiens GN=WDR77 PE=1 SV=1 - [MEP50_HUMAN] |
| Q9NX63 | MICOS complex subunit MIC19 OS=Homo sapiens GN=CHCHD3 PE=1 SV=1 - [MIC19_HUMAN] |
| Q8NEM0 | Microcephalin OS=Homo sapiens GN=MCPH1 PE=1 SV=3 - [MCPH1_HUMAN] |
| Q96EZ8 | Microspherule protein 1 OS=Homo sapiens GN=MCRS1 PE=1 SV=1 - [MCRS1_HUMAN] |
| Q9Y4B5 | Microtubule cross-linking factor 1 OS=Homo sapiens GN=MTCL1 PE=1 SV=5 - [MTCL1_HUMAN] |
| Q9UPN3 | Microtubule-actin cross-linking factor 1, isoforms 1/2/3/5 OS=Homo sapiens GN=MACF1 PE=1 SV=4 - [MACF1_HUMAN] |
| Q9P2G4 | Microtubule-associated protein 10 OS=Homo sapiens GN=MAP10 PE=1 SV=2 - [MAP10_HUMAN] |
| P78559 | Microtubule-associated protein 1A OS=Homo sapiens GN=MAP1A PE=1 SV=6 - [MAP1A_HUMAN] |
| Q96JE9 | Microtubule-associated protein 6 OS=Homo sapiens GN=MAP6 PE=1 SV=2 - [MAP6_HUMAN] |
| Q6P0Q8 | Microtubule-associated serine/threonine-protein kinase 2 OS=Homo sapiens GN=MAST2 PE=1 SV=2 - [MAST2_HUMAN] |
| O60307 | Microtubule-associated serine/threonine-protein kinase 3 OS=Homo sapiens GN=MAST3 PE=1 SV=2 - [MAST3_HUMAN] |
| Q9ULD2 | Microtubule-associated tumor suppressor 1 OS=Homo sapiens GN=MTUS1 PE=1 SV=2 - [MTUS1_HUMAN] |
| Q6P0N0 | Mis18-binding protein 1 OS=Homo sapiens GN=MIS18BP1 PE=1 SV=1 - [M18BP_HUMAN] |
| Q8TC71 | Mitochondria-eating protein OS=Homo sapiens GN=SPATA18 PE=1 SV=1 - [MIEAP_HUMAN] |
| Q7Z434 | Mitochondrial antiviral-signaling protein OS=Homo sapiens GN=MAVS PE=1 SV=2 - [MAVS_HUMAN] |
| Q96C03 | Mitochondrial dynamics protein MID49 OS=Homo sapiens GN=MIEF2 PE=1 SV=1 - [MID49_HUMAN] |
| Q9NQG6 | Mitochondrial dynamics protein MID51 OS=Homo sapiens GN=MIEF1 PE=1 SV=1 - [MID51_HUMAN] |
| Q7L5Y1 | Mitochondrial enolase superfamily member 1 OS=Homo sapiens GN=ENOSF1 PE=1 SV=1 - [ENOF1_HUMAN] |
| Q9UDX5 | Mitochondrial fission process protein 1 OS=Homo sapiens GN=MTFP1 PE=1 SV=1 - [MTFP1_HUMAN] |
| Q6P444 | Mitochondrial fission regulator 2 OS=Homo sapiens GN=MTFR2 PE=1 SV=2 - [MTFR2_HUMAN] |
| O14925 | Mitochondrial import inner membrane translocase subunit Tim23 OS=Homo sapiens GN=TIMM23 PE=1 SV=1 - [TIM23_HUMAN] |
| Q9BT17 | Mitochondrial ribosome-associated GTPase 1 OS=Homo sapiens GN=MTG1 PE=1 SV=2 - [MTG1_HUMAN] |
| O95140 | Mitofusin-2 OS=Homo sapiens GN=MFN2 PE=1 SV=3 - [MFN2_HUMAN] |
| Q13233 | Mitogen-activated protein kinase kinase kinase 1 OS=Homo sapiens GN=MAP3K1 PE=1 SV=4 - [M3K1_HUMAN] |
| Q02779 | Mitogen-activated protein kinase kinase kinase 10 OS=Homo sapiens GN=MAP3K10 PE=1 SV=3 - [M3K10_HUMAN] |
| Q16584 | Mitogen-activated protein kinase kinase kinase 11 OS=Homo sapiens GN=MAP3K11 PE=1 SV=1 - [M3K11_HUMAN] |
| Q99558 | Mitogen-activated protein kinase kinase kinase 14 OS=Homo sapiens GN=MAP3K14 PE=1 SV=2 - [M3K14_HUMAN] |
| Q56UN5 | Mitogen-activated protein kinase kinase kinase 19 OS=Homo sapiens GN=MAP3K19 PE=2 SV=1 - [M3K19_HUMAN] |
| Q99683 | Mitogen-activated protein kinase kinase kinase 5 OS=Homo sapiens GN=MAP3K5 PE=1 SV=1 - [M3K5_HUMAN] |
| Q12851 | Mitogen-activated protein kinase kinase kinase kinase 2 OS=Homo sapiens GN=MAP4K2 PE=1 SV=2 - [M4K2_HUMAN] |
| O95819 | Mitogen-activated protein kinase kinase kinase kinase 4 OS=Homo sapiens GN=MAP4K4 PE=1 SV=2 - [M4K4_HUMAN] |
| O60336 | Mitogen-activated protein kinase-binding protein 1 OS=Homo sapiens GN=MAPKBP1 PE=1 SV=4 - [MABP1_HUMAN] |
| O60566 | Mitotic checkpoint serine/threonine-protein kinase BUB1 beta OS=Homo sapiens GN=BUB1B PE=1 SV=3 - [BUB1B_HUMAN] |
| Q9Y6D9 | Mitotic spindle assembly checkpoint protein MAD1 OS=Homo sapiens GN=MAD1L1 PE=1 SV=2 - [MD1L1_HUMAN] |
| Q96BY2 | Modulator of apoptosis 1 OS=Homo sapiens GN=MOAP1 PE=1 SV=1 - [MOAP1_HUMAN] |
| O15374 | Monocarboxylate transporter 5 OS=Homo sapiens GN=SLC16A4 PE=2 SV=1 - [MOT5_HUMAN] |
| Q6UB35 | Monofunctional C1-tetrahydrofolate synthase, mitochondrial OS=Homo sapiens GN=MTHFD1L PE=1 SV=1 - [C1TM_HUMAN] |
| Q15014 | Mortality factor 4-like protein 2 OS=Homo sapiens GN=MORF4L2 PE=1 SV=1 - [MO4L2_HUMAN] |
| Q8NHP6 | Motile sperm domain-containing protein 2 OS=Homo sapiens GN=MOSPD2 PE=1 SV=1 - [MSPD2_HUMAN] |
| Q99549 | M-phase phosphoprotein 8 OS=Homo sapiens GN=MPHOSPH8 PE=1 SV=2 - [MPP8_HUMAN] |
| Q8WXI7 | Mucin-16 OS=Homo sapiens GN=MUC16 PE=1 SV=2 - [MUC16_HUMAN] |
| Q7Z5P9 | Mucin-19 OS=Homo sapiens GN=MUC19 PE=1 SV=2 - [MUC19_HUMAN] |
| Q02817 | Mucin-2 OS=Homo sapiens GN=MUC2 PE=1 SV=2 - [MUC2_HUMAN] |
| Q9H195 | Mucin-3B (Fragments) OS=Homo sapiens GN=MUC3B PE=2 SV=2 - [MUC3B_HUMAN] |
| P08183 | Multidrug resistance protein 1 OS=Homo sapiens GN=ABCB1 PE=1 SV=3 - [MDR1_HUMAN] |
| P33527 | Multidrug resistance-associated protein 1 OS=Homo sapiens GN=ABCC1 PE=1 SV=3 - [MRP1_HUMAN] |
| O95255 | Multidrug resistance-associated protein 6 OS=Homo sapiens GN=ABCC6 PE=1 SV=2 - [MRP6_HUMAN] |
| Q6DN12 | Multiple C2 and transmembrane domain-containing protein 2 OS=Homo sapiens GN=MCTP2 PE=1 SV=3 - [MCTP2_HUMAN] |
| Q96KG7 | Multiple epidermal growth factor-like domains protein 10 OS=Homo sapiens GN=MEGF10 PE=1 SV=1 - [MEG10_HUMAN] |
| O75970 | Multiple PDZ domain protein OS=Homo sapiens GN=MPDZ PE=1 SV=2 - [MPDZ_HUMAN] |
| P20309 | Muscarinic acetylcholine receptor M3 OS=Homo sapiens GN=CHRM3 PE=1 SV=1 - [ACM3_HUMAN] |
| Q9NUK0 | Muscleblind-like protein 3 OS=Homo sapiens GN=MBNL3 PE=1 SV=2 - [MBNL3_HUMAN] |
| O15457 | MutS protein homolog 4 OS=Homo sapiens GN=MSH4 PE=1 SV=2 - [MSH4_HUMAN] |
| Q9BQG0 | Myb-binding protein 1A OS=Homo sapiens GN=MYBBP1A PE=1 SV=2 - [MBB1A_HUMAN] |
| P10244 | Myb-related protein B OS=Homo sapiens GN=MYBL2 PE=1 SV=1 - [MYBB_HUMAN] |
| O00499 | Myc box-dependent-interacting protein 1 OS=Homo sapiens GN=BIN1 PE=1 SV=1 - [BIN1_HUMAN] |
| Q9NUJ1 | Mycophenolic acid acyl-glucuronide esterase, mitochondrial OS=Homo sapiens GN=ABHD10 PE=1 SV=1 - [ABHDA_HUMAN] |
| Q9Y2G1 | Myelin regulatory factor OS=Homo sapiens GN=MYRF PE=1 SV=3 - [MRF_HUMAN] |
| Q96LU7 | Myelin regulatory factor-like protein OS=Homo sapiens GN=MYRFL PE=2 SV=2 - [MRFL_HUMAN] |
| Q9UL68 | Myelin transcription factor 1-like protein OS=Homo sapiens GN=MYT1L PE=2 SV=3 - [MYT1L_HUMAN] |
| P05164 | Myeloperoxidase OS=Homo sapiens GN=MPO PE=1 SV=1 - [PERM_HUMAN] |
| Q8IZQ8 | Myocardin OS=Homo sapiens GN=MYOCD PE=1 SV=1 - [MYCD_HUMAN] |
| Q5VU43 | Myomegalin OS=Homo sapiens GN=PDE4DIP PE=1 SV=1 - [MYOME_HUMAN] |
| Q15746 | Myosin light chain kinase, smooth muscle OS=Homo sapiens GN=MYLK PE=1 SV=4 - [MYLK_HUMAN] |
| P60660 | Myosin light polypeptide 6 OS=Homo sapiens GN=MYL6 PE=1 SV=2 - [MYL6_HUMAN] |
| Q6WCQ1 | Myosin phosphatase Rho-interacting protein OS=Homo sapiens GN=MPRIP PE=1 SV=3 - [MPRIP_HUMAN] |
| P35580 | Myosin-10 OS=Homo sapiens GN=MYH10 PE=1 SV=3 - [MYH10_HUMAN] |
| Q9Y2K3 | Myosin-15 OS=Homo sapiens GN=MYH15 PE=1 SV=5 - [MYH15_HUMAN] |
| Q9Y623 | Myosin-4 OS=Homo sapiens GN=MYH4 PE=1 SV=2 - [MYH4_HUMAN] |
| A7E2Y1 | Myosin-7B OS=Homo sapiens GN=MYH7B PE=1 SV=3 - [MYH7B_HUMAN] |
| P13535 | Myosin-8 OS=Homo sapiens GN=MYH8 PE=1 SV=3 - [MYH8_HUMAN] |
| P35579 | Myosin-9 OS=Homo sapiens GN=MYH9 PE=1 SV=4 - [MYH9_HUMAN] |
| Q14896 | Myosin-binding protein C, cardiac-type OS=Homo sapiens GN=MYBPC3 PE=1 SV=4 - [MYPC3_HUMAN] |
| Q00872 | Myosin-binding protein C, slow-type OS=Homo sapiens GN=MYBPC1 PE=1 SV=2 - [MYPC1_HUMAN] |
| A2RUH7 | Myosin-binding protein H-like OS=Homo sapiens GN=MYBPHL PE=1 SV=2 - [MBPHL_HUMAN] |
| Q8NEV4 | Myosin-IIIa OS=Homo sapiens GN=MYO3A PE=2 SV=2 - [MYO3A_HUMAN] |
| Q9NXD2 | Myotubularin-related protein 10 OS=Homo sapiens GN=MTMR10 PE=1 SV=3 - [MTMRA_HUMAN] |
| A4FU01 | Myotubularin-related protein 11 OS=Homo sapiens GN=MTMR11 PE=2 SV=2 - [MTMRB_HUMAN] |
| Q9Y216 | Myotubularin-related protein 7 OS=Homo sapiens GN=MTMR7 PE=1 SV=3 - [MTMR7_HUMAN] |
| O94760 | N(G),N(G)-dimethylarginine dimethylaminohydrolase 1 OS=Homo sapiens GN=DDAH1 PE=1 SV=3 - [DDAH1_HUMAN] |
| Q15599 | Na(+)/H(+) exchange regulatory cofactor NHE-RF2 OS=Homo sapiens GN=SLC9A3R2 PE=1 SV=2 - [NHRF2_HUMAN] |
| Q76KP1 | N-acetyl-beta-glucosaminyl-glycoprotein 4-beta-N-acetylgalactosaminyltransferase 1 OS=Homo sapiens GN=B4GALNT4 PE=1 SV=1 - [B4GN4_HUMAN] |
| Q9UJ70 | N-acetyl-D-glucosamine kinase OS=Homo sapiens GN=NAGK PE=1 SV=4 - [NAGK_HUMAN] |
| Q9ULI1 | NACHT and WD repeat domain-containing protein 2 OS=Homo sapiens GN=NWD2 PE=2 SV=3 - [NWD2_HUMAN] |
| Q149M9 | NACHT domain- and WD repeat-containing protein 1 OS=Homo sapiens GN=NWD1 PE=1 SV=3 - [NWD1_HUMAN] |
| Q9C000 | NACHT, LRR and PYD domains-containing protein 1 OS=Homo sapiens GN=NLRP1 PE=1 SV=1 - [NALP1_HUMAN] |
| P59046 | NACHT, LRR and PYD domains-containing protein 12 OS=Homo sapiens GN=NLRP12 PE=1 SV=2 - [NAL12_HUMAN] |
| Q86W24 | NACHT, LRR and PYD domains-containing protein 14 OS=Homo sapiens GN=NLRP14 PE=1 SV=1 - [NAL14_HUMAN] |
| P59047 | NACHT, LRR and PYD domains-containing protein 5 OS=Homo sapiens GN=NLRP5 PE=2 SV=2 - [NALP5_HUMAN] |
| Q13423 | NAD(P) transhydrogenase, mitochondrial OS=Homo sapiens GN=NNT PE=1 SV=3 - [NNTM_HUMAN] |
| Q8IXJ6 | NAD-dependent protein deacetylase sirtuin-2 OS=Homo sapiens GN=SIRT2 PE=1 SV=2 - [SIR2_HUMAN] |
| Q9Y6E7 | NAD-dependent protein lipoamidase sirtuin-4, mitochondrial OS=Homo sapiens GN=SIRT4 PE=1 SV=1 - [SIR4_HUMAN] |
| O43674 | NADH dehydrogenase [ubiquinone] 1 beta subcomplex subunit 5, mitochondrial OS=Homo sapiens GN=NDUFB5 PE=1 SV=1 - [NDUB5_HUMAN] |
| O95139 | NADH dehydrogenase [ubiquinone] 1 beta subcomplex subunit 6 OS=Homo sapiens GN=NDUFB6 PE=1 SV=3 - [NDUB6_HUMAN] |
| Q7L592 | NADH dehydrogenase [ubiquinone] complex I, assembly factor 7 OS=Homo sapiens GN=NDUFAF7 PE=1 SV=1 - [NDUF7_HUMAN] |
| O43181 | NADH dehydrogenase [ubiquinone] iron-sulfur protein 4, mitochondrial OS=Homo sapiens GN=NDUFS4 PE=1 SV=1 - [NDUS4_HUMAN] |
| Q9UHQ9 | NADH-cytochrome b5 reductase 1 OS=Homo sapiens GN=CYB5R1 PE=1 SV=1 - [NB5R1_HUMAN] |
| P28331 | NADH-ubiquinone oxidoreductase 75 kDa subunit, mitochondrial OS=Homo sapiens GN=NDUFS1 PE=1 SV=3 - [NDUS1_HUMAN] |
| P03905 | NADH-ubiquinone oxidoreductase chain 4 OS=Homo sapiens GN=MT-ND4 PE=1 SV=1 - [NU4M_HUMAN] |
| P03915 | NADH-ubiquinone oxidoreductase chain 5 OS=Homo sapiens GN=MT-ND5 PE=1 SV=2 - [NU5M_HUMAN] |
| Q16798 | NADP-dependent malic enzyme, mitochondrial OS=Homo sapiens GN=ME3 PE=2 SV=2 - [MAON_HUMAN] |
| Q6NXP6 | NADP-dependent oxidoreductase domain-containing protein 1 OS=Homo sapiens GN=NOXRED1 PE=2 SV=2 - [NXRD1_HUMAN] |
| Q9Y5S8 | NADPH oxidase 1 OS=Homo sapiens GN=NOX1 PE=1 SV=2 - [NOX1_HUMAN] |
| Q6N069 | N-alpha-acetyltransferase 16, NatA auxiliary subunit OS=Homo sapiens GN=NAA16 PE=1 SV=2 - [NAA16_HUMAN] |
| P61599 | N-alpha-acetyltransferase 20 OS=Homo sapiens GN=NAA20 PE=1 SV=1 - [NAA20_HUMAN] |
| Q86UY6 | N-alpha-acetyltransferase 40 OS=Homo sapiens GN=NAA40 PE=1 SV=1 - [NAA40_HUMAN] |
| Q6T4R5 | Nance-Horan syndrome protein OS=Homo sapiens GN=NHS PE=1 SV=2 - [NHS_HUMAN] |
| Q14330 | N-arachidonyl glycine receptor OS=Homo sapiens GN=GPR18 PE=2 SV=2 - [GPR18_HUMAN] |
| E9PAV3 | Nascent polypeptide-associated complex subunit alpha, muscle-specific form OS=Homo sapiens GN=NACA PE=1 SV=1 - [NACAM_HUMAN] |
| O14513 | Nck-associated protein 5 OS=Homo sapiens GN=NCKAP5 PE=1 SV=2 - [NCKP5_HUMAN] |
| Q9HCH0 | Nck-associated protein 5-like OS=Homo sapiens GN=NCKAP5L PE=1 SV=2 - [NCK5L_HUMAN] |
| P20929 | Nebulin OS=Homo sapiens GN=NEB PE=1 SV=5 - [NEBU_HUMAN] |
| Q86VF7 | Nebulin-related-anchoring protein OS=Homo sapiens GN=NRAP PE=2 SV=2 - [NRAP_HUMAN] |
| Q9NQS3 | Nectin-3 OS=Homo sapiens GN=PVRL3 PE=1 SV=1 - [PVRL3_HUMAN] |
| Q86UW6 | NEDD4-binding protein 2 OS=Homo sapiens GN=N4BP2 PE=1 SV=2 - [N4BP2_HUMAN] |
| O00308 | NEDD4-like E3 ubiquitin-protein ligase WWP2 OS=Homo sapiens GN=WWP2 PE=1 SV=2 - [WWP2_HUMAN] |
| Q9Y5A7 | NEDD8 ultimate buster 1 OS=Homo sapiens GN=NUB1 PE=1 SV=2 - [NUB1_HUMAN] |
| Q9BU70 | Nef-associated protein 1 OS=Homo sapiens GN=C9orf156 PE=1 SV=2 - [NAP1_HUMAN] |
| P18615 | Negative elongation factor E OS=Homo sapiens GN=NELFE PE=1 SV=3 - [NELFE_HUMAN] |
| Q86YC3 | Negative regulator of reactive oxygen species OS=Homo sapiens GN=NRROS PE=1 SV=1 - [NRROS_HUMAN] |
| O60500 | Nephrin OS=Homo sapiens GN=NPHS1 PE=1 SV=1 - [NPHN_HUMAN] |
| Q7Z494 | Nephrocystin-3 OS=Homo sapiens GN=NPHP3 PE=1 SV=1 - [NPHP3_HUMAN] |
| Q8NF91 | Nesprin-1 OS=Homo sapiens GN=SYNE1 PE=1 SV=4 - [SYNE1_HUMAN] |
| Q8WXH0 | Nesprin-2 OS=Homo sapiens GN=SYNE2 PE=1 SV=3 - [SYNE2_HUMAN] |
| Q6ZMZ3 | Nesprin-3 OS=Homo sapiens GN=SYNE3 PE=1 SV=2 - [SYNE3_HUMAN] |
| P48681 | Nestin OS=Homo sapiens GN=NES PE=1 SV=2 - [NEST_HUMAN] |
| Q6ZN44 | Netrin receptor UNC5A OS=Homo sapiens GN=UNC5A PE=1 SV=3 - [UNC5A_HUMAN] |
| Q6UXZ4 | Netrin receptor UNC5D OS=Homo sapiens GN=UNC5D PE=2 SV=1 - [UNC5D_HUMAN] |
| Q8WUJ1 | Neuferricin OS=Homo sapiens GN=CYB5D2 PE=2 SV=1 - [NEUFC_HUMAN] |
| O15394 | Neural cell adhesion molecule 2 OS=Homo sapiens GN=NCAM2 PE=1 SV=2 - [NCAM2_HUMAN] |
| Q8NFP9 | Neurobeachin OS=Homo sapiens GN=NBEA PE=1 SV=3 - [NBEA_HUMAN] |
| Q09666 | Neuroblast differentiation-associated protein AHNAK OS=Homo sapiens GN=AHNAK PE=1 SV=2 - [AHNK_HUMAN] |
| A2RRP1 | Neuroblastoma-amplified sequence OS=Homo sapiens GN=NBAS PE=1 SV=2 - [NBAS_HUMAN] |
| P07196 | Neurofilament light polypeptide OS=Homo sapiens GN=NEFL PE=1 SV=3 - [NFL_HUMAN] |
| P07197 | Neurofilament medium polypeptide OS=Homo sapiens GN=NEFM PE=1 SV=3 - [NFM_HUMAN] |
| Q99466 | Neurogenic locus notch homolog protein 4 OS=Homo sapiens GN=NOTCH4 PE=1 SV=2 - [NOTC4_HUMAN] |
| Q9BYT8 | Neurolysin, mitochondrial OS=Homo sapiens GN=NLN PE=1 SV=1 - [NEUL_HUMAN] |
| P28336 | Neuromedin-B receptor OS=Homo sapiens GN=NMBR PE=1 SV=2 - [NMBR_HUMAN] |
| Q9GZQ4 | Neuromedin-U receptor 2 OS=Homo sapiens GN=NMUR2 PE=1 SV=2 - [NMUR2_HUMAN] |
| Q8NEY1 | Neuron navigator 1 OS=Homo sapiens GN=NAV1 PE=1 SV=2 - [NAV1_HUMAN] |
| Q8IVL1 | Neuron navigator 2 OS=Homo sapiens GN=NAV2 PE=1 SV=3 - [NAV2_HUMAN] |
| Q9Y5X5 | Neuropeptide FF receptor 2 OS=Homo sapiens GN=NPFFR2 PE=1 SV=2 - [NPFF2_HUMAN] |
| Q8TDF5 | Neuropilin and tolloid-like protein 1 OS=Homo sapiens GN=NETO1 PE=2 SV=2 - [NETO1_HUMAN] |
| P43007 | Neutral amino acid transporter A OS=Homo sapiens GN=SLC1A4 PE=1 SV=1 - [SATT_HUMAN] |
| Q6PIU2 | Neutral cholesterol ester hydrolase 1 OS=Homo sapiens GN=NCEH1 PE=1 SV=3 - [NCEH1_HUMAN] |
| P59665 | Neutrophil defensin 1 OS=Homo sapiens GN=DEFA1 PE=1 SV=1 - [DEF1_HUMAN] |
| Q8NI38 | NF-kappa-B inhibitor delta OS=Homo sapiens GN=NFKBID PE=1 SV=1 - [IKBD_HUMAN] |
| Q8N5F7 | NF-kappa-B-activating protein OS=Homo sapiens GN=NKAP PE=1 SV=1 - [NKAP_HUMAN] |
| Q96TA1 | Niban-like protein 1 OS=Homo sapiens GN=FAM129B PE=1 SV=3 - [NIBL1_HUMAN] |
| Q86XR2 | Niban-like protein 2 OS=Homo sapiens GN=FAM129C PE=1 SV=2 - [NIBL2_HUMAN] |
| P43490 | Nicotinamide phosphoribosyltransferase OS=Homo sapiens GN=NAMPT PE=1 SV=1 - [NAMPT_HUMAN] |
| Q9HAN9 | Nicotinamide/nicotinic acid mononucleotide adenylyltransferase 1 OS=Homo sapiens GN=NMNAT1 PE=1 SV=1 - [NMNA1_HUMAN] |
| Q14112 | Nidogen-2 OS=Homo sapiens GN=NID2 PE=1 SV=3 - [NID2_HUMAN] |
| Q7Z2Y5 | Nik-related protein kinase OS=Homo sapiens GN=NRK PE=1 SV=2 - [NRK_HUMAN] |
| Q9Y2I6 | Ninein-like protein OS=Homo sapiens GN=NINL PE=1 SV=2 - [NINL_HUMAN] |
| Q9H841 | NIPA-like protein 2 OS=Homo sapiens GN=NIPAL2 PE=2 SV=1 - [NPAL2_HUMAN] |
| Q9Y2I1 | Nischarin OS=Homo sapiens GN=NISCH PE=1 SV=3 - [NISCH_HUMAN] |
| P29475 | Nitric oxide synthase, brain OS=Homo sapiens GN=NOS1 PE=1 SV=2 - [NOS1_HUMAN] |
| P29474 | Nitric oxide synthase, endothelial OS=Homo sapiens GN=NOS3 PE=1 SV=3 - [NOS3_HUMAN] |
| Q12980 | Nitrogen permease regulator 3-like protein OS=Homo sapiens GN=NPRL3 PE=1 SV=1 - [NPRL3_HUMAN] |
| P30414 | NK-tumor recognition protein OS=Homo sapiens GN=NKTR PE=1 SV=2 - [NKTR_HUMAN] |
| Q9NPP4 | NLR family CARD domain-containing protein 4 OS=Homo sapiens GN=NLRC4 PE=1 SV=2 - [NLRC4_HUMAN] |
| Q9NRG4 | N-lysine methyltransferase SMYD2 OS=Homo sapiens GN=SMYD2 PE=1 SV=2 - [SMYD2_HUMAN] |
| Q8NDF8 | Non-canonical poly(A) RNA polymerase PAPD5 OS=Homo sapiens GN=PAPD5 PE=1 SV=2 - [PAPD5_HUMAN] |
| Q8IVI9 | Nostrin OS=Homo sapiens GN=NOSTRIN PE=1 SV=2 - [NOSTN_HUMAN] |
| O60285 | NUAK family SNF1-like kinase 1 OS=Homo sapiens GN=NUAK1 PE=1 SV=1 - [NUAK1_HUMAN] |
| P49321 | Nuclear autoantigenic sperm protein OS=Homo sapiens GN=NASP PE=1 SV=2 - [NASP_HUMAN] |
| Q8N9A8 | Nuclear envelope phosphatase-regulatory subunit 1 OS=Homo sapiens GN=CNEP1R1 PE=1 SV=1 - [NEPR1_HUMAN] |
| Q00653 | Nuclear factor NF-kappa-B p100 subunit OS=Homo sapiens GN=NFKB2 PE=1 SV=4 - [NFKB2_HUMAN] |
| P19838 | Nuclear factor NF-kappa-B p105 subunit OS=Homo sapiens GN=NFKB1 PE=1 SV=2 - [NFKB1_HUMAN] |
| Q6P4R8 | Nuclear factor related to kappa-B-binding protein OS=Homo sapiens GN=NFRKB PE=1 SV=2 - [NFRKB_HUMAN] |
| Q7Z417 | Nuclear fragile X mental retardation-interacting protein 2 OS=Homo sapiens GN=NUFIP2 PE=1 SV=1 - [NUFP2_HUMAN] |
| P57740 | Nuclear pore complex protein Nup107 OS=Homo sapiens GN=NUP107 PE=1 SV=1 - [NU107_HUMAN] |
| O75694 | Nuclear pore complex protein Nup155 OS=Homo sapiens GN=NUP155 PE=1 SV=1 - [NU155_HUMAN] |
| Q12769 | Nuclear pore complex protein Nup160 OS=Homo sapiens GN=NUP160 PE=1 SV=3 - [NU160_HUMAN] |
| P35658 | Nuclear pore complex protein Nup214 OS=Homo sapiens GN=NUP214 PE=1 SV=2 - [NU214_HUMAN] |
| Q8N1F7 | Nuclear pore complex protein Nup93 OS=Homo sapiens GN=NUP93 PE=1 SV=2 - [NUP93_HUMAN] |
| Q5VU65 | Nuclear pore membrane glycoprotein 210-like OS=Homo sapiens GN=NUP210L PE=2 SV=1 - [P210L_HUMAN] |
| Q9HCD5 | Nuclear receptor coactivator 5 OS=Homo sapiens GN=NCOA5 PE=1 SV=2 - [NCOA5_HUMAN] |
| Q9Y618 | Nuclear receptor corepressor 2 OS=Homo sapiens GN=NCOR2 PE=1 SV=2 - [NCOR2_HUMAN] |
| P20393 | Nuclear receptor subfamily 1 group D member 1 OS=Homo sapiens GN=NR1D1 PE=1 SV=1 - [NR1D1_HUMAN] |
| P22736 | Nuclear receptor subfamily 4 group A member 1 OS=Homo sapiens GN=NR4A1 PE=1 SV=1 - [NR4A1_HUMAN] |
| Q15406 | Nuclear receptor subfamily 6 group A member 1 OS=Homo sapiens GN=NR6A1 PE=1 SV=2 - [NR6A1_HUMAN] |
| Q9NSY0 | Nuclear receptor-binding protein 2 OS=Homo sapiens GN=NRBP2 PE=2 SV=2 - [NRBP2_HUMAN] |
| P48552 | Nuclear receptor-interacting protein 1 OS=Homo sapiens GN=NRIP1 PE=1 SV=2 - [NRIP1_HUMAN] |
| Q13823 | Nucleolar GTP-binding protein 2 OS=Homo sapiens GN=GNL2 PE=1 SV=1 - [NOG2_HUMAN] |
| Q5C9Z4 | Nucleolar MIF4G domain-containing protein 1 OS=Homo sapiens GN=NOM1 PE=1 SV=1 - [NOM1_HUMAN] |
| O60287 | Nucleolar pre-ribosomal-associated protein 1 OS=Homo sapiens GN=URB1 PE=1 SV=4 - [NPA1P_HUMAN] |
| Q9H8H0 | Nucleolar protein 11 OS=Homo sapiens GN=NOL11 PE=1 SV=1 - [NOL11_HUMAN] |
| P78316 | Nucleolar protein 14 OS=Homo sapiens GN=NOP14 PE=1 SV=3 - [NOP14_HUMAN] |
| Q76FK4 | Nucleolar protein 8 OS=Homo sapiens GN=NOL8 PE=1 SV=1 - [NOL8_HUMAN] |
| Q9NR30 | Nucleolar RNA helicase 2 OS=Homo sapiens GN=DDX21 PE=1 SV=5 - [DDX21_HUMAN] |
| P06748 | Nucleophosmin OS=Homo sapiens GN=NPM1 PE=1 SV=2 - [NPM_HUMAN] |
| Q5SRE5 | Nucleoporin NUP188 homolog OS=Homo sapiens GN=NUP188 PE=1 SV=1 - [NU188_HUMAN] |
| P12270 | Nucleoprotein TPR OS=Homo sapiens GN=TPR PE=1 SV=3 - [TPR_HUMAN] |
| A8MXV4 | Nucleoside diphosphate-linked moiety X motif 19, mitochondrial OS=Homo sapiens GN=NUDT19 PE=1 SV=1 - [NUD19_HUMAN] |
| Q12830 | Nucleosome-remodeling factor subunit BPTF OS=Homo sapiens GN=BPTF PE=1 SV=3 - [BPTF_HUMAN] |
| Q5VT03 | NUT family member 2D OS=Homo sapiens GN=NUTM2D PE=3 SV=2 - [NTM2D_HUMAN] |
| Q6UWF7 | NXPE family member 4 OS=Homo sapiens GN=NXPE4 PE=2 SV=1 - [NXPE4_HUMAN] |
| Q9BQ69 | O-acetyl-ADP-ribose deacetylase MACROD1 OS=Homo sapiens GN=MACROD1 PE=1 SV=2 - [MACD1_HUMAN] |
| Q5VST9 | Obscurin OS=Homo sapiens GN=OBSCN PE=1 SV=3 - [OBSCN_HUMAN] |
| Q6UWY5 | Olfactomedin-like protein 1 OS=Homo sapiens GN=OLFML1 PE=1 SV=2 - [OLFL1_HUMAN] |
| Q6IF99 | Olfactory receptor 10K2 OS=Homo sapiens GN=OR10K2 PE=3 SV=1 - [O10K2_HUMAN] |
| Q8NGN2 | Olfactory receptor 10S1 OS=Homo sapiens GN=OR10S1 PE=2 SV=2 - [O10S1_HUMAN] |
| Q8NGY1 | Olfactory receptor 10Z1 OS=Homo sapiens GN=OR10Z1 PE=3 SV=1 - [O10Z1_HUMAN] |
| Q8NGC8 | Olfactory receptor 11H7 OS=Homo sapiens GN=OR11H7 PE=3 SV=2 - [O11H7_HUMAN] |
| Q9UGF7 | Olfactory receptor 12D3 OS=Homo sapiens GN=OR12D3 PE=2 SV=1 - [O12D3_HUMAN] |
| Q96R54 | Olfactory receptor 14A2 OS=Homo sapiens GN=OR14A2 PE=3 SV=2 - [O14A2_HUMAN] |
| Q8WZA6 | Olfactory receptor 1E3 OS=Homo sapiens GN=OR1E3 PE=3 SV=2 - [OR1E3_HUMAN] |
| Q8NGE2 | Olfactory receptor 2AP1 OS=Homo sapiens GN=OR2AP1 PE=3 SV=1 - [O2AP1_HUMAN] |
| Q8NGZ4 | Olfactory receptor 2G3 OS=Homo sapiens GN=OR2G3 PE=2 SV=1 - [OR2G3_HUMAN] |
| O43869 | Olfactory receptor 2T1 OS=Homo sapiens GN=OR2T1 PE=3 SV=3 - [OR2T1_HUMAN] |
| P58180 | Olfactory receptor 4D2 OS=Homo sapiens GN=OR4D2 PE=2 SV=1 - [OR4D2_HUMAN] |
| Q8NGJ6 | Olfactory receptor 51A4 OS=Homo sapiens GN=OR51A4 PE=3 SV=1 - [O51A4_HUMAN] |
| Q9H340 | Olfactory receptor 51B6 OS=Homo sapiens GN=OR51B6 PE=3 SV=2 - [O51B6_HUMAN] |
| Q9H255 | Olfactory receptor 51E2 OS=Homo sapiens GN=OR51E2 PE=2 SV=1 - [O51E2_HUMAN] |
| Q8NGJ5 | Olfactory receptor 51L1 OS=Homo sapiens GN=OR51L1 PE=3 SV=1 - [O51L1_HUMAN] |
| Q9H2C8 | Olfactory receptor 51V1 OS=Homo sapiens GN=OR51V1 PE=3 SV=2 - [O51V1_HUMAN] |
| Q96RD2 | Olfactory receptor 52B2 OS=Homo sapiens GN=OR52B2 PE=2 SV=3 - [O52B2_HUMAN] |
| Q8NGK2 | Olfactory receptor 52B4 OS=Homo sapiens GN=OR52B4 PE=3 SV=2 - [O52B4_HUMAN] |
| Q8NGI2 | Olfactory receptor 52N4 OS=Homo sapiens GN=OR52N4 PE=2 SV=2 - [O52N4_HUMAN] |
| Q8NH54 | Olfactory receptor 56A3 OS=Homo sapiens GN=OR56A3 PE=3 SV=2 - [O56A3_HUMAN] |
| Q8NGL1 | Olfactory receptor 5D18 OS=Homo sapiens GN=OR5D18 PE=2 SV=1 - [OR5DI_HUMAN] |
| Q8NGV7 | Olfactory receptor 5H2 OS=Homo sapiens GN=OR5H2 PE=3 SV=3 - [OR5H2_HUMAN] |
| Q96RA2 | Olfactory receptor 7D2 OS=Homo sapiens GN=OR7D2 PE=2 SV=2 - [OR7D2_HUMAN] |
| Q8NGQ6 | Olfactory receptor 9I1 OS=Homo sapiens GN=OR9I1 PE=3 SV=1 - [OR9I1_HUMAN] |
| Q8TAK6 | Oligodendrocyte transcription factor 1 OS=Homo sapiens GN=OLIG1 PE=1 SV=2 - [OLIG1_HUMAN] |
| Q6U736 | Opsin-5 OS=Homo sapiens GN=OPN5 PE=1 SV=3 - [OPN5_HUMAN] |
| Q96CV9 | Optineurin OS=Homo sapiens GN=OPTN PE=1 SV=2 - [OPTN_HUMAN] |
| O75665 | Oral-facial-digital syndrome 1 protein OS=Homo sapiens GN=OFD1 PE=1 SV=1 - [OFD1_HUMAN] |
| O43613 | Orexin receptor type 1 OS=Homo sapiens GN=HCRTR1 PE=2 SV=2 - [OX1R_HUMAN] |
| P11926 | Ornithine decarboxylase OS=Homo sapiens GN=ODC1 PE=1 SV=2 - [DCOR_HUMAN] |
| Q9HC10 | Otoferlin OS=Homo sapiens GN=OTOF PE=1 SV=3 - [OTOF_HUMAN] |
| Q8TE49 | OTU domain-containing protein 7A OS=Homo sapiens GN=OTUD7A PE=1 SV=1 - [OTU7A_HUMAN] |
| Q9NX31 | Oxidative stress-responsive serine-rich protein 1 OS=Homo sapiens GN=OSER1 PE=2 SV=2 - [OSER1_HUMAN] |
| P56715 | Oxygen-regulated protein 1 OS=Homo sapiens GN=RP1 PE=1 SV=1 - [RP1_HUMAN] |
| P30559 | Oxytocin receptor OS=Homo sapiens GN=OXTR PE=2 SV=2 - [OXYR_HUMAN] |
| Q04671 | P protein OS=Homo sapiens GN=OCA2 PE=1 SV=2 - [P_HUMAN] |
| Q9UBL9 | P2X purinoceptor 2 OS=Homo sapiens GN=P2RX2 PE=1 SV=1 - [P2RX2_HUMAN] |
| Q93086 | P2X purinoceptor 5 OS=Homo sapiens GN=P2RX5 PE=2 SV=4 - [P2RX5_HUMAN] |
| Q58A45 | PAB-dependent poly(A)-specific ribonuclease subunit PAN3 OS=Homo sapiens GN=PAN3 PE=1 SV=3 - [PAN3_HUMAN] |
| O75182 | Paired amphipathic helix protein Sin3b OS=Homo sapiens GN=SIN3B PE=1 SV=2 - [SIN3B_HUMAN] |
| O43316 | Paired box protein Pax-4 OS=Homo sapiens GN=PAX4 PE=1 SV=1 - [PAX4_HUMAN] |
| P23759 | Paired box protein Pax-7 OS=Homo sapiens GN=PAX7 PE=1 SV=4 - [PAX7_HUMAN] |
| Q9C0B5 | Palmitoyltransferase ZDHHC5 OS=Homo sapiens GN=ZDHHC5 PE=1 SV=2 - [ZDHC5_HUMAN] |
| P20962 | Parathymosin OS=Homo sapiens GN=PTMS PE=1 SV=2 - [PTMS_HUMAN] |
| P01270 | Parathyroid hormone OS=Homo sapiens GN=PTH PE=1 SV=1 - [PTHY_HUMAN] |
| Q8TEW8 | Partitioning defective 3 homolog B OS=Homo sapiens GN=PARD3B PE=1 SV=2 - [PAR3L_HUMAN] |
| Q8TEW0 | Partitioning defective 3 homolog OS=Homo sapiens GN=PARD3 PE=1 SV=2 - [PARD3_HUMAN] |
| Q8N8W4 | Patatin-like phospholipase domain-containing protein 1 OS=Homo sapiens GN=PNPLA1 PE=1 SV=3 - [PLPL1_HUMAN] |
| Q9NWS1 | PCNA-interacting partner OS=Homo sapiens GN=PARPBP PE=1 SV=3 - [PARI_HUMAN] |
| O15018 | PDZ domain-containing protein 2 OS=Homo sapiens GN=PDZD2 PE=1 SV=4 - [PDZD2_HUMAN] |
| Q76G19 | PDZ domain-containing protein 4 OS=Homo sapiens GN=PDZD4 PE=1 SV=1 - [PDZD4_HUMAN] |
| Q8NEN9 | PDZ domain-containing protein 8 OS=Homo sapiens GN=PDZD8 PE=1 SV=1 - [PDZD8_HUMAN] |
| Q8TF65 | PDZ domain-containing protein GIPC2 OS=Homo sapiens GN=GIPC2 PE=1 SV=1 - [GIPC2_HUMAN] |
| Q6ZMN7 | PDZ domain-containing RING finger protein 4 OS=Homo sapiens GN=PDZRN4 PE=2 SV=3 - [PZRN4_HUMAN] |
| Q96RV3 | Pecanex-like protein 1 OS=Homo sapiens GN=PCNX PE=1 SV=2 - [PCX1_HUMAN] |
| Q9H6A9 | Pecanex-like protein 3 OS=Homo sapiens GN=PCNXL3 PE=1 SV=2 - [PCX3_HUMAN] |
| O43511 | Pendrin OS=Homo sapiens GN=SLC26A4 PE=1 SV=1 - [S26A4_HUMAN] |
| Q9UGC7 | Peptide chain release factor 1-like, mitochondrial OS=Homo sapiens GN=MTRF1L PE=1 SV=1 - [RF1ML_HUMAN] |
| P62937 | Peptidyl-prolyl cis-trans isomerase A OS=Homo sapiens GN=PPIA PE=1 SV=2 - [PPIA_HUMAN] |
| Q9Y680 | Peptidyl-prolyl cis-trans isomerase FKBP7 OS=Homo sapiens GN=FKBP7 PE=1 SV=1 - [FKBP7_HUMAN] |
| Q13427 | Peptidyl-prolyl cis-trans isomerase G OS=Homo sapiens GN=PPIG PE=1 SV=2 - [PPIG_HUMAN] |
| P55201 | Peregrin OS=Homo sapiens GN=BRPF1 PE=1 SV=2 - [BRPF1_HUMAN] |
| O95613 | Pericentrin OS=Homo sapiens GN=PCNT PE=1 SV=4 - [PCNT_HUMAN] |
| Q15154 | Pericentriolar material 1 protein OS=Homo sapiens GN=PCM1 PE=1 SV=4 - [PCM1_HUMAN] |
| Q15063 | Periostin OS=Homo sapiens GN=POSTN PE=1 SV=2 - [POSTN_HUMAN] |
| O14936 | Peripheral plasma membrane protein CASK OS=Homo sapiens GN=CASK PE=1 SV=3 - [CSKP_HUMAN] |
| O95153 | Peripheral-type benzodiazepine receptor-associated protein 1 OS=Homo sapiens GN=BZRAP1 PE=1 SV=2 - [RIMB1_HUMAN] |
| O60437 | Periplakin OS=Homo sapiens GN=PPL PE=1 SV=4 - [PEPL_HUMAN] |
| A1KZ92 | Peroxidasin-like protein OS=Homo sapiens GN=PXDNL PE=1 SV=3 - [PXDNL_HUMAN] |
| P32119 | Peroxiredoxin-2 OS=Homo sapiens GN=PRDX2 PE=1 SV=5 - [PRDX2_HUMAN] |
| O75192 | Peroxisomal membrane protein 11A OS=Homo sapiens GN=PEX11A PE=1 SV=1 - [PX11A_HUMAN] |
| Q9P0Z9 | Peroxisomal sarcosine oxidase OS=Homo sapiens GN=PIPOX PE=1 SV=2 - [SOX_HUMAN] |
| O43933 | Peroxisome biogenesis factor 1 OS=Homo sapiens GN=PEX1 PE=1 SV=1 - [PEX1_HUMAN] |
| O60683 | Peroxisome biogenesis factor 10 OS=Homo sapiens GN=PEX10 PE=1 SV=1 - [PEX10_HUMAN] |
| Q86YN6 | Peroxisome proliferator-activated receptor gamma coactivator 1-beta OS=Homo sapiens GN=PPARGC1B PE=1 SV=2 - [PRGC2_HUMAN] |
| P37231 | Peroxisome proliferator-activated receptor gamma OS=Homo sapiens GN=PPARG PE=1 SV=3 - [PPARG_HUMAN] |
| Q9H720 | PGAP2-interacting protein OS=Homo sapiens GN=CWH43 PE=2 SV=2 - [PG2IP_HUMAN] |
| Q9NYI0 | PH and SEC7 domain-containing protein 3 OS=Homo sapiens GN=PSD3 PE=1 SV=2 - [PSD3_HUMAN] |
| O43189 | PHD finger protein 1 OS=Homo sapiens GN=PHF1 PE=1 SV=3 - [PHF1_HUMAN] |
| Q96QT6 | PHD finger protein 12 OS=Homo sapiens GN=PHF12 PE=1 SV=2 - [PHF12_HUMAN] |
| O94880 | PHD finger protein 14 OS=Homo sapiens GN=PHF14 PE=1 SV=2 - [PHF14_HUMAN] |
| Q96EK2 | PHD finger protein 21B OS=Homo sapiens GN=PHF21B PE=2 SV=1 - [PF21B_HUMAN] |
| Q9NSD9 | Phenylalanine--tRNA ligase beta subunit OS=Homo sapiens GN=FARSB PE=1 SV=3 - [SYFB_HUMAN] |
| Q8WWQ0 | PH-interacting protein OS=Homo sapiens GN=PHIP PE=1 SV=2 - [PHIP_HUMAN] |
| Q5VZY2 | Phosphatidate phosphatase PPAPDC1A OS=Homo sapiens GN=PPAPDC1A PE=1 SV=2 - [PPC1A_HUMAN] |
| Q9Y2H2 | Phosphatidylinositide phosphatase SAC2 OS=Homo sapiens GN=INPP5F PE=1 SV=3 - [SAC2_HUMAN] |
| Q6XPS3 | Phosphatidylinositol 3,4,5-trisphosphate 3-phosphatase TPTE2 OS=Homo sapiens GN=TPTE2 PE=1 SV=2 - [TPTE2_HUMAN] |
| Q8TCU6 | Phosphatidylinositol 3,4,5-trisphosphate-dependent Rac exchanger 1 protein OS=Homo sapiens GN=PREX1 PE=1 SV=3 - [PREX1_HUMAN] |
| Q9BTU6 | Phosphatidylinositol 4-kinase type 2-alpha OS=Homo sapiens GN=PI4K2A PE=1 SV=1 - [P4K2A_HUMAN] |
| O00443 | Phosphatidylinositol 4-phosphate 3-kinase C2 domain-containing subunit alpha OS=Homo sapiens GN=PIK3C2A PE=1 SV=2 - [P3C2A_HUMAN] |
| O00750 | Phosphatidylinositol 4-phosphate 3-kinase C2 domain-containing subunit beta OS=Homo sapiens GN=PIK3C2B PE=1 SV=2 - [P3C2B_HUMAN] |
| O75747 | Phosphatidylinositol 4-phosphate 3-kinase C2 domain-containing subunit gamma OS=Homo sapiens GN=PIK3C2G PE=1 SV=3 - [P3C2G_HUMAN] |
| Q92535 | Phosphatidylinositol N-acetylglucosaminyltransferase subunit C OS=Homo sapiens GN=PIGC PE=2 SV=1 - [PIGC_HUMAN] |
| Q13492 | Phosphatidylinositol-binding clathrin assembly protein OS=Homo sapiens GN=PICALM PE=1 SV=2 - [PICAL_HUMAN] |
| Q7Z7B1 | Phosphatidylinositol-glycan biosynthesis class W protein OS=Homo sapiens GN=PIGW PE=1 SV=1 - [PIGW_HUMAN] |
| Q16822 | Phosphoenolpyruvate carboxykinase [GTP], mitochondrial OS=Homo sapiens GN=PCK2 PE=1 SV=3 - [PCKGM_HUMAN] |
| Q96G03 | Phosphoglucomutase-2 OS=Homo sapiens GN=PGM2 PE=1 SV=4 - [PGM2_HUMAN] |
| Q15124 | Phosphoglucomutase-like protein 5 OS=Homo sapiens GN=PGM5 PE=1 SV=2 - [PGM5_HUMAN] |
| P00558 | Phosphoglycerate kinase 1 OS=Homo sapiens GN=PGK1 PE=1 SV=3 - [PGK1_HUMAN] |
| Q6ZUJ8 | Phosphoinositide 3-kinase adapter protein 1 OS=Homo sapiens GN=PIK3AP1 PE=1 SV=2 - [BCAP_HUMAN] |
| Q99570 | Phosphoinositide 3-kinase regulatory subunit 4 OS=Homo sapiens GN=PIK3R4 PE=1 SV=3 - [PI3R4_HUMAN] |
| Q5UE93 | Phosphoinositide 3-kinase regulatory subunit 6 OS=Homo sapiens GN=PIK3R6 PE=1 SV=1 - [PI3R6_HUMAN] |
| Q8WU67 | Phospholipase ABHD3 OS=Homo sapiens GN=ABHD3 PE=1 SV=2 - [ABHD3_HUMAN] |
| Q6P1J6 | Phospholipase B1, membrane-associated OS=Homo sapiens GN=PLB1 PE=1 SV=3 - [PLB1_HUMAN] |
| P55058 | Phospholipid transfer protein OS=Homo sapiens GN=PLTP PE=1 SV=1 - [PLTP_HUMAN] |
| O43520 | Phospholipid-transporting ATPase IC OS=Homo sapiens GN=ATP8B1 PE=1 SV=3 - [AT8B1_HUMAN] |
| P98198 | Phospholipid-transporting ATPase ID OS=Homo sapiens GN=ATP8B2 PE=1 SV=2 - [AT8B2_HUMAN] |
| Q8NB49 | Phospholipid-transporting ATPase IG OS=Homo sapiens GN=ATP11C PE=1 SV=3 - [AT11C_HUMAN] |
| O15305 | Phosphomannomutase 2 OS=Homo sapiens GN=PMM2 PE=1 SV=1 - [PMM2_HUMAN] |
| Q9HAB8 | Phosphopantothenate--cysteine ligase OS=Homo sapiens GN=PPCS PE=1 SV=2 - [PPCS_HUMAN] |
| O60256 | Phosphoribosyl pyrophosphate synthase-associated protein 2 OS=Homo sapiens GN=PRPSAP2 PE=1 SV=1 - [KPRB_HUMAN] |
| O15067 | Phosphoribosylformylglycinamidine synthase OS=Homo sapiens GN=PFAS PE=1 SV=4 - [PUR4_HUMAN] |
| P46019 | Phosphorylase b kinase regulatory subunit alpha, liver isoform OS=Homo sapiens GN=PHKA2 PE=1 SV=1 - [KPB2_HUMAN] |
| Q93100 | Phosphorylase b kinase regulatory subunit beta OS=Homo sapiens GN=PHKB PE=1 SV=3 - [KPBB_HUMAN] |
| Q6NYC8 | Phostensin OS=Homo sapiens GN=PPP1R18 PE=1 SV=1 - [PPR18_HUMAN] |
| Q9H5I5 | Piezo-type mechanosensitive ion channel component 2 OS=Homo sapiens GN=PIEZO2 PE=1 SV=2 - [PIEZ2_HUMAN] |
| Q8WWB5 | PIH1 domain-containing protein 2 OS=Homo sapiens GN=PIH1D2 PE=1 SV=1 - [PIHD2_HUMAN] |
| Q63HQ2 | Pikachurin OS=Homo sapiens GN=EGFLAM PE=1 SV=2 - [EGFLA_HUMAN] |
| O75364 | Pituitary homeobox 3 OS=Homo sapiens GN=PITX3 PE=1 SV=1 - [PITX3_HUMAN] |
| P28069 | Pituitary-specific positive transcription factor 1 OS=Homo sapiens GN=POU1F1 PE=1 SV=1 - [PIT1_HUMAN] |
| Q7Z3Z3 | Piwi-like protein 3 OS=Homo sapiens GN=PIWIL3 PE=2 SV=2 - [PIWL3_HUMAN] |
| Q7Z3Z4 | Piwi-like protein 4 OS=Homo sapiens GN=PIWIL4 PE=2 SV=2 - [PIWL4_HUMAN] |
| Q99959 | Plakophilin-2 OS=Homo sapiens GN=PKP2 PE=1 SV=2 - [PKP2_HUMAN] |
| P20020 | Plasma membrane calcium-transporting ATPase 1 OS=Homo sapiens GN=ATP2B1 PE=1 SV=3 - [AT2B1_HUMAN] |
| P05155 | Plasma protease C1 inhibitor OS=Homo sapiens GN=SERPING1 PE=1 SV=2 - [IC1_HUMAN] |
| P13797 | Plastin-3 OS=Homo sapiens GN=PLS3 PE=1 SV=4 - [PLST_HUMAN] |
| P16284 | Platelet endothelial cell adhesion molecule OS=Homo sapiens GN=PECAM1 PE=1 SV=1 - [PECA1_HUMAN] |
| Q9HB19 | Pleckstrin homology domain-containing family A member 2 OS=Homo sapiens GN=PLEKHA2 PE=1 SV=2 - [PKHA2_HUMAN] |
| Q9H7P9 | Pleckstrin homology domain-containing family G member 2 OS=Homo sapiens GN=PLEKHG2 PE=1 SV=3 - [PKHG2_HUMAN] |
| Q96PX9 | Pleckstrin homology domain-containing family G member 4B OS=Homo sapiens GN=PLEKHG4B PE=2 SV=4 - [PKH4B_HUMAN] |
| Q7Z736 | Pleckstrin homology domain-containing family H member 3 OS=Homo sapiens GN=PLEKHH3 PE=1 SV=2 - [PKHH3_HUMAN] |
| Q6ZWE6 | Pleckstrin homology domain-containing family M member 3 OS=Homo sapiens GN=PLEKHM3 PE=2 SV=2 - [PKHM3_HUMAN] |
| Q86UU1 | Pleckstrin homology-like domain family B member 1 OS=Homo sapiens GN=PHLDB1 PE=1 SV=1 - [PHLB1_HUMAN] |
| Q86SQ0 | Pleckstrin homology-like domain family B member 2 OS=Homo sapiens GN=PHLDB2 PE=1 SV=2 - [PHLB2_HUMAN] |
| Q15149 | Plectin OS=Homo sapiens GN=PLEC PE=1 SV=3 - [PLEC_HUMAN] |
| O75051 | Plexin-A2 OS=Homo sapiens GN=PLXNA2 PE=1 SV=4 - [PLXA2_HUMAN] |
| O60486 | Plexin-C1 OS=Homo sapiens GN=PLXNC1 PE=1 SV=1 - [PLXC1_HUMAN] |
| Q8NBT0 | POC1 centriolar protein homolog A OS=Homo sapiens GN=POC1A PE=1 SV=2 - [POC1A_HUMAN] |
| P09874 | Poly [ADP-ribose] polymerase 1 OS=Homo sapiens GN=PARP1 PE=1 SV=4 - [PARP1_HUMAN] |
| Q460N3 | Poly [ADP-ribose] polymerase 15 OS=Homo sapiens GN=PARP15 PE=1 SV=2 - [PAR15_HUMAN] |
| Q9BWT3 | Poly(A) polymerase gamma OS=Homo sapiens GN=PAPOLG PE=1 SV=2 - [PAPOG_HUMAN] |
| Q15365 | Poly(rC)-binding protein 1 OS=Homo sapiens GN=PCBP1 PE=1 SV=2 - [PCBP1_HUMAN] |
| Q9UHX1 | Poly(U)-binding-splicing factor PUF60 OS=Homo sapiens GN=PUF60 PE=1 SV=1 - [PUF60_HUMAN] |
| Q9H361 | Polyadenylate-binding protein 3 OS=Homo sapiens GN=PABPC3 PE=1 SV=2 - [PABP3_HUMAN] |
| Q96GD3 | Polycomb protein SCMH1 OS=Homo sapiens GN=SCMH1 PE=1 SV=1 - [SCMH1_HUMAN] |
| Q9NTG1 | Polycystic kidney disease and receptor for egg jelly-related protein OS=Homo sapiens GN=PKDREJ PE=2 SV=2 - [PKDRE_HUMAN] |
| Q7Z443 | Polycystic kidney disease protein 1-like 3 OS=Homo sapiens GN=PKD1L3 PE=1 SV=1 - [PK1L3_HUMAN] |
| P98161 | Polycystin-1 OS=Homo sapiens GN=PKD1 PE=1 SV=3 - [PKD1_HUMAN] |
| Q13563 | Polycystin-2 OS=Homo sapiens GN=PKD2 PE=1 SV=3 - [PKD2_HUMAN] |
| Q8IXK0 | Polyhomeotic-like protein 2 OS=Homo sapiens GN=PHC2 PE=1 SV=1 - [PHC2_HUMAN] |
| Q8IXK2 | Polypeptide N-acetylgalactosaminyltransferase 12 OS=Homo sapiens GN=GALNT12 PE=1 SV=3 - [GLT12_HUMAN] |
| Q8NCL4 | Polypeptide N-acetylgalactosaminyltransferase 6 OS=Homo sapiens GN=GALNT6 PE=2 SV=2 - [GALT6_HUMAN] |
| Q49A17 | Polypeptide N-acetylgalactosaminyltransferase-like 6 OS=Homo sapiens GN=GALNTL6 PE=2 SV=2 - [GLTL6_HUMAN] |
| Q9UKA9 | Polypyrimidine tract-binding protein 2 OS=Homo sapiens GN=PTBP2 PE=1 SV=1 - [PTBP2_HUMAN] |
| P0CG48 | Polyubiquitin-C OS=Homo sapiens GN=UBC PE=1 SV=3 - [UBC_HUMAN] |
| Q96KW2 | POM121-like protein 2 OS=Homo sapiens GN=POM121L2 PE=3 SV=2 - [P12L2_HUMAN] |
| P57789 | Potassium channel subfamily K member 10 OS=Homo sapiens GN=KCNK10 PE=1 SV=1 - [KCNKA_HUMAN] |
| Q96T55 | Potassium channel subfamily K member 16 OS=Homo sapiens GN=KCNK16 PE=1 SV=1 - [KCNKG_HUMAN] |
| O95279 | Potassium channel subfamily K member 5 OS=Homo sapiens GN=KCNK5 PE=1 SV=1 - [KCNK5_HUMAN] |
| A8MYU2 | Potassium channel subfamily U member 1 OS=Homo sapiens GN=KCNU1 PE=1 SV=2 - [KCNU1_HUMAN] |
| Q14721 | Potassium voltage-gated channel subfamily B member 1 OS=Homo sapiens GN=KCNB1 PE=1 SV=2 - [KCNB1_HUMAN] |
| Q03721 | Potassium voltage-gated channel subfamily C member 4 OS=Homo sapiens GN=KCNC4 PE=1 SV=2 - [KCNC4_HUMAN] |
| Q8TDN1 | Potassium voltage-gated channel subfamily G member 4 OS=Homo sapiens GN=KCNG4 PE=1 SV=1 - [KCNG4_HUMAN] |
| O95259 | Potassium voltage-gated channel subfamily H member 1 OS=Homo sapiens GN=KCNH1 PE=1 SV=1 - [KCNH1_HUMAN] |
| Q96L42 | Potassium voltage-gated channel subfamily H member 8 OS=Homo sapiens GN=KCNH8 PE=2 SV=2 - [KCNH8_HUMAN] |
| P51787 | Potassium voltage-gated channel subfamily KQT member 1 OS=Homo sapiens GN=KCNQ1 PE=1 SV=3 - [KCNQ1_HUMAN] |
| Q9Y3Q4 | Potassium/sodium hyperpolarization-activated cyclic nucleotide-gated channel 4 OS=Homo sapiens GN=HCN4 PE=1 SV=1 - [HCN4_HUMAN] |
| Q01860 | POU domain, class 5, transcription factor 1 OS=Homo sapiens GN=POU5F1 PE=1 SV=1 - [PO5F1_HUMAN] |
| Q14863 | POU domain, class 6, transcription factor 1 OS=Homo sapiens GN=POU6F1 PE=1 SV=1 - [PO6F1_HUMAN] |
| Q9H4Q3 | PR domain zinc finger protein 13 OS=Homo sapiens GN=PRDM13 PE=2 SV=2 - [PRD13_HUMAN] |
| Q9GZV8 | PR domain zinc finger protein 14 OS=Homo sapiens GN=PRDM14 PE=1 SV=1 - [PRD14_HUMAN] |
| Q13029 | PR domain zinc finger protein 2 OS=Homo sapiens GN=PRDM2 PE=1 SV=3 - [PRDM2_HUMAN] |
| P40425 | Pre-B-cell leukemia transcription factor 2 OS=Homo sapiens GN=PBX2 PE=1 SV=2 - [PBX2_HUMAN] |
| P20742 | Pregnancy zone protein OS=Homo sapiens GN=PZP PE=1 SV=4 - [PZP_HUMAN] |
| Q86UA1 | Pre-mRNA-processing factor 39 OS=Homo sapiens GN=PRPF39 PE=1 SV=3 - [PRP39_HUMAN] |
| O75400 | Pre-mRNA-processing factor 40 homolog A OS=Homo sapiens GN=PRPF40A PE=1 SV=2 - [PR40A_HUMAN] |
| O94906 | Pre-mRNA-processing factor 6 OS=Homo sapiens GN=PRPF6 PE=1 SV=1 - [PRP6_HUMAN] |
| Q9HCG8 | Pre-mRNA-splicing factor CWC22 homolog OS=Homo sapiens GN=CWC22 PE=1 SV=3 - [CWC22_HUMAN] |
| Q9NXE8 | Pre-mRNA-splicing factor CWC25 homolog OS=Homo sapiens GN=CWC25 PE=1 SV=1 - [CWC25_HUMAN] |
| Q15007 | Pre-mRNA-splicing regulator WTAP OS=Homo sapiens GN=WTAP PE=1 SV=2 - [FL2D_HUMAN] |
| Q5JRX3 | Presequence protease, mitochondrial OS=Homo sapiens GN=PITRM1 PE=1 SV=3 - [PREP_HUMAN] |
| O43900 | Prickle-like protein 3 OS=Homo sapiens GN=PRICKLE3 PE=1 SV=2 - [PRIC3_HUMAN] |
| Q6NUJ1 | Proactivator polypeptide-like 1 OS=Homo sapiens GN=PSAPL1 PE=2 SV=2 - [SAPL1_HUMAN] |
| A2PYH4 | Probable ATP-dependent DNA helicase HFM1 OS=Homo sapiens GN=HFM1 PE=1 SV=2 - [HFM1_HUMAN] |
| Q13206 | Probable ATP-dependent RNA helicase DDX10 OS=Homo sapiens GN=DDX10 PE=1 SV=2 - [DDX10_HUMAN] |
| Q9UJV9 | Probable ATP-dependent RNA helicase DDX41 OS=Homo sapiens GN=DDX41 PE=1 SV=2 - [DDX41_HUMAN] |
| Q9NXZ2 | Probable ATP-dependent RNA helicase DDX43 OS=Homo sapiens GN=DDX43 PE=2 SV=2 - [DDX43_HUMAN] |
| Q7L014 | Probable ATP-dependent RNA helicase DDX46 OS=Homo sapiens GN=DDX46 PE=1 SV=2 - [DDX46_HUMAN] |
| O95786 | Probable ATP-dependent RNA helicase DDX58 OS=Homo sapiens GN=DDX58 PE=1 SV=2 - [DDX58_HUMAN] |
| Q5T1V6 | Probable ATP-dependent RNA helicase DDX59 OS=Homo sapiens GN=DDX59 PE=1 SV=1 - [DDX59_HUMAN] |
| Q8IY21 | Probable ATP-dependent RNA helicase DDX60 OS=Homo sapiens GN=DDX60 PE=1 SV=3 - [DDX60_HUMAN] |
| Q9H7F0 | Probable cation-transporting ATPase 13A3 OS=Homo sapiens GN=ATP13A3 PE=1 SV=4 - [AT133_HUMAN] |
| Q6NUT2 | Probable C-mannosyltransferase DPY19L2 OS=Homo sapiens GN=DPY19L2 PE=1 SV=2 - [D19L2_HUMAN] |
| Q6ZPD9 | Probable C-mannosyltransferase DPY19L3 OS=Homo sapiens GN=DPY19L3 PE=2 SV=1 - [D19L3_HUMAN] |
| Q9HA77 | Probable cysteine--tRNA ligase, mitochondrial OS=Homo sapiens GN=CARS2 PE=1 SV=1 - [SYCM_HUMAN] |
| Q15751 | Probable E3 ubiquitin-protein ligase HERC1 OS=Homo sapiens GN=HERC1 PE=1 SV=2 - [HERC1_HUMAN] |
| P28370 | Probable global transcription activator SNF2L1 OS=Homo sapiens GN=SMARCA1 PE=1 SV=2 - [SMCA1_HUMAN] |
| Q7Z602 | Probable G-protein coupled receptor 141 OS=Homo sapiens GN=GPR141 PE=2 SV=1 - [GP141_HUMAN] |
| Q96CH1 | Probable G-protein coupled receptor 146 OS=Homo sapiens GN=GPR146 PE=2 SV=1 - [GP146_HUMAN] |
| Q86SP6 | Probable G-protein coupled receptor 149 OS=Homo sapiens GN=GPR149 PE=2 SV=2 - [GP149_HUMAN] |
| Q5T848 | Probable G-protein coupled receptor 158 OS=Homo sapiens GN=GPR158 PE=1 SV=1 - [GP158_HUMAN] |
| Q9UJ42 | Probable G-protein coupled receptor 160 OS=Homo sapiens GN=GPR160 PE=2 SV=1 - [GP160_HUMAN] |
| Q6PRD1 | Probable G-protein coupled receptor 179 OS=Homo sapiens GN=GPR179 PE=1 SV=2 - [GP179_HUMAN] |
| Q9BZJ8 | Probable G-protein coupled receptor 61 OS=Homo sapiens GN=GPR61 PE=2 SV=2 - [GPR61_HUMAN] |
| Q9BZJ7 | Probable G-protein coupled receptor 62 OS=Homo sapiens GN=GPR62 PE=2 SV=2 - [GPR62_HUMAN] |
| O95800 | Probable G-protein coupled receptor 75 OS=Homo sapiens GN=GPR75 PE=1 SV=1 - [GPR75_HUMAN] |
| Q7Z333 | Probable helicase senataxin OS=Homo sapiens GN=SETX PE=1 SV=4 - [SETX_HUMAN] |
| Q96NU7 | Probable imidazolonepropionase OS=Homo sapiens GN=AMDHD1 PE=1 SV=2 - [HUTI_HUMAN] |
| Q13395 | Probable methyltransferase TARBP1 OS=Homo sapiens GN=TARBP1 PE=1 SV=1 - [TARB1_HUMAN] |
| P0C7U3 | Probable palmitoyltransferase ZDHHC11B OS=Homo sapiens GN=ZDHHC11B PE=3 SV=1 - [ZH11B_HUMAN] |
| Q8IZN3 | Probable palmitoyltransferase ZDHHC14 OS=Homo sapiens GN=ZDHHC14 PE=1 SV=1 - [ZDH14_HUMAN] |
| Q9Y2G3 | Probable phospholipid-transporting ATPase IF OS=Homo sapiens GN=ATP11B PE=1 SV=2 - [AT11B_HUMAN] |
| P98196 | Probable phospholipid-transporting ATPase IH OS=Homo sapiens GN=ATP11A PE=1 SV=3 - [AT11A_HUMAN] |
| Q8WWH5 | Probable tRNA pseudouridine synthase 1 OS=Homo sapiens GN=TRUB1 PE=1 SV=1 - [TRUB1_HUMAN] |
| O95900 | Probable tRNA pseudouridine synthase 2 OS=Homo sapiens GN=TRUB2 PE=1 SV=1 - [TRUB2_HUMAN] |
| O95922 | Probable tubulin polyglutamylase TTLL1 OS=Homo sapiens GN=TTLL1 PE=2 SV=1 - [TTLL1_HUMAN] |
| Q3SXZ7 | Probable tubulin polyglutamylase TTLL9 OS=Homo sapiens GN=TTLL9 PE=2 SV=3 - [TTLL9_HUMAN] |
| P01133 | Pro-epidermal growth factor OS=Homo sapiens GN=EGF PE=1 SV=2 - [EGF_HUMAN] |
| P07737 | Profilin-1 OS=Homo sapiens GN=PFN1 PE=1 SV=2 - [PROF1_HUMAN] |
| P06401 | Progesterone receptor OS=Homo sapiens GN=PGR PE=1 SV=4 - [PRGR_HUMAN] |
| Q9BUL8 | Programmed cell death protein 10 OS=Homo sapiens GN=PDCD10 PE=1 SV=1 - [PDC10_HUMAN] |
| Q53EL6 | Programmed cell death protein 4 OS=Homo sapiens GN=PDCD4 PE=1 SV=2 - [PDCD4_HUMAN] |
| Q9HCJ1 | Progressive ankylosis protein homolog OS=Homo sapiens GN=ANKH PE=1 SV=2 - [ANKH_HUMAN] |
| P35232 | Prohibitin OS=Homo sapiens GN=PHB PE=1 SV=1 - [PHB_HUMAN] |
| Q99623 | Prohibitin-2 OS=Homo sapiens GN=PHB2 PE=1 SV=2 - [PHB2_HUMAN] |
| Q6PGN9 | Proline/serine-rich coiled-coil protein 1 OS=Homo sapiens GN=PSRC1 PE=1 SV=1 - [PSRC1_HUMAN] |
| Q96B36 | Proline-rich AKT1 substrate 1 OS=Homo sapiens GN=AKT1S1 PE=1 SV=1 - [AKTS1_HUMAN] |
| Q86XR5 | Proline-rich membrane anchor 1 OS=Homo sapiens GN=PRIMA1 PE=1 SV=2 - [PRIMA_HUMAN] |
| Q96HE9 | Proline-rich protein 11 OS=Homo sapiens GN=PRR11 PE=1 SV=1 - [PRR11_HUMAN] |
| Q9NZ81 | Proline-rich protein 13 OS=Homo sapiens GN=PRR13 PE=1 SV=1 - [PRR13_HUMAN] |
| A6NEV1 | Proline-rich protein 23A OS=Homo sapiens GN=PRR23A PE=3 SV=1 - [PR23A_HUMAN] |
| Q9H6K5 | Proline-rich protein 36 OS=Homo sapiens GN=PRR36 PE=2 SV=2 - [PRR36_HUMAN] |
| Q07954 | Prolow-density lipoprotein receptor-related protein 1 OS=Homo sapiens GN=LRP1 PE=1 SV=2 - [LRP1_HUMAN] |
| Q8IVL5 | Prolyl 3-hydroxylase 2 OS=Homo sapiens GN=LEPREL1 PE=1 SV=1 - [P3H2_HUMAN] |
| P13674 | Prolyl 4-hydroxylase subunit alpha-1 OS=Homo sapiens GN=P4HA1 PE=1 SV=2 - [P4HA1_HUMAN] |
| O43490 | Prominin-1 OS=Homo sapiens GN=PROM1 PE=1 SV=1 - [PROM1_HUMAN] |
| Q02297 | Pro-neuregulin-1, membrane-bound isoform OS=Homo sapiens GN=NRG1 PE=1 SV=3 - [NRG1_HUMAN] |
| P05165 | Propionyl-CoA carboxylase alpha chain, mitochondrial OS=Homo sapiens GN=PCCA PE=1 SV=4 - [PCCA_HUMAN] |
| Q8NBP7 | Proprotein convertase subtilisin/kexin type 9 OS=Homo sapiens GN=PCSK9 PE=1 SV=3 - [PCSK9_HUMAN] |
| P04808 | Prorelaxin H1 OS=Homo sapiens GN=RLN1 PE=2 SV=1 - [REL1_HUMAN] |
| O15354 | Prosaposin receptor GPR37 OS=Homo sapiens GN=GPR37 PE=1 SV=2 - [GPR37_HUMAN] |
| Q9H7Z7 | Prostaglandin E synthase 2 OS=Homo sapiens GN=PTGES2 PE=1 SV=1 - [PGES2_HUMAN] |
| P43115 | Prostaglandin E2 receptor EP3 subtype OS=Homo sapiens GN=PTGER3 PE=2 SV=1 - [PE2R3_HUMAN] |
| P35408 | Prostaglandin E2 receptor EP4 subtype OS=Homo sapiens GN=PTGER4 PE=1 SV=1 - [PE2R4_HUMAN] |
| Q16186 | Proteasomal ubiquitin receptor ADRM1 OS=Homo sapiens GN=ADRM1 PE=1 SV=2 - [ADRM1_HUMAN] |
| P25787 | Proteasome subunit alpha type-2 OS=Homo sapiens GN=PSMA2 PE=1 SV=2 - [PSA2_HUMAN] |
| Q5VYK3 | Proteasome-associated protein ECM29 homolog OS=Homo sapiens GN=ECM29 PE=1 SV=2 - [ECM29_HUMAN] |
| P55198 | Protein AF-17 OS=Homo sapiens GN=MLLT6 PE=1 SV=2 - [AF17_HUMAN] |
| Q8IVF2 | Protein AHNAK2 OS=Homo sapiens GN=AHNAK2 PE=1 SV=2 - [AHNK2_HUMAN] |
| Q5T1N1 | Protein AKNAD1 OS=Homo sapiens GN=AKNAD1 PE=2 SV=3 - [AKND1_HUMAN] |
| A6NFN9 | Protein ANKUB1 OS=Homo sapiens GN=ANKUB1 PE=2 SV=2 - [ANKUB_HUMAN] |
| Q9HCK5 | Protein argonaute-4 OS=Homo sapiens GN=AGO4 PE=1 SV=2 - [AGO4_HUMAN] |
| Q9UPA5 | Protein bassoon OS=Homo sapiens GN=BSN PE=2 SV=4 - [BSN_HUMAN] |
| Q96NH3 | Protein broad-minded OS=Homo sapiens GN=TBC1D32 PE=2 SV=4 - [BROMI_HUMAN] |
| Q8TCG1 | Protein CIP2A OS=Homo sapiens GN=KIAA1524 PE=1 SV=2 - [CIP2A_HUMAN] |
| Q9UBY8 | Protein CLN8 OS=Homo sapiens GN=CLN8 PE=1 SV=3 - [CLN8_HUMAN] |
| Q9P219 | Protein Daple OS=Homo sapiens GN=CCDC88C PE=1 SV=3 - [DAPLE_HUMAN] |
| Q9UBU7 | Protein DBF4 homolog A OS=Homo sapiens GN=DBF4 PE=1 SV=1 - [DBF4A_HUMAN] |
| Q5TDH0 | Protein DDI1 homolog 2 OS=Homo sapiens GN=DDI2 PE=1 SV=1 - [DDI2_HUMAN] |
| P35659 | Protein DEK OS=Homo sapiens GN=DEK PE=1 SV=1 - [DEK_HUMAN] |
| Q8NEG7 | Protein DENND6B OS=Homo sapiens GN=DENND6B PE=2 SV=1 - [DEN6B_HUMAN] |
| O60610 | Protein diaphanous homolog 1 OS=Homo sapiens GN=DIAPH1 PE=1 SV=2 - [DIAP1_HUMAN] |
| O60879 | Protein diaphanous homolog 2 OS=Homo sapiens GN=DIAPH2 PE=1 SV=1 - [DIAP2_HUMAN] |
| Q13087 | Protein disulfide-isomerase A2 OS=Homo sapiens GN=PDIA2 PE=1 SV=2 - [PDIA2_HUMAN] |
| Q14554 | Protein disulfide-isomerase A5 OS=Homo sapiens GN=PDIA5 PE=1 SV=1 - [PDIA5_HUMAN] |
| Q9Y3R5 | Protein dopey-2 OS=Homo sapiens GN=DOPEY2 PE=1 SV=5 - [DOP2_HUMAN] |
| Q9NYP3 | Protein downstream neighbor of Son OS=Homo sapiens GN=DONSON PE=1 SV=2 - [DONS_HUMAN] |
| Q14156 | Protein EFR3 homolog A OS=Homo sapiens GN=EFR3A PE=1 SV=2 - [EFR3A_HUMAN] |
| Q9Y2G0 | Protein EFR3 homolog B OS=Homo sapiens GN=EFR3B PE=1 SV=2 - [EFR3B_HUMAN] |
| Q03111 | Protein ENL OS=Homo sapiens GN=MLLT1 PE=1 SV=2 - [ENL_HUMAN] |
| Q5T1H1 | Protein eyes shut homolog OS=Homo sapiens GN=EYS PE=1 SV=5 - [EYS_HUMAN] |
| O95990 | Protein FAM107A OS=Homo sapiens GN=FAM107A PE=1 SV=1 - [F107A_HUMAN] |
| Q9BPY3 | Protein FAM118B OS=Homo sapiens GN=FAM118B PE=1 SV=1 - [F118B_HUMAN] |
| Q5BKY9 | Protein FAM133B OS=Homo sapiens GN=FAM133B PE=1 SV=1 - [F133B_HUMAN] |
| O94988 | Protein FAM13A OS=Homo sapiens GN=FAM13A PE=1 SV=2 - [FA13A_HUMAN] |
| A5PLN7 | Protein FAM149A OS=Homo sapiens GN=FAM149A PE=2 SV=2 - [F149A_HUMAN] |
| Q5T6X4 | Protein FAM162B OS=Homo sapiens GN=FAM162B PE=2 SV=1 - [F162B_HUMAN] |
| Q5VUB5 | Protein FAM171A1 OS=Homo sapiens GN=FAM171A1 PE=1 SV=1 - [F1711_HUMAN] |
| A8MVW0 | Protein FAM171A2 OS=Homo sapiens GN=FAM171A2 PE=1 SV=1 - [F1712_HUMAN] |
| Q8N128 | Protein FAM177A1 OS=Homo sapiens GN=FAM177A1 PE=1 SV=1 - [F177A_HUMAN] |
| Q6ZUX3 | Protein FAM179A OS=Homo sapiens GN=FAM179A PE=2 SV=2 - [F179A_HUMAN] |
| Q9Y4F4 | Protein FAM179B OS=Homo sapiens GN=FAM179B PE=1 SV=4 - [F179B_HUMAN] |
| Q8IYM0 | Protein FAM186B OS=Homo sapiens GN=FAM186B PE=2 SV=2 - [F186B_HUMAN] |
| Q96PV7 | Protein FAM193B OS=Homo sapiens GN=FAM193B PE=1 SV=3 - [F193B_HUMAN] |
| Q96KR6 | Protein FAM210B OS=Homo sapiens GN=FAM210B PE=1 SV=2 - [F210B_HUMAN] |
| Q32MH5 | Protein FAM214A OS=Homo sapiens GN=FAM214A PE=1 SV=2 - [F214A_HUMAN] |
| Q7Z4H9 | Protein FAM220A OS=Homo sapiens GN=FAM220A PE=2 SV=1 - [F220A_HUMAN] |
| Q8WU58 | Protein FAM222B OS=Homo sapiens GN=FAM222B PE=2 SV=1 - [F222B_HUMAN] |
| Q5JW98 | Protein FAM26D OS=Homo sapiens GN=FAM26D PE=2 SV=1 - [FA26D_HUMAN] |
| Q86V20 | Protein FAM35A OS=Homo sapiens GN=FAM35A PE=2 SV=1 - [FA35A_HUMAN] |
| Q96BQ1 | Protein FAM3D OS=Homo sapiens GN=FAM3D PE=1 SV=1 - [FAM3D_HUMAN] |
| Q96A09 | Protein FAM46B OS=Homo sapiens GN=FAM46B PE=1 SV=2 - [FA46B_HUMAN] |
| Q6ZV65 | Protein FAM47E OS=Homo sapiens GN=FAM47E PE=2 SV=3 - [FA47E_HUMAN] |
| Q8N5Q1 | Protein FAM71E2 OS=Homo sapiens GN=FAM71E2 PE=2 SV=3 - [F71E2_HUMAN] |
| Q96LP2 | Protein FAM81B OS=Homo sapiens GN=FAM81B PE=1 SV=3 - [FA81B_HUMAN] |
| Q96KN1 | Protein FAM84B OS=Homo sapiens GN=FAM84B PE=1 SV=1 - [FA84B_HUMAN] |
| Q68CZ1 | Protein fantom OS=Homo sapiens GN=RPGRIP1L PE=1 SV=2 - [FTM_HUMAN] |
| Q9UK73 | Protein fem-1 homolog B OS=Homo sapiens GN=FEM1B PE=1 SV=1 - [FEM1B_HUMAN] |
| Q5TBA9 | Protein furry homolog OS=Homo sapiens GN=FRY PE=1 SV=1 - [FRY_HUMAN] |
| O94915 | Protein furry homolog-like OS=Homo sapiens GN=FRYL PE=1 SV=2 - [FRYL_HUMAN] |
| Q96NT3 | Protein GUCD1 OS=Homo sapiens GN=GUCD1 PE=1 SV=2 - [GUCD1_HUMAN] |
| Q9BTY7 | Protein HGH1 homolog OS=Homo sapiens GN=HGH1 PE=1 SV=1 - [HGH1_HUMAN] |
| Q9NQC1 | Protein Jade-2 OS=Homo sapiens GN=JADE2 PE=1 SV=2 - [JADE2_HUMAN] |
| Q92613 | Protein Jade-3 OS=Homo sapiens GN=JADE3 PE=1 SV=1 - [JADE3_HUMAN] |
| Q92833 | Protein Jumonji OS=Homo sapiens GN=JARID2 PE=1 SV=2 - [JARD2_HUMAN] |
| Q04759 | Protein kinase C theta type OS=Homo sapiens GN=PRKCQ PE=1 SV=3 - [KPCT_HUMAN] |
| Q7Z429 | Protein lifeguard 1 OS=Homo sapiens GN=GRINA PE=2 SV=1 - [LFG1_HUMAN] |
| Q8N485 | Protein limb expression 1 homolog OS=Homo sapiens GN=LIX1 PE=2 SV=2 - [LIX1_HUMAN] |
| Q9H9Z2 | Protein lin-28 homolog A OS=Homo sapiens GN=LIN28A PE=1 SV=1 - [LN28A_HUMAN] |
| Q6UX01 | Protein LMBR1L OS=Homo sapiens GN=LMBR1L PE=1 SV=2 - [LMBRL_HUMAN] |
| Q7Z4T9 | Protein MAATS1 OS=Homo sapiens GN=MAATS1 PE=1 SV=2 - [MAAT1_HUMAN] |
| Q6ZRQ5 | Protein MMS22-like OS=Homo sapiens GN=MMS22L PE=1 SV=3 - [MMS22_HUMAN] |
| Q9P2P1 | Protein NYNRIN OS=Homo sapiens GN=NYNRIN PE=2 SV=3 - [NYNRI_HUMAN] |
| Q8NBL1 | Protein O-glucosyltransferase 1 OS=Homo sapiens GN=POGLUT1 PE=1 SV=1 - [PGLT1_HUMAN] |
| Q13438 | Protein OS-9 OS=Homo sapiens GN=OS9 PE=1 SV=1 - [OS9_HUMAN] |
| Q86TB9 | Protein PAT1 homolog 1 OS=Homo sapiens GN=PATL1 PE=1 SV=2 - [PATL1_HUMAN] |
| Q13635 | Protein patched homolog 1 OS=Homo sapiens GN=PTCH1 PE=1 SV=2 - [PTC1_HUMAN] |
| O14974 | Protein phosphatase 1 regulatory subunit 12A OS=Homo sapiens GN=PPP1R12A PE=1 SV=1 - [MYPT1_HUMAN] |
| Q9NXH3 | Protein phosphatase 1 regulatory subunit 14D OS=Homo sapiens GN=PPP1R14D PE=1 SV=1 - [PP14D_HUMAN] |
| O75807 | Protein phosphatase 1 regulatory subunit 15A OS=Homo sapiens GN=PPP1R15A PE=1 SV=1 - [PR15A_HUMAN] |
| Q5SWA1 | Protein phosphatase 1 regulatory subunit 15B OS=Homo sapiens GN=PPP1R15B PE=1 SV=1 - [PR15B_HUMAN] |
| Q96LQ0 | Protein phosphatase 1 regulatory subunit 36 OS=Homo sapiens GN=PPP1R36 PE=1 SV=1 - [PPR36_HUMAN] |
| Q9UQK1 | Protein phosphatase 1 regulatory subunit 3C OS=Homo sapiens GN=PPP1R3C PE=1 SV=2 - [PPR3C_HUMAN] |
| Q5JR12 | Protein phosphatase 1J OS=Homo sapiens GN=PPM1J PE=1 SV=1 - [PPM1J_HUMAN] |
| Q8WYL5 | Protein phosphatase Slingshot homolog 1 OS=Homo sapiens GN=SSH1 PE=1 SV=2 - [SSH1_HUMAN] |
| Q8TE77 | Protein phosphatase Slingshot homolog 3 OS=Homo sapiens GN=SSH3 PE=1 SV=2 - [SSH3_HUMAN] |
| P31949 | Protein S100-A11 OS=Homo sapiens GN=S100A11 PE=1 SV=2 - [S10AB_HUMAN] |
| P06703 | Protein S100-A6 OS=Homo sapiens GN=S100A6 PE=1 SV=1 - [S10A6_HUMAN] |
| P05109 | Protein S100-A8 OS=Homo sapiens GN=S100A8 PE=1 SV=1 - [S10A8_HUMAN] |
| P06702 | Protein S100-A9 OS=Homo sapiens GN=S100A9 PE=1 SV=1 - [S10A9_HUMAN] |
| P25815 | Protein S100-P OS=Homo sapiens GN=S100P PE=1 SV=2 - [S100P_HUMAN] |
| Q99590 | Protein SCAF11 OS=Homo sapiens GN=SCAF11 PE=1 SV=2 - [SCAFB_HUMAN] |
| Q8N9R8 | Protein SCAI OS=Homo sapiens GN=SCAI PE=1 SV=2 - [SCAI_HUMAN] |
| Q14160 | Protein scribble homolog OS=Homo sapiens GN=SCRIB PE=1 SV=4 - [SCRIB_HUMAN] |
| Q9UBV2 | Protein sel-1 homolog 1 OS=Homo sapiens GN=SEL1L PE=1 SV=3 - [SE1L1_HUMAN] |
| Q5TEA6 | Protein sel-1 homolog 2 OS=Homo sapiens GN=SEL1L2 PE=1 SV=2 - [SE1L2_HUMAN] |
| Q96JX3 | Protein SERAC1 OS=Homo sapiens GN=SERAC1 PE=1 SV=1 - [SRAC1_HUMAN] |
| Q8TF72 | Protein Shroom3 OS=Homo sapiens GN=SHROOM3 PE=1 SV=2 - [SHRM3_HUMAN] |
| Q7Z5N4 | Protein sidekick-1 OS=Homo sapiens GN=SDK1 PE=2 SV=3 - [SDK1_HUMAN] |
| O94964 | Protein SOGA1 OS=Homo sapiens GN=SOGA1 PE=1 SV=2 - [SOGA1_HUMAN] |
| Q5TF21 | Protein SOGA3 OS=Homo sapiens GN=SOGA3 PE=3 SV=1 - [SOGA3_HUMAN] |
| O43597 | Protein sprouty homolog 2 OS=Homo sapiens GN=SPRY2 PE=1 SV=1 - [SPY2_HUMAN] |
| A3KN83 | Protein strawberry notch homolog 1 OS=Homo sapiens GN=SBNO1 PE=1 SV=1 - [SBNO1_HUMAN] |
| Q9HCD6 | Protein TANC2 OS=Homo sapiens GN=TANC2 PE=1 SV=3 - [TANC2_HUMAN] |
| Q86X45 | Protein tilB homolog OS=Homo sapiens GN=LRRC6 PE=1 SV=3 - [TILB_HUMAN] |
| Q9UNS1 | Protein timeless homolog OS=Homo sapiens GN=TIMELESS PE=1 SV=2 - [TIM_HUMAN] |
| Q969W9 | Protein TMEPAI OS=Homo sapiens GN=PMEPA1 PE=1 SV=1 - [PMEPA_HUMAN] |
| O95487 | Protein transport protein Sec24B OS=Homo sapiens GN=SEC24B PE=1 SV=2 - [SC24B_HUMAN] |
| Q9UPX0 | Protein turtle homolog B OS=Homo sapiens GN=IGSF9B PE=2 SV=2 - [TUTLB_HUMAN] |
| O14795 | Protein unc-13 homolog B OS=Homo sapiens GN=UNC13B PE=1 SV=2 - [UN13B_HUMAN] |
| Q8NB66 | Protein unc-13 homolog C OS=Homo sapiens GN=UNC13C PE=2 SV=3 - [UN13C_HUMAN] |
| Q9H3U1 | Protein unc-45 homolog A OS=Homo sapiens GN=UNC45A PE=1 SV=1 - [UN45A_HUMAN] |
| Q8IWX7 | Protein unc-45 homolog B OS=Homo sapiens GN=UNC45B PE=1 SV=1 - [UN45B_HUMAN] |
| Q9P2D8 | Protein unc-79 homolog OS=Homo sapiens GN=UNC79 PE=2 SV=4 - [UNC79_HUMAN] |
| Q8N2C7 | Protein unc-80 homolog OS=Homo sapiens GN=UNC80 PE=2 SV=2 - [UNC80_HUMAN] |
| Q8NEX6 | Protein WFDC11 OS=Homo sapiens GN=WFDC11 PE=3 SV=1 - [WFD11_HUMAN] |
| Q9ULE0 | Protein WWC3 OS=Homo sapiens GN=WWC3 PE=1 SV=3 - [WWC3_HUMAN] |
| Q9GZM5 | Protein YIPF3 OS=Homo sapiens GN=YIPF3 PE=1 SV=1 - [YIPF3_HUMAN] |
| P62699 | Protein yippee-like 5 OS=Homo sapiens GN=YPEL5 PE=1 SV=1 - [YPEL5_HUMAN] |
| Q70YC5 | Protein ZNF365 OS=Homo sapiens GN=ZNF365 PE=1 SV=3 - [ZN365_HUMAN] |
| Q8NE65 | Protein ZNF738 OS=Homo sapiens GN=ZNF738 PE=2 SV=1 - [ZN738_HUMAN] |
| Q9C0D3 | Protein zyg-11 homolog B OS=Homo sapiens GN=ZYG11B PE=1 SV=2 - [ZY11B_HUMAN] |
| Q96RI0 | Proteinase-activated receptor 4 OS=Homo sapiens GN=F2RL3 PE=1 SV=3 - [PAR4_HUMAN] |
| Q9H867 | Protein-lysine methyltransferase METTL21D OS=Homo sapiens GN=VCPKMT PE=1 SV=2 - [MT21D_HUMAN] |
| O60507 | Protein-tyrosine sulfotransferase 1 OS=Homo sapiens GN=TPST1 PE=2 SV=1 - [TPST1_HUMAN] |
| Q92954 | Proteoglycan 4 OS=Homo sapiens GN=PRG4 PE=1 SV=2 - [PRG4_HUMAN] |
| P06454 | Prothymosin alpha OS=Homo sapiens GN=PTMA PE=1 SV=2 - [PTMA_HUMAN] |
| Q9Y5H7 | Protocadherin alpha-5 OS=Homo sapiens GN=PCDHA5 PE=2 SV=1 - [PCDA5_HUMAN] |
| Q9NRJ7 | Protocadherin beta-16 OS=Homo sapiens GN=PCDHB16 PE=1 SV=3 - [PCDBG_HUMAN] |
| Q6V0I7 | Protocadherin Fat 4 OS=Homo sapiens GN=FAT4 PE=1 SV=2 - [FAT4_HUMAN] |
| Q9Y5H1 | Protocadherin gamma-A2 OS=Homo sapiens GN=PCDHGA2 PE=2 SV=1 - [PCDG2_HUMAN] |
| Q9Y5F9 | Protocadherin gamma-B6 OS=Homo sapiens GN=PCDHGB6 PE=2 SV=1 - [PCDGI_HUMAN] |
| Q96JQ0 | Protocadherin-16 OS=Homo sapiens GN=DCHS1 PE=1 SV=1 - [PCD16_HUMAN] |
| Q8TAB3 | Protocadherin-19 OS=Homo sapiens GN=PCDH19 PE=1 SV=3 - [PCD19_HUMAN] |
| Q8N6Y1 | Protocadherin-20 OS=Homo sapiens GN=PCDH20 PE=2 SV=2 - [PCD20_HUMAN] |
| Q96QE2 | Proton myo-inositol cotransporter OS=Homo sapiens GN=SLC2A13 PE=1 SV=3 - [MYCT_HUMAN] |
| P04201 | Proto-oncogene Mas OS=Homo sapiens GN=MAS1 PE=1 SV=1 - [MAS_HUMAN] |
| P15498 | Proto-oncogene vav OS=Homo sapiens GN=VAV1 PE=1 SV=4 - [VAV_HUMAN] |
| P04628 | Proto-oncogene Wnt-1 OS=Homo sapiens GN=WNT1 PE=1 SV=1 - [WNT1_HUMAN] |
| Q9H792 | Pseudopodium-enriched atypical kinase 1 OS=Homo sapiens GN=PEAK1 PE=1 SV=4 - [PEAK1_HUMAN] |
| P55786 | Puromycin-sensitive aminopeptidase OS=Homo sapiens GN=NPEPPS PE=1 SV=2 - [PSA_HUMAN] |
| Q92771 | Putative ATP-dependent RNA helicase DDX12 OS=Homo sapiens GN=DDX12P PE=5 SV=3 - [DDX12_HUMAN] |
| Q9BYX7 | Putative beta-actin-like protein 3 OS=Homo sapiens GN=POTEKP PE=5 SV=1 - [ACTBM_HUMAN] |
| Q9NP73 | Putative bifunctional UDP-N-acetylglucosamine transferase and deubiquitinase ALG13 OS=Homo sapiens GN=ALG13 PE=1 SV=2 - [ALG13_HUMAN] |
| Q6NXN4 | Putative C-mannosyltransferase DPY19L2P1 OS=Homo sapiens GN=DPY19L2P1 PE=2 SV=1 - [D19P1_HUMAN] |
| Q8IX95 | Putative cTAGE family member 3 OS=Homo sapiens GN=CTAGE3P PE=5 SV=1 - [CTGE3_HUMAN] |
| A0A087X1C5 | Putative cytochrome P450 2D7 OS=Homo sapiens GN=CYP2D7 PE=5 SV=1 - [CP2D7_HUMAN] |
| P0CG22 | Putative dehydrogenase/reductase SDR family member 4-like 1 OS=Homo sapiens GN=DHRS4L1 PE=5 SV=1 - [DR4L1_HUMAN] |
| Q8N806 | Putative E3 ubiquitin-protein ligase UBR7 OS=Homo sapiens GN=UBR7 PE=1 SV=2 - [UBR7_HUMAN] |
| Q5T036 | Putative FAM120A opposite strand protein OS=Homo sapiens GN=FAM120AOS PE=5 SV=1 - [F120S_HUMAN] |
| Q9BXT6 | Putative helicase Mov10l1 OS=Homo sapiens GN=MOV10L1 PE=2 SV=1 - [M10L1_HUMAN] |
| Q9NQX0 | Putative histone-lysine N-methyltransferase PRDM6 OS=Homo sapiens GN=PRDM6 PE=2 SV=2 - [PRDM6_HUMAN] |
| Q8N3S3 | Putative homeodomain transcription factor 2 OS=Homo sapiens GN=PHTF2 PE=2 SV=2 - [PHTF2_HUMAN] |
| A6NCN2 | Putative keratin-87 protein OS=Homo sapiens GN=KRT87P PE=5 SV=4 - [KR87P_HUMAN] |
| Q5JSQ8 | Putative KHDC1-like protein OS=Homo sapiens GN=KHDC1L PE=5 SV=1 - [KHDCL_HUMAN] |
| Q8NE18 | Putative methyltransferase NSUN7 OS=Homo sapiens GN=NSUN7 PE=2 SV=4 - [NSUN7_HUMAN] |
| Q49A26 | Putative oxidoreductase GLYR1 OS=Homo sapiens GN=GLYR1 PE=1 SV=3 - [GLYR1_HUMAN] |
| A2A3N6 | Putative PIP5K1A and PSMD4-like protein OS=Homo sapiens GN=PIPSL PE=5 SV=1 - [PIPSL_HUMAN] |
| Q9C0F0 | Putative Polycomb group protein ASXL3 OS=Homo sapiens GN=ASXL3 PE=2 SV=3 - [ASXL3_HUMAN] |
| A6NNC1 | Putative POM121-like protein 1-like OS=Homo sapiens PE=5 SV=3 - [P12LL_HUMAN] |
| A4D2B8 | Putative postmeiotic segregation increased 2-like protein 1 OS=Homo sapiens GN=PMS2P1 PE=5 SV=1 - [PM2P1_HUMAN] |
| P0DMR3 | Putative protein ATXN8OS OS=Homo sapiens GN=ATXN8OS PE=5 SV=1 - [AT8OS_HUMAN] |
| O60756 | Putative protein BCE-1 OS=Homo sapiens GN=BCE1 PE=5 SV=1 - [BCE1_HUMAN] |
| A6NHR8 | Putative protein FAM47D OS=Homo sapiens GN=FAM47DP PE=5 SV=3 - [FA47D_HUMAN] |
| Q8IXW5 | Putative RNA polymerase II subunit B1 CTD phosphatase RPAP2 OS=Homo sapiens GN=RPAP2 PE=1 SV=1 - [RPAP2_HUMAN] |
| Q8NDT2 | Putative RNA-binding protein 15B OS=Homo sapiens GN=RBM15B PE=1 SV=3 - [RB15B_HUMAN] |
| Q9Y383 | Putative RNA-binding protein Luc7-like 2 OS=Homo sapiens GN=LUC7L2 PE=1 SV=2 - [LC7L2_HUMAN] |
| Q9GZW5 | Putative SCAN domain-containing protein SCAND2P OS=Homo sapiens GN=SCAND2P PE=5 SV=2 - [SCND2_HUMAN] |
| Q08AI6 | Putative sodium-coupled neutral amino acid transporter 11 OS=Homo sapiens GN=SLC38A11 PE=2 SV=1 - [S38AB_HUMAN] |
| A6NKX4 | Putative solute carrier family 22 member 31 OS=Homo sapiens GN=SLC22A31 PE=3 SV=3 - [S22AV_HUMAN] |
| P0C874 | Putative spermatogenesis-associated protein 31D3 OS=Homo sapiens GN=SPATA31D3 PE=5 SV=1 - [S31D3_HUMAN] |
| Q9H489 | Putative testis-specific Y-encoded-like protein 3 OS=Homo sapiens GN=TSPY26P PE=5 SV=1 - [TSY26_HUMAN] |
| Q8IY50 | Putative thiamine transporter SLC35F3 OS=Homo sapiens GN=SLC35F3 PE=2 SV=2 - [S35F3_HUMAN] |
| O14753 | Putative transcription factor Ovo-like 1 OS=Homo sapiens GN=OVOL1 PE=2 SV=3 - [OVOL1_HUMAN] |
| P0C7V6 | Putative transcription factor SPT20 homolog-like 2 OS=Homo sapiens GN=SUPT20HL2 PE=5 SV=1 - [SP202_HUMAN] |
| A8K010 | Putative transcriptional regulator encoded by LINC00473 OS=Homo sapiens GN=LINC00473 PE=5 SV=2 - [CF176_HUMAN] |
| A6NLI5 | Putative tripartite motif-containing protein 64C OS=Homo sapiens GN=TRIM64C PE=5 SV=3 - [TR64C_HUMAN] |
| Q495Z4 | Putative uncharacterized protein ASB16-AS1 OS=Homo sapiens GN=ASB16-AS1 PE=5 SV=2 - [ASAS1_HUMAN] |
| Q8TAV5 | Putative uncharacterized protein C11orf45 OS=Homo sapiens GN=C11orf45 PE=2 SV=1 - [CK045_HUMAN] |
| Q6ZS72 | Putative uncharacterized protein C19orf35 OS=Homo sapiens GN=C19orf35 PE=2 SV=1 - [CS035_HUMAN] |
| Q9NVV2 | Putative uncharacterized protein C19orf73 OS=Homo sapiens GN=C19orf73 PE=1 SV=2 - [CS073_HUMAN] |
| B1AJZ1 | Putative uncharacterized protein C1orf196 OS=Homo sapiens GN=C1orf196 PE=5 SV=2 - [CA196_HUMAN] |
| Q6ZS94 | Putative uncharacterized protein C1orf229 OS=Homo sapiens GN=C1orf229 PE=2 SV=1 - [CA229_HUMAN] |
| A6NFR6 | Putative uncharacterized protein C5orf60 OS=Homo sapiens GN=C5orf60 PE=1 SV=2 - [CE060_HUMAN] |
| A4D174 | Putative uncharacterized protein C7orf71 OS=Homo sapiens GN=C7orf71 PE=2 SV=1 - [CG071_HUMAN] |
| Q96LI9 | Putative uncharacterized protein CXorf58 OS=Homo sapiens GN=CXorf58 PE=2 SV=2 - [CX058_HUMAN] |
| A6NH13 | Putative uncharacterized protein DNAJC9-AS1 OS=Homo sapiens GN=DNAJC9-AS1 PE=2 SV=2 - [DAS1_HUMAN] |
| Q6ZNR8 | Putative uncharacterized protein encoded by LINC00176 OS=Homo sapiens GN=LINC00176 PE=5 SV=1 - [PRR17_HUMAN] |
| Q5T6M2 | Putative uncharacterized protein encoded by LINC00242 OS=Homo sapiens GN=LINC00242 PE=5 SV=1 - [CF122_HUMAN] |
| Q6ZUF6 | Putative uncharacterized protein encoded by LINC00336 OS=Homo sapiens GN=LINC00336 PE=5 SV=1 - [NC336_HUMAN] |
| Q8NI28 | Putative uncharacterized protein encoded by LINC01006 OS=Homo sapiens GN=LINC01006 PE=5 SV=1 - [CG013_HUMAN] |
| Q9H7H1 | Putative uncharacterized protein encoded by RUNX1-IT1 OS=Homo sapiens GN=RUNX1-IT1 PE=2 SV=1 - [RUIT1_HUMAN] |
| A8MVM7 | Putative uncharacterized protein ENSP00000382790 OS=Homo sapiens PE=5 SV=3 - [YD021_HUMAN] |
| Q8NA97 | Putative uncharacterized protein FER1L6-AS1 OS=Homo sapiens GN=FER1L6-AS1 PE=2 SV=1 - [FEAS1_HUMAN] |
| Q8N1Y9 | Putative uncharacterized protein FLJ37218 OS=Homo sapiens PE=5 SV=2 - [YI025_HUMAN] |
| Q6ZSR6 | Putative uncharacterized protein FLJ45256 OS=Homo sapiens PE=2 SV=3 - [YP007_HUMAN] |
| Q8IYB0 | Putative uncharacterized protein MGC39545 OS=Homo sapiens PE=5 SV=2 - [YK038_HUMAN] |
| Q0IIN9 | Putative uncharacterized protein ZNF252P-AS1 OS=Homo sapiens GN=ZNF252P-AS1 PE=5 SV=1 - [ZNFS1_HUMAN] |
| A6NL46 | Putative UPF0607 protein ENSP00000332738 OS=Homo sapiens PE=3 SV=3 - [YF016_HUMAN] |
| A8MU76 | Putative UPF0607 protein ENSP00000381418 OS=Homo sapiens PE=3 SV=2 - [YP034_HUMAN] |
| Q8N9G6 | Putative UPF0607 protein FLJ37424 OS=Homo sapiens PE=2 SV=1 - [YJ012_HUMAN] |
| Q92670 | Putative zinc finger protein 75C OS=Homo sapiens GN=ZNF75CP PE=5 SV=2 - [ZN75C_HUMAN] |
| Q6ZT77 | Putative zinc finger protein 826 OS=Homo sapiens GN=ZNF826P PE=5 SV=2 - [ZN826_HUMAN] |
| Q8WU10 | Pyridine nucleotide-disulfide oxidoreductase domain-containing protein 1 OS=Homo sapiens GN=PYROXD1 PE=1 SV=1 - [PYRD1_HUMAN] |
| Q8TCD6 | Pyridoxal phosphate phosphatase PHOSPHO2 OS=Homo sapiens GN=PHOSPHO2 PE=1 SV=1 - [PHOP2_HUMAN] |
| A6NFU8 | Pyroglutamyl-peptidase 1-like protein OS=Homo sapiens GN=PGPEP1L PE=2 SV=4 - [PGPIL_HUMAN] |
| P30613 | Pyruvate kinase PKLR OS=Homo sapiens GN=PKLR PE=1 SV=2 - [KPYR_HUMAN] |
| Q9H974 | Queuine tRNA-ribosyltransferase subunit QTRTD1 OS=Homo sapiens GN=QTRTD1 PE=1 SV=1 - [QTRD1_HUMAN] |
| Q9Y2K5 | R3H domain-containing protein 2 OS=Homo sapiens GN=R3HDM2 PE=1 SV=3 - [R3HD2_HUMAN] |
| Q8NFW9 | Rab effector MyRIP OS=Homo sapiens GN=MYRIP PE=1 SV=2 - [MYRIP_HUMAN] |
| Q5HYI8 | Rab-like protein 3 OS=Homo sapiens GN=RABL3 PE=1 SV=1 - [RABL3_HUMAN] |
| Q86UC2 | Radial spoke head protein 3 homolog OS=Homo sapiens GN=RSPH3 PE=1 SV=1 - [RSPH3_HUMAN] |
| P46695 | Radiation-inducible immediate-early gene IEX-1 OS=Homo sapiens GN=IER3 PE=1 SV=4 - [IEX1_HUMAN] |
| Q14699 | Raftlin OS=Homo sapiens GN=RFTN1 PE=1 SV=4 - [RFTN1_HUMAN] |
| Q86X10 | Ral GTPase-activating protein subunit beta OS=Homo sapiens GN=RALGAPB PE=1 SV=1 - [RLGPB_HUMAN] |
| Q9NZL6 | Ral guanine nucleotide dissociation stimulator-like 1 OS=Homo sapiens GN=RGL1 PE=1 SV=1 - [RGL1_HUMAN] |
| Q96D71 | RalBP1-associated Eps domain-containing protein 1 OS=Homo sapiens GN=REPS1 PE=1 SV=3 - [REPS1_HUMAN] |
| Q8NFH8 | RalBP1-associated Eps domain-containing protein 2 OS=Homo sapiens GN=REPS2 PE=1 SV=2 - [REPS2_HUMAN] |
| P46060 | Ran GTPase-activating protein 1 OS=Homo sapiens GN=RANGAP1 PE=1 SV=1 - [RAGP1_HUMAN] |
| Q6VN20 | Ran-binding protein 10 OS=Homo sapiens GN=RANBP10 PE=1 SV=1 - [RBP10_HUMAN] |
| Q9H2T7 | Ran-binding protein 17 OS=Homo sapiens GN=RANBP17 PE=2 SV=1 - [RBP17_HUMAN] |
| O60518 | Ran-binding protein 6 OS=Homo sapiens GN=RANBP6 PE=1 SV=2 - [RNBP6_HUMAN] |
| P0DJD0 | RANBP2-like and GRIP domain-containing protein 1 OS=Homo sapiens GN=RGPD1 PE=2 SV=1 - [RGPD1_HUMAN] |
| Q7Z3J3 | RanBP2-like and GRIP domain-containing protein 4 OS=Homo sapiens GN=RGPD4 PE=2 SV=3 - [RGPD4_HUMAN] |
| Q9UHV5 | Rap guanine nucleotide exchange factor-like 1 OS=Homo sapiens GN=RAPGEFL1 PE=1 SV=2 - [RPGFL_HUMAN] |
| Q684P5 | Rap1 GTPase-activating protein 2 OS=Homo sapiens GN=RAP1GAP2 PE=1 SV=2 - [RPGP2_HUMAN] |
| Q8WWW0 | Ras association domain-containing protein 5 OS=Homo sapiens GN=RASSF5 PE=1 SV=1 - [RASF5_HUMAN] |
| Q13283 | Ras GTPase-activating protein-binding protein 1 OS=Homo sapiens GN=G3BP1 PE=1 SV=1 - [G3BP1_HUMAN] |
| Q86VI3 | Ras GTPase-activating-like protein IQGAP3 OS=Homo sapiens GN=IQGAP3 PE=1 SV=2 - [IQGA3_HUMAN] |
| O95267 | RAS guanyl-releasing protein 1 OS=Homo sapiens GN=RASGRP1 PE=1 SV=2 - [GRP1_HUMAN] |
| P60763 | Ras-related C3 botulinum toxin substrate 3 OS=Homo sapiens GN=RAC3 PE=1 SV=1 - [RAC3_HUMAN] |
| Q9NX57 | Ras-related protein Rab-20 OS=Homo sapiens GN=RAB20 PE=1 SV=1 - [RAB20_HUMAN] |
| Q9ULW5 | Ras-related protein Rab-26 OS=Homo sapiens GN=RAB26 PE=1 SV=3 - [RAB26_HUMAN] |
| Q14088 | Ras-related protein Rab-33A OS=Homo sapiens GN=RAB33A PE=1 SV=2 - [RB33A_HUMAN] |
| P20337 | Ras-related protein Rab-3B OS=Homo sapiens GN=RAB3B PE=1 SV=2 - [RAB3B_HUMAN] |
| Q8TDY2 | RB1-inducible coiled-coil protein 1 OS=Homo sapiens GN=RB1CC1 PE=1 SV=3 - [RBCC1_HUMAN] |
| Q13127 | RE1-silencing transcription factor OS=Homo sapiens GN=REST PE=1 SV=3 - [REST_HUMAN] |
| P04626 | Receptor tyrosine-protein kinase erbB-2 OS=Homo sapiens GN=ERBB2 PE=1 SV=1 - [ERBB2_HUMAN] |
| P57078 | Receptor-interacting serine/threonine-protein kinase 4 OS=Homo sapiens GN=RIPK4 PE=1 SV=1 - [RIPK4_HUMAN] |
| P36888 | Receptor-type tyrosine-protein kinase FLT3 OS=Homo sapiens GN=FLT3 PE=1 SV=2 - [FLT3_HUMAN] |
| P18433 | Receptor-type tyrosine-protein phosphatase alpha OS=Homo sapiens GN=PTPRA PE=1 SV=2 - [PTPRA_HUMAN] |
| P23467 | Receptor-type tyrosine-protein phosphatase beta OS=Homo sapiens GN=PTPRB PE=1 SV=3 - [PTPRB_HUMAN] |
| P23468 | Receptor-type tyrosine-protein phosphatase delta OS=Homo sapiens GN=PTPRD PE=1 SV=2 - [PTPRD_HUMAN] |
| P28827 | Receptor-type tyrosine-protein phosphatase mu OS=Homo sapiens GN=PTPRM PE=1 SV=2 - [PTPRM_HUMAN] |
| Q92932 | Receptor-type tyrosine-protein phosphatase N2 OS=Homo sapiens GN=PTPRN2 PE=1 SV=2 - [PTPR2_HUMAN] |
| P23471 | Receptor-type tyrosine-protein phosphatase zeta OS=Homo sapiens GN=PTPRZ1 PE=1 SV=4 - [PTPRZ_HUMAN] |
| P35243 | Recoverin OS=Homo sapiens GN=RCVRN PE=1 SV=2 - [RECO_HUMAN] |
| Q96E14 | RecQ-mediated genome instability protein 2 OS=Homo sapiens GN=RMI2 PE=1 SV=2 - [RMI2_HUMAN] |
| Q86UR5 | Regulating synaptic membrane exocytosis protein 1 OS=Homo sapiens GN=RIMS1 PE=1 SV=1 - [RIMS1_HUMAN] |
| Q96P16 | Regulation of nuclear pre-mRNA domain-containing protein 1A OS=Homo sapiens GN=RPRD1A PE=1 SV=1 - [RPR1A_HUMAN] |
| Q5VT52 | Regulation of nuclear pre-mRNA domain-containing protein 2 OS=Homo sapiens GN=RPRD2 PE=1 SV=1 - [RPRD2_HUMAN] |
| O94810 | Regulator of G-protein signaling 11 OS=Homo sapiens GN=RGS11 PE=1 SV=2 - [RGS11_HUMAN] |
| O14924 | Regulator of G-protein signaling 12 OS=Homo sapiens GN=RGS12 PE=1 SV=1 - [RGS12_HUMAN] |
| Q8NE09 | Regulator of G-protein signaling 22 OS=Homo sapiens GN=RGS22 PE=1 SV=3 - [RGS22_HUMAN] |
| P49758 | Regulator of G-protein signaling 6 OS=Homo sapiens GN=RGS6 PE=1 SV=5 - [RGS6_HUMAN] |
| Q92900 | Regulator of nonsense transcripts 1 OS=Homo sapiens GN=UPF1 PE=1 SV=2 - [RENT1_HUMAN] |
| P00797 | Renin OS=Homo sapiens GN=REN PE=1 SV=1 - [RENI_HUMAN] |
| O75787 | Renin receptor OS=Homo sapiens GN=ATP6AP2 PE=1 SV=2 - [RENR_HUMAN] |
| P40938 | Replication factor C subunit 3 OS=Homo sapiens GN=RFC3 PE=1 SV=2 - [RFC3_HUMAN] |
| P40937 | Replication factor C subunit 5 OS=Homo sapiens GN=RFC5 PE=1 SV=1 - [RFC5_HUMAN] |
| Q9NWS8 | Required for meiotic nuclear division protein 1 homolog OS=Homo sapiens GN=RMND1 PE=1 SV=2 - [RMND1_HUMAN] |
| Q16799 | Reticulon-1 OS=Homo sapiens GN=RTN1 PE=1 SV=1 - [RTN1_HUMAN] |
| P78363 | Retinal-specific ATP-binding cassette transporter OS=Homo sapiens GN=ABCA4 PE=1 SV=3 - [ABCA4_HUMAN] |
| P06400 | Retinoblastoma-associated protein OS=Homo sapiens GN=RB1 PE=1 SV=2 - [RB_HUMAN] |
| P28749 | Retinoblastoma-like protein 1 OS=Homo sapiens GN=RBL1 PE=1 SV=3 - [RBL1_HUMAN] |
| P10276 | Retinoic acid receptor alpha OS=Homo sapiens GN=RARA PE=1 SV=2 - [RARA_HUMAN] |
| P10826 | Retinoic acid receptor beta OS=Homo sapiens GN=RARB PE=1 SV=2 - [RARB_HUMAN] |
| Q9UL19 | Retinoic acid receptor responder protein 3 OS=Homo sapiens GN=RARRES3 PE=1 SV=1 - [HRSL4_HUMAN] |
| Q7Z5J4 | Retinoic acid-induced protein 1 OS=Homo sapiens GN=RAI1 PE=1 SV=2 - [RAI1_HUMAN] |
| P10745 | Retinol-binding protein 3 OS=Homo sapiens GN=RBP3 PE=1 SV=2 - [RET3_HUMAN] |
| Q5T5U3 | Rho GTPase-activating protein 21 OS=Homo sapiens GN=ARHGAP21 PE=1 SV=1 - [RHG21_HUMAN] |
| Q9P227 | Rho GTPase-activating protein 23 OS=Homo sapiens GN=ARHGAP23 PE=1 SV=2 - [RHG23_HUMAN] |
| Q52LW3 | Rho GTPase-activating protein 29 OS=Homo sapiens GN=ARHGAP29 PE=1 SV=2 - [RHG29_HUMAN] |
| Q9NRY4 | Rho GTPase-activating protein 35 OS=Homo sapiens GN=ARHGAP35 PE=1 SV=3 - [RHG35_HUMAN] |
| Q6ZRI8 | Rho GTPase-activating protein 36 OS=Homo sapiens GN=ARHGAP36 PE=2 SV=1 - [RHG36_HUMAN] |
| Q9C0H5 | Rho GTPase-activating protein 39 OS=Homo sapiens GN=ARHGAP39 PE=1 SV=2 - [RHG39_HUMAN] |
| Q9BRR9 | Rho GTPase-activating protein 9 OS=Homo sapiens GN=ARHGAP9 PE=1 SV=2 - [RHG09_HUMAN] |
| Q92888 | Rho guanine nucleotide exchange factor 1 OS=Homo sapiens GN=ARHGEF1 PE=1 SV=2 - [ARHG1_HUMAN] |
| Q96PE2 | Rho guanine nucleotide exchange factor 17 OS=Homo sapiens GN=ARHGEF17 PE=1 SV=1 - [ARHGH_HUMAN] |
| Q8IW93 | Rho guanine nucleotide exchange factor 19 OS=Homo sapiens GN=ARHGEF19 PE=1 SV=1 - [ARHGJ_HUMAN] |
| A5YM69 | Rho guanine nucleotide exchange factor 35 OS=Homo sapiens GN=ARHGEF35 PE=1 SV=1 - [ARG35_HUMAN] |
| Q12774 | Rho guanine nucleotide exchange factor 5 OS=Homo sapiens GN=ARHGEF5 PE=1 SV=3 - [ARHG5_HUMAN] |
| P61587 | Rho-related GTP-binding protein RhoE OS=Homo sapiens GN=RND3 PE=1 SV=1 - [RND3_HUMAN] |
| Q8IY67 | Ribonucleoprotein PTB-binding 1 OS=Homo sapiens GN=RAVER1 PE=1 SV=1 - [RAVR1_HUMAN] |
| O75582 | Ribosomal protein S6 kinase alpha-5 OS=Homo sapiens GN=RPS6KA5 PE=1 SV=1 - [KS6A5_HUMAN] |
| Q9UK32 | Ribosomal protein S6 kinase alpha-6 OS=Homo sapiens GN=RPS6KA6 PE=1 SV=1 - [KS6A6_HUMAN] |
| Q9H7B2 | Ribosome production factor 2 homolog OS=Homo sapiens GN=RPF2 PE=1 SV=2 - [RPF2_HUMAN] |
| O94941 | RING finger protein 37 OS=Homo sapiens GN=UBOX5 PE=1 SV=1 - [RNF37_HUMAN] |
| Q96E39 | RNA binding motif protein, X-linked-like-1 OS=Homo sapiens GN=RBMXL1 PE=1 SV=1 - [RMXL1_HUMAN] |
| Q9GZR2 | RNA exonuclease 4 OS=Homo sapiens GN=REXO4 PE=1 SV=2 - [REXO4_HUMAN] |
| P55199 | RNA polymerase II elongation factor ELL OS=Homo sapiens GN=ELL PE=1 SV=1 - [ELL_HUMAN] |
| Q92541 | RNA polymerase-associated protein RTF1 homolog OS=Homo sapiens GN=RTF1 PE=1 SV=4 - [RTF1_HUMAN] |
| Q5U5Q3 | RNA-binding E3 ubiquitin-protein ligase MEX3C OS=Homo sapiens GN=MEX3C PE=1 SV=3 - [MEX3C_HUMAN] |
| O75526 | RNA-binding motif protein, X-linked-like-2 OS=Homo sapiens GN=RBMXL2 PE=1 SV=3 - [RMXL2_HUMAN] |
| Q8N7X1 | RNA-binding motif protein, X-linked-like-3 OS=Homo sapiens GN=RBMXL3 PE=2 SV=2 - [RMXL3_HUMAN] |
| A6NDE4 | RNA-binding motif protein, Y chromosome, family 1 member B OS=Homo sapiens GN=RBMY1B PE=2 SV=2 - [RBY1B_HUMAN] |
| P29558 | RNA-binding motif, single-stranded-interacting protein 1 OS=Homo sapiens GN=RBMS1 PE=1 SV=3 - [RBMS1_HUMAN] |
| Q15434 | RNA-binding motif, single-stranded-interacting protein 2 OS=Homo sapiens GN=RBMS2 PE=1 SV=1 - [RBMS2_HUMAN] |
| P98175 | RNA-binding protein 10 OS=Homo sapiens GN=RBM10 PE=1 SV=3 - [RBM10_HUMAN] |
| Q5T481 | RNA-binding protein 20 OS=Homo sapiens GN=RBM20 PE=1 SV=3 - [RBM20_HUMAN] |
| P49756 | RNA-binding protein 25 OS=Homo sapiens GN=RBM25 PE=1 SV=3 - [RBM25_HUMAN] |
| Q5T8P6 | RNA-binding protein 26 OS=Homo sapiens GN=RBM26 PE=1 SV=3 - [RBM26_HUMAN] |
| Q96EV2 | RNA-binding protein 33 OS=Homo sapiens GN=RBM33 PE=1 SV=3 - [RBM33_HUMAN] |
| Q14498 | RNA-binding protein 39 OS=Homo sapiens GN=RBM39 PE=1 SV=2 - [RBM39_HUMAN] |
| Q01844 | RNA-binding protein EWS OS=Homo sapiens GN=EWSR1 PE=1 SV=1 - [EWS_HUMAN] |
| Q15287 | RNA-binding protein with serine-rich domain 1 OS=Homo sapiens GN=RNPS1 PE=1 SV=1 - [RNPS1_HUMAN] |
| Q5TZA2 | Rootletin OS=Homo sapiens GN=CROCC PE=1 SV=1 - [CROCC_HUMAN] |
| Q5TC82 | Roquin-1 OS=Homo sapiens GN=RC3H1 PE=1 SV=1 - [RC3H1_HUMAN] |
| Q96MS0 | Roundabout homolog 3 OS=Homo sapiens GN=ROBO3 PE=1 SV=2 - [ROBO3_HUMAN] |
| Q6IN84 | rRNA methyltransferase 1, mitochondrial OS=Homo sapiens GN=MRM1 PE=1 SV=1 - [MRM1_HUMAN] |
| Q9BVN2 | RUN and SH3 domain-containing protein 1 OS=Homo sapiens GN=RUSC1 PE=1 SV=3 - [RUSC1_HUMAN] |
| Q96NL0 | RUN domain-containing protein 3B OS=Homo sapiens GN=RUNDC3B PE=2 SV=1 - [RUN3B_HUMAN] |
| Q9Y230 | RuvB-like 2 OS=Homo sapiens GN=RUVBL2 PE=1 SV=3 - [RUVB2_HUMAN] |
| P21817 | Ryanodine receptor 1 OS=Homo sapiens GN=RYR1 PE=1 SV=3 - [RYR1_HUMAN] |
| Q92736 | Ryanodine receptor 2 OS=Homo sapiens GN=RYR2 PE=1 SV=3 - [RYR2_HUMAN] |
| Q15413 | Ryanodine receptor 3 OS=Homo sapiens GN=RYR3 PE=1 SV=3 - [RYR3_HUMAN] |
| Q8N5C6 | S1 RNA-binding domain-containing protein 1 OS=Homo sapiens GN=SRBD1 PE=1 SV=2 - [SRBD1_HUMAN] |
| A6NKF1 | SAC3 domain-containing protein 1 OS=Homo sapiens GN=SAC3D1 PE=1 SV=2 - [SAC31_HUMAN] |
| Q9NZJ4 | Sacsin OS=Homo sapiens GN=SACS PE=1 SV=2 - [SACS_HUMAN] |
| P31153 | S-adenosylmethionine synthase isoform type-2 OS=Homo sapiens GN=MAT2A PE=1 SV=1 - [METK2_HUMAN] |
| Q9NWH9 | SAFB-like transcription modulator OS=Homo sapiens GN=SLTM PE=1 SV=2 - [SLTM_HUMAN] |
| O94885 | SAM and SH3 domain-containing protein 1 OS=Homo sapiens GN=SASH1 PE=1 SV=3 - [SASH1_HUMAN] |
| Q14BN4 | Sarcolemmal membrane-associated protein OS=Homo sapiens GN=SLMAP PE=1 SV=1 - [SLMAP_HUMAN] |
| Q9NXZ1 | Sarcoma antigen 1 OS=Homo sapiens GN=SAGE1 PE=1 SV=2 - [SAGE1_HUMAN] |
| P16615 | Sarcoplasmic/endoplasmic reticulum calcium ATPase 2 OS=Homo sapiens GN=ATP2A2 PE=1 SV=1 - [AT2A2_HUMAN] |
| Q9UL12 | Sarcosine dehydrogenase, mitochondrial OS=Homo sapiens GN=SARDH PE=1 SV=1 - [SARDH_HUMAN] |
| Q6R2W3 | SCAN domain-containing protein 3 OS=Homo sapiens GN=ZBED9 PE=2 SV=1 - [SCND3_HUMAN] |
| Q6AZY7 | Scavenger receptor class A member 3 OS=Homo sapiens GN=SCARA3 PE=1 SV=1 - [SCAR3_HUMAN] |
| Q7Z7L1 | Schlafen family member 11 OS=Homo sapiens GN=SLFN11 PE=1 SV=2 - [SLN11_HUMAN] |
| P0C7P3 | Schlafen family member 14 OS=Homo sapiens GN=SLFN14 PE=2 SV=2 - [SLN14_HUMAN] |
| Q08AF3 | Schlafen family member 5 OS=Homo sapiens GN=SLFN5 PE=1 SV=1 - [SLFN5_HUMAN] |
| A2VEC9 | SCO-spondin OS=Homo sapiens GN=SSPO PE=2 SV=1 - [SSPO_HUMAN] |
| O75711 | Scrapie-responsive protein 1 OS=Homo sapiens GN=SCRG1 PE=1 SV=1 - [SCRG1_HUMAN] |
| Q6P3W7 | SCY1-like protein 2 OS=Homo sapiens GN=SCYL2 PE=1 SV=1 - [SCYL2_HUMAN] |
| Q8WVM8 | Sec1 family domain-containing protein 1 OS=Homo sapiens GN=SCFD1 PE=1 SV=4 - [SCFD1_HUMAN] |
| Q8WU76 | Sec1 family domain-containing protein 2 OS=Homo sapiens GN=SCFD2 PE=1 SV=2 - [SCFD2_HUMAN] |
| O76054 | SEC14-like protein 2 OS=Homo sapiens GN=SEC14L2 PE=1 SV=1 - [S14L2_HUMAN] |
| Q9UDX4 | SEC14-like protein 3 OS=Homo sapiens GN=SEC14L3 PE=1 SV=1 - [S14L3_HUMAN] |
| O43304 | SEC14-like protein 5 OS=Homo sapiens GN=SEC14L5 PE=2 SV=3 - [S14L5_HUMAN] |
| Q9Y6Y8 | SEC23-interacting protein OS=Homo sapiens GN=SEC23IP PE=1 SV=1 - [S23IP_HUMAN] |
| Q8N474 | Secreted frizzled-related protein 1 OS=Homo sapiens GN=SFRP1 PE=1 SV=1 - [SFRP1_HUMAN] |
| Q9BYH1 | Seizure 6-like protein OS=Homo sapiens GN=SEZ6L PE=1 SV=1 - [SE6L1_HUMAN] |
| Q99985 | Semaphorin-3C OS=Homo sapiens GN=SEMA3C PE=2 SV=2 - [SEM3C_HUMAN] |
| O95025 | Semaphorin-3D OS=Homo sapiens GN=SEMA3D PE=2 SV=2 - [SEM3D_HUMAN] |
| P04279 | Semenogelin-1 OS=Homo sapiens GN=SEMG1 PE=1 SV=2 - [SEMG1_HUMAN] |
| Q02383 | Semenogelin-2 OS=Homo sapiens GN=SEMG2 PE=1 SV=1 - [SEMG2_HUMAN] |
| Q9P0U3 | Sentrin-specific protease 1 OS=Homo sapiens GN=SENP1 PE=1 SV=2 - [SENP1_HUMAN] |
| Q96HI0 | Sentrin-specific protease 5 OS=Homo sapiens GN=SENP5 PE=1 SV=3 - [SENP5_HUMAN] |
| Q8WYJ6 | Septin-1 OS=Homo sapiens GN=SEPT1 PE=1 SV=2 - [SEPT1_HUMAN] |
| Q9P0V9 | Septin-10 OS=Homo sapiens GN=SEPT10 PE=1 SV=2 - [SEP10_HUMAN] |
| Q16181 | Septin-7 OS=Homo sapiens GN=SEPT7 PE=1 SV=2 - [SEPT7_HUMAN] |
| Q9UHD8 | Septin-9 OS=Homo sapiens GN=SEPT9 PE=1 SV=2 - [SEPT9_HUMAN] |
| O15270 | Serine palmitoyltransferase 2 OS=Homo sapiens GN=SPTLC2 PE=1 SV=1 - [SPTC2_HUMAN] |
| Q7RTY3 | Serine protease 45 OS=Homo sapiens GN=PRSS45 PE=2 SV=1 - [PRS45_HUMAN] |
| Q8IYB3 | Serine/arginine repetitive matrix protein 1 OS=Homo sapiens GN=SRRM1 PE=1 SV=2 - [SRRM1_HUMAN] |
| Q9UQ35 | Serine/arginine repetitive matrix protein 2 OS=Homo sapiens GN=SRRM2 PE=1 SV=2 - [SRRM2_HUMAN] |
| B3KS81 | Serine/arginine repetitive matrix protein 5 OS=Homo sapiens GN=SRRM5 PE=1 SV=3 - [SRRM5_HUMAN] |
| P84103 | Serine/arginine-rich splicing factor 3 OS=Homo sapiens GN=SRSF3 PE=1 SV=1 - [SRSF3_HUMAN] |
| Q8WUJ0 | Serine/threonine/tyrosine-interacting protein OS=Homo sapiens GN=STYX PE=1 SV=1 - [STYX_HUMAN] |
| P49842 | Serine/threonine-protein kinase 19 OS=Homo sapiens GN=STK19 PE=1 SV=2 - [STK19_HUMAN] |
| Q9P289 | Serine/threonine-protein kinase 26 OS=Homo sapiens GN=STK26 PE=1 SV=2 - [STK26_HUMAN] |
| Q9BXU1 | Serine/threonine-protein kinase 31 OS=Homo sapiens GN=STK31 PE=2 SV=2 - [STK31_HUMAN] |
| Q8WU08 | Serine/threonine-protein kinase 32A OS=Homo sapiens GN=STK32A PE=1 SV=2 - [ST32A_HUMAN] |
| Q9BYT3 | Serine/threonine-protein kinase 33 OS=Homo sapiens GN=STK33 PE=1 SV=1 - [STK33_HUMAN] |
| Q9NRP7 | Serine/threonine-protein kinase 36 OS=Homo sapiens GN=STK36 PE=1 SV=2 - [STK36_HUMAN] |
| Q9BZL6 | Serine/threonine-protein kinase D2 OS=Homo sapiens GN=PRKD2 PE=1 SV=2 - [KPCD2_HUMAN] |
| O94806 | Serine/threonine-protein kinase D3 OS=Homo sapiens GN=PRKD3 PE=1 SV=1 - [KPCD3_HUMAN] |
| Q96Q04 | Serine/threonine-protein kinase LMTK3 OS=Homo sapiens GN=LMTK3 PE=1 SV=2 - [LMTK3_HUMAN] |
| P20794 | Serine/threonine-protein kinase MAK OS=Homo sapiens GN=MAK PE=1 SV=2 - [MAK_HUMAN] |
| Q6DT37 | Serine/threonine-protein kinase MRCK gamma OS=Homo sapiens GN=CDC42BPG PE=1 SV=2 - [MRCKG_HUMAN] |
| Q16513 | Serine/threonine-protein kinase N2 OS=Homo sapiens GN=PKN2 PE=1 SV=1 - [PKN2_HUMAN] |
| P51957 | Serine/threonine-protein kinase Nek4 OS=Homo sapiens GN=NEK4 PE=1 SV=2 - [NEK4_HUMAN] |
| Q8TDX7 | Serine/threonine-protein kinase Nek7 OS=Homo sapiens GN=NEK7 PE=1 SV=1 - [NEK7_HUMAN] |
| O95747 | Serine/threonine-protein kinase OSR1 OS=Homo sapiens GN=OXSR1 PE=1 SV=1 - [OXSR1_HUMAN] |
| Q13153 | Serine/threonine-protein kinase PAK 1 OS=Homo sapiens GN=PAK1 PE=1 SV=2 - [PAK1_HUMAN] |
| O96013 | Serine/threonine-protein kinase PAK 4 OS=Homo sapiens GN=PAK4 PE=1 SV=1 - [PAK4_HUMAN] |
| Q9BVS4 | Serine/threonine-protein kinase RIO2 OS=Homo sapiens GN=RIOK2 PE=1 SV=2 - [RIOK2_HUMAN] |
| Q52WX2 | Serine/threonine-protein kinase SBK1 OS=Homo sapiens GN=SBK1 PE=2 SV=1 - [SBK1_HUMAN] |
| Q96Q15 | Serine/threonine-protein kinase SMG1 OS=Homo sapiens GN=SMG1 PE=1 SV=3 - [SMG1_HUMAN] |
| Q9H2K8 | Serine/threonine-protein kinase TAO3 OS=Homo sapiens GN=TAOK3 PE=1 SV=2 - [TAOK3_HUMAN] |
| O75385 | Serine/threonine-protein kinase ULK1 OS=Homo sapiens GN=ULK1 PE=1 SV=2 - [ULK1_HUMAN] |
| Q6PHR2 | Serine/threonine-protein kinase ULK3 OS=Homo sapiens GN=ULK3 PE=1 SV=2 - [ULK3_HUMAN] |
| Q9Y3S1 | Serine/threonine-protein kinase WNK2 OS=Homo sapiens GN=WNK2 PE=1 SV=4 - [WNK2_HUMAN] |
| Q16537 | Serine/threonine-protein phosphatase 2A 56 kDa regulatory subunit epsilon isoform OS=Homo sapiens GN=PPP2R5E PE=1 SV=1 - [2A5E_HUMAN] |
| Q13362 | Serine/threonine-protein phosphatase 2A 56 kDa regulatory subunit gamma isoform OS=Homo sapiens GN=PPP2R5C PE=1 SV=3 - [2A5G_HUMAN] |
| P48454 | Serine/threonine-protein phosphatase 2B catalytic subunit gamma isoform OS=Homo sapiens GN=PPP3CC PE=1 SV=3 - [PP2BC_HUMAN] |
| Q6NUP7 | Serine/threonine-protein phosphatase 4 regulatory subunit 4 OS=Homo sapiens GN=PPP4R4 PE=1 SV=1 - [PP4R4_HUMAN] |
| O00743 | Serine/threonine-protein phosphatase 6 catalytic subunit OS=Homo sapiens GN=PPP6C PE=1 SV=1 - [PPP6_HUMAN] |
| Q9UPN7 | Serine/threonine-protein phosphatase 6 regulatory subunit 1 OS=Homo sapiens GN=PPP6R1 PE=1 SV=5 - [PP6R1_HUMAN] |
| Q13315 | Serine-protein kinase ATM OS=Homo sapiens GN=ATM PE=1 SV=4 - [ATM_HUMAN] |
| P02787 | Serotransferrin OS=Homo sapiens GN=TF PE=1 SV=3 - [TRFE_HUMAN] |
| P29508 | Serpin B3 OS=Homo sapiens GN=SERPINB3 PE=1 SV=2 - [SPB3_HUMAN] |
| P50454 | Serpin H1 OS=Homo sapiens GN=SERPINH1 PE=1 SV=2 - [SERPH_HUMAN] |
| Q9NUC0 | SERTA domain-containing protein 4 OS=Homo sapiens GN=SERTAD4 PE=2 SV=1 - [SRTD4_HUMAN] |
| P02768 | Serum albumin OS=Homo sapiens GN=ALB PE=1 SV=2 - [ALBU_HUMAN] |
| Q15166 | Serum paraoxonase/lactonase 3 OS=Homo sapiens GN=PON3 PE=1 SV=3 - [PON3_HUMAN] |
| Q9Y6P5 | Sestrin-1 OS=Homo sapiens GN=SESN1 PE=1 SV=2 - [SESN1_HUMAN] |
| Q9NVD3 | SET domain-containing protein 4 OS=Homo sapiens GN=SETD4 PE=2 SV=1 - [SETD4_HUMAN] |
| Q9UBL3 | Set1/Ash2 histone methyltransferase complex subunit ASH2 OS=Homo sapiens GN=ASH2L PE=1 SV=1 - [ASH2L_HUMAN] |
| Q9UQR0 | Sex comb on midleg-like protein 2 OS=Homo sapiens GN=SCML2 PE=1 SV=1 - [SCML2_HUMAN] |
| Q8N228 | Sex comb on midleg-like protein 4 OS=Homo sapiens GN=SCML4 PE=1 SV=2 - [SCML4_HUMAN] |
| Q5VZ18 | SH2 domain-containing adapter protein E OS=Homo sapiens GN=SHE PE=1 SV=1 - [SHE_HUMAN] |
| Q9H788 | SH2 domain-containing protein 4A OS=Homo sapiens GN=SH2D4A PE=1 SV=1 - [SH24A_HUMAN] |
| Q9UPX8 | SH3 and multiple ankyrin repeat domains protein 2 OS=Homo sapiens GN=SHANK2 PE=1 SV=3 - [SHAN2_HUMAN] |
| Q9BYB0 | SH3 and multiple ankyrin repeat domains protein 3 OS=Homo sapiens GN=SHANK3 PE=1 SV=3 - [SHAN3_HUMAN] |
| Q5TCZ1 | SH3 and PX domain-containing protein 2A OS=Homo sapiens GN=SH3PXD2A PE=1 SV=1 - [SPD2A_HUMAN] |
| Q9Y3L3 | SH3 domain-binding protein 1 OS=Homo sapiens GN=SH3BP1 PE=1 SV=3 - [3BP1_HUMAN] |
| Q7L8J4 | SH3 domain-binding protein 5-like OS=Homo sapiens GN=SH3BP5L PE=1 SV=1 - [3BP5L_HUMAN] |
| A4FU49 | SH3 domain-containing protein 21 OS=Homo sapiens GN=SH3D21 PE=2 SV=2 - [SH321_HUMAN] |
| Q8NEM2 | SHC SH2 domain-binding protein 1 OS=Homo sapiens GN=SHCBP1 PE=1 SV=3 - [SHCBP_HUMAN] |
| Q9UIL1 | Short coiled-coil protein OS=Homo sapiens GN=SCOC PE=1 SV=2 - [SCOC_HUMAN] |
| A6NMB1 | Sialic acid-binding Ig-like lectin 16 OS=Homo sapiens GN=SIGLEC16 PE=2 SV=3 - [SIG16_HUMAN] |
| Q9NYZ4 | Sialic acid-binding Ig-like lectin 8 OS=Homo sapiens GN=SIGLEC8 PE=1 SV=2 - [SIGL8_HUMAN] |
| Q8WWR8 | Sialidase-4 OS=Homo sapiens GN=NEU4 PE=1 SV=3 - [NEUR4_HUMAN] |
| Q5T5P2 | Sickle tail protein homolog OS=Homo sapiens GN=KIAA1217 PE=1 SV=2 - [SKT_HUMAN] |
| Q96NB2 | Sideroflexin-2 OS=Homo sapiens GN=SFXN2 PE=1 SV=2 - [SFXN2_HUMAN] |
| Q9BWM7 | Sideroflexin-3 OS=Homo sapiens GN=SFXN3 PE=1 SV=2 - [SFXN3_HUMAN] |
| Q8TCT7 | Signal peptide peptidase-like 2B OS=Homo sapiens GN=SPPL2B PE=1 SV=2 - [SPP2B_HUMAN] |
| Q9Y5M8 | Signal recognition particle receptor subunit beta OS=Homo sapiens GN=SRPRB PE=1 SV=3 - [SRPRB_HUMAN] |
| P40763 | Signal transducer and activator of transcription 3 OS=Homo sapiens GN=STAT3 PE=1 SV=2 - [STAT3_HUMAN] |
| O43166 | Signal-induced proliferation-associated 1-like protein 1 OS=Homo sapiens GN=SIPA1L1 PE=1 SV=4 - [SI1L1_HUMAN] |
| Q9P2F8 | Signal-induced proliferation-associated 1-like protein 2 OS=Homo sapiens GN=SIPA1L2 PE=1 SV=2 - [SI1L2_HUMAN] |
| Q5JXA9 | Signal-regulatory protein beta-2 OS=Homo sapiens GN=SIRPB2 PE=2 SV=1 - [SIRB2_HUMAN] |
| Q9NTI5 | Sister chromatid cohesion protein PDS5 homolog B OS=Homo sapiens GN=PDS5B PE=1 SV=1 - [PDS5B_HUMAN] |
| Q9P0V8 | SLAM family member 8 OS=Homo sapiens GN=SLAMF8 PE=1 SV=1 - [SLAF8_HUMAN] |
| Q96PX8 | SLIT and NTRK-like protein 1 OS=Homo sapiens GN=SLITRK1 PE=1 SV=2 - [SLIK1_HUMAN] |
| Q9H5Y7 | SLIT and NTRK-like protein 6 OS=Homo sapiens GN=SLITRK6 PE=2 SV=3 - [SLIK6_HUMAN] |
| O75093 | Slit homolog 1 protein OS=Homo sapiens GN=SLIT1 PE=2 SV=4 - [SLIT1_HUMAN] |
| O75094 | Slit homolog 3 protein OS=Homo sapiens GN=SLIT3 PE=2 SV=3 - [SLIT3_HUMAN] |
| O75044 | SLIT-ROBO Rho GTPase-activating protein 2 OS=Homo sapiens GN=SRGAP2 PE=1 SV=2 - [SRGP2_HUMAN] |
| Q8TAD8 | Smad nuclear-interacting protein 1 OS=Homo sapiens GN=SNIP1 PE=1 SV=1 - [SNIP1_HUMAN] |
| Q9H2S1 | Small conductance calcium-activated potassium channel protein 2 OS=Homo sapiens GN=KCNN2 PE=1 SV=2 - [KCNN2_HUMAN] |
| P62306 | Small nuclear ribonucleoprotein F OS=Homo sapiens GN=SNRPF PE=1 SV=1 - [RUXF_HUMAN] |
| P62314 | Small nuclear ribonucleoprotein Sm D1 OS=Homo sapiens GN=SNRPD1 PE=1 SV=1 - [SMD1_HUMAN] |
| Q5T8I9 | Small RNA 2'-O-methyltransferase OS=Homo sapiens GN=HENMT1 PE=1 SV=1 - [HENMT_HUMAN] |
| Q8NHG7 | Small VCP/p97-interacting protein OS=Homo sapiens GN=SVIP PE=1 SV=1 - [SVIP_HUMAN] |
| Q9Y4D2 | Sn1-specific diacylglycerol lipase alpha OS=Homo sapiens GN=DAGLA PE=1 SV=3 - [DGLA_HUMAN] |
| Q9NRH2 | SNF-related serine/threonine-protein kinase OS=Homo sapiens GN=SNRK PE=1 SV=2 - [SNRK_HUMAN] |
| Q9Y6M7 | Sodium bicarbonate cotransporter 3 OS=Homo sapiens GN=SLC4A7 PE=1 SV=2 - [S4A7_HUMAN] |
| Q9NY46 | Sodium channel protein type 3 subunit alpha OS=Homo sapiens GN=SCN3A PE=1 SV=2 - [SCN3A_HUMAN] |
| Q9UPR5 | Sodium/calcium exchanger 2 OS=Homo sapiens GN=SLC8A2 PE=2 SV=2 - [NAC2_HUMAN] |
| P19634 | Sodium/hydrogen exchanger 1 OS=Homo sapiens GN=SLC9A1 PE=1 SV=2 - [SL9A1_HUMAN] |
| Q5TAH2 | Sodium/hydrogen exchanger 11 OS=Homo sapiens GN=SLC9C2 PE=2 SV=1 - [SL9C2_HUMAN] |
| P48764 | Sodium/hydrogen exchanger 3 OS=Homo sapiens GN=SLC9A3 PE=1 SV=2 - [SL9A3_HUMAN] |
| Q14940 | Sodium/hydrogen exchanger 5 OS=Homo sapiens GN=SLC9A5 PE=1 SV=2 - [SL9A5_HUMAN] |
| P13637 | Sodium/potassium-transporting ATPase subunit alpha-3 OS=Homo sapiens GN=ATP1A3 PE=1 SV=3 - [AT1A3_HUMAN] |
| Q9H2J7 | Sodium-dependent neutral amino acid transporter B(0)AT2 OS=Homo sapiens GN=SLC6A15 PE=1 SV=1 - [S6A15_HUMAN] |
| Q96N87 | Sodium-dependent neutral amino acid transporter B(0)AT3 OS=Homo sapiens GN=SLC6A18 PE=2 SV=2 - [S6A18_HUMAN] |
| Q9H2X9 | Solute carrier family 12 member 5 OS=Homo sapiens GN=SLC12A5 PE=2 SV=3 - [S12A5_HUMAN] |
| Q9BXP2 | Solute carrier family 12 member 9 OS=Homo sapiens GN=SLC12A9 PE=1 SV=1 - [S12A9_HUMAN] |
| O95528 | Solute carrier family 2, facilitated glucose transporter member 10 OS=Homo sapiens GN=SLC2A10 PE=1 SV=2 - [GTR10_HUMAN] |
| Q8TD20 | Solute carrier family 2, facilitated glucose transporter member 12 OS=Homo sapiens GN=SLC2A12 PE=2 SV=1 - [GTR12_HUMAN] |
| O15245 | Solute carrier family 22 member 1 OS=Homo sapiens GN=SLC22A1 PE=1 SV=2 - [S22A1_HUMAN] |
| A1A5C7 | Solute carrier family 22 member 23 OS=Homo sapiens GN=SLC22A23 PE=1 SV=2 - [S22AN_HUMAN] |
| Q8IVM8 | Solute carrier family 22 member 9 OS=Homo sapiens GN=SLC22A9 PE=1 SV=1 - [S22A9_HUMAN] |
| Q3KQZ1 | Solute carrier family 25 member 35 OS=Homo sapiens GN=SLC25A35 PE=2 SV=1 - [S2535_HUMAN] |
| Q96H78 | Solute carrier family 25 member 44 OS=Homo sapiens GN=SLC25A44 PE=2 SV=1 - [S2544_HUMAN] |
| Q8NG04 | Solute carrier family 26 member 10 OS=Homo sapiens GN=SLC26A10 PE=2 SV=1 - [S2610_HUMAN] |
| Q9NQQ7 | Solute carrier family 35 member C2 OS=Homo sapiens GN=SLC35C2 PE=1 SV=2 - [S35C2_HUMAN] |
| Q8WV83 | Solute carrier family 35 member F5 OS=Homo sapiens GN=SLC35F5 PE=2 SV=1 - [S35F5_HUMAN] |
| O94956 | Solute carrier organic anion transporter family member 2B1 OS=Homo sapiens GN=SLCO2B1 PE=1 SV=2 - [SO2B1_HUMAN] |
| Q9UIG8 | Solute carrier organic anion transporter family member 3A1 OS=Homo sapiens GN=SLCO3A1 PE=1 SV=3 - [SO3A1_HUMAN] |
| Q9H2Y9 | Solute carrier organic anion transporter family member 5A1 OS=Homo sapiens GN=SLCO5A1 PE=2 SV=2 - [SO5A1_HUMAN] |
| P01241 | Somatotropin OS=Homo sapiens GN=GH1 PE=1 SV=2 - [SOMA_HUMAN] |
| Q07889 | Son of sevenless homolog 1 OS=Homo sapiens GN=SOS1 PE=1 SV=1 - [SOS1_HUMAN] |
| Q9BX66 | Sorbin and SH3 domain-containing protein 1 OS=Homo sapiens GN=SORBS1 PE=1 SV=3 - [SRBS1_HUMAN] |
| Q00796 | Sorbitol dehydrogenase OS=Homo sapiens GN=SORD PE=1 SV=4 - [DHSO_HUMAN] |
| Q92673 | Sortilin-related receptor OS=Homo sapiens GN=SORL1 PE=1 SV=2 - [SORL_HUMAN] |
| Q9BW04 | Specifically androgen-regulated gene protein OS=Homo sapiens GN=SARG PE=1 SV=2 - [SARG_HUMAN] |
| P02549 | Spectrin alpha chain, erythrocytic 1 OS=Homo sapiens GN=SPTA1 PE=1 SV=5 - [SPTA1_HUMAN] |
| P11277 | Spectrin beta chain, erythrocytic OS=Homo sapiens GN=SPTB PE=1 SV=5 - [SPTB1_HUMAN] |
| Q01082 | Spectrin beta chain, non-erythrocytic 1 OS=Homo sapiens GN=SPTBN1 PE=1 SV=2 - [SPTB2_HUMAN] |
| Q9H254 | Spectrin beta chain, non-erythrocytic 4 OS=Homo sapiens GN=SPTBN4 PE=1 SV=2 - [SPTN4_HUMAN] |
| Q9NRC6 | Spectrin beta chain, non-erythrocytic 5 OS=Homo sapiens GN=SPTBN5 PE=1 SV=2 - [SPTN5_HUMAN] |
| Q8NFV5 | Speedy protein E1 OS=Homo sapiens GN=SPDYE1 PE=2 SV=3 - [SPDE1_HUMAN] |
| Q5VSR9 | Sperm protein associated with the nucleus on the X chromosome N1 OS=Homo sapiens GN=SPANXN1 PE=3 SV=1 - [SPXN1_HUMAN] |
| Q07617 | Sperm-associated antigen 1 OS=Homo sapiens GN=SPAG1 PE=1 SV=3 - [SPAG1_HUMAN] |
| P09430 | Spermatid nuclear transition protein 1 OS=Homo sapiens GN=TNP1 PE=1 SV=2 - [STP1_HUMAN] |
| Q96SI9 | Spermatid perinuclear RNA-binding protein OS=Homo sapiens GN=STRBP PE=1 SV=1 - [STRBP_HUMAN] |
| Q9BXB7 | Spermatogenesis-associated protein 16 OS=Homo sapiens GN=SPATA16 PE=1 SV=3 - [SPT16_HUMAN] |
| Q8TB22 | Spermatogenesis-associated protein 20 OS=Homo sapiens GN=SPATA20 PE=2 SV=3 - [SPT20_HUMAN] |
| Q5VYP0 | Spermatogenesis-associated protein 31A3 OS=Homo sapiens GN=SPATA31A3 PE=2 SV=1 - [S31A3_HUMAN] |
| Q96LK8 | Spermatogenesis-associated protein 32 OS=Homo sapiens GN=SPATA32 PE=1 SV=3 - [SPT32_HUMAN] |
| Q96N06 | Spermatogenesis-associated protein 33 OS=Homo sapiens GN=SPATA33 PE=2 SV=1 - [SPT33_HUMAN] |
| Q537H7 | Spermatogenesis-associated protein 45 OS=Homo sapiens GN=SPATA45 PE=3 SV=1 - [SPT45_HUMAN] |
| Q9BVQ7 | Spermatogenesis-associated protein 5-like protein 1 OS=Homo sapiens GN=SPATA5L1 PE=1 SV=2 - [SPA5L_HUMAN] |
| P28290 | Sperm-specific antigen 2 OS=Homo sapiens GN=SSFA2 PE=1 SV=3 - [SSFA2_HUMAN] |
| Q9NXE4 | Sphingomyelin phosphodiesterase 4 OS=Homo sapiens GN=SMPD4 PE=1 SV=2 - [NSMA3_HUMAN] |
| O95977 | Sphingosine 1-phosphate receptor 4 OS=Homo sapiens GN=S1PR4 PE=1 SV=1 - [S1PR4_HUMAN] |
| Q9H228 | Sphingosine 1-phosphate receptor 5 OS=Homo sapiens GN=S1PR5 PE=2 SV=1 - [S1PR5_HUMAN] |
| Q8N0Z3 | Spindle and centriole-associated protein 1 OS=Homo sapiens GN=SPICE1 PE=1 SV=1 - [SPICE_HUMAN] |
| Q96BD8 | Spindle and kinetochore-associated protein 1 OS=Homo sapiens GN=SKA1 PE=1 SV=1 - [SKA1_HUMAN] |
| Q6UVJ0 | Spindle assembly abnormal protein 6 homolog OS=Homo sapiens GN=SASS6 PE=1 SV=1 - [SAS6_HUMAN] |
| Q99865 | Spindlin-2A OS=Homo sapiens GN=SPIN2A PE=1 SV=3 - [SPI2A_HUMAN] |
| Q9H7N4 | Splicing factor, arginine/serine-rich 19 OS=Homo sapiens GN=SCAF1 PE=1 SV=3 - [SFR19_HUMAN] |
| Q6ZMY3 | SPOC domain-containing protein 1 OS=Homo sapiens GN=SPOCD1 PE=2 SV=1 - [SPOC1_HUMAN] |
| Q8WW59 | SPRY domain-containing protein 4 OS=Homo sapiens GN=SPRYD4 PE=1 SV=2 - [SPRY4_HUMAN] |
| Q6PJ21 | SPRY domain-containing SOCS box protein 3 OS=Homo sapiens GN=SPSB3 PE=1 SV=2 - [SPSB3_HUMAN] |
| Q8WWQ8 | Stabilin-2 OS=Homo sapiens GN=STAB2 PE=1 SV=3 - [STAB2_HUMAN] |
| Q14849 | StAR-related lipid transfer protein 3 OS=Homo sapiens GN=STARD3 PE=1 SV=2 - [STAR3_HUMAN] |
| Q92502 | StAR-related lipid transfer protein 8 OS=Homo sapiens GN=STARD8 PE=1 SV=2 - [STAR8_HUMAN] |
| Q9P2P6 | StAR-related lipid transfer protein 9 OS=Homo sapiens GN=STARD9 PE=1 SV=3 - [STAR9_HUMAN] |
| Q6SZW1 | Sterile alpha and TIR motif-containing protein 1 OS=Homo sapiens GN=SARM1 PE=1 SV=1 - [SARM1_HUMAN] |
| Q8N6K7 | Sterile alpha motif domain-containing protein 3 OS=Homo sapiens GN=SAMD3 PE=1 SV=2 - [SAMD3_HUMAN] |
| Q7Z3H4 | Sterile alpha motif domain-containing protein 7 OS=Homo sapiens GN=SAMD7 PE=2 SV=1 - [SAMD7_HUMAN] |
| Q5K651 | Sterile alpha motif domain-containing protein 9 OS=Homo sapiens GN=SAMD9 PE=1 SV=1 - [SAMD9_HUMAN] |
| P49675 | Steroidogenic acute regulatory protein, mitochondrial OS=Homo sapiens GN=STAR PE=1 SV=2 - [STAR_HUMAN] |
| Q02318 | Sterol 26-hydroxylase, mitochondrial OS=Homo sapiens GN=CYP27A1 PE=1 SV=1 - [CP27A_HUMAN] |
| Q86WV6 | Stimulator of interferon genes protein OS=Homo sapiens GN=TMEM173 PE=1 SV=1 - [STING_HUMAN] |
| Q9UBI4 | Stomatin-like protein 1 OS=Homo sapiens GN=STOML1 PE=1 SV=1 - [STML1_HUMAN] |
| Q5VSL9 | Striatin-interacting protein 1 OS=Homo sapiens GN=STRIP1 PE=1 SV=1 - [STRP1_HUMAN] |
| Q9UQE7 | Structural maintenance of chromosomes protein 3 OS=Homo sapiens GN=SMC3 PE=1 SV=2 - [SMC3_HUMAN] |
| Q96SB8 | Structural maintenance of chromosomes protein 6 OS=Homo sapiens GN=SMC6 PE=1 SV=2 - [SMC6_HUMAN] |
| Q9NX18 | Succinate dehydrogenase assembly factor 2, mitochondrial OS=Homo sapiens GN=SDHAF2 PE=1 SV=1 - [SDHF2_HUMAN] |
| Q99643 | Succinate dehydrogenase cytochrome b560 subunit, mitochondrial OS=Homo sapiens GN=SDHC PE=1 SV=1 - [C560_HUMAN] |
| P51649 | Succinate-semialdehyde dehydrogenase, mitochondrial OS=Homo sapiens GN=ALDH5A1 PE=1 SV=2 - [SSDH_HUMAN] |
| Q9H2B4 | Sulfate anion transporter 1 OS=Homo sapiens GN=SLC26A1 PE=1 SV=2 - [S26A1_HUMAN] |
| O00391 | Sulfhydryl oxidase 1 OS=Homo sapiens GN=QSOX1 PE=1 SV=3 - [QSOX1_HUMAN] |
| P50225 | Sulfotransferase 1A1 OS=Homo sapiens GN=SULT1A1 PE=1 SV=3 - [ST1A1_HUMAN] |
| Q9UBT2 | SUMO-activating enzyme subunit 2 OS=Homo sapiens GN=UBA2 PE=1 SV=2 - [SAE2_HUMAN] |
| O14544 | Suppressor of cytokine signaling 6 OS=Homo sapiens GN=SOCS6 PE=1 SV=2 - [SOCS6_HUMAN] |
| O75683 | Surfeit locus protein 6 OS=Homo sapiens GN=SURF6 PE=1 SV=3 - [SURF6_HUMAN] |
| Q6UWL2 | Sushi domain-containing protein 1 OS=Homo sapiens GN=SUSD1 PE=1 SV=1 - [SUSD1_HUMAN] |
| O60279 | Sushi domain-containing protein 5 OS=Homo sapiens GN=SUSD5 PE=1 SV=3 - [SUSD5_HUMAN] |
| Q9H4L7 | SWI/SNF-related matrix-associated actin-dependent regulator of chromatin subfamily A containing DEAD/H box 1 OS=Homo sapiens GN=SMARCAD1 PE=1 SV=2 - [SMRCD_HUMAN] |
| Q9NZC9 | SWI/SNF-related matrix-associated actin-dependent regulator of chromatin subfamily A-like protein 1 OS=Homo sapiens GN=SMARCAL1 PE=1 SV=1 - [SMAL1_HUMAN] |
| A6NDD5 | Synapse differentiation-inducing gene protein 1-like OS=Homo sapiens GN=SYNDIG1L PE=3 SV=1 - [SYN1L_HUMAN] |
| Q8N4V2 | Synaptic vesicle 2-related protein OS=Homo sapiens GN=SVOP PE=2 SV=1 - [SVOP_HUMAN] |
| Q15431 | Synaptonemal complex protein 1 OS=Homo sapiens GN=SYCP1 PE=1 SV=2 - [SYCP1_HUMAN] |
| Q8IZU3 | Synaptonemal complex protein 3 OS=Homo sapiens GN=SYCP3 PE=1 SV=1 - [SYCP3_HUMAN] |
| Q6XYQ8 | Synaptotagmin-10 OS=Homo sapiens GN=SYT10 PE=2 SV=1 - [SYT10_HUMAN] |
| Q9BQS2 | Synaptotagmin-15 OS=Homo sapiens GN=SYT15 PE=2 SV=3 - [SYT15_HUMAN] |
| Q9BSW7 | Synaptotagmin-17 OS=Homo sapiens GN=SYT17 PE=1 SV=1 - [SYT17_HUMAN] |
| Q9HCH5 | Synaptotagmin-like protein 2 OS=Homo sapiens GN=SYTL2 PE=1 SV=3 - [SYTL2_HUMAN] |
| Q9NPQ8 | Synembryn-A OS=Homo sapiens GN=RIC8A PE=1 SV=3 - [RIC8A_HUMAN] |
| Q9NX95 | Syntabulin OS=Homo sapiens GN=SYBU PE=1 SV=2 - [SYBU_HUMAN] |
| Q13277 | Syntaxin-3 OS=Homo sapiens GN=STX3 PE=1 SV=3 - [STX3_HUMAN] |
| Q5T5C0 | Syntaxin-binding protein 5 OS=Homo sapiens GN=STXBP5 PE=1 SV=1 - [STXB5_HUMAN] |
| Q9UHF0 | Tachykinin-3 OS=Homo sapiens GN=TAC3 PE=1 SV=1 - [TKNK_HUMAN] |
| Q9Y490 | Talin-1 OS=Homo sapiens GN=TLN1 PE=1 SV=3 - [TLN1_HUMAN] |
| Q9Y4G6 | Talin-2 OS=Homo sapiens GN=TLN2 PE=1 SV=4 - [TLN2_HUMAN] |
| A7MCY6 | TANK-binding kinase 1-binding protein 1 OS=Homo sapiens GN=TBKBP1 PE=1 SV=1 - [TBKB1_HUMAN] |
| P59541 | Taste receptor type 2 member 30 OS=Homo sapiens GN=TAS2R30 PE=2 SV=3 - [T2R30_HUMAN] |
| P59538 | Taste receptor type 2 member 31 OS=Homo sapiens GN=TAS2R31 PE=2 SV=2 - [T2R31_HUMAN] |
| P59551 | Taste receptor type 2 member 60 OS=Homo sapiens GN=TAS2R60 PE=2 SV=1 - [T2R60_HUMAN] |
| P82094 | TATA element modulatory factor OS=Homo sapiens GN=TMF1 PE=1 SV=2 - [TMF1_HUMAN] |
| O14981 | TATA-binding protein-associated factor 172 OS=Homo sapiens GN=BTAF1 PE=1 SV=2 - [BTAF1_HUMAN] |
| Q5TCY1 | Tau-tubulin kinase 1 OS=Homo sapiens GN=TTBK1 PE=1 SV=2 - [TTBK1_HUMAN] |
| Q8TEA7 | TBC domain-containing protein kinase-like protein OS=Homo sapiens GN=TBCK PE=1 SV=4 - [TBCK_HUMAN] |
| Q9ULP9 | TBC1 domain family member 24 OS=Homo sapiens GN=TBC1D24 PE=1 SV=2 - [TBC24_HUMAN] |
| Q9UPU7 | TBC1 domain family member 2B OS=Homo sapiens GN=TBC1D2B PE=1 SV=2 - [TBD2B_HUMAN] |
| Q7Z3E1 | TCDD-inducible poly [ADP-ribose] polymerase OS=Homo sapiens GN=TIPARP PE=2 SV=1 - [PARPT_HUMAN] |
| Q8N3R3 | T-cell activation inhibitor, mitochondrial OS=Homo sapiens GN=TCAIM PE=2 SV=2 - [TCAIM_HUMAN] |
| Q8N103 | T-cell activation Rho GTPase-activating protein OS=Homo sapiens GN=TAGAP PE=1 SV=1 - [TAGAP_HUMAN] |
| P06729 | T-cell surface antigen CD2 OS=Homo sapiens GN=CD2 PE=1 SV=2 - [CD2_HUMAN] |
| P01730 | T-cell surface glycoprotein CD4 OS=Homo sapiens GN=CD4 PE=1 SV=1 - [CD4_HUMAN] |
| Q5JU00 | T-complex-associated testis-expressed protein 1 OS=Homo sapiens GN=TCTE1 PE=2 SV=1 - [TCTE1_HUMAN] |
| Q6ZSZ6 | Teashirt homolog 1 OS=Homo sapiens GN=TSHZ1 PE=2 SV=2 - [TSH1_HUMAN] |
| Q63HK5 | Teashirt homolog 3 OS=Homo sapiens GN=TSHZ3 PE=1 SV=2 - [TSH3_HUMAN] |
| Q7Z6L1 | Tectonin beta-propeller repeat-containing protein 1 OS=Homo sapiens GN=TECPR1 PE=1 SV=1 - [TCPR1_HUMAN] |
| Q86US8 | Telomerase-binding protein EST1A OS=Homo sapiens GN=SMG6 PE=1 SV=2 - [EST1A_HUMAN] |
| Q6N022 | Teneurin-4 OS=Homo sapiens GN=TENM4 PE=1 SV=2 - [TEN4_HUMAN] |
| Q68CZ2 | Tensin-3 OS=Homo sapiens GN=TNS3 PE=1 SV=2 - [TENS3_HUMAN] |
| Q5TAX3 | Terminal uridylyltransferase 4 OS=Homo sapiens GN=ZCCHC11 PE=1 SV=3 - [TUT4_HUMAN] |
| Q9BXT5 | Testis-expressed sequence 15 protein OS=Homo sapiens GN=TEX15 PE=2 SV=2 - [TEX15_HUMAN] |
| Q8IWB9 | Testis-expressed sequence 2 protein OS=Homo sapiens GN=TEX2 PE=1 SV=2 - [TEX2_HUMAN] |
| Q9BZW7 | Testis-specific gene 10 protein OS=Homo sapiens GN=TSGA10 PE=1 SV=1 - [TSG10_HUMAN] |
| Q9BXA7 | Testis-specific serine/threonine-protein kinase 1 OS=Homo sapiens GN=TSSK1B PE=1 SV=1 - [TSSK1_HUMAN] |
| Q9BZE9 | Tether containing UBX domain for GLUT4 OS=Homo sapiens GN=ASPSCR1 PE=1 SV=1 - [ASPC1_HUMAN] |
| Q9UKR8 | Tetraspanin-16 OS=Homo sapiens GN=TSPAN16 PE=2 SV=1 - [TSN16_HUMAN] |
| Q8NEE8 | Tetratricopeptide repeat protein 16 OS=Homo sapiens GN=TTC16 PE=2 SV=2 - [TTC16_HUMAN] |
| Q8NDW8 | Tetratricopeptide repeat protein 21A OS=Homo sapiens GN=TTC21A PE=2 SV=3 - [TT21A_HUMAN] |
| Q7Z4L5 | Tetratricopeptide repeat protein 21B OS=Homo sapiens GN=TTC21B PE=1 SV=2 - [TT21B_HUMAN] |
| Q96AY4 | Tetratricopeptide repeat protein 28 OS=Homo sapiens GN=TTC28 PE=1 SV=4 - [TTC28_HUMAN] |
| A8MYJ7 | Tetratricopeptide repeat protein 34 OS=Homo sapiens GN=TTC34 PE=2 SV=2 - [TTC34_HUMAN] |
| Q5R3I4 | Tetratricopeptide repeat protein 38 OS=Homo sapiens GN=TTC38 PE=1 SV=1 - [TTC38_HUMAN] |
| Q8N0Z6 | Tetratricopeptide repeat protein 5 OS=Homo sapiens GN=TTC5 PE=1 SV=2 - [TTC5_HUMAN] |
| Q8TAM2 | Tetratricopeptide repeat protein 8 OS=Homo sapiens GN=TTC8 PE=1 SV=2 - [TTC8_HUMAN] |
| O95411 | TGFB1-induced anti-apoptotic factor 1 OS=Homo sapiens GN=TIAF1 PE=2 SV=2 - [TIAF1_HUMAN] |
| P36897 | TGF-beta receptor type-1 OS=Homo sapiens GN=TGFBR1 PE=1 SV=1 - [TGFR1_HUMAN] |
| Q8WTV1 | THAP domain-containing protein 3 OS=Homo sapiens GN=THAP3 PE=1 SV=1 - [THAP3_HUMAN] |
| Q8WY91 | THAP domain-containing protein 4 OS=Homo sapiens GN=THAP4 PE=1 SV=2 - [THAP4_HUMAN] |
| Q96J42 | Thioredoxin domain-containing protein 15 OS=Homo sapiens GN=TXNDC15 PE=1 SV=1 - [TXD15_HUMAN] |
| Q9P2K2 | Thioredoxin domain-containing protein 16 OS=Homo sapiens GN=TXNDC16 PE=2 SV=4 - [TXD16_HUMAN] |
| Q9H3M7 | Thioredoxin-interacting protein OS=Homo sapiens GN=TXNIP PE=1 SV=1 - [TXNIP_HUMAN] |
| Q8NI27 | THO complex subunit 2 OS=Homo sapiens GN=THOC2 PE=1 SV=2 - [THOC2_HUMAN] |
| P26639 | Threonine--tRNA ligase, cytoplasmic OS=Homo sapiens GN=TARS PE=1 SV=3 - [SYTC_HUMAN] |
| P40225 | Thrombopoietin OS=Homo sapiens GN=THPO PE=1 SV=1 - [TPO_HUMAN] |
| P21731 | Thromboxane A2 receptor OS=Homo sapiens GN=TBXA2R PE=1 SV=3 - [TA2R_HUMAN] |
| Q9BV44 | THUMP domain-containing protein 3 OS=Homo sapiens GN=THUMPD3 PE=1 SV=1 - [THUM3_HUMAN] |
| O00142 | Thymidine kinase 2, mitochondrial OS=Homo sapiens GN=TK2 PE=1 SV=4 - [KITM_HUMAN] |
| P19971 | Thymidine phosphorylase OS=Homo sapiens GN=TYMP PE=1 SV=2 - [TYPH_HUMAN] |
| P23919 | Thymidylate kinase OS=Homo sapiens GN=DTYMK PE=1 SV=4 - [KTHY_HUMAN] |
| Q6YHU6 | Thyroid adenoma-associated protein OS=Homo sapiens GN=THADA PE=1 SV=1 - [THADA_HUMAN] |
| Q96MW7 | Tigger transposable element-derived protein 1 OS=Homo sapiens GN=TIGD1 PE=1 SV=1 - [TIGD1_HUMAN] |
| Q8IY51 | Tigger transposable element-derived protein 4 OS=Homo sapiens GN=TIGD4 PE=2 SV=2 - [TIGD4_HUMAN] |
| Q07157 | Tight junction protein ZO-1 OS=Homo sapiens GN=TJP1 PE=1 SV=3 - [ZO1_HUMAN] |
| Q9UDY2 | Tight junction protein ZO-2 OS=Homo sapiens GN=TJP2 PE=1 SV=2 - [ZO2_HUMAN] |
| O95049 | Tight junction protein ZO-3 OS=Homo sapiens GN=TJP3 PE=1 SV=3 - [ZO3_HUMAN] |
| Q8WZ42 | Titin OS=Homo sapiens GN=TTN PE=1 SV=4 - [TITIN_HUMAN] |
| Q6P9B6 | TLD domain-containing protein 1 OS=Homo sapiens GN=TLDC1 PE=1 SV=2 - [TLDC1_HUMAN] |
| Q96KP6 | TNFAIP3-interacting protein 3 OS=Homo sapiens GN=TNIP3 PE=1 SV=2 - [TNIP3_HUMAN] |
| Q15399 | Toll-like receptor 1 OS=Homo sapiens GN=TLR1 PE=1 SV=3 - [TLR1_HUMAN] |
| Q9BXR5 | Toll-like receptor 10 OS=Homo sapiens GN=TLR10 PE=1 SV=2 - [TLR10_HUMAN] |
| Q9Y2C9 | Toll-like receptor 6 OS=Homo sapiens GN=TLR6 PE=1 SV=2 - [TLR6_HUMAN] |
| Q96HA7 | Tonsoku-like protein OS=Homo sapiens GN=TONSL PE=1 SV=2 - [TONSL_HUMAN] |
| Q96RI8 | Trace amine-associated receptor 6 OS=Homo sapiens GN=TAAR6 PE=2 SV=1 - [TAAR6_HUMAN] |
| Q9Y228 | TRAF3-interacting JNK-activating modulator OS=Homo sapiens GN=TRAF3IP3 PE=1 SV=2 - [T3JAM_HUMAN] |
| P48553 | Trafficking protein particle complex subunit 10 OS=Homo sapiens GN=TRAPPC10 PE=1 SV=2 - [TPC10_HUMAN] |
| Q7Z392 | Trafficking protein particle complex subunit 11 OS=Homo sapiens GN=TRAPPC11 PE=1 SV=2 - [TPC11_HUMAN] |
| Q8WVT3 | Trafficking protein particle complex subunit 12 OS=Homo sapiens GN=TRAPPC12 PE=1 SV=3 - [TPC12_HUMAN] |
| Q9Y2L5 | Trafficking protein particle complex subunit 8 OS=Homo sapiens GN=TRAPPC8 PE=1 SV=2 - [TPPC8_HUMAN] |
| Q96EI5 | Transcription elongation factor A protein-like 4 OS=Homo sapiens GN=TCEAL4 PE=1 SV=2 - [TCAL4_HUMAN] |
| O14776 | Transcription elongation regulator 1 OS=Homo sapiens GN=TCERG1 PE=1 SV=2 - [TCRG1_HUMAN] |
| Q9UGU0 | Transcription factor 20 OS=Homo sapiens GN=TCF20 PE=1 SV=3 - [TCF20_HUMAN] |
| Q7RTU1 | Transcription factor 23 OS=Homo sapiens GN=TCF23 PE=2 SV=1 - [TCF23_HUMAN] |
| Q00059 | Transcription factor A, mitochondrial OS=Homo sapiens GN=TFAM PE=1 SV=1 - [TFAM_HUMAN] |
| O75461 | Transcription factor E2F6 OS=Homo sapiens GN=E2F6 PE=1 SV=1 - [E2F6_HUMAN] |
| Q96AV8 | Transcription factor E2F7 OS=Homo sapiens GN=E2F7 PE=1 SV=3 - [E2F7_HUMAN] |
| Q9HCC6 | Transcription factor HES-4 OS=Homo sapiens GN=HES4 PE=2 SV=1 - [HES4_HUMAN] |
| Q5T1R4 | Transcription factor HIVEP3 OS=Homo sapiens GN=HIVEP3 PE=2 SV=1 - [ZEP3_HUMAN] |
| P48380 | Transcription factor RFX3 OS=Homo sapiens GN=RFX3 PE=1 SV=2 - [RFX3_HUMAN] |
| O94993 | Transcription factor SOX-30 OS=Homo sapiens GN=SOX30 PE=1 SV=1 - [SOX30_HUMAN] |
| P35711 | Transcription factor SOX-5 OS=Homo sapiens GN=SOX5 PE=1 SV=3 - [SOX5_HUMAN] |
| P08047 | Transcription factor Sp1 OS=Homo sapiens GN=SP1 PE=1 SV=3 - [SP1_HUMAN] |
| A6H8Y1 | Transcription factor TFIIIB component B'' homolog OS=Homo sapiens GN=BDP1 PE=1 SV=3 - [BDP1_HUMAN] |
| Q12962 | Transcription initiation factor TFIID subunit 10 OS=Homo sapiens GN=TAF10 PE=1 SV=1 - [TAF10_HUMAN] |
| O15164 | Transcription intermediary factor 1-alpha OS=Homo sapiens GN=TRIM24 PE=1 SV=3 - [TIF1A_HUMAN] |
| Q49AM1 | Transcription termination factor 2, mitochondrial OS=Homo sapiens GN=MTERF2 PE=1 SV=2 - [MTEF2_HUMAN] |
| Q86TJ2 | Transcriptional adapter 2-beta OS=Homo sapiens GN=TADA2B PE=1 SV=2 - [TAD2B_HUMAN] |
| Q15562 | Transcriptional enhancer factor TEF-4 OS=Homo sapiens GN=TEAD2 PE=1 SV=2 - [TEAD2_HUMAN] |
| Q04724 | Transducin-like enhancer protein 1 OS=Homo sapiens GN=TLE1 PE=1 SV=2 - [TLE1_HUMAN] |
| Q04726 | Transducin-like enhancer protein 3 OS=Homo sapiens GN=TLE3 PE=1 SV=2 - [TLE3_HUMAN] |
| Q9Y4A5 | Transformation/transcription domain-associated protein OS=Homo sapiens GN=TRRAP PE=1 SV=3 - [TRRAP_HUMAN] |
| Q13595 | Transformer-2 protein homolog alpha OS=Homo sapiens GN=TRA2A PE=1 SV=1 - [TRA2A_HUMAN] |
| Q01995 | Transgelin OS=Homo sapiens GN=TAGLN PE=1 SV=4 - [TAGL_HUMAN] |
| O94759 | Transient receptor potential cation channel subfamily M member 2 OS=Homo sapiens GN=TRPM2 PE=1 SV=2 - [TRPM2_HUMAN] |
| Q8TD43 | Transient receptor potential cation channel subfamily M member 4 OS=Homo sapiens GN=TRPM4 PE=1 SV=1 - [TRPM4_HUMAN] |
| Q7Z2W7 | Transient receptor potential cation channel subfamily M member 8 OS=Homo sapiens GN=TRPM8 PE=1 SV=2 - [TRPM8_HUMAN] |
| Q8NET8 | Transient receptor potential cation channel subfamily V member 3 OS=Homo sapiens GN=TRPV3 PE=1 SV=2 - [TRPV3_HUMAN] |
| P51854 | Transketolase-like protein 1 OS=Homo sapiens GN=TKTL1 PE=1 SV=2 - [TKTL1_HUMAN] |
| P46199 | Translation initiation factor IF-2, mitochondrial OS=Homo sapiens GN=MTIF2 PE=1 SV=2 - [IF2M_HUMAN] |
| Q8N609 | Translocating chain-associated membrane protein 1-like 1 OS=Homo sapiens GN=TRAM1L1 PE=2 SV=2 - [TR1L1_HUMAN] |
| Q8N6Q1 | Transmembrane and coiled-coil domain-containing protein 5A OS=Homo sapiens GN=TMCO5A PE=2 SV=2 - [TMC5A_HUMAN] |
| O75069 | Transmembrane and coiled-coil domains protein 2 OS=Homo sapiens GN=TMCC2 PE=1 SV=3 - [TMCC2_HUMAN] |
| Q6NXT6 | Transmembrane anterior posterior transformation protein 1 homolog OS=Homo sapiens GN=TAPT1 PE=1 SV=1 - [TAPT1_HUMAN] |
| Q8IU68 | Transmembrane channel-like protein 8 OS=Homo sapiens GN=TMC8 PE=1 SV=1 - [TMC8_HUMAN] |
| Q9BZD6 | Transmembrane gamma-carboxyglutamic acid protein 4 OS=Homo sapiens GN=PRRG4 PE=1 SV=1 - [TMG4_HUMAN] |
| Q6ZMR5 | Transmembrane protease serine 11A OS=Homo sapiens GN=TMPRSS11A PE=1 SV=1 - [TM11A_HUMAN] |
| Q86WS5 | Transmembrane protease serine 12 OS=Homo sapiens GN=TMPRSS12 PE=1 SV=2 - [TMPSC_HUMAN] |
| Q9BYE2 | Transmembrane protease serine 13 OS=Homo sapiens GN=TMPRSS13 PE=2 SV=4 - [TMPSD_HUMAN] |
| P57727 | Transmembrane protease serine 3 OS=Homo sapiens GN=TMPRSS3 PE=1 SV=2 - [TMPS3_HUMAN] |
| Q96IK0 | Transmembrane protein 101 OS=Homo sapiens GN=TMEM101 PE=1 SV=1 - [TM101_HUMAN] |
| Q9BVC6 | Transmembrane protein 109 OS=Homo sapiens GN=TMEM109 PE=1 SV=1 - [TM109_HUMAN] |
| Q86TL2 | Transmembrane protein 110 OS=Homo sapiens GN=TMEM110 PE=2 SV=1 - [TM110_HUMAN] |
| Q96AN5 | Transmembrane protein 143 OS=Homo sapiens GN=TMEM143 PE=2 SV=1 - [TM143_HUMAN] |
| Q8N614 | Transmembrane protein 156 OS=Homo sapiens GN=TMEM156 PE=2 SV=2 - [TM156_HUMAN] |
| Q9H0V1 | Transmembrane protein 168 OS=Homo sapiens GN=TMEM168 PE=2 SV=2 - [TM168_HUMAN] |
| Q96HH4 | Transmembrane protein 169 OS=Homo sapiens GN=TMEM169 PE=2 SV=1 - [TM169_HUMAN] |
| Q9BSA9 | Transmembrane protein 175 OS=Homo sapiens GN=TMEM175 PE=1 SV=1 - [TM175_HUMAN] |
| Q9H0A3 | Transmembrane protein 191A OS=Homo sapiens GN=TMEM191A PE=2 SV=1 - [T191A_HUMAN] |
| Q69YZ2 | Transmembrane protein 200B OS=Homo sapiens GN=TMEM200B PE=2 SV=1 - [T200B_HUMAN] |
| Q9H6L2 | Transmembrane protein 231 OS=Homo sapiens GN=TMEM231 PE=1 SV=1 - [TM231_HUMAN] |
| C9JQI7 | Transmembrane protein 232 OS=Homo sapiens GN=TMEM232 PE=2 SV=2 - [TM232_HUMAN] |
| Q9NWH2 | Transmembrane protein 242 OS=Homo sapiens GN=TMEM242 PE=1 SV=1 - [TM242_HUMAN] |
| Q9BRR3 | Transmembrane protein 246 OS=Homo sapiens GN=TMEM246 PE=1 SV=1 - [TM246_HUMAN] |
| Q2WGJ8 | Transmembrane protein 249 OS=Homo sapiens GN=TMEM249 PE=2 SV=1 - [TM249_HUMAN] |
| Q96HV5 | Transmembrane protein 41A OS=Homo sapiens GN=TMEM41A PE=1 SV=1 - [TM41A_HUMAN] |
| Q6P2H8 | Transmembrane protein 53 OS=Homo sapiens GN=TMEM53 PE=2 SV=1 - [TMM53_HUMAN] |
| Q0P6H9 | Transmembrane protein 62 OS=Homo sapiens GN=TMEM62 PE=1 SV=1 - [TMM62_HUMAN] |
| Q9BSE2 | Transmembrane protein 79 OS=Homo sapiens GN=TMEM79 PE=1 SV=1 - [TMM79_HUMAN] |
| Q9P0T7 | Transmembrane protein 9 OS=Homo sapiens GN=TMEM9 PE=1 SV=1 - [TMEM9_HUMAN] |
| Q2M3C6 | Transmembrane protein C15orf27 OS=Homo sapiens GN=C15orf27 PE=2 SV=2 - [CO027_HUMAN] |
| O75949 | Transmembrane protein FAM155B OS=Homo sapiens GN=FAM155B PE=2 SV=2 - [F155B_HUMAN] |
| Q13428 | Treacle protein OS=Homo sapiens GN=TCOF1 PE=1 SV=3 - [TCOF_HUMAN] |
| Q13061 | Triadin OS=Homo sapiens GN=TRDN PE=1 SV=4 - [TRDN_HUMAN] |
| P40939 | Trifunctional enzyme subunit alpha, mitochondrial OS=Homo sapiens GN=HADHA PE=1 SV=2 - [ECHA_HUMAN] |
| Q9H2D6 | TRIO and F-actin-binding protein OS=Homo sapiens GN=TRIOBP PE=1 SV=3 - [TARA_HUMAN] |
| P60174 | Triosephosphate isomerase OS=Homo sapiens GN=TPI1 PE=1 SV=3 - [TPIS_HUMAN] |
| Q9C040 | Tripartite motif-containing protein 2 OS=Homo sapiens GN=TRIM2 PE=1 SV=1 - [TRIM2_HUMAN] |
| Q14134 | Tripartite motif-containing protein 29 OS=Homo sapiens GN=TRIM29 PE=1 SV=2 - [TRI29_HUMAN] |
| O75382 | Tripartite motif-containing protein 3 OS=Homo sapiens GN=TRIM3 PE=1 SV=2 - [TRIM3_HUMAN] |
| Q9UPQ4 | Tripartite motif-containing protein 35 OS=Homo sapiens GN=TRIM35 PE=1 SV=2 - [TRI35_HUMAN] |
| O15016 | Tripartite motif-containing protein 66 OS=Homo sapiens GN=TRIM66 PE=2 SV=4 - [TRI66_HUMAN] |
| Q9C029 | Tripartite motif-containing protein 7 OS=Homo sapiens GN=TRIM7 PE=1 SV=2 - [TRIM7_HUMAN] |
| O14773 | Tripeptidyl-peptidase 1 OS=Homo sapiens GN=TPP1 PE=1 SV=2 - [TPP1_HUMAN] |
| Q9UBP6 | tRNA (guanine-N(7)-)-methyltransferase OS=Homo sapiens GN=METTL1 PE=1 SV=1 - [TRMB_HUMAN] |
| Q6PF06 | tRNA methyltransferase 10 homolog B OS=Homo sapiens GN=TRMT10B PE=2 SV=1 - [TM10B_HUMAN] |
| Q969Y2 | tRNA modification GTPase GTPBP3, mitochondrial OS=Homo sapiens GN=GTPBP3 PE=1 SV=2 - [GTPB3_HUMAN] |
| Q9NX74 | tRNA-dihydrouridine(20) synthase [NAD(P)+]-like OS=Homo sapiens GN=DUS2 PE=1 SV=1 - [DUS2L_HUMAN] |
| Q9BSV6 | tRNA-splicing endonuclease subunit Sen34 OS=Homo sapiens GN=TSEN34 PE=1 SV=1 - [SEN34_HUMAN] |
| Q9NZQ9 | Tropomodulin-4 OS=Homo sapiens GN=TMOD4 PE=2 SV=1 - [TMOD4_HUMAN] |
| Q15661 | Tryptase alpha/beta-1 OS=Homo sapiens GN=TPSAB1 PE=1 SV=1 - [TRYB1_HUMAN] |
| Q9UGM6 | Tryptophan--tRNA ligase, mitochondrial OS=Homo sapiens GN=WARS2 PE=1 SV=1 - [SYWM_HUMAN] |
| Q9Y3Q8 | TSC22 domain family protein 4 OS=Homo sapiens GN=TSC22D4 PE=1 SV=2 - [T22D4_HUMAN] |
| Q9NRJ4 | Tubby-related protein 4 OS=Homo sapiens GN=TULP4 PE=2 SV=2 - [TULP4_HUMAN] |
| Q9NY65 | Tubulin alpha-8 chain OS=Homo sapiens GN=TUBA8 PE=1 SV=1 - [TBA8_HUMAN] |
| P07437 | Tubulin beta chain OS=Homo sapiens GN=TUBB PE=1 SV=2 - [TBB5_HUMAN] |
| Q6ZTW0 | Tubulin polyglutamylase complex subunit 1 OS=Homo sapiens GN=TPGS1 PE=2 SV=2 - [TPGS1_HUMAN] |
| A6NNM8 | Tubulin polyglutamylase TTLL13P OS=Homo sapiens GN=TTLL13P PE=2 SV=2 - [TTL13_HUMAN] |
| Q6ZT98 | Tubulin polyglutamylase TTLL7 OS=Homo sapiens GN=TTLL7 PE=2 SV=2 - [TTLL7_HUMAN] |
| Q5VZ19 | Tudor domain-containing protein 10 OS=Homo sapiens GN=TDRD10 PE=2 SV=3 - [TDR10_HUMAN] |
| Q8NAT2 | Tudor domain-containing protein 5 OS=Homo sapiens GN=TDRD5 PE=1 SV=3 - [TDRD5_HUMAN] |
| Q8NHU6 | Tudor domain-containing protein 7 OS=Homo sapiens GN=TDRD7 PE=1 SV=2 - [TDRD7_HUMAN] |
| Q9UBB9 | Tuftelin-interacting protein 11 OS=Homo sapiens GN=TFIP11 PE=1 SV=1 - [TFP11_HUMAN] |
| Q5GJ75 | Tumor necrosis factor alpha-induced protein 8-like protein 3 OS=Homo sapiens GN=TNFAIP8L3 PE=1 SV=1 - [TP8L3_HUMAN] |
| Q969Z4 | Tumor necrosis factor receptor superfamily member 19L OS=Homo sapiens GN=RELT PE=1 SV=1 - [TR19L_HUMAN] |
| O75509 | Tumor necrosis factor receptor superfamily member 21 OS=Homo sapiens GN=TNFRSF21 PE=1 SV=1 - [TNR21_HUMAN] |
| Q16890 | Tumor protein D53 OS=Homo sapiens GN=TPD52L1 PE=1 SV=1 - [TPD53_HUMAN] |
| O43399 | Tumor protein D54 OS=Homo sapiens GN=TPD52L2 PE=1 SV=2 - [TPD54_HUMAN] |
| Q8NHX9 | Two pore calcium channel protein 2 OS=Homo sapiens GN=TPCN2 PE=1 SV=2 - [TPC2_HUMAN] |
| Q96PE3 | Type I inositol 3,4-bisphosphate 4-phosphatase OS=Homo sapiens GN=INPP4A PE=1 SV=1 - [INP4A_HUMAN] |
| O15327 | Type II inositol 3,4-bisphosphate 4-phosphatase OS=Homo sapiens GN=INPP4B PE=2 SV=4 - [INP4B_HUMAN] |
| P30556 | Type-1 angiotensin II receptor OS=Homo sapiens GN=AGTR1 PE=1 SV=1 - [AGTR1_HUMAN] |
| P50052 | Type-2 angiotensin II receptor OS=Homo sapiens GN=AGTR2 PE=1 SV=1 - [AGTR2_HUMAN] |
| P17735 | Tyrosine aminotransferase OS=Homo sapiens GN=TAT PE=1 SV=1 - [ATTY_HUMAN] |
| Q9UIG0 | Tyrosine-protein kinase BAZ1B OS=Homo sapiens GN=BAZ1B PE=1 SV=2 - [BAZ1B_HUMAN] |
| P16591 | Tyrosine-protein kinase Fer OS=Homo sapiens GN=FER PE=1 SV=2 - [FER_HUMAN] |
| P09769 | Tyrosine-protein kinase Fgr OS=Homo sapiens GN=FGR PE=1 SV=2 - [FGR_HUMAN] |
| O60674 | Tyrosine-protein kinase JAK2 OS=Homo sapiens GN=JAK2 PE=1 SV=2 - [JAK2_HUMAN] |
| P07948 | Tyrosine-protein kinase Lyn OS=Homo sapiens GN=LYN PE=1 SV=3 - [LYN_HUMAN] |
| P35590 | Tyrosine-protein kinase receptor Tie-1 OS=Homo sapiens GN=TIE1 PE=1 SV=1 - [TIE1_HUMAN] |
| Q06418 | Tyrosine-protein kinase receptor TYRO3 OS=Homo sapiens GN=TYRO3 PE=1 SV=1 - [TYRO3_HUMAN] |
| Q6J9G0 | Tyrosine-protein kinase STYK1 OS=Homo sapiens GN=STYK1 PE=1 SV=4 - [STYK1_HUMAN] |
| Q01973 | Tyrosine-protein kinase transmembrane receptor ROR1 OS=Homo sapiens GN=ROR1 PE=1 SV=2 - [ROR1_HUMAN] |
| P07947 | Tyrosine-protein kinase Yes OS=Homo sapiens GN=YES1 PE=1 SV=3 - [YES_HUMAN] |
| P18031 | Tyrosine-protein phosphatase non-receptor type 1 OS=Homo sapiens GN=PTPN1 PE=1 SV=1 - [PTN1_HUMAN] |
| P29074 | Tyrosine-protein phosphatase non-receptor type 4 OS=Homo sapiens GN=PTPN4 PE=1 SV=1 - [PTN4_HUMAN] |
| P43378 | Tyrosine-protein phosphatase non-receptor type 9 OS=Homo sapiens GN=PTPN9 PE=1 SV=1 - [PTN9_HUMAN] |
| P54577 | Tyrosine--tRNA ligase, cytoplasmic OS=Homo sapiens GN=YARS PE=1 SV=4 - [SYYC_HUMAN] |
| O15042 | U2 snRNP-associated SURP motif-containing protein OS=Homo sapiens GN=U2SURP PE=1 SV=2 - [SR140_HUMAN] |
| O00566 | U3 small nucleolar ribonucleoprotein protein MPP10 OS=Homo sapiens GN=MPHOSPH10 PE=1 SV=2 - [MPP10_HUMAN] |
| Q9BVJ6 | U3 small nucleolar RNA-associated protein 14 homolog A OS=Homo sapiens GN=UTP14A PE=1 SV=1 - [UT14A_HUMAN] |
| O43818 | U3 small nucleolar RNA-interacting protein 2 OS=Homo sapiens GN=RRP9 PE=1 SV=1 - [U3IP2_HUMAN] |
| O43290 | U4/U6.U5 tri-snRNP-associated protein 1 OS=Homo sapiens GN=SART1 PE=1 SV=1 - [SNUT1_HUMAN] |
| O75643 | U5 small nuclear ribonucleoprotein 200 kDa helicase OS=Homo sapiens GN=SNRNP200 PE=1 SV=2 - [U520_HUMAN] |
| P83369 | U7 snRNA-associated Sm-like protein LSm11 OS=Homo sapiens GN=LSM11 PE=1 SV=2 - [LSM11_HUMAN] |
| Q6ZU65 | Ubinuclein-2 OS=Homo sapiens GN=UBN2 PE=1 SV=2 - [UBN2_HUMAN] |
| Q9UMX0 | Ubiquilin-1 OS=Homo sapiens GN=UBQLN1 PE=1 SV=2 - [UBQL1_HUMAN] |
| Q9NRR5 | Ubiquilin-4 OS=Homo sapiens GN=UBQLN4 PE=1 SV=2 - [UBQL4_HUMAN] |
| A6NCW0 | Ubiquitin carboxyl-terminal hydrolase 17-like protein 3 OS=Homo sapiens GN=USP17L3 PE=3 SV=1 - [U17L3_HUMAN] |
| Q9Y2K6 | Ubiquitin carboxyl-terminal hydrolase 20 OS=Homo sapiens GN=USP20 PE=1 SV=2 - [UBP20_HUMAN] |
| Q9BXU7 | Ubiquitin carboxyl-terminal hydrolase 26 OS=Homo sapiens GN=USP26 PE=1 SV=1 - [UBP26_HUMAN] |
| Q70CQ4 | Ubiquitin carboxyl-terminal hydrolase 31 OS=Homo sapiens GN=USP31 PE=2 SV=2 - [UBP31_HUMAN] |
| Q9P2H5 | Ubiquitin carboxyl-terminal hydrolase 35 OS=Homo sapiens GN=USP35 PE=1 SV=3 - [UBP35_HUMAN] |
| Q9P275 | Ubiquitin carboxyl-terminal hydrolase 36 OS=Homo sapiens GN=USP36 PE=1 SV=3 - [UBP36_HUMAN] |
| Q86T82 | Ubiquitin carboxyl-terminal hydrolase 37 OS=Homo sapiens GN=USP37 PE=1 SV=2 - [UBP37_HUMAN] |
| Q70EL4 | Ubiquitin carboxyl-terminal hydrolase 43 OS=Homo sapiens GN=USP43 PE=1 SV=2 - [UBP43_HUMAN] |
| P35125 | Ubiquitin carboxyl-terminal hydrolase 6 OS=Homo sapiens GN=USP6 PE=1 SV=2 - [UBP6_HUMAN] |
| Q9HAC8 | Ubiquitin domain-containing protein 1 OS=Homo sapiens GN=UBTD1 PE=1 SV=1 - [UBTD1_HUMAN] |
| O14562 | Ubiquitin domain-containing protein UBFD1 OS=Homo sapiens GN=UBFD1 PE=1 SV=2 - [UBFD1_HUMAN] |
| Q96BN8 | Ubiquitin thioesterase otulin OS=Homo sapiens GN=OTULIN PE=1 SV=3 - [OTUL_HUMAN] |
| P57075 | Ubiquitin-associated and SH3 domain-containing protein A OS=Homo sapiens GN=UBASH3A PE=1 SV=1 - [UBS3A_HUMAN] |
| Q8TF42 | Ubiquitin-associated and SH3 domain-containing protein B OS=Homo sapiens GN=UBASH3B PE=1 SV=2 - [UBS3B_HUMAN] |
| Q9BSL1 | Ubiquitin-associated domain-containing protein 1 OS=Homo sapiens GN=UBAC1 PE=1 SV=1 - [UBAC1_HUMAN] |
| O00762 | Ubiquitin-conjugating enzyme E2 C OS=Homo sapiens GN=UBE2C PE=1 SV=1 - [UBE2C_HUMAN] |
| P22314 | Ubiquitin-like modifier-activating enzyme 1 OS=Homo sapiens GN=UBA1 PE=1 SV=3 - [UBA1_HUMAN] |
| P05161 | Ubiquitin-like protein ISG15 OS=Homo sapiens GN=ISG15 PE=1 SV=5 - [ISG15_HUMAN] |
| Q7Z3V4 | Ubiquitin-protein ligase E3B OS=Homo sapiens GN=UBE3B PE=1 SV=3 - [UBE3B_HUMAN] |
| P78381 | UDP-galactose translocator OS=Homo sapiens GN=SLC35A2 PE=1 SV=1 - [S35A2_HUMAN] |
| O75752 | UDP-GalNAc:beta-1,3-N-acetylgalactosaminyltransferase 1 OS=Homo sapiens GN=B3GALNT1 PE=2 SV=1 - [B3GL1_HUMAN] |
| O60701 | UDP-glucose 6-dehydrogenase OS=Homo sapiens GN=UGDH PE=1 SV=1 - [UGDH_HUMAN] |
| Q9NYU1 | UDP-glucose:glycoprotein glucosyltransferase 2 OS=Homo sapiens GN=UGGT2 PE=1 SV=4 - [UGGG2_HUMAN] |
| P54855 | UDP-glucuronosyltransferase 2B15 OS=Homo sapiens GN=UGT2B15 PE=1 SV=3 - [UDB15_HUMAN] |
| P16662 | UDP-glucuronosyltransferase 2B7 OS=Homo sapiens GN=UGT2B7 PE=1 SV=1 - [UD2B7_HUMAN] |
| O15294 | UDP-N-acetylglucosamine--peptide N-acetylglucosaminyltransferase 110 kDa subunit OS=Homo sapiens GN=OGT PE=1 SV=3 - [OGT1_HUMAN] |
| Q5EBM0 | UMP-CMP kinase 2, mitochondrial OS=Homo sapiens GN=CMPK2 PE=1 SV=3 - [CMPK2_HUMAN] |
| Q3MIX3 | Uncharacterized aarF domain-containing protein kinase 5 OS=Homo sapiens GN=ADCK5 PE=1 SV=2 - [ADCK5_HUMAN] |
| Q8N7S6 | Uncharacterized protein ARIH2OS OS=Homo sapiens GN=ARIH2OS PE=2 SV=1 - [ARI2O_HUMAN] |
| Q8N655 | Uncharacterized protein C10orf12 OS=Homo sapiens GN=C10orf12 PE=1 SV=1 - [CJ012_HUMAN] |
| Q9H943 | Uncharacterized protein C10orf68 OS=Homo sapiens GN=C10orf68 PE=2 SV=2 - [CJ068_HUMAN] |
| Q6ZUT1 | Uncharacterized protein C11orf57 OS=Homo sapiens GN=C11orf57 PE=1 SV=2 - [CK057_HUMAN] |
| C9JLR9 | Uncharacterized protein C11orf95 OS=Homo sapiens GN=C11orf95 PE=2 SV=1 - [CK095_HUMAN] |
| Q7Z7L8 | Uncharacterized protein C11orf96 OS=Homo sapiens GN=C11orf96 PE=1 SV=3 - [CK096_HUMAN] |
| Q96C57 | Uncharacterized protein C12orf43 OS=Homo sapiens GN=C12orf43 PE=1 SV=2 - [CL043_HUMAN] |
| Q86SX3 | Uncharacterized protein C14orf80 OS=Homo sapiens GN=C14orf80 PE=2 SV=2 - [CN080_HUMAN] |
| A8K5M9 | Uncharacterized protein C15orf62, mitochondrial OS=Homo sapiens GN=C15orf62 PE=2 SV=1 - [CO062_HUMAN] |
| Q96LL3 | Uncharacterized protein C16orf92 OS=Homo sapiens GN=C16orf92 PE=2 SV=1 - [CP092_HUMAN] |
| F2Z3M2 | Uncharacterized protein C17orf112 OS=Homo sapiens GN=C17orf112 PE=4 SV=1 - [CQ112_HUMAN] |
| Q5SNV9 | Uncharacterized protein C1orf167 OS=Homo sapiens GN=C1orf167 PE=2 SV=2 - [CA167_HUMAN] |
| Q5TEA3 | Uncharacterized protein C20orf194 OS=Homo sapiens GN=C20orf194 PE=1 SV=1 - [CT194_HUMAN] |
| Q08AI8 | Uncharacterized protein C2orf54 OS=Homo sapiens GN=C2orf54 PE=2 SV=2 - [CB054_HUMAN] |
| A6NGG8 | Uncharacterized protein C2orf71 OS=Homo sapiens GN=C2orf71 PE=1 SV=1 - [CB071_HUMAN] |
| A6NCS6 | Uncharacterized protein C2orf72 OS=Homo sapiens GN=C2orf72 PE=1 SV=2 - [CB072_HUMAN] |
| Q8ND61 | Uncharacterized protein C3orf20 OS=Homo sapiens GN=C3orf20 PE=2 SV=2 - [CC020_HUMAN] |
| Q96MH7 | Uncharacterized protein C5orf34 OS=Homo sapiens GN=C5orf34 PE=2 SV=2 - [CE034_HUMAN] |
| Q5T5N4 | Uncharacterized protein C6orf118 OS=Homo sapiens GN=C6orf118 PE=2 SV=1 - [CF118_HUMAN] |
| Q5T0Z8 | Uncharacterized protein C6orf132 OS=Homo sapiens GN=C6orf132 PE=1 SV=4 - [CF132_HUMAN] |
| Q5TEZ5 | Uncharacterized protein C6orf163 OS=Homo sapiens GN=C6orf163 PE=4 SV=2 - [CF163_HUMAN] |
| P0C671 | Uncharacterized protein C6orf222 OS=Homo sapiens GN=C6orf222 PE=1 SV=1 - [CF222_HUMAN] |
| Q6ZTR5 | Uncharacterized protein CXorf22 OS=Homo sapiens GN=CXorf22 PE=2 SV=3 - [CX022_HUMAN] |
| Q9UF83 | Uncharacterized protein DKFZp434B061 OS=Homo sapiens PE=2 SV=2 - [YM012_HUMAN] |
| Q8N2X6 | Uncharacterized protein EXOC3-AS1 OS=Homo sapiens GN=EXOC3-AS1 PE=1 SV=1 - [EXAS1_HUMAN] |
| O15063 | Uncharacterized protein KIAA0355 OS=Homo sapiens GN=KIAA0355 PE=1 SV=2 - [K0355_HUMAN] |
| Q68EN5 | Uncharacterized protein KIAA0895-like OS=Homo sapiens GN=KIAA0895L PE=2 SV=1 - [K895L_HUMAN] |
| Q2LD37 | Uncharacterized protein KIAA1109 OS=Homo sapiens GN=KIAA1109 PE=1 SV=2 - [K1109_HUMAN] |
| Q9ULL0 | Uncharacterized protein KIAA1210 OS=Homo sapiens GN=KIAA1210 PE=2 SV=3 - [K1210_HUMAN] |
| Q6NV74 | Uncharacterized protein KIAA1211-like OS=Homo sapiens GN=KIAA1211L PE=2 SV=3 - [K121L_HUMAN] |
| Q9HCM1 | Uncharacterized protein KIAA1551 OS=Homo sapiens GN=KIAA1551 PE=1 SV=3 - [K1551_HUMAN] |
| Q0VF49 | Uncharacterized protein KIAA2012 OS=Homo sapiens GN=KIAA2012 PE=2 SV=2 - [K2012_HUMAN] |
| Q5HYC2 | Uncharacterized protein KIAA2026 OS=Homo sapiens GN=KIAA2026 PE=2 SV=2 - [K2026_HUMAN] |
| Q96FF7 | Uncharacterized protein LOC113230 OS=Homo sapiens PE=2 SV=4 - [YS003_HUMAN] |
| Q8NCS4 | Uncharacterized protein ZMYM6NB OS=Homo sapiens GN=ZMYM6NB PE=2 SV=1 - [ZMYNB_HUMAN] |
| Q9UBC5 | Unconventional myosin-Ia OS=Homo sapiens GN=MYO1A PE=1 SV=1 - [MYO1A_HUMAN] |
| O43795 | Unconventional myosin-Ib OS=Homo sapiens GN=MYO1B PE=1 SV=3 - [MYO1B_HUMAN] |
| O94832 | Unconventional myosin-Id OS=Homo sapiens GN=MYO1D PE=1 SV=2 - [MYO1D_HUMAN] |
| O00160 | Unconventional myosin-If OS=Homo sapiens GN=MYO1F PE=1 SV=3 - [MYO1F_HUMAN] |
| B2RTY4 | Unconventional myosin-IXa OS=Homo sapiens GN=MYO9A PE=1 SV=2 - [MYO9A_HUMAN] |
| Q13459 | Unconventional myosin-IXb OS=Homo sapiens GN=MYO9B PE=1 SV=3 - [MYO9B_HUMAN] |
| Q9Y4I1 | Unconventional myosin-Va OS=Homo sapiens GN=MYO5A PE=1 SV=2 - [MYO5A_HUMAN] |
| Q9UM54 | Unconventional myosin-VI OS=Homo sapiens GN=MYO6 PE=1 SV=4 - [MYO6_HUMAN] |
| Q6PIF6 | Unconventional myosin-VIIb OS=Homo sapiens GN=MYO7B PE=1 SV=2 - [MYO7B_HUMAN] |
| Q9UKN7 | Unconventional myosin-XV OS=Homo sapiens GN=MYO15A PE=1 SV=2 - [MYO15_HUMAN] |
| Q96JP2 | Unconventional myosin-XVB OS=Homo sapiens GN=MYO15B PE=1 SV=2 - [MY15B_HUMAN] |
| Q8IUG5 | Unconventional myosin-XVIIIb OS=Homo sapiens GN=MYO18B PE=1 SV=1 - [MY18B_HUMAN] |
| Q9BZE7 | UPF0193 protein EVG1 OS=Homo sapiens GN=C22orf23 PE=1 SV=1 - [EVG1_HUMAN] |
| Q9BPX7 | UPF0415 protein C7orf25 OS=Homo sapiens GN=C7orf25 PE=1 SV=1 - [CG025_HUMAN] |
| Q49AR2 | UPF0489 protein C5orf22 OS=Homo sapiens GN=C5orf22 PE=1 SV=2 - [CE022_HUMAN] |
| Q8N8R5 | UPF0565 protein C2orf69 OS=Homo sapiens GN=C2orf69 PE=1 SV=1 - [CB069_HUMAN] |
| A8MWY0 | UPF0577 protein KIAA1324-like OS=Homo sapiens GN=KIAA1324L PE=2 SV=2 - [K132L_HUMAN] |
| Q92738 | USP6 N-terminal-like protein OS=Homo sapiens GN=USP6NL PE=1 SV=3 - [US6NL_HUMAN] |
| Q16851 | UTP--glucose-1-phosphate uridylyltransferase OS=Homo sapiens GN=UGP2 PE=1 SV=5 - [UGPA_HUMAN] |
| P46939 | Utrophin OS=Homo sapiens GN=UTRN PE=1 SV=2 - [UTRO_HUMAN] |
| Q9BZF9 | Uveal autoantigen with coiled-coil domains and ankyrin repeats OS=Homo sapiens GN=UACA PE=1 SV=2 - [UACA_HUMAN] |
| P15918 | V(D)J recombination-activating protein 1 OS=Homo sapiens GN=RAG1 PE=1 SV=2 - [RAG1_HUMAN] |
| Q7Z7G8 | Vacuolar protein sorting-associated protein 13B OS=Homo sapiens GN=VPS13B PE=1 SV=2 - [VP13B_HUMAN] |
| Q709C8 | Vacuolar protein sorting-associated protein 13C OS=Homo sapiens GN=VPS13C PE=1 SV=1 - [VP13C_HUMAN] |
| Q9H269 | Vacuolar protein sorting-associated protein 16 homolog OS=Homo sapiens GN=VPS16 PE=1 SV=2 - [VPS16_HUMAN] |
| Q9NRW7 | Vacuolar protein sorting-associated protein 45 OS=Homo sapiens GN=VPS45 PE=1 SV=1 - [VPS45_HUMAN] |
| Q15906 | Vacuolar protein sorting-associated protein 72 homolog OS=Homo sapiens GN=VPS72 PE=1 SV=1 - [VPS72_HUMAN] |
| Q8N3P4 | Vacuolar protein sorting-associated protein 8 homolog OS=Homo sapiens GN=VPS8 PE=1 SV=3 - [VPS8_HUMAN] |
| Q86WA6 | Valacyclovir hydrolase OS=Homo sapiens GN=BPHL PE=1 SV=1 - [BPHL_HUMAN] |
| Q9ULK5 | Vang-like protein 2 OS=Homo sapiens GN=VANGL2 PE=1 SV=2 - [VANG2_HUMAN] |
| Q6UXB2 | VEGF coregulated chemokine 1 OS=Homo sapiens GN=CXCL17 PE=1 SV=1 - [VCC1_HUMAN] |
| Q9H8Y1 | Vertnin OS=Homo sapiens GN=VRTN PE=1 SV=1 - [VRTN_HUMAN] |
| Q5VWC8 | Very-long-chain (3R)-3-hydroxyacyl-CoA dehydratase 4 OS=Homo sapiens GN=HACD4 PE=1 SV=1 - [HACD4_HUMAN] |
| Q9NZ43 | Vesicle transport protein USE1 OS=Homo sapiens GN=USE1 PE=1 SV=2 - [USE1_HUMAN] |
| P63027 | Vesicle-associated membrane protein 2 OS=Homo sapiens GN=VAMP2 PE=1 SV=3 - [VAMP2_HUMAN] |
| P46459 | Vesicle-fusing ATPase OS=Homo sapiens GN=NSF PE=1 SV=3 - [NSF_HUMAN] |
| Q9BRL7 | Vesicle-trafficking protein SEC22c OS=Homo sapiens GN=SEC22C PE=1 SV=1 - [SC22C_HUMAN] |
| Q8NDX2 | Vesicular glutamate transporter 3 OS=Homo sapiens GN=SLC17A8 PE=1 SV=1 - [VGLU3_HUMAN] |
| Q9HBM0 | Vezatin OS=Homo sapiens GN=VEZT PE=1 SV=3 - [VEZA_HUMAN] |
| P09327 | Villin-1 OS=Homo sapiens GN=VIL1 PE=1 SV=4 - [VILI_HUMAN] |
| P08670 | Vimentin OS=Homo sapiens GN=VIM PE=1 SV=4 - [VIME_HUMAN] |
| O60504 | Vinexin OS=Homo sapiens GN=SORBS3 PE=1 SV=2 - [VINEX_HUMAN] |
| Q9NZR4 | Visual system homeobox 1 OS=Homo sapiens GN=VSX1 PE=1 SV=2 - [VSX1_HUMAN] |
| Q6UXI7 | Vitrin OS=Homo sapiens GN=VIT PE=2 SV=1 - [VITRN_HUMAN] |
| P21796 | Voltage-dependent anion-selective channel protein 1 OS=Homo sapiens GN=VDAC1 PE=1 SV=2 - [VDAC1_HUMAN] |
| Q13936 | Voltage-dependent L-type calcium channel subunit alpha-1C OS=Homo sapiens GN=CACNA1C PE=1 SV=4 - [CAC1C_HUMAN] |
| Q02641 | Voltage-dependent L-type calcium channel subunit beta-1 OS=Homo sapiens GN=CACNB1 PE=2 SV=3 - [CACB1_HUMAN] |
| Q08289 | Voltage-dependent L-type calcium channel subunit beta-2 OS=Homo sapiens GN=CACNB2 PE=1 SV=3 - [CACB2_HUMAN] |
| O43497 | Voltage-dependent T-type calcium channel subunit alpha-1G OS=Homo sapiens GN=CACNA1G PE=2 SV=3 - [CAC1G_HUMAN] |
| O95180 | Voltage-dependent T-type calcium channel subunit alpha-1H OS=Homo sapiens GN=CACNA1H PE=1 SV=4 - [CAC1H_HUMAN] |
| Q9P0X4 | Voltage-dependent T-type calcium channel subunit alpha-1I OS=Homo sapiens GN=CACNA1I PE=1 SV=1 - [CAC1I_HUMAN] |
| Q8IWT6 | Volume-regulated anion channel subunit LRRC8A OS=Homo sapiens GN=LRRC8A PE=1 SV=1 - [LRC8A_HUMAN] |
| Q9BXE9 | Vomeronasal type-1 receptor 3 OS=Homo sapiens GN=VN1R3 PE=2 SV=1 - [VN1R3_HUMAN] |
| Q5TIE3 | von Willebrand factor A domain-containing protein 5B1 OS=Homo sapiens GN=VWA5B1 PE=1 SV=2 - [VW5B1_HUMAN] |
| A3KMH1 | von Willebrand factor A domain-containing protein 8 OS=Homo sapiens GN=VWA8 PE=1 SV=2 - [VWA8_HUMAN] |
| Q8WY21 | VPS10 domain-containing receptor SorCS1 OS=Homo sapiens GN=SORCS1 PE=1 SV=3 - [SORC1_HUMAN] |
| Q86VR7 | V-set and immunoglobulin domain-containing protein 10-like OS=Homo sapiens GN=VSIG10L PE=2 SV=2 - [VS10L_HUMAN] |
| Q5VU13 | V-set and immunoglobulin domain-containing protein 8 OS=Homo sapiens GN=VSIG8 PE=2 SV=1 - [VSIG8_HUMAN] |
| P15313 | V-type proton ATPase subunit B, kidney isoform OS=Homo sapiens GN=ATP6V1B1 PE=1 SV=3 - [VATB1_HUMAN] |
| P21283 | V-type proton ATPase subunit C 1 OS=Homo sapiens GN=ATP6V1C1 PE=1 SV=4 - [VATC1_HUMAN] |
| Q8N8Y2 | V-type proton ATPase subunit d 2 OS=Homo sapiens GN=ATP6V0D2 PE=2 SV=1 - [VA0D2_HUMAN] |
| Q15904 | V-type proton ATPase subunit S1 OS=Homo sapiens GN=ATP6AP1 PE=1 SV=2 - [VAS1_HUMAN] |
| Q8TF74 | WAS/WASL-interacting protein family member 2 OS=Homo sapiens GN=WIPF2 PE=1 SV=1 - [WIPF2_HUMAN] |
| Q2M389 | WASH complex subunit 7 OS=Homo sapiens GN=KIAA1033 PE=1 SV=2 - [WASH7_HUMAN] |
| Q8IWB7 | WD repeat and FYVE domain-containing protein 1 OS=Homo sapiens GN=WDFY1 PE=1 SV=1 - [WDFY1_HUMAN] |
| Q8IZQ1 | WD repeat and FYVE domain-containing protein 3 OS=Homo sapiens GN=WDFY3 PE=1 SV=2 - [WDFY3_HUMAN] |
| Q6ZS81 | WD repeat- and FYVE domain-containing protein 4 OS=Homo sapiens GN=WDFY4 PE=1 SV=3 - [WDFY4_HUMAN] |
| O75717 | WD repeat and HMG-box DNA-binding protein 1 OS=Homo sapiens GN=WDHD1 PE=1 SV=1 - [WDHD1_HUMAN] |
| Q8IZU2 | WD repeat-containing protein 17 OS=Homo sapiens GN=WDR17 PE=2 SV=2 - [WDR17_HUMAN] |
| Q9UNX4 | WD repeat-containing protein 3 OS=Homo sapiens GN=WDR3 PE=1 SV=1 - [WDR3_HUMAN] |
| Q8IWG1 | WD repeat-containing protein 63 OS=Homo sapiens GN=WDR63 PE=2 SV=1 - [WDR63_HUMAN] |
| B1ANS9 | WD repeat-containing protein 64 OS=Homo sapiens GN=WDR64 PE=2 SV=1 - [WDR64_HUMAN] |
| Q3MJ13 | WD repeat-containing protein 72 OS=Homo sapiens GN=WDR72 PE=2 SV=2 - [WDR72_HUMAN] |
| Q5VTH9 | WD repeat-containing protein 78 OS=Homo sapiens GN=WDR78 PE=2 SV=1 - [WDR78_HUMAN] |
| Q6ZQQ6 | WD repeat-containing protein 87 OS=Homo sapiens GN=WDR87 PE=1 SV=3 - [WDR87_HUMAN] |
| A4D1P6 | WD repeat-containing protein 91 OS=Homo sapiens GN=WDR91 PE=1 SV=2 - [WDR91_HUMAN] |
| Q9NXC5 | WD repeat-containing protein mio OS=Homo sapiens GN=MIOS PE=1 SV=2 - [MIO_HUMAN] |
| P0C1S8 | Wee1-like protein kinase 2 OS=Homo sapiens GN=WEE2 PE=2 SV=2 - [WEE2_HUMAN] |
| P30291 | Wee1-like protein kinase OS=Homo sapiens GN=WEE1 PE=1 SV=2 - [WEE1_HUMAN] |
| Q14191 | Werner syndrome ATP-dependent helicase OS=Homo sapiens GN=WRN PE=1 SV=2 - [WRN_HUMAN] |
| A6NIX2 | Wilms tumor protein 1-interacting protein OS=Homo sapiens GN=WTIP PE=1 SV=3 - [WTIP_HUMAN] |
| O95389 | WNT1-inducible-signaling pathway protein 3 OS=Homo sapiens GN=WISP3 PE=1 SV=1 - [WISP3_HUMAN] |
| O43895 | Xaa-Pro aminopeptidase 2 OS=Homo sapiens GN=XPNPEP2 PE=2 SV=3 - [XPP2_HUMAN] |
| Q702N8 | Xin actin-binding repeat-containing protein 1 OS=Homo sapiens GN=XIRP1 PE=1 SV=1 - [XIRP1_HUMAN] |
| A4UGR9 | Xin actin-binding repeat-containing protein 2 OS=Homo sapiens GN=XIRP2 PE=1 SV=2 - [XIRP2_HUMAN] |
| Q5GH72 | XK-related protein 7 OS=Homo sapiens GN=XKR7 PE=2 SV=1 - [XKR7_HUMAN] |
| Q9H6D3 | XK-related protein 8 OS=Homo sapiens GN=XKR8 PE=1 SV=1 - [XKR8_HUMAN] |
| Q6P2D8 | X-ray radiation resistance-associated protein 1 OS=Homo sapiens GN=XRRA1 PE=2 SV=2 - [XRRA1_HUMAN] |
| Q9H1B5 | Xylosyltransferase 2 OS=Homo sapiens GN=XYLT2 PE=2 SV=2 - [XYLT2_HUMAN] |
| Q9Y5A9 | YTH domain-containing family protein 2 OS=Homo sapiens GN=YTHDF2 PE=1 SV=2 - [YTHD2_HUMAN] |
| Q96MU7 | YTH domain-containing protein 1 OS=Homo sapiens GN=YTHDC1 PE=1 SV=3 - [YTDC1_HUMAN] |
| Q8IY57 | YY1-associated factor 2 OS=Homo sapiens GN=YAF2 PE=1 SV=3 - [YAF2_HUMAN] |
| Q9H869 | YY1-associated protein 1 OS=Homo sapiens GN=YY1AP1 PE=1 SV=2 - [YYAP1_HUMAN] |
| Q99592 | Zinc finger and BTB domain-containing protein 18 OS=Homo sapiens GN=ZBTB18 PE=1 SV=1 - [ZBT18_HUMAN] |
| Q9ULJ3 | Zinc finger and BTB domain-containing protein 21 OS=Homo sapiens GN=ZBTB21 PE=1 SV=2 - [ZBT21_HUMAN] |
| Q8NCN2 | Zinc finger and BTB domain-containing protein 34 OS=Homo sapiens GN=ZBTB34 PE=2 SV=4 - [ZBT34_HUMAN] |
| Q5SVQ8 | Zinc finger and BTB domain-containing protein 41 OS=Homo sapiens GN=ZBTB41 PE=1 SV=1 - [ZBT41_HUMAN] |
| Q15916 | Zinc finger and BTB domain-containing protein 6 OS=Homo sapiens GN=ZBTB6 PE=1 SV=1 - [ZBTB6_HUMAN] |
| O15156 | Zinc finger and BTB domain-containing protein 7B OS=Homo sapiens GN=ZBTB7B PE=1 SV=2 - [ZBT7B_HUMAN] |
| Q96SZ4 | Zinc finger and SCAN domain-containing protein 10 OS=Homo sapiens GN=ZSCAN10 PE=1 SV=1 - [ZSC10_HUMAN] |
| Q96IU2 | Zinc finger BED domain-containing protein 3 OS=Homo sapiens GN=ZBED3 PE=1 SV=1 - [ZBED3_HUMAN] |
| O75132 | Zinc finger BED domain-containing protein 4 OS=Homo sapiens GN=ZBED4 PE=1 SV=2 - [ZBED4_HUMAN] |
| Q49AG3 | Zinc finger BED domain-containing protein 5 OS=Homo sapiens GN=ZBED5 PE=2 SV=2 - [ZBED5_HUMAN] |
| P86452 | Zinc finger BED domain-containing protein 6 OS=Homo sapiens GN=ZBED6 PE=3 SV=1 - [ZBED6_HUMAN] |
| O75152 | Zinc finger CCCH domain-containing protein 11A OS=Homo sapiens GN=ZC3H11A PE=1 SV=3 - [ZC11A_HUMAN] |
| Q5T200 | Zinc finger CCCH domain-containing protein 13 OS=Homo sapiens GN=ZC3H13 PE=1 SV=1 - [ZC3HD_HUMAN] |
| Q86VM9 | Zinc finger CCCH domain-containing protein 18 OS=Homo sapiens GN=ZC3H18 PE=1 SV=2 - [ZCH18_HUMAN] |
| Q9C0B9 | Zinc finger CCHC domain-containing protein 2 OS=Homo sapiens GN=ZCCHC2 PE=1 SV=6 - [ZCHC2_HUMAN] |
| Q9NUD5 | Zinc finger CCHC domain-containing protein 3 OS=Homo sapiens GN=ZCCHC3 PE=1 SV=1 - [ZCHC3_HUMAN] |
| Q9H5U6 | Zinc finger CCHC domain-containing protein 4 OS=Homo sapiens GN=ZCCHC4 PE=1 SV=3 - [ZCHC4_HUMAN] |
| P37275 | Zinc finger E-box-binding homeobox 1 OS=Homo sapiens GN=ZEB1 PE=1 SV=2 - [ZEB1_HUMAN] |
| O60315 | Zinc finger E-box-binding homeobox 2 OS=Homo sapiens GN=ZEB2 PE=1 SV=1 - [ZEB2_HUMAN] |
| Q68DK2 | Zinc finger FYVE domain-containing protein 26 OS=Homo sapiens GN=ZFYVE26 PE=1 SV=3 - [ZFY26_HUMAN] |
| O95405 | Zinc finger FYVE domain-containing protein 9 OS=Homo sapiens GN=ZFYVE9 PE=1 SV=2 - [ZFYV9_HUMAN] |
| Q9C0A1 | Zinc finger homeobox protein 2 OS=Homo sapiens GN=ZFHX2 PE=2 SV=3 - [ZFHX2_HUMAN] |
| Q86UP3 | Zinc finger homeobox protein 4 OS=Homo sapiens GN=ZFHX4 PE=1 SV=1 - [ZFHX4_HUMAN] |
| O95789 | Zinc finger MYM-type protein 6 OS=Homo sapiens GN=ZMYM6 PE=2 SV=2 - [ZMYM6_HUMAN] |
| Q8IZC7 | Zinc finger protein 101 OS=Homo sapiens GN=ZNF101 PE=1 SV=1 - [ZN101_HUMAN] |
| Q9H2Y7 | Zinc finger protein 106 OS=Homo sapiens GN=ZNF106 PE=1 SV=1 - [ZN106_HUMAN] |
| P52737 | Zinc finger protein 136 OS=Homo sapiens GN=ZNF136 PE=1 SV=1 - [ZN136_HUMAN] |
| P52746 | Zinc finger protein 142 OS=Homo sapiens GN=ZNF142 PE=2 SV=4 - [ZN142_HUMAN] |
| Q13106 | Zinc finger protein 154 OS=Homo sapiens GN=ZNF154 PE=2 SV=3 - [ZN154_HUMAN] |
| P17020 | Zinc finger protein 16 OS=Homo sapiens GN=ZNF16 PE=1 SV=3 - [ZNF16_HUMAN] |
| P98182 | Zinc finger protein 200 OS=Homo sapiens GN=ZNF200 PE=2 SV=2 - [ZN200_HUMAN] |
| O43345 | Zinc finger protein 208 OS=Homo sapiens GN=ZNF208 PE=2 SV=2 - [ZN208_HUMAN] |
| Q9UDV6 | Zinc finger protein 212 OS=Homo sapiens GN=ZNF212 PE=1 SV=3 - [ZN212_HUMAN] |
| Q9UNY5 | Zinc finger protein 232 OS=Homo sapiens GN=ZNF232 PE=1 SV=1 - [ZN232_HUMAN] |
| P17035 | Zinc finger protein 28 OS=Homo sapiens GN=ZNF28 PE=2 SV=5 - [ZNF28_HUMAN] |
| Q9HBT8 | Zinc finger protein 286A OS=Homo sapiens GN=ZNF286A PE=1 SV=1 - [Z286A_HUMAN] |
| Q96JL9 | Zinc finger protein 333 OS=Homo sapiens GN=ZNF333 PE=2 SV=3 - [ZN333_HUMAN] |
| Q9Y3M9 | Zinc finger protein 337 OS=Homo sapiens GN=ZNF337 PE=1 SV=2 - [ZN337_HUMAN] |
| Q06730 | Zinc finger protein 33A OS=Homo sapiens GN=ZNF33A PE=1 SV=3 - [ZN33A_HUMAN] |
| Q9BYN7 | Zinc finger protein 341 OS=Homo sapiens GN=ZNF341 PE=1 SV=2 - [ZN341_HUMAN] |
| Q96SE7 | Zinc finger protein 347 OS=Homo sapiens GN=ZNF347 PE=1 SV=2 - [ZN347_HUMAN] |
| Q9Y6Q3 | Zinc finger protein 37 homolog OS=Homo sapiens GN=ZFP37 PE=2 SV=3 - [ZFP37_HUMAN] |
| P17032 | Zinc finger protein 37A OS=Homo sapiens GN=ZNF37A PE=2 SV=3 - [ZN37A_HUMAN] |
| Q96PM9 | Zinc finger protein 385A OS=Homo sapiens GN=ZNF385A PE=1 SV=2 - [Z385A_HUMAN] |
| Q53GI3 | Zinc finger protein 394 OS=Homo sapiens GN=ZNF394 PE=1 SV=2 - [ZN394_HUMAN] |
| P15822 | Zinc finger protein 40 OS=Homo sapiens GN=HIVEP1 PE=1 SV=3 - [ZEP1_HUMAN] |
| Q8TAU3 | Zinc finger protein 417 OS=Homo sapiens GN=ZNF417 PE=1 SV=2 - [ZN417_HUMAN] |
| Q96MM3 | Zinc finger protein 42 homolog OS=Homo sapiens GN=ZFP42 PE=1 SV=2 - [ZFP42_HUMAN] |
| Q9BUY5 | Zinc finger protein 426 OS=Homo sapiens GN=ZNF426 PE=1 SV=1 - [ZN426_HUMAN] |
| Q9H8G1 | Zinc finger protein 430 OS=Homo sapiens GN=ZNF430 PE=1 SV=3 - [ZN430_HUMAN] |
| Q7Z4V0 | Zinc finger protein 438 OS=Homo sapiens GN=ZNF438 PE=2 SV=1 - [ZN438_HUMAN] |
| Q9Y4E5 | Zinc finger protein 451 OS=Homo sapiens GN=ZNF451 PE=1 SV=2 - [ZN451_HUMAN] |
| Q8TF39 | Zinc finger protein 483 OS=Homo sapiens GN=ZNF483 PE=1 SV=3 - [ZN483_HUMAN] |
| Q9P255 | Zinc finger protein 492 OS=Homo sapiens GN=ZNF492 PE=2 SV=2 - [ZN492_HUMAN] |
| Q96IT1 | Zinc finger protein 496 OS=Homo sapiens GN=ZNF496 PE=1 SV=1 - [ZN496_HUMAN] |
| Q96KM6 | Zinc finger protein 512B OS=Homo sapiens GN=ZNF512B PE=1 SV=1 - [Z512B_HUMAN] |
| Q6AHZ1 | Zinc finger protein 518A OS=Homo sapiens GN=ZNF518A PE=2 SV=2 - [Z518A_HUMAN] |
| Q8NB42 | Zinc finger protein 527 OS=Homo sapiens GN=ZNF527 PE=2 SV=2 - [ZN527_HUMAN] |
| O15090 | Zinc finger protein 536 OS=Homo sapiens GN=ZNF536 PE=1 SV=3 - [ZN536_HUMAN] |
| Q9H0D2 | Zinc finger protein 541 OS=Homo sapiens GN=ZNF541 PE=2 SV=3 - [ZN541_HUMAN] |
| Q8N184 | Zinc finger protein 567 OS=Homo sapiens GN=ZNF567 PE=1 SV=3 - [ZN567_HUMAN] |
| Q96N58 | Zinc finger protein 578 OS=Homo sapiens GN=ZNF578 PE=2 SV=2 - [ZN578_HUMAN] |
| Q92610 | Zinc finger protein 592 OS=Homo sapiens GN=ZNF592 PE=1 SV=2 - [ZN592_HUMAN] |
| O15014 | Zinc finger protein 609 OS=Homo sapiens GN=ZNF609 PE=1 SV=2 - [ZN609_HUMAN] |
| Q6AZW8 | Zinc finger protein 660 OS=Homo sapiens GN=ZNF660 PE=1 SV=1 - [ZN660_HUMAN] |
| Q96CS4 | Zinc finger protein 689 OS=Homo sapiens GN=ZNF689 PE=2 SV=1 - [ZN689_HUMAN] |
| Q9H0M5 | Zinc finger protein 700 OS=Homo sapiens GN=ZNF700 PE=2 SV=1 - [ZN700_HUMAN] |
| Q6ZNC4 | Zinc finger protein 704 OS=Homo sapiens GN=ZNF704 PE=1 SV=1 - [ZN704_HUMAN] |
| A8MVS1 | Zinc finger protein 705F OS=Homo sapiens GN=ZNF705F PE=3 SV=1 - [Z705F_HUMAN] |
| P0DKX0 | Zinc finger protein 728 OS=Homo sapiens GN=ZNF728 PE=3 SV=1 - [ZN728_HUMAN] |
| Q96N20 | Zinc finger protein 75A OS=Homo sapiens GN=ZNF75A PE=2 SV=1 - [ZN75A_HUMAN] |
| Q6ZMW2 | Zinc finger protein 782 OS=Homo sapiens GN=ZNF782 PE=2 SV=1 - [ZN782_HUMAN] |
| Q3KP31 | Zinc finger protein 791 OS=Homo sapiens GN=ZNF791 PE=2 SV=1 - [ZN791_HUMAN] |
| Q7Z570 | Zinc finger protein 804A OS=Homo sapiens GN=ZNF804A PE=1 SV=3 - [Z804A_HUMAN] |
| P0C7X5 | Zinc finger protein 806 OS=Homo sapiens GN=ZNF806 PE=3 SV=1 - [ZN806_HUMAN] |
| Q0VGE8 | Zinc finger protein 816 OS=Homo sapiens GN=ZNF816 PE=2 SV=2 - [ZN816_HUMAN] |
| Q8N141 | Zinc finger protein 82 homolog OS=Homo sapiens GN=ZFP82 PE=2 SV=1 - [ZFP82_HUMAN] |
| O75541 | Zinc finger protein 821 OS=Homo sapiens GN=ZNF821 PE=1 SV=3 - [ZN821_HUMAN] |
| Q5JPB2 | Zinc finger protein 831 OS=Homo sapiens GN=ZNF831 PE=2 SV=4 - [ZN831_HUMAN] |
| A6NHJ4 | Zinc finger protein 860 OS=Homo sapiens GN=ZNF860 PE=2 SV=3 - [ZN860_HUMAN] |
| O60290 | Zinc finger protein 862 OS=Homo sapiens GN=ZNF862 PE=2 SV=2 - [ZN862_HUMAN] |
| A8MXY4 | Zinc finger protein 99 OS=Homo sapiens GN=ZNF99 PE=2 SV=3 - [ZNF99_HUMAN] |
| Q6ZN18 | Zinc finger protein AEBP2 OS=Homo sapiens GN=AEBP2 PE=1 SV=2 - [AEBP2_HUMAN] |
| Q92782 | Zinc finger protein neuro-d4 OS=Homo sapiens GN=DPF1 PE=2 SV=2 - [DPF1_HUMAN] |
| Q63HK3 | Zinc finger protein with KRAB and SCAN domains 2 OS=Homo sapiens GN=ZKSCAN2 PE=1 SV=2 - [ZKSC2_HUMAN] |
| O95218 | Zinc finger Ran-binding domain-containing protein 2 OS=Homo sapiens GN=ZRANB2 PE=1 SV=2 - [ZRAB2_HUMAN] |
| O43149 | Zinc finger ZZ-type and EF-hand domain-containing protein 1 OS=Homo sapiens GN=ZZEF1 PE=1 SV=6 - [ZZEF1_HUMAN] |
| Q9Y6X8 | Zinc fingers and homeoboxes protein 2 OS=Homo sapiens GN=ZHX2 PE=1 SV=1 - [ZHX2_HUMAN] |
| Q9BQ52 | Zinc phosphodiesterase ELAC protein 2 OS=Homo sapiens GN=ELAC2 PE=1 SV=2 - [RNZ2_HUMAN] |
| Q9BRY0 | Zinc transporter ZIP3 OS=Homo sapiens GN=SLC39A3 PE=1 SV=2 - [S39A3_HUMAN] |
| Q9NUM3 | Zinc transporter ZIP9 OS=Homo sapiens GN=SLC39A9 PE=2 SV=2 - [S39A9_HUMAN] |
| Q401N2 | Zinc-activated ligand-gated ion channel OS=Homo sapiens GN=ZACN PE=1 SV=2 - [ZACN_HUMAN] |
| Q15942 | Zyxin OS=Homo sapiens GN=ZYX PE=1 SV=1 - [ZYX_HUMAN] |

**Table 2S.** Identified membrane glycoproteins from Tn-negative, blood group A negative, STn-positive MIBC, with O-HexNAc as posttranslational modifications after neuraminiase treatment.

| Accession | Description |
| --- | --- |
| Q04917 | 14-3-3 protein eta OS=Homo sapiens GN=YWHAH PE=1 SV=4 - [1433F_HUMAN] |
| P51178 | 1-phosphatidylinositol 4,5-bisphosphate phosphodiesterase delta-1 OS=Homo sapiens GN=PLCD1 PE=1 SV=2 - [PLCD1_HUMAN] |
| Q8N3E9 | 1-phosphatidylinositol 4,5-bisphosphate phosphodiesterase delta-3 OS=Homo sapiens GN=PLCD3 PE=1 SV=3 - [PLCD3_HUMAN] |
| Q9P212 | 1-phosphatidylinositol 4,5-bisphosphate phosphodiesterase epsilon-1 OS=Homo sapiens GN=PLCE1 PE=1 SV=3 - [PLCE1_HUMAN] |
| Q4KWH8 | 1-phosphatidylinositol 4,5-bisphosphate phosphodiesterase eta-1 OS=Homo sapiens GN=PLCH1 PE=1 SV=1 - [PLCH1_HUMAN] |
| O75038 | 1-phosphatidylinositol 4,5-bisphosphate phosphodiesterase eta-2 OS=Homo sapiens GN=PLCH2 PE=2 SV=3 - [PLCH2_HUMAN] |
| P19174 | 1-phosphatidylinositol 4,5-bisphosphate phosphodiesterase gamma-1 OS=Homo sapiens GN=PLCG1 PE=1 SV=1 - [PLCG1_HUMAN] |
| P16885 | 1-phosphatidylinositol 4,5-bisphosphate phosphodiesterase gamma-2 OS=Homo sapiens GN=PLCG2 PE=1 SV=4 - [PLCG2_HUMAN] |
| P34969 | 5-hydroxytryptamine receptor 7 OS=Homo sapiens GN=HTR7 PE=1 SV=2 - [5HT7R_HUMAN] |
| Q96FT7 | Acid-sensing ion channel 4 OS=Homo sapiens GN=ASIC4 PE=1 SV=2 - [ASIC4_HUMAN] |
| Q8NER5 | Activin receptor type-1C OS=Homo sapiens GN=ACVR1C PE=1 SV=1 - [ACV1C_HUMAN] |
| O95996 | Adenomatous polyposis coli protein 2 OS=Homo sapiens GN=APC2 PE=1 SV=1 - [APC2_HUMAN] |
| P25054 | Adenomatous polyposis coli protein OS=Homo sapiens GN=APC PE=1 SV=2 - [APC_HUMAN] |
| P51828 | Adenylate cyclase type 7 OS=Homo sapiens GN=ADCY7 PE=2 SV=1 - [ADCY7_HUMAN] |
| O60503 | Adenylate cyclase type 9 OS=Homo sapiens GN=ADCY9 PE=1 SV=4 - [ADCY9_HUMAN] |
| O60241 | Adhesion G protein-coupled receptor B2 OS=Homo sapiens GN=ADGRB2 PE=1 SV=2 - [AGRB2_HUMAN] |
| Q9UHX3 | Adhesion G protein-coupled receptor E2 OS=Homo sapiens GN=ADGRE2 PE=1 SV=2 - [AGRE2_HUMAN] |
| Q10588 | ADP-ribosyl cyclase/cyclic ADP-ribose hydrolase 2 OS=Homo sapiens GN=BST1 PE=1 SV=2 - [BST1_HUMAN] |
| P55196 | Afadin OS=Homo sapiens GN=MLLT4 PE=1 SV=3 - [AFAD_HUMAN] |
| P55008 | Allograft inflammatory factor 1 OS=Homo sapiens GN=AIF1 PE=1 SV=1 - [AIF1_HUMAN] |
| P30533 | Alpha-2-macroglobulin receptor-associated protein OS=Homo sapiens GN=LRPAP1 PE=1 SV=1 - [AMRP_HUMAN] |
| P12814 | Alpha-actinin-1 OS=Homo sapiens GN=ACTN1 PE=1 SV=2 - [ACTN1_HUMAN] |
| P35611 | Alpha-adducin OS=Homo sapiens GN=ADD1 PE=1 SV=2 - [ADDA_HUMAN] |
| P06733 | Alpha-enolase OS=Homo sapiens GN=ENO1 PE=1 SV=2 - [ENOA_HUMAN] |
| Q969X2 | Alpha-N-acetylgalactosaminide alpha-2,6-sialyltransferase 6 OS=Homo sapiens GN=ST6GALNAC6 PE=1 SV=1 - [SIA7F_HUMAN] |
| P51172 | Amiloride-sensitive sodium channel subunit delta OS=Homo sapiens GN=SCNN1D PE=1 SV=2 - [SCNND_HUMAN] |
| Q99767 | Amyloid beta A4 precursor protein-binding family A member 2 OS=Homo sapiens GN=APBA2 PE=1 SV=3 - [APBA2_HUMAN] |
| Q8IY63 | Angiomotin-like protein 1 OS=Homo sapiens GN=AMOTL1 PE=1 SV=1 - [AMOL1_HUMAN] |
| P04083 | Annexin A1 OS=Homo sapiens GN=ANXA1 PE=1 SV=2 - [ANXA1_HUMAN] |
| P09525 | Annexin A4 OS=Homo sapiens GN=ANXA4 PE=1 SV=4 - [ANXA4_HUMAN] |
| P08758 | Annexin A5 OS=Homo sapiens GN=ANXA5 PE=1 SV=2 - [ANXA5_HUMAN] |
| P20073 | Annexin A7 OS=Homo sapiens GN=ANXA7 PE=1 SV=3 - [ANXA7_HUMAN] |
| Q9NQ90 | Anoctamin-2 OS=Homo sapiens GN=ANO2 PE=1 SV=2 - [ANO2_HUMAN] |
| Q9HCE9 | Anoctamin-8 OS=Homo sapiens GN=ANO8 PE=1 SV=3 - [ANO8_HUMAN] |
| Q8N7J2 | APC membrane recruitment protein 2 OS=Homo sapiens GN=AMER2 PE=1 SV=3 - [AMER2_HUMAN] |
| Q8N944 | APC membrane recruitment protein 3 OS=Homo sapiens GN=AMER3 PE=1 SV=2 - [AMER3_HUMAN] |
| Q96P48 | Arf-GAP with Rho-GAP domain, ANK repeat and PH domain-containing protein 1 OS=Homo sapiens GN=ARAP1 PE=1 SV=3 - [ARAP1_HUMAN] |
| Q8WWN8 | Arf-GAP with Rho-GAP domain, ANK repeat and PH domain-containing protein 3 OS=Homo sapiens GN=ARAP3 PE=1 SV=1 - [ARAP3_HUMAN] |
| Q8N5I2 | Arrestin domain-containing protein 1 OS=Homo sapiens GN=ARRDC1 PE=1 SV=1 - [ARRD1_HUMAN] |
| Q8TBH0 | Arrestin domain-containing protein 2 OS=Homo sapiens GN=ARRDC2 PE=2 SV=2 - [ARRD2_HUMAN] |
| P15848 | Arylsulfatase B OS=Homo sapiens GN=ARSB PE=1 SV=1 - [ARSB_HUMAN] |
| O14525 | Astrotactin-1 OS=Homo sapiens GN=ASTN1 PE=2 SV=3 - [ASTN1_HUMAN] |
| Q9Y2T1 | Axin-2 OS=Homo sapiens GN=AXIN2 PE=1 SV=1 - [AXIN2_HUMAN] |
| Q9HCM4 | Band 4.1-like protein 5 OS=Homo sapiens GN=EPB41L5 PE=1 SV=3 - [E41L5_HUMAN] |
| P98160 | Basement membrane-specific heparan sulfate proteoglycan core protein OS=Homo sapiens GN=HSPG2 PE=1 SV=4 - [PGBM_HUMAN] |
| Q8WV28 | B-cell linker protein OS=Homo sapiens GN=BLNK PE=1 SV=2 - [BLNK_HUMAN] |
| Q5H9F3 | BCL-6 corepressor-like protein 1 OS=Homo sapiens GN=BCORL1 PE=1 SV=1 - [BCORL_HUMAN] |
| Q8N1M1 | Bestrophin-3 OS=Homo sapiens GN=BEST3 PE=2 SV=1 - [BEST3_HUMAN] |
| Q13884 | Beta-1-syntrophin OS=Homo sapiens GN=SNTB1 PE=1 SV=3 - [SNTB1_HUMAN] |
| Q7Z7B7 | Beta-defensin 132 OS=Homo sapiens GN=DEFB132 PE=3 SV=1 - [DB132_HUMAN] |
| P13929 | Beta-enolase OS=Homo sapiens GN=ENO3 PE=1 SV=5 - [ENOB_HUMAN] |
| P32247 | Bombesin receptor subtype-3 OS=Homo sapiens GN=BRS3 PE=1 SV=1 - [BRS3_HUMAN] |
| P80723 | Brain acid soluble protein 1 OS=Homo sapiens GN=BASP1 PE=1 SV=2 - [BASP1_HUMAN] |
| P38398 | Breast cancer type 1 susceptibility protein OS=Homo sapiens GN=BRCA1 PE=1 SV=2 - [BRCA1_HUMAN] |
| Q8NCU7 | C2 calcium-dependent domain-containing protein 4A OS=Homo sapiens GN=C2CD4A PE=2 SV=2 - [C2C4A_HUMAN] |
| A6NLJ0 | C2 calcium-dependent domain-containing protein 4B OS=Homo sapiens GN=C2CD4B PE=2 SV=1 - [C2C4B_HUMAN] |
| Q8TF44 | C2 calcium-dependent domain-containing protein 4C OS=Homo sapiens GN=C2CD4C PE=1 SV=2 - [C2C4C_HUMAN] |
| Q8IZJ3 | C3 and PZP-like alpha-2-macroglobulin domain-containing protein 8 OS=Homo sapiens GN=CPAMD8 PE=1 SV=2 - [CPMD8_HUMAN] |
| P27708 | CAD protein OS=Homo sapiens GN=CAD PE=1 SV=3 - [PYR1_HUMAN] |
| P55287 | Cadherin-11 OS=Homo sapiens GN=CDH11 PE=2 SV=2 - [CAD11_HUMAN] |
| Q9H251 | Cadherin-23 OS=Homo sapiens GN=CDH23 PE=1 SV=2 - [CAD23_HUMAN] |
| Q8IXH8 | Cadherin-like protein 26 OS=Homo sapiens GN=CDH26 PE=2 SV=3 - [CAD26_HUMAN] |
| P30988 | Calcitonin receptor OS=Homo sapiens GN=CALCR PE=1 SV=2 - [CALCR_HUMAN] |
| Q96NX5 | Calcium/calmodulin-dependent protein kinase type 1G OS=Homo sapiens GN=CAMK1G PE=1 SV=3 - [KCC1G_HUMAN] |
| Q9UQM7 | Calcium/calmodulin-dependent protein kinase type II subunit alpha OS=Homo sapiens GN=CAMK2A PE=1 SV=2 - [KCC2A_HUMAN] |
| Q12791 | Calcium-activated potassium channel subunit alpha-1 OS=Homo sapiens GN=KCNMA1 PE=1 SV=2 - [KCMA1_HUMAN] |
| Q86UW7 | Calcium-dependent secretion activator 2 OS=Homo sapiens GN=CADPS2 PE=1 SV=2 - [CAPS2_HUMAN] |
| P07384 | Calpain-1 catalytic subunit OS=Homo sapiens GN=CAPN1 PE=1 SV=1 - [CAN1_HUMAN] |
| Q9HC96 | Calpain-10 OS=Homo sapiens GN=CAPN10 PE=1 SV=2 - [CAN10_HUMAN] |
| P06731 | Carcinoembryonic antigen-related cell adhesion molecule 5 OS=Homo sapiens GN=CEACAM5 PE=1 SV=3 - [CEAM5_HUMAN] |
| Q9BXL7 | Caspase recruitment domain-containing protein 11 OS=Homo sapiens GN=CARD11 PE=1 SV=3 - [CAR11_HUMAN] |
| Q9BXL6 | Caspase recruitment domain-containing protein 14 OS=Homo sapiens GN=CARD14 PE=1 SV=2 - [CAR14_HUMAN] |
| Q92851 | Caspase-10 OS=Homo sapiens GN=CASP10 PE=1 SV=3 - [CASPA_HUMAN] |
| P04040 | Catalase OS=Homo sapiens GN=CAT PE=1 SV=3 - [CATA_HUMAN] |
| P26232 | Catenin alpha-2 OS=Homo sapiens GN=CTNNA2 PE=1 SV=5 - [CTNA2_HUMAN] |
| P08311 | Cathepsin G OS=Homo sapiens GN=CTSG PE=1 SV=2 - [CATG_HUMAN] |
| P52569 | Cationic amino acid transporter 2 OS=Homo sapiens GN=SLC7A2 PE=1 SV=2 - [CTR2_HUMAN] |
| P46092 | C-C chemokine receptor type 10 OS=Homo sapiens GN=CCR10 PE=1 SV=3 - [CCR10_HUMAN] |
| P51684 | C-C chemokine receptor type 6 OS=Homo sapiens GN=CCR6 PE=2 SV=2 - [CCR6_HUMAN] |
| Q6YHK3 | CD109 antigen OS=Homo sapiens GN=CD109 PE=1 SV=2 - [CD109_HUMAN] |
| O95971 | CD160 antigen OS=Homo sapiens GN=CD160 PE=1 SV=1 - [BY55_HUMAN] |
| Q9Y5K6 | CD2-associated protein OS=Homo sapiens GN=CD2AP PE=1 SV=1 - [CD2AP_HUMAN] |
| P16070 | CD44 antigen OS=Homo sapiens GN=CD44 PE=1 SV=3 - [CD44_HUMAN] |
| Q99795 | Cell surface A33 antigen OS=Homo sapiens GN=GPA33 PE=1 SV=1 - [GPA33_HUMAN] |
| P35523 | Chloride channel protein 1 OS=Homo sapiens GN=CLCN1 PE=1 SV=3 - [CLCN1_HUMAN] |
| Q00610 | Clathrin heavy chain 1 OS=Homo sapiens GN=CLTC PE=1 SV=5 - [CLH1_HUMAN] |
| P09497 | Clathrin light chain B OS=Homo sapiens GN=CLTB PE=1 SV=1 - [CLCB_HUMAN] |
| Q6UXG3 | CMRF35-like molecule 9 OS=Homo sapiens GN=CD300LG PE=1 SV=2 - [CLM9_HUMAN] |
| P12259 | Coagulation factor V OS=Homo sapiens GN=F5 PE=1 SV=4 - [FA5_HUMAN] |
| P00451 | Coagulation factor VIII OS=Homo sapiens GN=F8 PE=1 SV=1 - [FA8_HUMAN] |
| P00748 | Coagulation factor XII OS=Homo sapiens GN=F12 PE=1 SV=3 - [FA12_HUMAN] |
| Q8IWY9 | Codanin-1 OS=Homo sapiens GN=CDAN1 PE=1 SV=4 - [CDAN1_HUMAN] |
| P23528 | Cofilin-1 OS=Homo sapiens GN=CFL1 PE=1 SV=3 - [COF1_HUMAN] |
| Q5KU26 | Collectin-12 OS=Homo sapiens GN=COLEC12 PE=1 SV=3 - [COL12_HUMAN] |
| P01024 | Complement C3 OS=Homo sapiens GN=C3 PE=1 SV=2 - [CO3_HUMAN] |
| P01031 | Complement C5 OS=Homo sapiens GN=C5 PE=1 SV=4 - [CO5_HUMAN] |
| Q9NPY3 | Complement component C1q receptor OS=Homo sapiens GN=CD93 PE=1 SV=3 - [C1QR1_HUMAN] |
| P10643 | Complement component C7 OS=Homo sapiens GN=C7 PE=1 SV=2 - [CO7_HUMAN] |
| P17927 | Complement receptor type 1 OS=Homo sapiens GN=CR1 PE=1 SV=3 - [CR1_HUMAN] |
| O94779 | Contactin-5 OS=Homo sapiens GN=CNTN5 PE=1 SV=2 - [CNTN5_HUMAN] |
| O75131 | Copine-3 OS=Homo sapiens GN=CPNE3 PE=1 SV=1 - [CPNE3_HUMAN] |
| Q04656 | Copper-transporting ATPase 1 OS=Homo sapiens GN=ATP7A PE=1 SV=3 - [ATP7A_HUMAN] |
| P31146 | Coronin-1A OS=Homo sapiens GN=CORO1A PE=1 SV=4 - [COR1A_HUMAN] |
| Q9BR76 | Coronin-1B OS=Homo sapiens GN=CORO1B PE=1 SV=1 - [COR1B_HUMAN] |
| P49238 | CX3C chemokine receptor 1 OS=Homo sapiens GN=CX3CR1 PE=1 SV=1 - [CX3C1_HUMAN] |
| P61073 | C-X-C chemokine receptor type 4 OS=Homo sapiens GN=CXCR4 PE=1 SV=1 - [CXCR4_HUMAN] |
| P13569 | Cystic fibrosis transmembrane conductance regulator OS=Homo sapiens GN=CFTR PE=1 SV=3 - [CFTR_HUMAN] |
| Q9UPY5 | Cystine/glutamate transporter OS=Homo sapiens GN=SLC7A11 PE=1 SV=1 - [XCT_HUMAN] |
| O95727 | Cytotoxic and regulatory T-cell molecule OS=Homo sapiens GN=CRTAM PE=1 SV=2 - [CRTAM_HUMAN] |
| P53355 | Death-associated protein kinase 1 OS=Homo sapiens GN=DAPK1 PE=1 SV=6 - [DAPK1_HUMAN] |
| A4D2P6 | Delphilin OS=Homo sapiens GN=GRID2IP PE=3 SV=2 - [GRD2I_HUMAN] |
| P17661 | Desmin OS=Homo sapiens GN=DES PE=1 SV=3 - [DESM_HUMAN] |
| Q14574 | Desmocollin-3 OS=Homo sapiens GN=DSC3 PE=1 SV=3 - [DSC3_HUMAN] |
| Q9Y6T7 | Diacylglycerol kinase beta OS=Homo sapiens GN=DGKB PE=2 SV=2 - [DGKB_HUMAN] |
| Q16760 | Diacylglycerol kinase delta OS=Homo sapiens GN=DGKD PE=1 SV=4 - [DGKD_HUMAN] |
| Q5KSL6 | Diacylglycerol kinase kappa OS=Homo sapiens GN=DGKK PE=1 SV=1 - [DGKK_HUMAN] |
| O94907 | Dickkopf-related protein 1 OS=Homo sapiens GN=DKK1 PE=1 SV=1 - [DKK1_HUMAN] |
| Q8TF46 | DIS3-like exonuclease 1 OS=Homo sapiens GN=DIS3L PE=1 SV=2 - [DI3L1_HUMAN] |
| P98082 | Disabled homolog 2 OS=Homo sapiens GN=DAB2 PE=1 SV=3 - [DAB2_HUMAN] |
| Q9Y4D1 | Disheveled-associated activator of morphogenesis 1 OS=Homo sapiens GN=DAAM1 PE=1 SV=2 - [DAAM1_HUMAN] |
| O14672 | Disintegrin and metalloproteinase domain-containing protein 10 OS=Homo sapiens GN=ADAM10 PE=1 SV=1 - [ADA10_HUMAN] |
| P78536 | Disintegrin and metalloproteinase domain-containing protein 17 OS=Homo sapiens GN=ADAM17 PE=1 SV=1 - [ADA17_HUMAN] |
| Q9UKJ8 | Disintegrin and metalloproteinase domain-containing protein 21 OS=Homo sapiens GN=ADAM21 PE=2 SV=2 - [ADA21_HUMAN] |
| Q92796 | Disks large homolog 3 OS=Homo sapiens GN=DLG3 PE=1 SV=2 - [DLG3_HUMAN] |
| O14490 | Disks large-associated protein 1 OS=Homo sapiens GN=DLGAP1 PE=1 SV=1 - [DLGP1_HUMAN] |
| Q9P1A6 | Disks large-associated protein 2 OS=Homo sapiens GN=DLGAP2 PE=1 SV=4 - [DLGP2_HUMAN] |
| O95886 | Disks large-associated protein 3 OS=Homo sapiens GN=DLGAP3 PE=1 SV=3 - [DLGP3_HUMAN] |
| O60469 | Down syndrome cell adhesion molecule OS=Homo sapiens GN=DSCAM PE=1 SV=2 - [DSCAM_HUMAN] |
| Q8TD84 | Down syndrome cell adhesion molecule-like protein 1 OS=Homo sapiens GN=DSCAML1 PE=1 SV=2 - [DSCL1_HUMAN] |
| Q8N1N2 | Dynactin-associated protein OS=Homo sapiens GN=DYNAP PE=1 SV=1 - [DYNAP_HUMAN] |
| Q05193 | Dynamin-1 OS=Homo sapiens GN=DNM1 PE=1 SV=2 - [DYN1_HUMAN] |
| Q96M86 | Dynein heavy chain domain-containing protein 1 OS=Homo sapiens GN=DNHD1 PE=2 SV=2 - [DNHD1_HUMAN] |
| Q9GZS0 | Dynein intermediate chain 2, axonemal OS=Homo sapiens GN=DNAI2 PE=1 SV=2 - [DNAI2_HUMAN] |
| O75923 | Dysferlin OS=Homo sapiens GN=DYSF PE=1 SV=1 - [DYSF_HUMAN] |
| Q03001 | Dystonin OS=Homo sapiens GN=DST PE=1 SV=4 - [DYST_HUMAN] |
| P11532 | Dystrophin OS=Homo sapiens GN=DMD PE=1 SV=3 - [DMD_HUMAN] |
| A2CJ06 | Dystrotelin OS=Homo sapiens GN=DYTN PE=2 SV=1 - [DYTN_HUMAN] |
| Q15075 | Early endosome antigen 1 OS=Homo sapiens GN=EEA1 PE=1 SV=2 - [EEA1_HUMAN] |
| Q14244 | Ensconsin OS=Homo sapiens GN=MAP7 PE=1 SV=1 - [MAP7_HUMAN] |
| P29320 | Ephrin type-A receptor 3 OS=Homo sapiens GN=EPHA3 PE=1 SV=2 - [EPHA3_HUMAN] |
| Q15375 | Ephrin type-A receptor 7 OS=Homo sapiens GN=EPHA7 PE=1 SV=3 - [EPHA7_HUMAN] |
| P29323 | Ephrin type-B receptor 2 OS=Homo sapiens GN=EPHB2 PE=1 SV=5 - [EPHB2_HUMAN] |
| P98172 | Ephrin-B1 OS=Homo sapiens GN=EFNB1 PE=1 SV=1 - [EFNB1_HUMAN] |
| Q9UBC2 | Epidermal growth factor receptor substrate 15-like 1 OS=Homo sapiens GN=EPS15L1 PE=1 SV=1 - [EP15R_HUMAN] |
| P16452 | Erythrocyte membrane protein band 4.2 OS=Homo sapiens GN=EPB42 PE=1 SV=3 - [EPB42_HUMAN] |
| Q8IWU5 | Extracellular sulfatase Sulf-2 OS=Homo sapiens GN=SULF2 PE=1 SV=1 - [SULF2_HUMAN] |
| P15311 | Ezrin OS=Homo sapiens GN=EZR PE=1 SV=4 - [EZRI_HUMAN] |
| P08F94 | Fibrocystin OS=Homo sapiens GN=PKHD1 PE=1 SV=1 - [PKHD1_HUMAN] |
| P21333 | Filamin-A OS=Homo sapiens GN=FLNA PE=1 SV=4 - [FLNA_HUMAN] |
| O75369 | Filamin-B OS=Homo sapiens GN=FLNB PE=1 SV=2 - [FLNB_HUMAN] |
| Q9P278 | Folliculin-interacting protein 2 OS=Homo sapiens GN=FNIP2 PE=1 SV=2 - [FNIP2_HUMAN] |
| Q68DA7 | Formin-1 OS=Homo sapiens GN=FMN1 PE=1 SV=3 - [FMN1_HUMAN] |
| Q8IVF7 | Formin-like protein 3 OS=Homo sapiens GN=FMNL3 PE=1 SV=3 - [FMNL3_HUMAN] |
| Q7Z2K8 | G protein-regulated inducer of neurite outgrowth 1 OS=Homo sapiens GN=GPRIN1 PE=2 SV=2 - [GRIN1_HUMAN] |
| P48169 | Gamma-aminobutyric acid receptor subunit alpha-4 OS=Homo sapiens GN=GABRA4 PE=2 SV=2 - [GBRA4_HUMAN] |
| Q99928 | Gamma-aminobutyric acid receptor subunit gamma-3 OS=Homo sapiens GN=GABRG3 PE=2 SV=2 - [GBRG3_HUMAN] |
| Q9UJ14 | Gamma-glutamyltransferase 7 OS=Homo sapiens GN=GGT7 PE=1 SV=2 - [GGT7_HUMAN] |
| Q9BX51 | Gamma-glutamyltransferase light chain 1 OS=Homo sapiens GN=GGTLC1 PE=2 SV=2 - [GGTL1_HUMAN] |
| Q969M2 | Gap junction alpha-10 protein OS=Homo sapiens GN=GJA10 PE=2 SV=1 - [CXA10_HUMAN] |
| O95452 | Gap junction beta-6 protein OS=Homo sapiens GN=GJB6 PE=1 SV=2 - [CXB6_HUMAN] |
| Q3V6T2 | Girdin OS=Homo sapiens GN=CCDC88A PE=1 SV=2 - [GRDN_HUMAN] |
| P48058 | Glutamate receptor 4 OS=Homo sapiens GN=GRIA4 PE=2 SV=2 - [GRIA4_HUMAN] |
| Q12879 | Glutamate receptor ionotropic, NMDA 2A OS=Homo sapiens GN=GRIN2A PE=1 SV=1 - [NMDE1_HUMAN] |
| Q9Y3R0 | Glutamate receptor-interacting protein 1 OS=Homo sapiens GN=GRIP1 PE=1 SV=3 - [GRIP1_HUMAN] |
| Q9C0E4 | Glutamate receptor-interacting protein 2 OS=Homo sapiens GN=GRIP2 PE=1 SV=3 - [GRIP2_HUMAN] |
| O75311 | Glycine receptor subunit alpha-3 OS=Homo sapiens GN=GLRA3 PE=2 SV=2 - [GLRA3_HUMAN] |
| P46091 | G-protein coupled receptor 1 OS=Homo sapiens GN=GPR1 PE=1 SV=2 - [GPR1_HUMAN] |
| Q96PE1 | G-protein coupled receptor 124 OS=Homo sapiens GN=GPR124 PE=1 SV=2 - [GP124_HUMAN] |
| Q8WXG9 | G-protein coupled receptor 98 OS=Homo sapiens GN=GPR98 PE=1 SV=2 - [GPR98_HUMAN] |
| Q9UQC2 | GRB2-associated-binding protein 2 OS=Homo sapiens GN=GAB2 PE=1 SV=1 - [GAB2_HUMAN] |
| O95661 | GTP-binding protein Di-Ras3 OS=Homo sapiens GN=DIRAS3 PE=1 SV=1 - [DIRA3_HUMAN] |
| P55042 | GTP-binding protein RAD OS=Homo sapiens GN=RRAD PE=1 SV=2 - [RAD_HUMAN] |
| Q96QV1 | Hedgehog-interacting protein OS=Homo sapiens GN=HHIP PE=1 SV=3 - [HHIP_HUMAN] |
| P08581 | Hepatocyte growth factor receptor OS=Homo sapiens GN=MET PE=1 SV=4 - [MET_HUMAN] |
| Q8WWV6 | High affinity immunoglobulin alpha and immunoglobulin mu Fc receptor OS=Homo sapiens GN=FCAMR PE=1 SV=1 - [FCAMR_HUMAN] |
| Q9H3N8 | Histamine H4 receptor OS=Homo sapiens GN=HRH4 PE=1 SV=2 - [HRH4_HUMAN] |
| P16188 | HLA class I histocompatibility antigen, A-30 alpha chain OS=Homo sapiens GN=HLA-A PE=1 SV=2 - [1A30_HUMAN] |
| P30459 | HLA class I histocompatibility antigen, A-74 alpha chain OS=Homo sapiens GN=HLA-A PE=1 SV=1 - [1A74_HUMAN] |
| P01889 | HLA class I histocompatibility antigen, B-7 alpha chain OS=Homo sapiens GN=HLA-B PE=1 SV=3 - [1B07_HUMAN] |
| P30504 | HLA class I histocompatibility antigen, Cw-4 alpha chain OS=Homo sapiens GN=HLA-C PE=1 SV=1 - [1C04_HUMAN] |
| P01909 | HLA class II histocompatibility antigen, DQ alpha 1 chain OS=Homo sapiens GN=HLA-DQA1 PE=1 SV=1 - [DQA1_HUMAN] |
| Q86YZ3 | Hornerin OS=Homo sapiens GN=HRNR PE=1 SV=2 - [HORN_HUMAN] |
| Q92819 | Hyaluronan synthase 2 OS=Homo sapiens GN=HAS2 PE=2 SV=1 - [HYAS2_HUMAN] |
| Q5DX21 | Immunoglobulin superfamily member 11 OS=Homo sapiens GN=IGSF11 PE=2 SV=3 - [IGS11_HUMAN] |
| Q93033 | Immunoglobulin superfamily member 2 OS=Homo sapiens GN=CD101 PE=1 SV=2 - [IGSF2_HUMAN] |
| Q9NSI5 | Immunoglobulin superfamily member 5 OS=Homo sapiens GN=IGSF5 PE=2 SV=2 - [IGSF5_HUMAN] |
| Q969P0 | Immunoglobulin superfamily member 8 OS=Homo sapiens GN=IGSF8 PE=1 SV=1 - [IGSF8_HUMAN] |
| Q86SU0 | Immunoglobulin-like domain-containing receptor 1 OS=Homo sapiens GN=ILDR1 PE=1 SV=2 - [ILDR1_HUMAN] |
| Q9H160 | Inhibitor of growth protein 2 OS=Homo sapiens GN=ING2 PE=1 SV=2 - [ING2_HUMAN] |
| P06213 | Insulin receptor OS=Homo sapiens GN=INSR PE=1 SV=4 - [INSR_HUMAN] |
| P35568 | Insulin receptor substrate 1 OS=Homo sapiens GN=IRS1 PE=1 SV=1 - [IRS1_HUMAN] |
| Q9Y4H2 | Insulin receptor substrate 2 OS=Homo sapiens GN=IRS2 PE=1 SV=2 - [IRS2_HUMAN] |
| P08069 | Insulin-like growth factor 1 receptor OS=Homo sapiens GN=IGF1R PE=1 SV=1 - [IGF1R_HUMAN] |
| Q9NQX7 | Integral membrane protein 2C OS=Homo sapiens GN=ITM2C PE=1 SV=1 - [ITM2C_HUMAN] |
| P23229 | Integrin alpha-6 OS=Homo sapiens GN=ITGA6 PE=1 SV=5 - [ITA6_HUMAN] |
| Q13683 | Integrin alpha-7 OS=Homo sapiens GN=ITGA7 PE=1 SV=3 - [ITA7_HUMAN] |
| Q13349 | Integrin alpha-D OS=Homo sapiens GN=ITGAD PE=1 SV=2 - [ITAD_HUMAN] |
| P06756 | Integrin alpha-V OS=Homo sapiens GN=ITGAV PE=1 SV=2 - [ITAV_HUMAN] |
| P05556 | Integrin beta-1 OS=Homo sapiens GN=ITGB1 PE=1 SV=2 - [ITB1_HUMAN] |
| P05106 | Integrin beta-3 OS=Homo sapiens GN=ITGB3 PE=1 SV=2 - [ITB3_HUMAN] |
| P16144 | Integrin beta-4 OS=Homo sapiens GN=ITGB4 PE=1 SV=5 - [ITB4_HUMAN] |
| P26012 | Integrin beta-8 OS=Homo sapiens GN=ITGB8 PE=2 SV=1 - [ITB8_HUMAN] |
| P01579 | Interferon gamma OS=Homo sapiens GN=IFNG PE=1 SV=1 - [IFNG_HUMAN] |
| Q16552 | Interleukin-17A OS=Homo sapiens GN=IL17A PE=1 SV=1 - [IL17_HUMAN] |
| Q13478 | Interleukin-18 receptor 1 OS=Homo sapiens GN=IL18R1 PE=1 SV=1 - [IL18R_HUMAN] |
| O15554 | Intermediate conductance calcium-activated potassium channel protein 4 OS=Homo sapiens GN=KCNN4 PE=1 SV=1 - [KCNN4_HUMAN] |
| Q15811 | Intersectin-1 OS=Homo sapiens GN=ITSN1 PE=1 SV=3 - [ITSN1_HUMAN] |
| P57087 | Junctional adhesion molecule B OS=Homo sapiens GN=JAM2 PE=1 SV=1 - [JAM2_HUMAN] |
| Q8N9B5 | Junction-mediating and -regulatory protein OS=Homo sapiens GN=JMY PE=1 SV=2 - [JMY_HUMAN] |
| O75449 | Katanin p60 ATPase-containing subunit A1 OS=Homo sapiens GN=KATNA1 PE=1 SV=1 - [KTNA1_HUMAN] |
| Q07666 | KH domain-containing, RNA-binding, signal transduction-associated protein 1 OS=Homo sapiens GN=KHDRBS1 PE=1 SV=1 - [KHDR1_HUMAN] |
| Q6UWL6 | Kin of IRRE-like protein 2 OS=Homo sapiens GN=KIRREL2 PE=1 SV=2 - [KIRR2_HUMAN] |
| P01042 | Kininogen-1 OS=Homo sapiens GN=KNG1 PE=1 SV=2 - [KNG1_HUMAN] |
| A6PVL3 | Kinocilin OS=Homo sapiens GN=KNCN PE=2 SV=1 - [KNCN_HUMAN] |
| Q9NS86 | LanC-like protein 2 OS=Homo sapiens GN=LANCL2 PE=1 SV=1 - [LANC2_HUMAN] |
| Q01650 | Large neutral amino acids transporter small subunit 1 OS=Homo sapiens GN=SLC7A5 PE=1 SV=2 - [LAT1_HUMAN] |
| Q9HAR2 | Latrophilin-3 OS=Homo sapiens GN=LPHN3 PE=2 SV=2 - [LPHN3_HUMAN] |
| Q5TDP6 | Lengsin OS=Homo sapiens GN=LGSN PE=1 SV=1 - [LGSN_HUMAN] |
| Q6P1M3 | Lethal(2) giant larvae protein homolog 2 OS=Homo sapiens GN=LLGL2 PE=1 SV=2 - [L2GL2_HUMAN] |
| P42702 | Leukemia inhibitory factor receptor OS=Homo sapiens GN=LIFR PE=1 SV=1 - [LIFR_HUMAN] |
| P16150 | Leukosialin OS=Homo sapiens GN=SPN PE=1 SV=1 - [LEUK_HUMAN] |
| Q7Z4I7 | LIM and senescent cell antigen-like-containing domain protein 2 OS=Homo sapiens GN=LIMS2 PE=1 SV=1 - [LIMS2_HUMAN] |
| P06858 | Lipoprotein lipase OS=Homo sapiens GN=LPL PE=1 SV=1 - [LIPL_HUMAN] |
| O75335 | Liprin-alpha-4 OS=Homo sapiens GN=PPFIA4 PE=2 SV=3 - [LIPA4_HUMAN] |
| P01130 | Low-density lipoprotein receptor OS=Homo sapiens GN=LDLR PE=1 SV=1 - [LDLR_HUMAN] |
| P98164 | Low-density lipoprotein receptor-related protein 2 OS=Homo sapiens GN=LRP2 PE=1 SV=3 - [LRP2_HUMAN] |
| Q6UWN0 | Ly6/PLAUR domain-containing protein 4 OS=Homo sapiens GN=LYPD4 PE=2 SV=2 - [LYPD4_HUMAN] |
| O60449 | Lymphocyte antigen 75 OS=Homo sapiens GN=LY75 PE=1 SV=3 - [LY75_HUMAN] |
| Q13094 | Lymphocyte cytosolic protein 2 OS=Homo sapiens GN=LCP2 PE=1 SV=1 - [LCP2_HUMAN] |
| P22897 | Macrophage mannose receptor 1 OS=Homo sapiens GN=MRC1 PE=1 SV=1 - [MRC1_HUMAN] |
| Q8WXG6 | MAP kinase-activating death domain protein OS=Homo sapiens GN=MADD PE=1 SV=2 - [MADD_HUMAN] |
| Q14680 | Maternal embryonic leucine zipper kinase OS=Homo sapiens GN=MELK PE=1 SV=3 - [MELK_HUMAN] |
| O15232 | Matrilin-3 OS=Homo sapiens GN=MATN3 PE=1 SV=2 - [MATN3_HUMAN] |
| P50281 | Matrix metalloproteinase-14 OS=Homo sapiens GN=MMP14 PE=1 SV=3 - [MMP14_HUMAN] |
| Q9UHM6 | Melanopsin OS=Homo sapiens GN=OPN4 PE=1 SV=1 - [OPN4_HUMAN] |
| Q5TCQ9 | Membrane-associated guanylate kinase, WW and PDZ domain-containing protein 3 OS=Homo sapiens GN=MAGI3 PE=1 SV=2 - [MAGI3_HUMAN] |
| Q16819 | Meprin A subunit alpha OS=Homo sapiens GN=MEP1A PE=1 SV=2 - [MEP1A_HUMAN] |
| Q14833 | Metabotropic glutamate receptor 4 OS=Homo sapiens GN=GRM4 PE=2 SV=1 - [GRM4_HUMAN] |
| P41594 | Metabotropic glutamate receptor 5 OS=Homo sapiens GN=GRM5 PE=1 SV=2 - [GRM5_HUMAN] |
| Q9H8M5 | Metal transporter CNNM2 OS=Homo sapiens GN=CNNM2 PE=1 SV=2 - [CNNM2_HUMAN] |
| Q6P4Q7 | Metal transporter CNNM4 OS=Homo sapiens GN=CNNM4 PE=1 SV=3 - [CNNM4_HUMAN] |
| Q687X5 | Metalloreductase STEAP4 OS=Homo sapiens GN=STEAP4 PE=1 SV=1 - [STEA4_HUMAN] |
| Q9Y4B5 | Microtubule cross-linking factor 1 OS=Homo sapiens GN=MTCL1 PE=1 SV=5 - [MTCL1_HUMAN] |
| Q9UPN3 | Microtubule-actin cross-linking factor 1, isoforms 1/2/3/5 OS=Homo sapiens GN=MACF1 PE=1 SV=4 - [MACF1_HUMAN] |
| P27816 | Microtubule-associated protein 4 OS=Homo sapiens GN=MAP4 PE=1 SV=3 - [MAP4_HUMAN] |
| O15021 | Microtubule-associated serine/threonine-protein kinase 4 OS=Homo sapiens GN=MAST4 PE=1 SV=3 - [MAST4_HUMAN] |
| Q8WXI7 | Mucin-16 OS=Homo sapiens GN=MUC16 PE=1 SV=2 - [MUC16_HUMAN] |
| Q86VL8 | Multidrug and toxin extrusion protein 2 OS=Homo sapiens GN=SLC47A2 PE=1 SV=1 - [S47A2_HUMAN] |
| P21439 | Multidrug resistance protein 3 OS=Homo sapiens GN=ABCB4 PE=1 SV=2 - [MDR3_HUMAN] |
| O75970 | Multiple PDZ domain protein OS=Homo sapiens GN=MPDZ PE=1 SV=2 - [MPDZ_HUMAN] |
| O15146 | Muscle, skeletal receptor tyrosine-protein kinase OS=Homo sapiens GN=MUSK PE=1 SV=1 - [MUSK_HUMAN] |
| Q9NZM1 | Myoferlin OS=Homo sapiens GN=MYOF PE=1 SV=1 - [MYOF_HUMAN] |
| P35580 | Myosin-10 OS=Homo sapiens GN=MYH10 PE=1 SV=3 - [MYH10_HUMAN] |
| P35579 | Myosin-9 OS=Homo sapiens GN=MYH9 PE=1 SV=4 - [MYH9_HUMAN] |
| Q13496 | Myotubularin OS=Homo sapiens GN=MTM1 PE=1 SV=2 - [MTM1_HUMAN] |
| Q6T4R5 | Nance-Horan syndrome protein OS=Homo sapiens GN=NHS PE=1 SV=2 - [NHS_HUMAN] |
| Q8NF91 | Nesprin-1 OS=Homo sapiens GN=SYNE1 PE=1 SV=4 - [SYNE1_HUMAN] |
| Q8WXH0 | Nesprin-2 OS=Homo sapiens GN=SYNE2 PE=1 SV=3 - [SYNE2_HUMAN] |
| O95185 | Netrin receptor UNC5C OS=Homo sapiens GN=UNC5C PE=2 SV=2 - [UNC5C_HUMAN] |
| Q8NC67 | Neuropilin and tolloid-like protein 2 OS=Homo sapiens GN=NETO2 PE=1 SV=1 - [NETO2_HUMAN] |
| Q9P121 | Neurotrimin OS=Homo sapiens GN=NTM PE=1 SV=1 - [NTRI_HUMAN] |
| P29474 | Nitric oxide synthase, endothelial OS=Homo sapiens GN=NOS3 PE=1 SV=3 - [NOS3_HUMAN] |
| P26717 | NKG2-C type II integral membrane protein OS=Homo sapiens GN=KLRC2 PE=1 SV=2 - [NKG2C_HUMAN] |
| Q96PE5 | Opalin OS=Homo sapiens GN=OPALIN PE=2 SV=1 - [OPALI_HUMAN] |
| Q9HC10 | Otoferlin OS=Homo sapiens GN=OTOF PE=1 SV=3 - [OTOF_HUMAN] |
| Q6ZRI0 | Otogelin OS=Homo sapiens GN=OTOG PE=1 SV=3 - [OTOG_HUMAN] |
| A5PKW4 | PH and SEC7 domain-containing protein 1 OS=Homo sapiens GN=PSD PE=1 SV=2 - [PSD1_HUMAN] |
| Q9NYI0 | PH and SEC7 domain-containing protein 3 OS=Homo sapiens GN=PSD3 PE=1 SV=2 - [PSD3_HUMAN] |
| O60346 | PH domain leucine-rich repeat-containing protein phosphatase 1 OS=Homo sapiens GN=PHLPP1 PE=1 SV=3 - [PHLP1_HUMAN] |
| Q70Z35 | Phosphatidylinositol 3,4,5-trisphosphate-dependent Rac exchanger 2 protein OS=Homo sapiens GN=PREX2 PE=2 SV=1 - [PREX2_HUMAN] |
| P42336 | Phosphatidylinositol 4,5-bisphosphate 3-kinase catalytic subunit alpha isoform OS=Homo sapiens GN=PIK3CA PE=1 SV=2 - [PK3CA_HUMAN] |
| P42356 | Phosphatidylinositol 4-kinase alpha OS=Homo sapiens GN=PI4KA PE=1 SV=3 - [PI4KA_HUMAN] |
| Q9BTU6 | Phosphatidylinositol 4-kinase type 2-alpha OS=Homo sapiens GN=PI4K2A PE=1 SV=1 - [P4K2A_HUMAN] |
| O00443 | Phosphatidylinositol 4-phosphate 3-kinase C2 domain-containing subunit alpha OS=Homo sapiens GN=PIK3C2A PE=1 SV=2 - [P3C2A_HUMAN] |
| O00750 | Phosphatidylinositol 4-phosphate 3-kinase C2 domain-containing subunit beta OS=Homo sapiens GN=PIK3C2B PE=1 SV=2 - [P3C2B_HUMAN] |
| O75747 | Phosphatidylinositol 4-phosphate 3-kinase C2 domain-containing subunit gamma OS=Homo sapiens GN=PIK3C2G PE=1 SV=3 - [P3C2G_HUMAN] |
| Q99755 | Phosphatidylinositol 4-phosphate 5-kinase type-1 alpha OS=Homo sapiens GN=PIP5K1A PE=1 SV=1 - [PI51A_HUMAN] |
| Q9H307 | Pinin OS=Homo sapiens GN=PNN PE=1 SV=4 - [PININ_HUMAN] |
| Q16720 | Plasma membrane calcium-transporting ATPase 3 OS=Homo sapiens GN=ATP2B3 PE=1 SV=3 - [AT2B3_HUMAN] |
| P23634 | Plasma membrane calcium-transporting ATPase 4 OS=Homo sapiens GN=ATP2B4 PE=1 SV=2 - [AT2B4_HUMAN] |
| P00747 | Plasminogen OS=Homo sapiens GN=PLG PE=1 SV=2 - [PLMN_HUMAN] |
| Q9HBL7 | Plasminogen receptor (KT) OS=Homo sapiens GN=PLGRKT PE=1 SV=1 - [PLRKT_HUMAN] |
| Q9HCN6 | Platelet glycoprotein VI OS=Homo sapiens GN=GP6 PE=1 SV=4 - [GPVI_HUMAN] |
| P16234 | Platelet-derived growth factor receptor alpha OS=Homo sapiens GN=PDGFRA PE=1 SV=1 - [PGFRA_HUMAN] |
| Q9HCM2 | Plexin-A4 OS=Homo sapiens GN=PLXNA4 PE=1 SV=4 - [PLXA4_HUMAN] |
| O15031 | Plexin-B2 OS=Homo sapiens GN=PLXNB2 PE=1 SV=3 - [PLXB2_HUMAN] |
| Q9Y4D7 | Plexin-D1 OS=Homo sapiens GN=PLXND1 PE=1 SV=3 - [PLXD1_HUMAN] |
| Q8TDX9 | Polycystic kidney disease protein 1-like 1 OS=Homo sapiens GN=PKD1L1 PE=1 SV=1 - [PK1L1_HUMAN] |
| Q7Z443 | Polycystic kidney disease protein 1-like 3 OS=Homo sapiens GN=PKD1L3 PE=1 SV=1 - [PK1L3_HUMAN] |
| A8MYU2 | Potassium channel subfamily U member 1 OS=Homo sapiens GN=KCNU1 PE=1 SV=2 - [KCNU1_HUMAN] |
| Q14721 | Potassium voltage-gated channel subfamily B member 1 OS=Homo sapiens GN=KCNB1 PE=1 SV=2 - [KCNB1_HUMAN] |
| Q03721 | Potassium voltage-gated channel subfamily C member 4 OS=Homo sapiens GN=KCNC4 PE=1 SV=2 - [KCNC4_HUMAN] |
| Q8TAE7 | Potassium voltage-gated channel subfamily G member 3 OS=Homo sapiens GN=KCNG3 PE=1 SV=1 - [KCNG3_HUMAN] |
| Q8NCM2 | Potassium voltage-gated channel subfamily H member 5 OS=Homo sapiens GN=KCNH5 PE=1 SV=3 - [KCNH5_HUMAN] |
| Q9NS40 | Potassium voltage-gated channel subfamily H member 7 OS=Homo sapiens GN=KCNH7 PE=2 SV=2 - [KCNH7_HUMAN] |
| Q9NR82 | Potassium voltage-gated channel subfamily KQT member 5 OS=Homo sapiens GN=KCNQ5 PE=1 SV=3 - [KCNQ5_HUMAN] |
| O60741 | Potassium/sodium hyperpolarization-activated cyclic nucleotide-gated channel 1 OS=Homo sapiens GN=HCN1 PE=1 SV=3 - [HCN1_HUMAN] |
| Q9UL51 | Potassium/sodium hyperpolarization-activated cyclic nucleotide-gated channel 2 OS=Homo sapiens GN=HCN2 PE=1 SV=3 - [HCN2_HUMAN] |
| P54707 | Potassium-transporting ATPase alpha chain 2 OS=Homo sapiens GN=ATP12A PE=1 SV=3 - [AT12A_HUMAN] |
| P01133 | Pro-epidermal growth factor OS=Homo sapiens GN=EGF PE=1 SV=2 - [EGF_HUMAN] |
| Q14005 | Pro-interleukin-16 OS=Homo sapiens GN=IL16 PE=1 SV=4 - [IL16_HUMAN] |
| O14511 | Pro-neuregulin-2, membrane-bound isoform OS=Homo sapiens GN=NRG2 PE=1 SV=1 - [NRG2_HUMAN] |
| Q8NBP7 | Proprotein convertase subtilisin/kexin type 9 OS=Homo sapiens GN=PCSK9 PE=1 SV=3 - [PCSK9_HUMAN] |
| Q16186 | Proteasomal ubiquitin receptor ADRM1 OS=Homo sapiens GN=ADRM1 PE=1 SV=2 - [ADRM1_HUMAN] |
| Q8IVF2 | Protein AHNAK2 OS=Homo sapiens GN=AHNAK2 PE=1 SV=2 - [AHNK2_HUMAN] |
| P02760 | Protein AMBP OS=Homo sapiens GN=AMBP PE=1 SV=1 - [AMBP_HUMAN] |
| Q9UPA5 | Protein bassoon OS=Homo sapiens GN=BSN PE=2 SV=4 - [BSN_HUMAN] |
| Q9ULD6 | Protein inturned OS=Homo sapiens GN=INTU PE=2 SV=2 - [INTU_HUMAN] |
| Q05655 | Protein kinase C delta type OS=Homo sapiens GN=PRKCD PE=1 SV=2 - [KPCD_HUMAN] |
| Q96RT1 | Protein LAP2 OS=Homo sapiens GN=ERBB2IP PE=1 SV=2 - [LAP2_HUMAN] |
| Q3SYG4 | Protein PTHB1 OS=Homo sapiens GN=BBS9 PE=1 SV=1 - [PTHB1_HUMAN] |
| Q9HCY8 | Protein S100-A14 OS=Homo sapiens GN=S100A14 PE=1 SV=1 - [S10AE_HUMAN] |
| P06703 | Protein S100-A6 OS=Homo sapiens GN=S100A6 PE=1 SV=1 - [S10A6_HUMAN] |
| P05109 | Protein S100-A8 OS=Homo sapiens GN=S100A8 PE=1 SV=1 - [S10A8_HUMAN] |
| P06702 | Protein S100-A9 OS=Homo sapiens GN=S100A9 PE=1 SV=1 - [S10A9_HUMAN] |
| P25815 | Protein S100-P OS=Homo sapiens GN=S100P PE=1 SV=2 - [S100P_HUMAN] |
| Q8TF72 | Protein Shroom3 OS=Homo sapiens GN=SHROOM3 PE=1 SV=2 - [SHRM3_HUMAN] |
| O94964 | Protein SOGA1 OS=Homo sapiens GN=SOGA1 PE=1 SV=2 - [SOGA1_HUMAN] |
| Q8WWL2 | Protein spire homolog 2 OS=Homo sapiens GN=SPIRE2 PE=1 SV=3 - [SPIR2_HUMAN] |
| Q9C0D5 | Protein TANC1 OS=Homo sapiens GN=TANC1 PE=1 SV=3 - [TANC1_HUMAN] |
| Q9UPX0 | Protein turtle homolog B OS=Homo sapiens GN=IGSF9B PE=2 SV=2 - [TUTLB_HUMAN] |
| Q9P2D8 | Protein unc-79 homolog OS=Homo sapiens GN=UNC79 PE=2 SV=4 - [UNC79_HUMAN] |
| Q8N2C7 | Protein unc-80 homolog OS=Homo sapiens GN=UNC80 PE=2 SV=2 - [UNC80_HUMAN] |
| P55085 | Proteinase-activated receptor 2 OS=Homo sapiens GN=F2RL1 PE=1 SV=1 - [PAR2_HUMAN] |
| P20396 | Pro-thyrotropin-releasing hormone OS=Homo sapiens GN=TRH PE=1 SV=1 - [TRH_HUMAN] |
| Q9Y5I1 | Protocadherin alpha-11 OS=Homo sapiens GN=PCDHA11 PE=2 SV=1 - [PCDAB_HUMAN] |
| Q9UN72 | Protocadherin alpha-7 OS=Homo sapiens GN=PCDHA7 PE=2 SV=1 - [PCDA7_HUMAN] |
| Q14517 | Protocadherin Fat 1 OS=Homo sapiens GN=FAT1 PE=1 SV=2 - [FAT1_HUMAN] |
| Q9NYQ8 | Protocadherin Fat 2 OS=Homo sapiens GN=FAT2 PE=1 SV=2 - [FAT2_HUMAN] |
| Q08174 | Protocadherin-1 OS=Homo sapiens GN=PCDH1 PE=1 SV=2 - [PCDH1_HUMAN] |
| O14917 | Protocadherin-17 OS=Homo sapiens GN=PCDH17 PE=2 SV=2 - [PCD17_HUMAN] |
| Q9HCL0 | Protocadherin-18 OS=Homo sapiens GN=PCDH18 PE=2 SV=3 - [PCD18_HUMAN] |
| Q8TAB3 | Protocadherin-19 OS=Homo sapiens GN=PCDH19 PE=1 SV=3 - [PCD19_HUMAN] |
| Q96QE2 | Proton myo-inositol cotransporter OS=Homo sapiens GN=SLC2A13 PE=1 SV=3 - [MYCT_HUMAN] |
| P16109 | P-selectin OS=Homo sapiens GN=SELP PE=1 SV=3 - [LYAM3_HUMAN] |
| P14618 | Pyruvate kinase PKM OS=Homo sapiens GN=PKM PE=1 SV=4 - [KPYM_HUMAN] |
| Q9H1K0 | Rabenosyn-5 OS=Homo sapiens GN=RBSN PE=1 SV=2 - [RBNS5_HUMAN] |
| P31751 | RAC-beta serine/threonine-protein kinase OS=Homo sapiens GN=AKT2 PE=1 SV=2 - [AKT2_HUMAN] |
| P23468 | Receptor-type tyrosine-protein phosphatase delta OS=Homo sapiens GN=PTPRD PE=1 SV=2 - [PTPRD_HUMAN] |
| P10586 | Receptor-type tyrosine-protein phosphatase F OS=Homo sapiens GN=PTPRF PE=1 SV=2 - [PTPRF_HUMAN] |
| Q92932 | Receptor-type tyrosine-protein phosphatase N2 OS=Homo sapiens GN=PTPRN2 PE=1 SV=2 - [PTPR2_HUMAN] |
| Q16827 | Receptor-type tyrosine-protein phosphatase O OS=Homo sapiens GN=PTPRO PE=1 SV=2 - [PTPRO_HUMAN] |
| O14924 | Regulator of G-protein signaling 12 OS=Homo sapiens GN=RGS12 PE=1 SV=1 - [RGS12_HUMAN] |
| P49796 | Regulator of G-protein signaling 3 OS=Homo sapiens GN=RGS3 PE=1 SV=2 - [RGS3_HUMAN] |
| O75787 | Renin receptor OS=Homo sapiens GN=ATP6AP2 PE=1 SV=2 - [RENR_HUMAN] |
| Q9NQC3 | Reticulon-4 OS=Homo sapiens GN=RTN4 PE=1 SV=2 - [RTN4_HUMAN] |
| P78363 | Retinal-specific ATP-binding cassette transporter OS=Homo sapiens GN=ABCA4 PE=1 SV=3 - [ABCA4_HUMAN] |
| Q14BN4 | Sarcolemmal membrane-associated protein OS=Homo sapiens GN=SLMAP PE=1 SV=1 - [SLMAP_HUMAN] |
| O43464 | Serine protease HTRA2, mitochondrial OS=Homo sapiens GN=HTRA2 PE=1 SV=2 - [HTRA2_HUMAN] |
| Q9P0L2 | Serine/threonine-protein kinase MARK1 OS=Homo sapiens GN=MARK1 PE=1 SV=2 - [MARK1_HUMAN] |
| Q9Y5S2 | Serine/threonine-protein kinase MRCK beta OS=Homo sapiens GN=CDC42BPB PE=1 SV=2 - [MRCKB_HUMAN] |
| Q9Y566 | SH3 and multiple ankyrin repeat domains protein 1 OS=Homo sapiens GN=SHANK1 PE=1 SV=2 - [SHAN1_HUMAN] |
| Q9UPX8 | SH3 and multiple ankyrin repeat domains protein 2 OS=Homo sapiens GN=SHANK2 PE=1 SV=3 - [SHAN2_HUMAN] |
| Q5BIV9 | Shadow of prion protein OS=Homo sapiens GN=SPRN PE=2 SV=1 - [SPRN_HUMAN] |
| A6NMB1 | Sialic acid-binding Ig-like lectin 16 OS=Homo sapiens GN=SIGLEC16 PE=2 SV=3 - [SIG16_HUMAN] |
| Q9BZZ2 | Sialoadhesin OS=Homo sapiens GN=SIGLEC1 PE=1 SV=2 - [SN_HUMAN] |
| Q8IWY4 | Signal peptide, CUB and EGF-like domain-containing protein 1 OS=Homo sapiens GN=SCUBE1 PE=1 SV=3 - [SCUB1_HUMAN] |
| P52630 | Signal transducer and activator of transcription 2 OS=Homo sapiens GN=STAT2 PE=1 SV=1 - [STAT2_HUMAN] |
| O43166 | Signal-induced proliferation-associated 1-like protein 1 OS=Homo sapiens GN=SIPA1L1 PE=1 SV=4 - [SI1L1_HUMAN] |
| Q9Y5Y9 | Sodium channel protein type 10 subunit alpha OS=Homo sapiens GN=SCN10A PE=1 SV=2 - [SCNAA_HUMAN] |
| Q99250 | Sodium channel protein type 2 subunit alpha OS=Homo sapiens GN=SCN2A PE=1 SV=3 - [SCN2A_HUMAN] |
| P35499 | Sodium channel protein type 4 subunit alpha OS=Homo sapiens GN=SCN4A PE=1 SV=4 - [SCN4A_HUMAN] |
| Q14524 | Sodium channel protein type 5 subunit alpha OS=Homo sapiens GN=SCN5A PE=1 SV=2 - [SCN5A_HUMAN] |
| Q9UQD0 | Sodium channel protein type 8 subunit alpha OS=Homo sapiens GN=SCN8A PE=1 SV=1 - [SCN8A_HUMAN] |
| Q96EP9 | Sodium/bile acid cotransporter 4 OS=Homo sapiens GN=SLC10A4 PE=1 SV=2 - [NTCP4_HUMAN] |
| P53794 | Sodium/myo-inositol cotransporter OS=Homo sapiens GN=SLC5A3 PE=3 SV=2 - [SC5A3_HUMAN] |
| O60721 | Sodium/potassium/calcium exchanger 1 OS=Homo sapiens GN=SLC24A1 PE=1 SV=1 - [NCKX1_HUMAN] |
| P05026 | Sodium/potassium-transporting ATPase subunit beta-1 OS=Homo sapiens GN=ATP1B1 PE=1 SV=1 - [AT1B1_HUMAN] |
| O00624 | Sodium-dependent phosphate transport protein 3 OS=Homo sapiens GN=SLC17A2 PE=2 SV=2 - [NPT3_HUMAN] |
| Q13621 | Solute carrier family 12 member 1 OS=Homo sapiens GN=SLC12A1 PE=1 SV=2 - [S12A1_HUMAN] |
| Q9H2X9 | Solute carrier family 12 member 5 OS=Homo sapiens GN=SLC12A5 PE=2 SV=3 - [S12A5_HUMAN] |
| Q9UHW9 | Solute carrier family 12 member 6 OS=Homo sapiens GN=SLC12A6 PE=1 SV=2 - [S12A6_HUMAN] |
| Q9Y666 | Solute carrier family 12 member 7 OS=Homo sapiens GN=SLC12A7 PE=1 SV=3 - [S12A7_HUMAN] |
| Q9Y267 | Solute carrier family 22 member 14 OS=Homo sapiens GN=SLC22A14 PE=2 SV=4 - [S22AE_HUMAN] |
| Q6T423 | Solute carrier family 22 member 25 OS=Homo sapiens GN=SLC22A25 PE=2 SV=2 - [S22AP_HUMAN] |
| Q9Y694 | Solute carrier family 22 member 7 OS=Homo sapiens GN=SLC22A7 PE=1 SV=1 - [S22A7_HUMAN] |
| Q9UHI7 | Solute carrier family 23 member 1 OS=Homo sapiens GN=SLC23A1 PE=1 SV=3 - [S23A1_HUMAN] |
| Q9BXS9 | Solute carrier family 26 member 6 OS=Homo sapiens GN=SLC26A6 PE=1 SV=1 - [S26A6_HUMAN] |
| Q9Y6L6 | Solute carrier organic anion transporter family member 1B1 OS=Homo sapiens GN=SLCO1B1 PE=1 SV=2 - [SO1B1_HUMAN] |
| Q96BD0 | Solute carrier organic anion transporter family member 4A1 OS=Homo sapiens GN=SLCO4A1 PE=1 SV=2 - [SO4A1_HUMAN] |
| P30626 | Sorcin OS=Homo sapiens GN=SRI PE=1 SV=1 - [SORCN_HUMAN] |
| Q7Z614 | Sorting nexin-20 OS=Homo sapiens GN=SNX20 PE=1 SV=1 - [SNX20_HUMAN] |
| P02549 | Spectrin alpha chain, erythrocytic 1 OS=Homo sapiens GN=SPTA1 PE=1 SV=5 - [SPTA1_HUMAN] |
| Q13813 | Spectrin alpha chain, non-erythrocytic 1 OS=Homo sapiens GN=SPTAN1 PE=1 SV=3 - [SPTN1_HUMAN] |
| Q01082 | Spectrin beta chain, non-erythrocytic 1 OS=Homo sapiens GN=SPTBN1 PE=1 SV=2 - [SPTB2_HUMAN] |
| P28290 | Sperm-specific antigen 2 OS=Homo sapiens GN=SSFA2 PE=1 SV=3 - [SSFA2_HUMAN] |
| Q8WWQ8 | Stabilin-2 OS=Homo sapiens GN=STAB2 PE=1 SV=3 - [STAB2_HUMAN] |
| Q9UBI4 | Stomatin-like protein 1 OS=Homo sapiens GN=STOML1 PE=1 SV=1 - [STML1_HUMAN] |
| Q9Y5Y6 | Suppressor of tumorigenicity 14 protein OS=Homo sapiens GN=ST14 PE=1 SV=2 - [ST14_HUMAN] |
| Q5SQN1 | Synaptosomal-associated protein 47 OS=Homo sapiens GN=SNAP47 PE=1 SV=3 - [SNP47_HUMAN] |
| Q7L8C5 | Synaptotagmin-13 OS=Homo sapiens GN=SYT13 PE=1 SV=1 - [SYT13_HUMAN] |
| Q86SS6 | Synaptotagmin-9 OS=Homo sapiens GN=SYT9 PE=2 SV=1 - [SYT9_HUMAN] |
| Q4VX76 | Synaptotagmin-like protein 3 OS=Homo sapiens GN=SYTL3 PE=2 SV=3 - [SYTL3_HUMAN] |
| Q9Y6H5 | Synphilin-1 OS=Homo sapiens GN=SNCAIP PE=1 SV=2 - [SNCAP_HUMAN] |
| Q8N4C7 | Syntaxin-19 OS=Homo sapiens GN=STX19 PE=1 SV=1 - [STX19_HUMAN] |
| Q9BPZ7 | Target of rapamycin complex 2 subunit MAPKAP1 OS=Homo sapiens GN=MAPKAP1 PE=1 SV=2 - [SIN1_HUMAN] |
| Q7RTX0 | Taste receptor type 1 member 3 OS=Homo sapiens GN=TAS1R3 PE=1 SV=2 - [TS1R3_HUMAN] |
| P59551 | Taste receptor type 2 member 60 OS=Homo sapiens GN=TAS2R60 PE=2 SV=1 - [T2R60_HUMAN] |
| Q9NYW3 | Taste receptor type 2 member 7 OS=Homo sapiens GN=TAS2R7 PE=1 SV=1 - [TA2R7_HUMAN] |
| P01730 | T-cell surface glycoprotein CD4 OS=Homo sapiens GN=CD4 PE=1 SV=1 - [CD4_HUMAN] |
| Q6N022 | Teneurin-4 OS=Homo sapiens GN=TENM4 PE=1 SV=2 - [TEN4_HUMAN] |
| Q63HR2 | Tensin-2 OS=Homo sapiens GN=TNS2 PE=1 SV=2 - [TNS2_HUMAN] |
| O95049 | Tight junction protein ZO-3 OS=Homo sapiens GN=TJP3 PE=1 SV=3 - [ZO3_HUMAN] |
| O60603 | Toll-like receptor 2 OS=Homo sapiens GN=TLR2 PE=1 SV=1 - [TLR2_HUMAN] |
| Q9NYK1 | Toll-like receptor 7 OS=Homo sapiens GN=TLR7 PE=2 SV=1 - [TLR7_HUMAN] |
| Q9UKE5 | TRAF2 and NCK-interacting protein kinase OS=Homo sapiens GN=TNIK PE=1 SV=1 - [TNIK_HUMAN] |
| Q15582 | Transforming growth factor-beta-induced protein ig-h3 OS=Homo sapiens GN=TGFBI PE=1 SV=1 - [BGH3_HUMAN] |
| Q7Z2W7 | Transient receptor potential cation channel subfamily M member 8 OS=Homo sapiens GN=TRPM8 PE=1 SV=2 - [TRPM8_HUMAN] |
| A2VDJ0 | Transmembrane protein 131-like OS=Homo sapiens GN=KIAA0922 PE=1 SV=2 - [T131L_HUMAN] |
| Q86YD3 | Transmembrane protein 25 OS=Homo sapiens GN=TMEM25 PE=1 SV=1 - [TMM25_HUMAN] |
| Q9HCN3 | Transmembrane protein 8A OS=Homo sapiens GN=TMEM8A PE=1 SV=3 - [TMM8A_HUMAN] |
| Q9NP99 | Triggering receptor expressed on myeloid cells 1 OS=Homo sapiens GN=TREM1 PE=1 SV=1 - [TREM1_HUMAN] |
| Q6ZMU5 | Tripartite motif-containing protein 72 OS=Homo sapiens GN=TRIM72 PE=1 SV=2 - [TRI72_HUMAN] |
| P50591 | Tumor necrosis factor ligand superfamily member 10 OS=Homo sapiens GN=TNFSF10 PE=1 SV=1 - [TNF10_HUMAN] |
| Q06187 | Tyrosine-protein kinase BTK OS=Homo sapiens GN=BTK PE=1 SV=3 - [BTK_HUMAN] |
| Q08881 | Tyrosine-protein kinase ITK/TSK OS=Homo sapiens GN=ITK PE=1 SV=1 - [ITK_HUMAN] |
| P23458 | Tyrosine-protein kinase JAK1 OS=Homo sapiens GN=JAK1 PE=1 SV=2 - [JAK1_HUMAN] |
| Q6J9G0 | Tyrosine-protein kinase STYK1 OS=Homo sapiens GN=STYK1 PE=1 SV=4 - [STYK1_HUMAN] |
| Q05209 | Tyrosine-protein phosphatase non-receptor type 12 OS=Homo sapiens GN=PTPN12 PE=1 SV=3 - [PTN12_HUMAN] |
| Q9Y2R2 | Tyrosine-protein phosphatase non-receptor type 22 OS=Homo sapiens GN=PTPN22 PE=1 SV=2 - [PTN22_HUMAN] |
| Q03405 | Urokinase plasminogen activator surface receptor OS=Homo sapiens GN=PLAUR PE=1 SV=1 - [UPAR_HUMAN] |
| O75445 | Usherin OS=Homo sapiens GN=USH2A PE=1 SV=3 - [USH2A_HUMAN] |
| P46939 | Utrophin OS=Homo sapiens GN=UTRN PE=1 SV=2 - [UTRO_HUMAN] |
| P35968 | Vascular endothelial growth factor receptor 2 OS=Homo sapiens GN=KDR PE=1 SV=2 - [VGFR2_HUMAN] |
| Q14D04 | Ventricular zone-expressed PH domain-containing protein homolog 1 OS=Homo sapiens GN=VEPH1 PE=2 SV=1 - [MELT_HUMAN] |
| P98155 | Very low-density lipoprotein receptor OS=Homo sapiens GN=VLDLR PE=1 SV=1 - [VLDLR_HUMAN] |
| P07225 | Vitamin K-dependent protein S OS=Homo sapiens GN=PROS1 PE=1 SV=1 - [PROS_HUMAN] |
| Q9Y698 | Voltage-dependent calcium channel gamma-2 subunit OS=Homo sapiens GN=CACNG2 PE=1 SV=1 - [CCG2_HUMAN] |
| P54289 | Voltage-dependent calcium channel subunit alpha-2/delta-1 OS=Homo sapiens GN=CACNA2D1 PE=1 SV=3 - [CA2D1_HUMAN] |
| Q13936 | Voltage-dependent L-type calcium channel subunit alpha-1C OS=Homo sapiens GN=CACNA1C PE=1 SV=4 - [CAC1C_HUMAN] |
| Q01668 | Voltage-dependent L-type calcium channel subunit alpha-1D OS=Homo sapiens GN=CACNA1D PE=1 SV=2 - [CAC1D_HUMAN] |
| P54284 | Voltage-dependent L-type calcium channel subunit beta-3 OS=Homo sapiens GN=CACNB3 PE=1 SV=1 - [CACB3_HUMAN] |
| O00555 | Voltage-dependent P/Q-type calcium channel subunit alpha-1A OS=Homo sapiens GN=CACNA1A PE=1 SV=2 - [CAC1A_HUMAN] |
| Q15878 | Voltage-dependent R-type calcium channel subunit alpha-1E OS=Homo sapiens GN=CACNA1E PE=1 SV=3 - [CAC1E_HUMAN] |
| O43497 | Voltage-dependent T-type calcium channel subunit alpha-1G OS=Homo sapiens GN=CACNA1G PE=2 SV=3 - [CAC1G_HUMAN] |
| O95180 | Voltage-dependent T-type calcium channel subunit alpha-1H OS=Homo sapiens GN=CACNA1H PE=1 SV=4 - [CAC1H_HUMAN] |
| Q9P0X4 | Voltage-dependent T-type calcium channel subunit alpha-1I OS=Homo sapiens GN=CACNA1I PE=1 SV=1 - [CAC1I_HUMAN] |
| Q14722 | Voltage-gated potassium channel subunit beta-1 OS=Homo sapiens GN=KCNAB1 PE=1 SV=1 - [KCAB1_HUMAN] |
| Q8IWT6 | Volume-regulated anion channel subunit LRRC8A OS=Homo sapiens GN=LRRC8A PE=1 SV=1 - [LRC8A_HUMAN] |
| Q7L1W4 | Volume-regulated anion channel subunit LRRC8D OS=Homo sapiens GN=LRRC8D PE=1 SV=1 - [LRC8D_HUMAN] |
| Q8N8Y2 | V-type proton ATPase subunit d 2 OS=Homo sapiens GN=ATP6V0D2 PE=2 SV=1 - [VA0D2_HUMAN] |
